# Nature’s defense against emerging neurodegenerative threats: Dynamic simulation, PCA, DCCM identified potential plant-based antiviral lead targeting borna disease virus nucleoprotein

**DOI:** 10.1371/journal.pone.0310802

**Published:** 2024-12-30

**Authors:** Noimul Hasan Siddiquee, Md. Ifteker Hossain, Farhana Mansoor Priya, Sakia Binte Azam, Md. Enamul Kabir Talukder, Durjoy Barua, Salina Malek, Niloy Saha, Sidratul Muntaha, Ridoy Paul, Israt Jahan Ritu, Farjana Islam Tuly, Abir Hossain

**Affiliations:** 1 Department of Microbiology, Noakhali Science and Technology University, Noakhali, Bangladesh; 2 Bioinformatics Laboratory (BioLab), Noakhali, Bangladesh; 3 Department of Microbiology, BRAC University, Dhaka, Bangladesh; 4 Department of Genetic Engineering and Biotechnology, Jashore University of Science and Technology, Jashore, Bangladesh; 5 Department of Pharmacy, BGC Trust University, Chandanaish, Bangladesh; 6 Department of Biotechnology and Genetic Engineering, Jahangirnagar University, Savar, Dhaka; 7 Department of Biotechnology & Genetic Engineering, Noakhali Science and Technology University, Noakhali, Bangladesh; 8 Department of Microbiology, Jahangirnagar University, Savar, Dhaka, Bangladesh; 9 Department of Microbiology, University of Dhaka, Dhaka, Bangladesh; Bowen University, NIGERIA

## Abstract

The rare zoonotic Borna disease virus (BDV) causes fatal neurological disease in various animals, with a high mortality rate exceeding 90% in central Europe. However, unlike most viruses, it establishes persistent infections within the host cell nucleus, hindering treatment. As successful BDV treatments remain elusive, the researchers turned to a computational approach, utilizing molecular docking, ADME/T, post-docking MMGBSA, MD simulation, DCCM, and PCA to identify promising phytochemical drug candidates targeting the BDV Nucleoprotein (PDB ID: 1N93). From IMPPAT 1940 unique phytochemical compounds of a total of 8617 compounds from 36 Indian medicinal plants were retrieved. Three compounds were chosen as leads with higher binding affinity of -6.244, -6.116, and -6.07 kcal/mol with CID 163114683 (IMPHY000668) Nimbochalcin, CID 20871246 (IMPHY007896) 3,4-Dihydroxy-5-oxocyclohex-3-ene-1-carboxylic acid, and CID 243 (IMPHY002962) Benzoic acid. The three top compounds coordinated with the protein’s common amino acid residues at GLN 161, ARG 165, ILE 145, ILE 162, ILE 149, and VAL 229 during molecular docking, which implies that both lead compounds and the control ligand interact within the protein’s shared active site. Afterwards, negative binding free energies of Nimbochalcin, 3,4-Dihydroxy-5-oxocyclohex-3-ene-1-carboxylic acid, and Benzoic acid were -51.21, -13.94, and -22.95 kcal/mol, accordingly. Favorable Pk and toxicological characteristics are shared by all of the chosen drugs, indicating their efficacy and safety. Using MD simulation, these three compounds were further assessed, and their stability in binding to the target protein was confirmed and subsequently, DCCM and PCA analyses were carried out from MD trajectory. MD simulations found that the protein binding site is highly stable when complexed with CID 20871246 and has a higher negative binding free energy value, indicating a strong interaction between the compound and the protein. Principal component analysis (PCA) identified three main components (PC1, PC2, and PC3) that accounted for 53.43%, 12.31%, and 5.97% of the variance, respectively. These findings provide intriguing evidence that the CID 20871246-1N93 complex is more stable than the other complexes. The BDV nucleoprotein was the target of this study’s investigation where CID 20871246 (3,4-dihydroxy-5-oxocyclohex-3-ene-1-carboxylic acid) exhibited tremendous antiviral activity which is found in the flower of the plant *Mangifera indica* revealing as a possible therapeutic candidate.

## 1. Introduction

Borna disease (BD) is a neurodegenerative syndrome with immune-mediated meningoencephalitis origins that presents notable deviations in behavioural patterns and is caused by the virus, which is known for its neurotropic nature and noncytopathic infection named Borna disease virus [1–4]. The Borna disease virus (BDV), which falls within the category of enveloped, spherical virus with a diameter of 80–130 nm, non-segmented, linear negative-strand RNA viruses (nsNSVs) with about 8.9kb, is classified under the *Bornaviridae* family, order *Mononegavirales* [2, 5, 6]. Borna disease, initially termed "hot-tempered head illness," was first identified in 1885 when all horses in a cavalry unit near Borna, Germany, perished, and distinctive inclusions, now known as Joest-Degen inclusion bodies, were later discovered in the nerves of affected horses. The geographic dispersion of BDV, an infectious disease of the CNS, is confined to specific regions in central Europe. While Borna disease has only been definitively identified in central Europe (including southern and eastern Germany, Liechtenstein, Switzerland, and Austria), recent reports suggest its potential presence in North America and parts of Asia (China, Japan, Israel, and Iran) [6]. The virus is believed to persist in the natural ecosystem through a cyclical interaction involving its wild reservoir host, the eurasian bicoloured shrew (*Crocidura leucodon*), intermediate hosts such as sheep and horses, infrequently observed in Equidae, bovines, caprines, lagomorphs, and, on rare occasions, diverse groups encompassing captive and domesticated animals, such as those found at zoological facilities and serving as companions [6, 7]. Prior research has indicated that BDV can infect a broad range of mammalian species, meaning that its host range likely encompasses all warm-blooded animals, including plants. The viral infection can potentially result in mortality among individuals [2, 8]. The prevalence and clinical consequences of human BDV infection are still questioned, while some studies suggest a connection with conditions like unipolar depression, bipolar disorder, and schizophrenia. Even so, BDV has also been connected to violent brain behavior, motor neuron disease, fatigue syndrome, chronic multiple sclerosis, and AIDS encephalopathy [6]. BDV has recently emerged as a severe threat to human health. In 2018, several organ transplant recipients in Germany contracted BDV encephalitis, resulting in two deaths and one survivor with significant disabilities [9]. More recently, in 2021, three additional cases of BDV encephalitis were identified in eastern Germany [10]. The case mortality rates exhibit a significant severity level above 90% [7].

As time passes, rats, mice, and tree shrews have developed rare BDV-induced disease symptoms. These animals can develop anxiety, aggressiveness, cognitive deficits, and hyperactivity from BDV without viral encephalitis symptoms. Humans may acquire psychiatric disorders after BDV infection, according to animal studies. Depression, psychosis, and idiopathic acute or chronic encephalitis may be linked to BDV [11]. As a Neurotropic virus, BDV targets neural crest-derived CNS cells like neurons, astrocytes, ependymal cells, and oligodendrocytes. It causes abnormalities in rats, horses, and humans’ brains, livers, spleens, and kidneys. Schizophrenia, affective disorders, and personality disorders can result from BDV-induced brain shrinkage, debility, inflammation, and injury, Seizure, tremor [12]. Infection of the spinal cord by BDV damages movement and sensory nerves. Meningoencephalitis, which damages the spinal cord and peripheral nerves and results in pain and paralysis in the limbs, is brought on by BDV infection in horses and lambs. Additionally, a spleen infection can result in anemia; a kidney infection rarely causes kidney failure, and this infection can cause inflammation and liver damage, which can lead to liver failure [12–14].

Pathological signs of BDV infection vary according to host species, immunological condition, age, method of infection, and virus strain after a variable incubation period. Ataxia, paralysis, and seizures define the deadly disease in horses and sheep. It can produce depression and aggression in rats or be subclinical or moderate [15]. Studies in experimentally infected animals have shown the path to BDV infection’s clinical manifestations. The virus likely enters by olfactory nerve ends intranasally. The trigeminal nerve oral route is yet another choice. Viruses enter the nervous system and move via axons to the brain, where they replicate in neurons and glial cells, primarily in the limbic system. Over time, the virus spreads from the CNS to the peripheral nervous system and retinal neurons [6]. While the immune system generates BDV-specific antibodies and T cells, the role of antibodies in BDV infection remains unclear. This is because the robust immune response, characterized by high-titer antibodies against BDV proteins and neutralizing activity in chronically infected animals, can paradoxically cause harm. The presence of BDV antibodies in the cerebrospinal fluid (CSF) of infected animals further complicates this picture, as their specificity and impact on viral spread or replication are unknown [16]. Compared to the humoral immune response to BDV, little is known about the cellular immunological response to BDV-specific proteins. Immunization with pure protein induces CD4+ T cells for the p24 and p40 nucleoproteins, although cytolytic CD8+ T cell antigen specificity has never been studied [15, 17].

The BDV genome, with its five primary open reading frames (ORFs) and six unique viral proteins, namely nucleoprotein, phosphoprotein, glycoprotein, X, matrix protein, and large protein, presents a complex structure of 8,903-nucleotide base pairs long with complementary 3’ and 5’ untranslated regions at their termini in the BDV genome sequence. These ORFs encode viral proteins crucial for the transcription, assembly, and replication of viruses. The 5’ and 3’ untranslated regions (UTRs) of the BDV genome play a vital role in these activities [15, 18]. Given the limitations of standard antiviral treatments for BDV, a neurotropic virus with severe neurological implications, medicinal plants emerge as a promising source of potential antiviral drugs. Among these plants, phytochemicals stand out as bioactive molecules with demonstrated antiviral properties against a range of viruses. Studies have shown that phytochemicals can target distinct stages of the viral life cycle. Some substances can disrupt the transcription of viral RNA or the expression of viral proteins, while others can inhibit viral enzymes necessary for replication. Understanding these specific mechanisms of action is crucial, as it paves the way for the development of targeted antiviral strategies against BDV [19].

Medicinal plants have long been a cornerstone of healthcare, with 80% of the world’s population still relying on them for primary healthcare due to their low cost and easy availability. The resurgence of interest in herbal medicine is driven by the limitations of modern medicine, such as the high cost and side effects of existing drugs and the growing demand for natural alternatives [20]. Medicinal plants contain bioactive compounds called phytochemicals, which possess various properties, including antiviral, antioxidant, anti-inflammatory, and anticancer properties. These compounds can target different stages of the viral lifecycle, such as viral attachment, viral replication, and cellular signalling, to prevent infections and viral replication. This research holds promise for developing new drugs to combat various diseases, such as BDV. Successful drugs derived from medicinal plants include Artemisinin (Antimalarial), Galantamine (Anti-Alzheimer’s), Taxol (Anticancer), and Vinca alkaloids (Anticancer) [21].

A diverse array of 1940 phytochemical compounds was extracted from a total number of 8617 compounds from 36 medicinal plants after eliminating similar found across multiple species and plant parts, they are *Ocimum americanum*, *Ocimum africanum*, *Ocimum carnosum*, *Artocarpus altilis*, *Phyllanthus niruri*, *Azadirachta indica*, *Ficus religiosa*, *Artocarpus integer*,*Artocarpus heterophyllus*, *Cynodon dactylon*, *Piper longum*, *Piper nigrum*, *Rosa centifolia*, *Citrus limon*, *Citrus sinensis*, *Curcuma longa*, *Panax ginseng*, *Aegle marmelos*, *Achyranthes aspera*, *Aloe vera*, *Bombax ceiba*, *Eclipta prostrata*, *Albizia procera*, *Flacourtia indica*, *Swertia angustifolia*, *Ocimium basilicum*, *Alstonia scholaris*, *Calotropis gigantea*, *Allium sativum*, *Cyperus rotundus*, *Ocimum campechianum*, *Ocimum tenuiflorum*, *Anogeissus acuminate*, *Mangifera indica*, *Butea monosperma*, *Ocimum gratissimum*. These plants, which are native to the South Asian subcontinent and were obtained from the IMPPAT (https://cb.imsc.res.in/imppat/) database, were utilized for *in-silico* drug discovery [22, 23]. In recent years, the use of phytochemicals as primary compounds in the search for novel drugs has gained significant attention [22]. These naturally occurring compounds provide valuable models for the development of innovative drugs and chemical entities [22]. Compounds like tannin, coumarin, steroid, thiosulfonate, lignin, polysaccharide, proanthocyanidin, quinone, terpene, saponin, flavonoid, alkaloid, and polyphenol have all demonstrated promising antiviral properties [25]. Medicinal plants have demonstrated their strong ability to inhibit the replication and spread of various viral strains. By specifically targeting key mechanisms within the viral life cycle, these compounds have shown potential in preventing viral attachment, entry, and replication. Additionally, their diverse chemical structures contribute to a broad spectrum of antiviral activities [24].

Despite ongoing research, there are currently no commercially available vaccines or effective antiviral treatments for BDV infection in humans. In this study, the control ligand of choice is Favipiravir, a modified pyrazine analogue, which is an antiviral drug that is used to treat influenza, viral infections [25–27]. Favipiravir has been studied for treating life-threatening diseases like Lassa virus, Ebola virus, and COVID-19 [25, 28, 29]. Unlike other influenza antiviral drugs, Favipiravir prevents virus entry and exit from cells [25]. It is a major target, the RNA-dependent RNA Polymerase (RdRP) catalytic domain, is likely to work similarly against other RNA viruses [25].

The purpose of this research is to evaluate and describe the binding affinities and molecular interactions of phytochemical compounds with the BDV viral protein through the use of computational methods, bioinformatics software, and statistical approaches. In our analysis, we utilized the Nucleoprotein (PDB ID: 1N93) of the BDV to conduct docking and structure-based virtual screening experiments to find novel inhibiting agents from a repository of phytochemical compounds with the aim of identifying the most effective pharmaceutical option.

## 2. Materials and methods

### 2.1 Identification, retrieval, and processing of the target protein

RCSB PDB (https://www.rcsb.org/) was used to retrieve the 3D protein crystal structure of BDV Nucleoprotein (PDB ID: 1N93). The nucleoprotein of the BDV PDB identity of 1N93 consists of one chain (chain A), 375 amino acids sequence length with a resolution of 1.76 **Å** value-free score of 0.187 [30]. The ligands, hetero atom, and water molecules of nucleoprotein were removed using the Protein Preparation Wizard, found in Schrödinger 2020–3. The protein’s bond ordering was determined, and the default protein preparation parameter, which utilized the OPLS3e force field—was used to add hydrogens and side chains that were absent [31].

### 2.2 Retrieval and optimization of compounds

The antiviral drug Favipiravir has been used as a control ligand for the target nucleoprotein. The 3D conformer of Favipiravir (CID 492405) was obtained in SDF format from PubChem (https://pubchem.ncbi.nlm.nih.gov/) and used as control. In quest of potential inhibitors, 8617 phytochemicals from 36 different plants that have antiviral activity and are available in the Indian subcontinent were used in this study (Supplementary Table 1 in S1 File). These phytochemical compounds were collected from the IMPPAT server (https://cb.imsc.res.in/imppat/) in SDF format and subsequently processed using the LigPrep function of maestro v11.4. Using the OPLS3e force field, the protein and ligands were finally optimized [31].

### 2.3 Computational analysis of protein-ligand binding affinity by molecular docking

Docking was performed between target macromolecules and unique 1940 phytochemicals using the Schrödinger Desmond program (Glide v8.8 and Maestro v12.5.139 modules) with the OPLS3e force field in standard precision mode. The native inhibitor, in combination with the target protein, was studied, and the binding location was selected for receptor grid generation. Using information about the residues in the receptor’s binding sites, a box with the coordinates (X = -33.315, Y = -13.57, and Z = 20.293) was used. Ligands binding with target protein energy were obtained, and different types of chemical bonds, as well as residues that interact with ligands, were visualized in the Maestro viewer.

### 2.4 Calculation of post-docking MM-GBSA

The free binding energies of the protein and ligand complexes were examined utilizing the MM-GBSA approach [32]. The analysis and visualization of the chosen compounds exhibiting the lowest energy binding were done using Glide v-8.8 and Maestro v-12.5.139. The protein binding site residues were identified by the co-crystallized ligand active site, which then generates a grid box that matches the binding site position. The molecular docking grid box has dimensions of X = 33.919 **Å**, Y = 36.781 **Å**, and Z = 1.174 **Å**. The MM-GBSA results were predicted using Prime MMGBSA v-3.059. To compare these results with recently screened drugs, both the MM-GBSA score and the docking score were used as controls. Using Maestro v-12.5.13948, available within Schrödinger, the residues, binding interactions, and binding free energy involved in the interaction plane were analyzed.

### 2.5 Prediction of pharmacokinetics (Pk) and toxicological profiles

Pharmacokinetic properties specify and confirm the effectiveness and quality of drugs in the beginning stages of Computer Aided Drug Design (CADD) [33]. The study examined the Pk properties of the best 80 among 1940 compounds with higher docking scores than the control ligand utilizing the SwissADME (http://www.swissadme.ch/) server, which is a free online tool that can predict several properties of small molecules, such as their physiochemical properties, lipophilicity, water solubility, and therapeutic potential [34].

To evaluate toxicity qualities such as Carcinogenicity, Hepatotoxicity, Mutagenicity, Immunotoxicity, and Cytotoxicity, the accessible internet server ProTox-II (https://tox-new.charite.de/protox_II/) was employed [35]. We examined compounds whose docking scores were higher than the control ligand in terms of toxicity prediction.

### 2.6 Molecular dynamics simulation

The structural stability of the protein-ligand complex in a given physiological condition was examined using molecular dynamics (MD) simulation. The chosen compound’s capacity to bind the required protein was investigated using 100 ns MD simulations to determine protein-ligand complex stability. A 100 ns MD simulation was carried out using the Desmond package provided by the Schrödinger suit. After the complex structure was created through molecular docking studies of protein-ligand complexes, it was pre-processed utilizing protein preparation wizard. To maintain the system’s volume, an orthorhombic periodic boundary box shape with an interval (10 × 10 × 10 **Å**^**3**^) was used for each complicated simple point-charge (SPC) water model which was used to analyze the system. Na^+^ and Cl^-^ ions were chosen and added at random to the solvated system to maintain the salt concentration at 0.15 M. The system was mitigated and relaxed by applying the OPLS3e force field. Finally, at 1.01325 bar pressure and 300.0 K temperature, the constant pressure-constant temperature (NPT) ensemble was run. After relaxing the system for each complex, the final production cycle was carried out with 100 ps recording intervals and an energy of 1.2. Furthermore, data for RMSD, rGyr, RMSF, ligand RMSD, protein-ligand contact, and SASA were computed to assess the stability and dynamic characteristics of these complexes. DCCM and PCA were calculated using the Bio3D package of R programming from the MD trajectories.

## 3. Result

### 3.1 Retrieval of plant-based bioactive compounds

The IMPPAT database includes phytochemicals, Indian medicinal plants, and therapeutic uses, which improves natural product-based drug discovery with an integrated cheminformatic platform. IMPPAT is also expected to enable systems-level techniques to elucidate molecular linkages between Indian medicinal plant compounds and therapeutic effects. This research aimed to analyze the efficacy of the drug provided by the website in countering the BDV. The study aimed to examine the efficacy of a drug from a website against BDV using 1940 unique phytochemical compounds from the IMPPAT server, which contains 8617 compounds from 36 medicinal plants and likely found overlaps between these compounds.

We initiated our study by sourcing potential bioactive compounds from medicinal plant materials. The compounds retrieved from plant sources are the starting point for docking studies.

### 3.2 Interpreting protein-ligand interactions and affinity analysis

**Fig 1:**
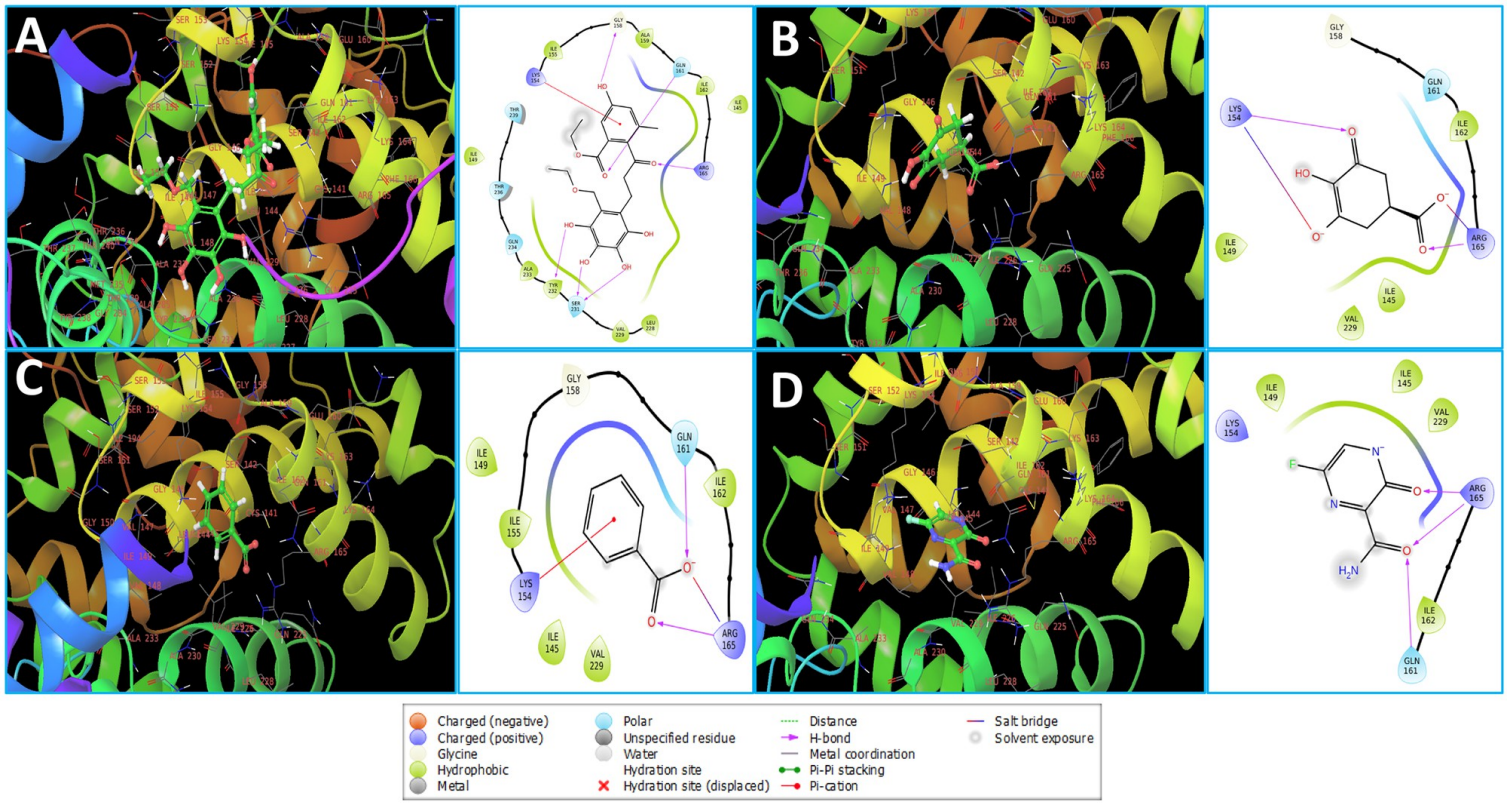
Interaction between BDV Nucleoprotein and three selected compounds, and control representing in 3D (left) and 2D (right) format and demonstrating the compounds, A, B, C, D (CID 163114683, CID 20871246, CID 243, control CID 492405), respectively.

Molecular docking was performed on 1940 unique phytochemical compounds, resulting in the selection of the three best compounds (CID 163114683, CID 20871246, and CID 243) based on their high docking scores and favorable interactions. The docking scores of these compounds were -6.244, -6.116, and -6.07 kcal/mol, respectively, eclipsing the control ligand’s (CID 492405) score of -5.373 (Table 1). Molecular interactions analysis identified hydrophobic, polar, electrostatic, and hydrogen bonds between the protein’s active site and the ligands displayed by Maestro viewer. The control ligand and the top three compounds interacted with frequent amino acid residues (GLN 161, ARG 165, ILE 145, ILE 162, ILE 149, VAL 229), indicating a similar binding mode. All of the three compounds reacted with a distinct amino acid, as illustrated in Fig 1 and Table 2.

**Table 1:** List of compound identity through PubChem CID, 3D structures, compound names, and docking score of the three selected ligands, and Favipiravir (control) with their biological use.

PubChem CID	Compound Name	Docking Score (kcal)	Plant Source	Biological Use
163114683	Nimbochalcin	-6.244	*Azadirachta indica* [36]	Anti-malarial [37], Anti-bacterial [38], Anti-cancer [39]
20871246	3,4-Dihydroxy-5-oxocyclohex-3-ene-1-carboxylic acid	-6.116	*Mangifera indica* [40]	Antioxidant [41] Parkinson’s disease and Schizophrenia [42]
243	Benzoic acid	-6.07	*Mangifera indica* [40]	Anti-microbial [43]
492405 (Control)	Favipiravir	-5.373		Anti-influenza [25] Anti-viral [44]

**Table 2:** Amino acid residues of three lead compounds and a control ligand that are involved in polar bonds, hydrogen bonds, electrostatic bonds, and hydrophobic bonds.

PubChem CID	Compound Name	H-Bond	Polar Bond	Hydrophobic Bond
163114683	Nimbochalcin	GLY 158, GLN 161, ARG 165, TYR 232, SER 231,	THR 239, THR 236, GLN 234, SER 231, GLN 161	ILE 149, ILE 155, ALA 159, ILE 162, ILE 145, LEU 228, VAL 229, TYR 232, ALA 233
20871246	3,4-Dihydroxy-5-oxocyclohex-3-ene-1-carboxylic acid	LYS 154, ARG 165	GLN 161	ILE 149, ILE 162, ILE 145, VAL 229
243	Benzoic acid	GLN 161, ARG 165	GLN 161	VAL 229, ILE 149, ILE 155, ILE 145, ILE 162
492405 (Control)	Favipiravir	ARG 165, GLN 161	GLN 161	ILE 149, ILE 145, VAL 229, ILE 162

### 3.3 Analysis of post-docking MM-GBSA

MM-GBSA calculations were conducted to estimate the binding free energies of the top three compounds (CIDs 163114683, 20871246, and 243) and the control ligand (CID 492405) to the 1N93 protein. The results indicated that all four compounds exhibit favorable binding free energies (-51.21, -13.94, -22.95, and -18.57 kcal/mol, respectively), (Table 3) suggesting their potential to bind to and inhibit the target protein.

**Table 3:** The BDV nucleoprotein’s various energy components are displayed in the table together with its net MM-GBSA binding free energy (kcal/mol) while the compounds are in complex.

CID	dG Bind	dG Bind Coulomb	dG Bind Covalent	dG Bind Hbond	dG Bind Lipo	dG Bind Packing	dG Bind Solv GB	dG Bind vdW
**20871246**	-13.94	-56.79	2.65	-3.26	-4.1	0	64.6	-17.04
**163114683**	-51.21	-54.67	5.8	-4.47	-10.63	-0.7	40.98	-27.53
**243**	-22.95	-28.45	0.48	-2.45	-7.68	0	27.93	-12.77
**492405 (Control)**	-18.57	-106.54	-0.21	-2.09	-4.38	-0.28	110.67	-15.74

After identifying promising ligands through docking and calculating their binding free energies using MM-GBSA, we proceeded to evaluate their pharmacokinetic properties.

### 3.4 Evaluation of Pk properties and potential toxic effects

According to Lipinski’s rule of five, drug-likeness, synthesis accessibility, water solubility, gastrointestinal absorption, lipophilicity, and physicochemical characteristics, the selected compounds (CIDs 163114683, 20871246, 243) and the control (CID 492405) were assessed (Table 4). Every molecule passed Lipinski’s five requirements, suggesting that they all had promising drug-like qualities. Positive pharmacokinetic profiles are suggested by these characteristics, which may lower the chance of clinical trial failure.

**Table 4:** Physicochemical characteristics, water solubility, drug-likeliness, lipophilicity, GI absorption, and accessibility of specific synthesis three lead compounds (CIDs 163114683, 20871246, 243) with control ligand (492405).

Properties		CID 163114683	CID 20871246	CID 243	CID 492405 (Control)
**Physico-chemical properties**	MW (g/mol)	434.44(g/mol)	172.14(g/mol)	122.12(g/mol)	157.1(g/mol)
	Heavy atoms	31	12	9	11
	Arom. heavy atoms	12	0	6	6
	Rotatable bonds	10	1	1	1
	H-Bond acceptors	9	5	2	4
	H-Bond donors	5	3	1	2
**Lipophilicity**	Consensus Log Po/w	2.23	-0.43	1.44	-0.27
**Water Solubility**	Log S (ESOL)	-3.62	-0.39	-2.2	-0.8
**Pharmacokinetics**	GI absorption	Low	High	High	High
	BBB permeant	No	No	Yes	No
**Drug likeness**	Lipinski	Yes, 0 violation	Yes, 0 violation	Yes, 0 violation	Yes, 0 violation
**Medi. Chemistry**	Synth. accessibility	3.48	3.35	1	2.08

Toxicological prediction also evaluated other endpoints (cytotoxicity, mutagenicity, carcinogenicity, and immunotoxicity) as well as possible organ toxicity. Table 5 provides an overview of the findings for each drug (CIDs 163114683, 20871246, 243, and 492405). These results imply that the chemicals have positive toxicity profiles, suggesting a low potential for side effects and safety for human use.

**Table 5:** Predicted toxicity profiles for three selected compounds (CID 163114683, CID 20871246, and CID 243) compared to a control ligand (CID 492405).

Classification	Target		CID: 163114683	CID: 20871246	CID: 243	CID: 492405 (control)
**Organ toxicity**	Hepatotoxicity	Prediction	Inactive	Inactive	Active	Inactive
		Probability	0.86	0.76	0.54	0.66
**Toxicity and points**	Carcinogenicity	Prediction	Inactive	Inactive	Inactive	Active
		Probability	0.63	0.68	0.74	0.53
	Immunotoxicity	Prediction	Inactive	Inactive	Inactive	Inactive
		Probability	0.82	0.99	0.99	0.99
	Mutagenicity	Prediction	Inactive	Inactive	Inactive	Inactive
		Probability	0.77	0.89	0.99	0.76
	Cytotoxicity	Prediction	Inactive	Inactive	Inactive	Inactive
		Probability	0.82	0.82	0.86	0.84

Following docking and subsequent MMGBSA calculations to assess binding free energies, molecular dynamics simulations were performed to assess the conformational stability in a simulated system.

### 3.5 Molecular dynamics simulation

#### 3.5.1 RMSD analysis

**Fig 2:**
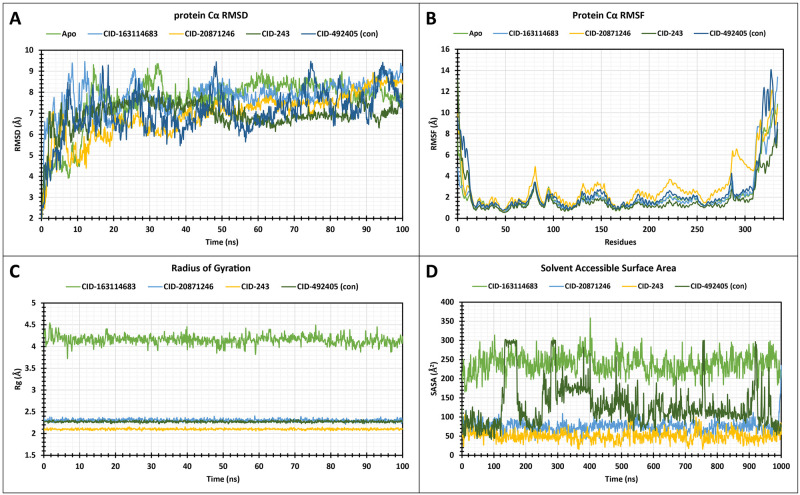
The apoprotein’s RMSD, RMSF, Rg, and SASA values are displayed complexed with the three lead compounds and control chosen and extracted from the complex system’s Cα atoms.

A 100 ns MD simulation was performed to assess the stability of the protein-ligand complexes. The average RMSD values for CID 20871246 and CID 243 were 6.89 and 6.93 **Å**, respectively, indicating better stability compared to the control (CID 492405). These compounds exhibited a slight fluctuation during the initial 20 ns but maintained a consistently lower RMSD throughout the simulation (Fig 2A). CID 163114683 also showed excellent stability, with an average RMSD of 7.76 **Å**. The control ligand had an average RMSD of 7.08 **Å**. Overall, CID 20871246 and CID 243 demonstrated the most stable protein-ligand complexes, suggesting their potential for maintaining protein structure and function.

#### 3.5.2 RMSF analysis

The protein-ligand complexes’ local flexibility changes were revealed by RMSF analysis. The average RMSF values for CID 163114683, CID 20871246, CID 243, and CID 492405 were 2.12 **Å**, 2.98 **Å**, 1.81 **Å**, and 2.51 **Å**, respectively (Fig 2B). A peak region was observed at residues GLU 109, ALA 251, SER 323, and GLY 328. The RMSF analysis also indicated that the protein structure is relatively rigid, with minimal fluctuations in the core regions. The N- and C-terminal domains, α-helix, and β-sheet exhibited higher flexibility, suggesting that these regions may be more susceptible to conformational changes upon ligand binding.

#### 3.5.3 Evaluation of Radius of Gyration (rGyr)

The rGyr analysis was performed to assess the conformational changes of the protein-ligand complexes during the 100 ns MD simulation. The average rGyr values for CID 163114683, CID 20871246, CID 243, and CID 492405 (control) were 4.15 **Å**, 2.30 **Å**, 2.10 **Å**, and 2.27 **Å**, respectively (Fig 2C). The results indicate that CID 20871246, CID 243, and CID 492405 (control) maintained a stable protein structure without significant conformational changes in the active site. In contrast, CID 163114683 induced conformational changes, as evidenced by its higher rGyr value and fluctuations throughout the simulation.

#### 3.5.4 Solvent Accessible Surface Area (SASA)

SASA analysis was performed to assess the solvent accessibility of the protein-ligand complexes. The average SASA values for CID 163114683, CID 20871246, CID 243, and CID 492405 (control) were 238.67 **Å**^**2**^, 75.25 **Å**^**2**^, 50.18 **Å**^**2**^, and 129.28 **Å**^**2**^, respectively (Fig 2D). These results indicate that CID 20871246 and CID 243 have lower SASA values, suggesting reduced solvent exposure and potentially stronger interactions with the protein. In contrast, CID 163114683 has a higher SASA value, indicating greater solvent accessibility and potentially weaker protein-ligand interactions.

#### 3.5.5 Protein-ligand contact analysis

**Fig 3:**
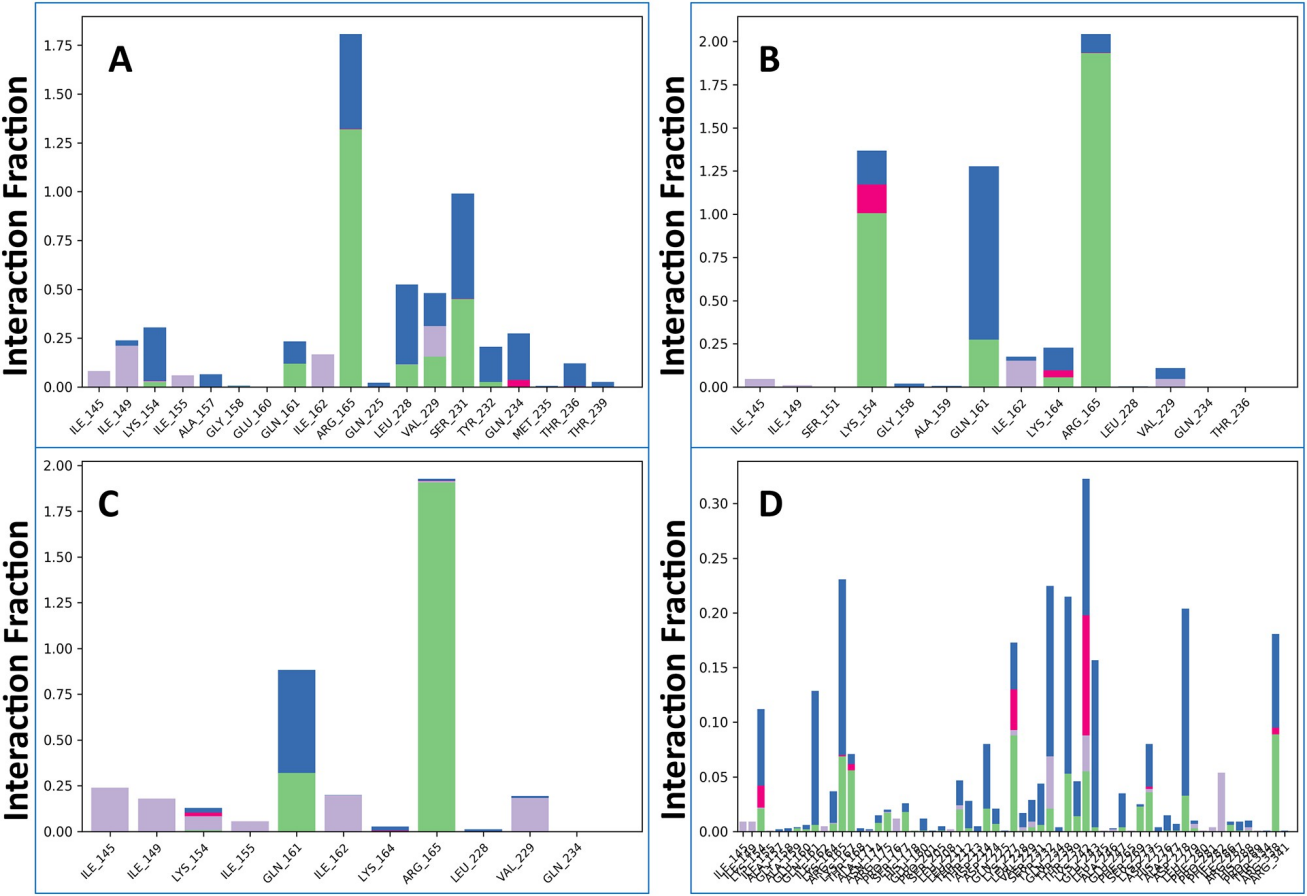
The protein-ligand interactions identified during the 100 ns simulation are displayed in the organized bar charts. Here, we see CID 163114683, CID 20871246, CID 243, and CID 492405 (control) in A, B, C, and D interacting with the apoprotein.

To understand the nature of protein-ligand interactions, contact analysis was performed using the simulated interactions diagram (SID). The results revealed that all compounds (CID 163114683, CID 20871246, CID 243, and CID 492405) formed multiple interactions with the target protein, including ionic, water bridge, non-covalent (hydrophobic), and hydrogen bonds. These interactions persisted throughout the 100 ns simulation, contributing to the stability of the protein-ligand complexes. The compound CID 163114683 formed multiple contacts with residues LYS 154, ARG 165, LEU 228, VAL 229, and SER 231, with interaction fractions (IF) ranging from 0.26 to 1.76 (Fig 3A). CID 20871246 formed multiple contacts with LYS 154, LYS 164, and ARG 164 (IFs: 1.37, 0.25, and 2.22, respectively) (Fig 3B). CID 243 formed contacts with LYS 154 and ARG 165 (IFs: 0.12 and 1.9) (Fig 3C). The control compound, CID 492405, formed contacts with multiple residues, including LYS 154, LYS 164, ARG 165, LYS 167, ARG 175, LEU 211, LYS 227, LEU 228, VAL 229, TYR 232, LYS 242, LYS 273, LEU 279, HIS 288, and ARG 338 (Fig 3D). Among the compounds, CID 20871246 exhibited the strongest hydrogen and other bond interactions with the apoprotein, suggesting a potentially more stable and potent binding.

#### 3.5.6 Analysis of ligand-protein contact

**Fig 4:**
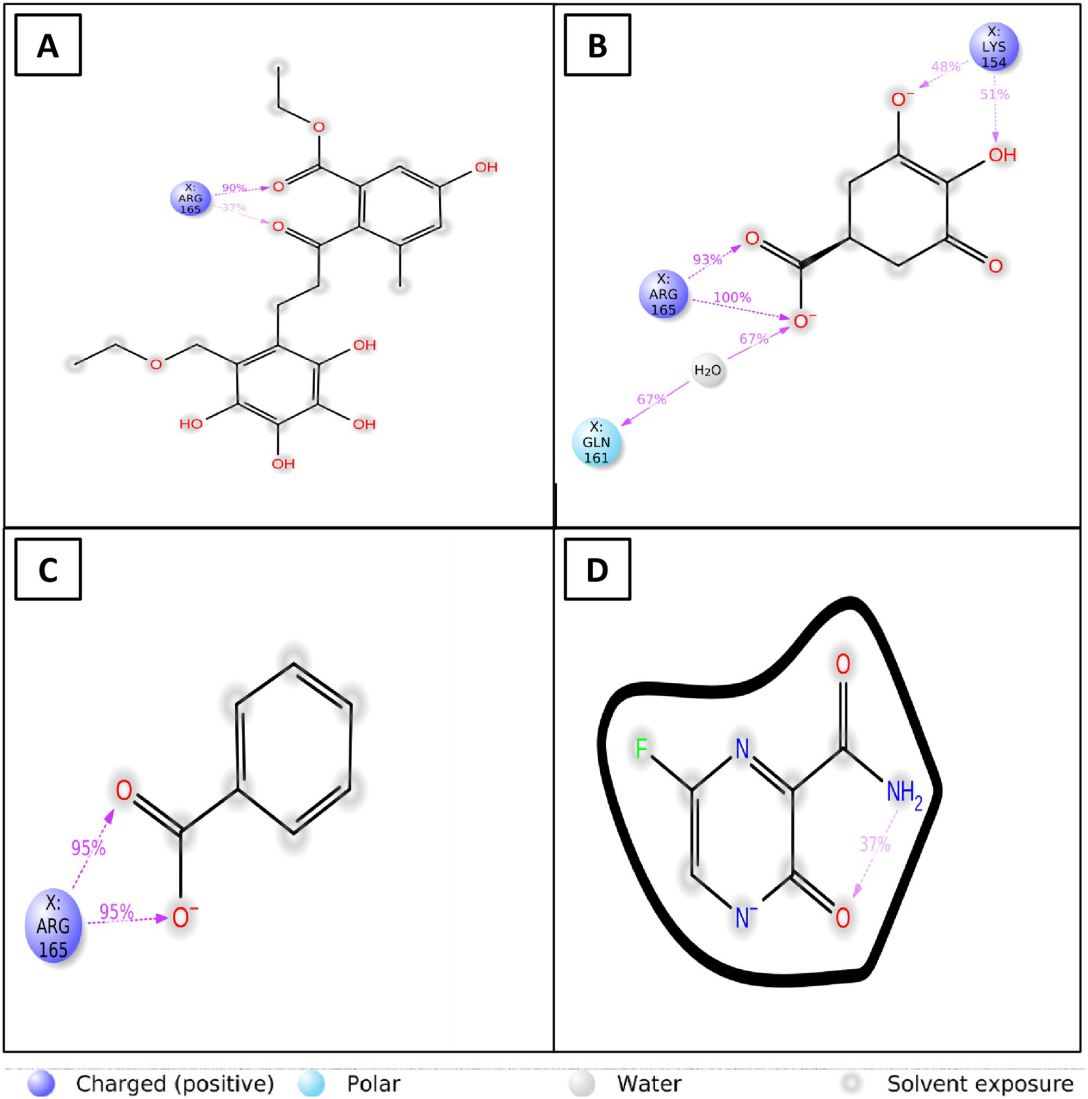
Graph showing the data regarding the interactions between proteins and ligands after a 100 ns simulation. Here, the CID 163114683, CID 20871246, CID 243 and CID 492405 (control) are recorded.

The three lead ligands, including CID 163114683, CID 20871246, CID 243, and CID 492405 (control), have been observed to have protein interactions across the entire simulated interactions diagram (SID). The compound CID 20871246 produced many (more than two) interaction residues during the simulation and multiple contacts with the same subtype of ligand preserve the unique interaction as a result (Fig 4) and exhibit greater stability in ligand-protein interaction study when compared with the control, CID 492405 (Fig 4).

#### 3.5.7 Ligand RMSD

**Fig 5:**
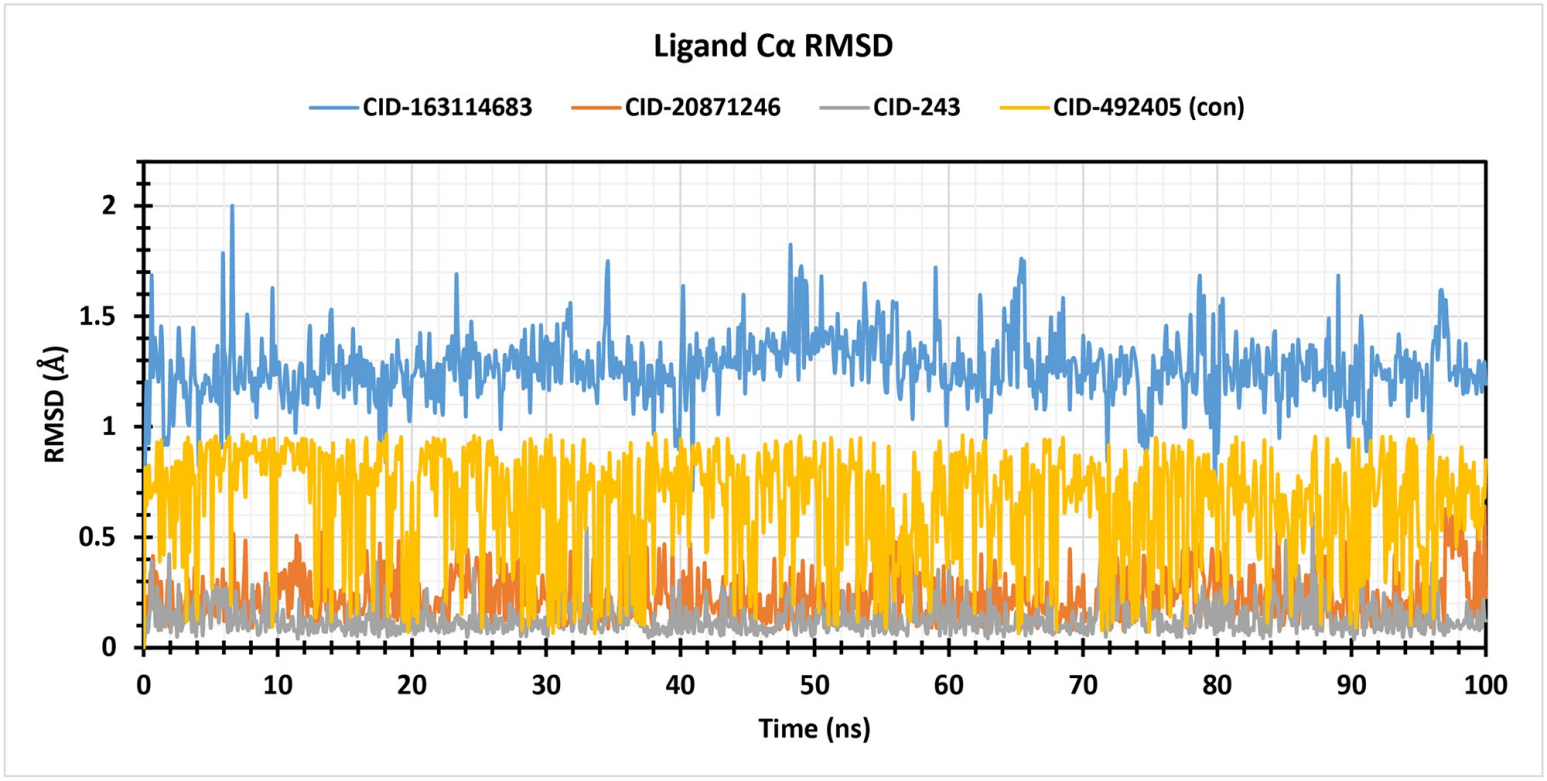
Illustrating the RMSD values obtained from the ligand atoms of the complex structure CID 163114683 (blue), CID 20871246 (orange), CID 243 (grey), and control CID 492405 (yellow) over a simulation duration of 100 ns.

Ligand RMSD measures the average distance between atom positions in a ligand’s current conformation and its reference conformation. A lower value indicates stability, while fluctuations indicate instabilities. It’s a useful technique for evaluating binding pocket stability. The average Ligand RMSD value for three selected compounds including the CID 163114683, CID 20871246, CID 243, and CID 492405 (control), were 1.26 **Å**, 0.23 **Å**, 0.13 **Å**, and 0.65 **Å** (control), respectively (Fig 5). In comparison to the control, the compound CID 163114683 displayed somewhat excessive fluctuation and continued to fluctuate continuously over the course of the 100 ns simulation period. However, CID 20871246 and CID 243 produced the lowest fluctuation with more stability in contrast to the other lead compound CID 163114683 and control CID 492405.

#### 3.5.8 PCA analysis

**Fig 6:**
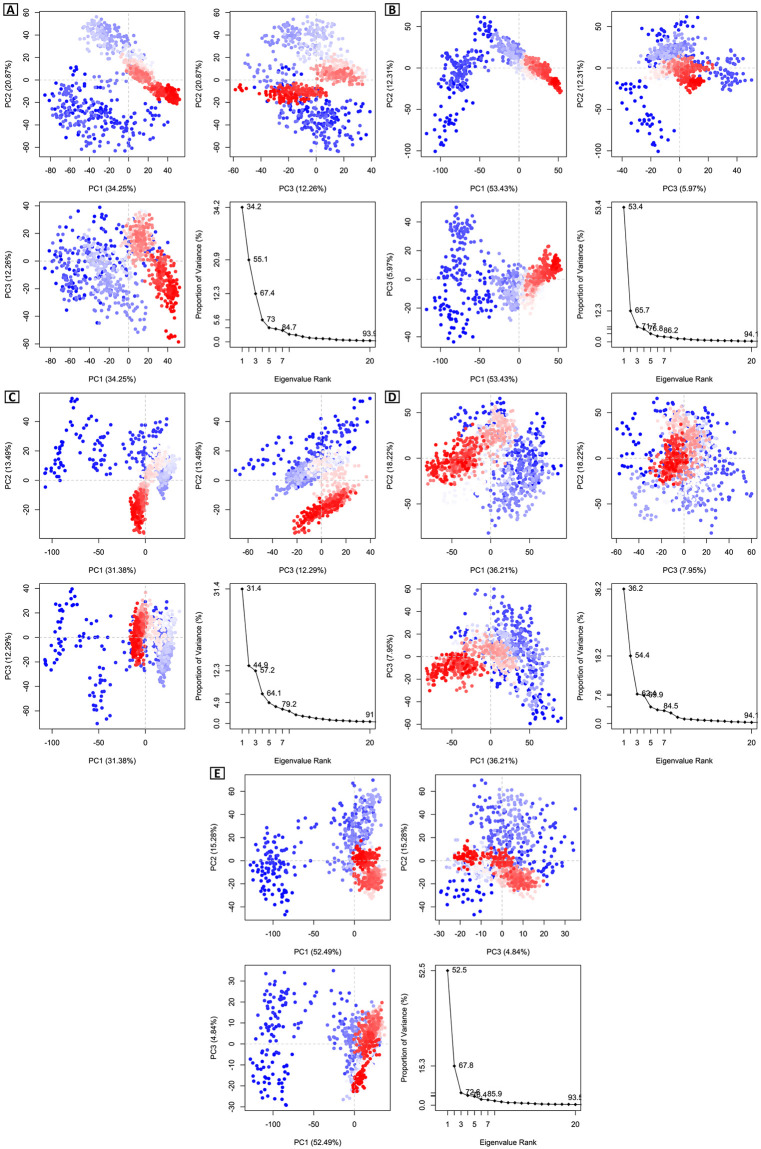
Eigenvalue in Principal Component Analysis versus proportion of variance. Three separate panels each display the different regions. There are three variations: PC1, PC2, and PC3. In this case, the CIDs are (A) 163114683, (B) 20871246, (C) 243, (D) 492405 (control), and (E) Apoprotein (1N93).

A method for reducing dimensionality, called Principal Component Analysis (PCA) can be used to study the dynamics and structures of proteins. A graph of the eigenvalues (protein) vs the eigenvector index (eigenmode) for the first 20 modes of motion (1N93-CID 20871246) is displayed (Fig 6B) and indicates more stability. The eigenvalues displayed the fluctuations in the hyperspace eigenvectors. The five eigenvectors with the highest eigenvalues (53.4%–86.2%) showed dominant motion in the systems under consideration. To chart every change, three principal components (PC1, PC2, and PC3) were employed. The results presented in Fig 6B indicate that PC1 clusters had the largest level of variability (53.43%), followed by PC2 clusters (12.31%), and PC3 clusters (5.97%). Due to its modest size and least changeable structure, PC3 was believed to be the most stable protein-ligand binding complex. Compared to the 1N93-CID 20871246 complex, the 1N93-CID 243 complex (Fig 6C) shows greater variability in PC1, PC2, and PC3, but less than the 1N93-CID 492405 (control) complex (Fig 6D). In comparison to the other three complexes, the 1N93-CID 163114683 complex (Fig 6A) exhibits greater variability. The PC1, PC2, and PC3 values of apoprotein with PDB ID 1N93 (Fig 6D) are 52.49, 15.28, and 4.84%, in that order. Due to its smaller and more compact structure than PC1 and PC2, PC3 was believed to be the most stable. The color blue indicates the highest degree of mobility, while the colors white and red indicate moderate and lowest degrees of mobility, respectively.

#### 3.5.9 DCCM analysis

**Fig 7:**
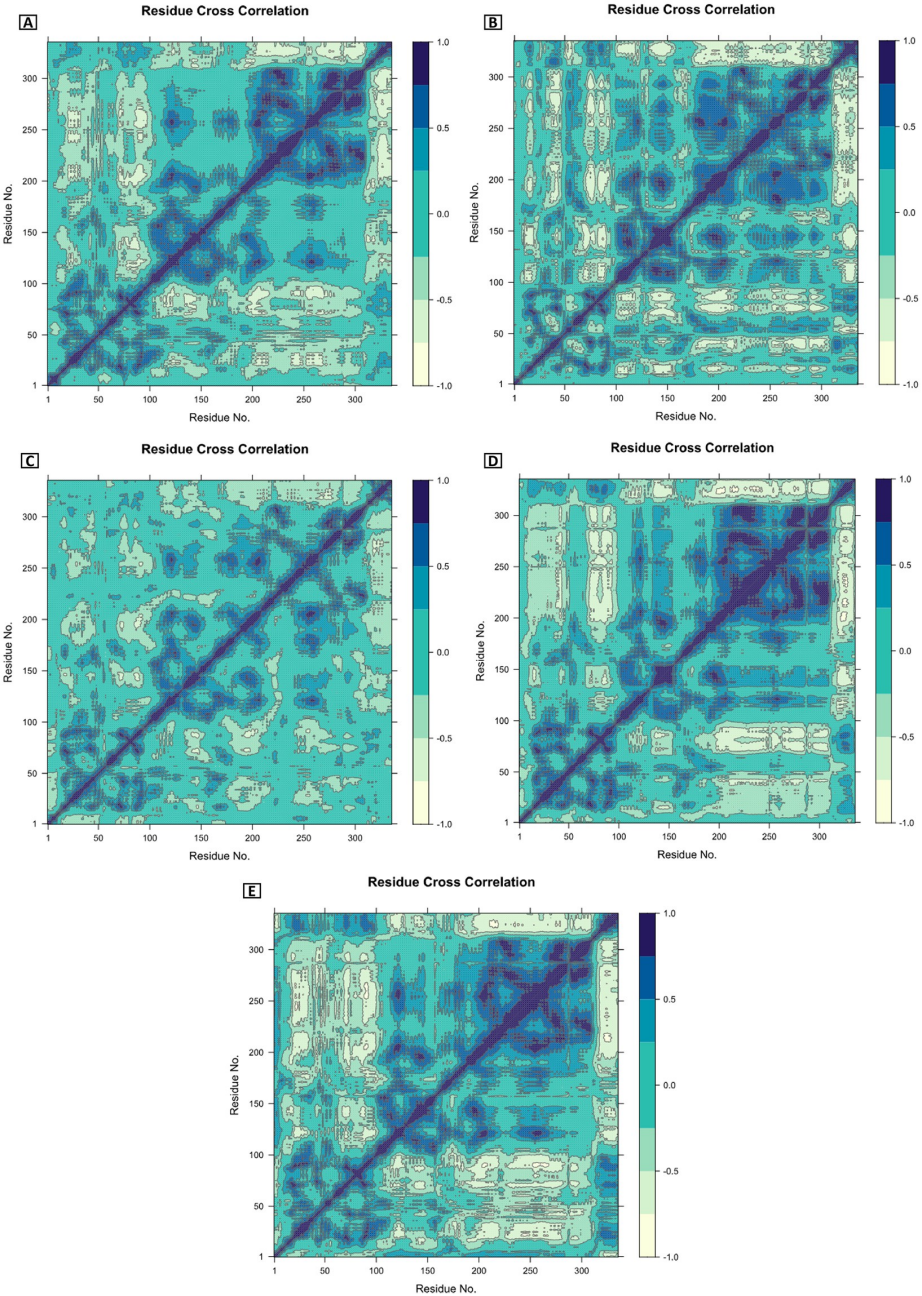
An intricate dynamic cross-correlation diagram represented by blue and sea green, respectively, as positive and negative. In this case, CIDs are (A) 163114683, (B) 20871246, (C) 243, (D) 492405 (control), and (E) Apoprotein (1N93).

There are several time scales at which protein movements occur ranging from femtoseconds to seconds [45, 46]. DCCM is also influenced by the time span during which the correlation data was gathered [47]. Using inter-residue DCCM analysis, the target protein (1N93) and its docked complexes with CIDs are (A) 163114683, (B) 20871246, (C) 243, (D) 492405 (control), and (E) Apoprotein (1N93) were examined for correlated and anti-correlated movements. The color blue indicates related residues, whereas the color sea green indicates anti-correlated residues. The distribution of target protein 1N93 among populations was shown by the residue index maps to have both positive and negative correlations. A significant association between the target protein (1N93) and the chosen ligand (CID 20871246) was discovered by comparing the pairwise cross-correlation coefficient value on the cross-correlation map (Fig 7B) to other complexes.

## 4. Discussion

In light of the significant public health concern posed by the BDV, particularly its potential association with various neurological and mental health disorders, the exploration of novel therapeutic avenues becomes increasingly imperative. BDV’s enigmatic nature, characterized by diagnostic challenges, persistence, widespread occurrence, and unconventional reservoirs, necessitates the investigation of alternative treatment strategies beyond conventional methods. Phytochemicals derived from Indian medicinal plants offer a promising approach in this regard, potentially leading to the discovery of effective therapeutic agents against BDV. This research direction holds immense potential for mitigating the detrimental effects of this elusive virus. Furthermore, the identification of a prospective drug molecule that targets BDV is immensely significant. To expedite this critical endeavor, we propose the design of a workable medicine *in- silico*, offering a rapid and cost-effective approach to drug discovery.

The conventional approach to drugs discovery and development process is a lengthy and expensive undertaking, often taking over a decade (an average of 10–15 years) and costing approximately US$800 million to US$1.8 billion [48]. However, advancements in CADD offer a promising alternative. CADD techniques, such as molecular docking and pharmacokinetic analysis, toxicity, post-docking MMGBSA, MD simulation, PCA, DCCM can significantly enhance the efficiency and effectiveness of drug discovery. This study adopts a cutting-edge *in-silico* approach CADD to fast-track the discovery of BDV inhibitors, bypassing the limitations of conventional, resource-intensive drug discovery methods.

One thousand nine hundred forty (1940) unique phytochemicals from 36 Indian medicinal plants against the BDV nucleoprotein (1N93) were screened using molecular docking. The compounds were further analyzed to identify those with the strongest binding affinities. The top three candidates that emerged from this study were CID 163114683 (IMPHY000668), CID 20871246 (IMPHY007896), and CID 243 (IMPHY002962). These compounds demonstrated exceptional binding affinities, with scores of -6.244, -6.116, and -6.07 kcal/mol, respectively. These scores significantly exceeded that of the control ligand (CID 492405), which had a docking score of -5.373 kcal/mol, emphasizing the superior binding potential of the identified candidates. To validate the docking results, post-docking MM-GBSA analysis was employed to evaluate the binding free energies of the selected compounds (CID 163114683, CID 20871246, and CID 243) and the control (CID 492405) in their interactions with the 1N93 protein. The complex analysis disclosed negative binding free energies of -51.21, -13.94, -22.95, and -18.57 kcal/mol, respectively. These significantly higher negative binding free energies for CID 163114683, and CID 243 compared to the control (CID 492405) indicate their enhanced binding affinities and suggest their potential to maintain long-lasting interactions with the chosen protein [49].

Drug discovery is a meticulous process requiring rigorous evaluation of a compound’s safety, efficacy, and bioavailability. ADME screening plays a critical role in this process, as these properties significantly influence drug success. High molecular weight and lipophilicity can hinder absorption, while good solubility and an optimal number of rotatable bonds favor bioavailability [34, 50, 51]. Data from this study analysis identified three lead compounds for further investigation and interestingly all compounds, including the control, adhered to Lipinski’s Rule of Five (RO5). This rule of thumb predicts good oral bioavailability, suggesting these compounds have favorable pharmacokinetic profiles. According to the Lipinski rule, a compound is more likely to have low permeability or absorption if it includes more than five hydrogen-bond donors, a molecular weight greater than 500, a computed log P value greater than 5, and more than ten nitrogen and oxygen atoms combined in the molecule [52]. A chemical’s high molecular weight may lessen pharmaceutical candidates’ capacity to pass past the biological barrier. The level of lipid solubility is measured using the lipophilicity indicator. The logarithm of the target molecule’s partition coefficient between the inorganic and aqueous phases is represented by logP, and the log P value affects how quickly the drug molecule enters the body. An inversely proportional relationship exists between the log P value and the body’s absorption of drug molecules. The Logs value, which indicates the likely solubility of the substance, is often chosen to be as low as feasible. Our research shows that the compounds CID 20871246, CID 243, CID 492405(Control), and CID 163114683 have molecular weights less than 500 and hydrogen bond donors less than or equal to 5. A Lipinski rule-defined good absorption, or permeability is indicated by a log P value of less than 5 and a sum of oxygen and hydrogen atoms in the molecule of less than 10.

The toxicity profiles of our lead compounds and the control CIDs 163114683, 20871246, 243, and the control 492405 were assessed. The compounds were assessed for organ toxicity, carcinogenicity, immunotoxicity, mutagenicity, and cytotoxicity. All four compounds demonstrated a favorable safety profile with predominantly inactive organ toxicities with the highest probability (0.76 to 0.99). However, CID 243 exhibited low-probability active hepatotoxicity (0.54), while the control compound CID 492405 showed low-probability active carcinogenicity (0.53). The remaining compounds, CID 163114683 and CID 20871246 exhibited inactive toxicity profiles across all categories, suggesting a promising safety margin. While these compounds appear safe for human use based on their toxicity profiles, their ability to cross the blood-brain barrier is crucial for treating BDV-related neurological diseases. ADMET analysis revealed that only CID 243 could effectively cross the blood-brain barrier. To address this limitation for the other compounds, the potential of carrier molecules to facilitate their transport across the blood-brain barrier and enable them to target BDV infections in the brain is a hopeful prospect.

MD simulations, a cornerstone of *in-silico* approaches, aim to predict the atomic movements of biomolecules like proteins. These simulations leverage a defined physical model to faithfully represent protein folding, conformational changes, and ligand binding events. Notably, MD simulations offer unparalleled temporal resolution in the femtosecond range, providing a wealth of information. Additionally, these simulations hold promise in predicting bio-molecular responses to external perturbations at the atomic level [53, 54]. MD simulations investigated the binding affinities of the three lead compounds (CID 20871246, CID 243, and CID 163114683) to an apoprotein. The RMSD of a protein-ligand complex system can be used to determine the average dislocation generated by a chosen atom over a specific period relative to a reference time. The stability and dependability of system equilibration are assessed using RMSD analysis [55]. The analysis revealed that CID 20871246 and CID 243 displayed the lowest average RMSD values (around 6.89 **Å** and 6.93 **Å**, respectively) throughout the 100 ns simulation, indicating the most robust and most stable binding compared to the control (CID 492405). Although CID 163114683 exhibited slightly higher RMSD values, it still maintained stable binding. The RMSF can be used to determine and describe local alterations in the protein chain that occur when drugs interact with particular residues. Here, RMSF values were calculated to assess protein flexibility upon ligand binding. The control (CID 492405) exhibited an average RMSF of 2.51 **Å**, while the selected compounds displayed varying degrees of flexibility: CID 163114683 (2.12 **Å**), CID 243 (1.81 **Å**), and CID 20871246 (2.98 **Å**). These results suggest that CID 243 and CID 163114683 induce minimal protein conformational changes upon binding, while CID 20871246 might cause slightly higher alterations. The rGyr is the unit of measurement for the atoms’ diffraction around the axis of a protein-ligand complex system. One of the most important markers for identifying the structural activity of a macromolecule is the rGyr calculation, which reflects differences in complex compactness [56]. The rGyr values for the compounds were 4.15 **Å** (CID 163114683), 2.30 **Å** (CID 20871246), 2.10 **Å** (CID 243), and 2.27 **Å** (CID 492405, control). These values suggest that CID 20871246, CID 243, and the control (CID 492405) induced minimal conformational changes in the protein’s active site. In contrast, CID: 163114683, with higher rGyr value (4.15 **Å**), likely caused some conformational shifts in the active site. SASA measures biomolecule surface area, influencing protein-ligand binding, folding, and interactions. It’s crucial in protein folding and stability studies, as protein surface amino acid residues serve as active sites, making understanding solvent-like behaviour easier. A higher SASA value generally suggests a weaker structure, whereas a lower value indicates a more tightly packed complex between protein residues and water molecules [57]. The compounds exhibited SASA values of 238.67 **Å**^**2**^ (CID 163114683), 75.25 **Å**^**2**^ (CID 20871246), 50.18 **Å**^**2**^ (CID 243), and 129.28 **Å**^**2**^ (CID 492405, control), suggesting strong binding for CID 20871246 and CID 243. Protein-ligand contact analysis simplifies identifying and examining intermolecular interactions between proteins and ligands, providing structural foundations and binding affinity. Simulation interaction diagram (SID) assesses the protein-ligand complex structure and interactions over 100 ns. Lastly, the MD simulation suggests that molecules with CIDs 20871246 and 243 form strong and stable bonds with the apoprotein, while molecule CID 163114683 causes the protein to change its shape slightly and instability of the protein-ligand complex.

A method for reducing dimensionality, called Principal Component Analysis (PCA) can be used to study the dynamics and structures of proteins. We examined every trajectory that the MD simulation generated as output files. PCA analysis revealed complexes CID 163114683-1N93 and CID 243-1N93 showed the highest PC3 values, 12.26% and 12.29%, respectively, indicating lower stability than other complexes. However, CID 20871246-1N93 emerged as the most stable complex (PC3 value of 5.97%) compared to the control (CID 492405-1N93, PC3 value of 7.95%) and other complexes. Additionally, the Apo protein displayed a high level of stability with a PC3 value of 4.84%. There are several time scales at which protein movements occur ranging from femtoseconds to seconds. DCCM is also influenced by the time span during which the correlation data was gathered. The target protein and compound CID 20871246 were shown to have a significant correlation, as evidenced by the greater pairwise cross-correlation coefficient value on the cross-correlation map when compared to other complexes. In this study, three compounds have been evaluated using multiple computational analyses: CID 163114683 (Nimbochalcin), CID 20871246 (3,4-Dihydroxy-5-oxocyclohex-3-ene-1-carboxylic acid), and CID 243 (Benzoic acid) in comparison to the control drug Favipiravir. Each of these compounds has distinct structural and functional properties. The most promising compound to treat BDV infection is 3,4-dihydroxy-5-oxocyclohex-3-ene-1-carboxylic acid, which can inhibit BDV nucleoprotein and block BDV replication at the initial phase. This phytochemical compound, 3,4-dihydroxy-5-oxocyclohex-3-ene-1-carboxylic acid, is found in the flower of the *Mangifera indica* [40] and exhibits antioxidant activity [41] with potential functionality against parkinson’s disease and schizophrenia [42]. *In vitro* and *in vivo* investigations are crucial for validating the efficacy of candidate drugs against target proteins. Favipiravir, Ribavirin, and Interferon-alpha were the antiviral medications that were compared to the identified compounds, CID 163114683, CID 20871246, and CID 243. The main points of emphasis were safety, *in vitro* and *in vivo* efficacy, target proteins, mechanism of action, and binding affinity. The objective was to comprehend these substances’ special qualities and potential as BDV inhibitors. Researchers can assess these drugs’ clinical potential and provide guidance for future medication development by contrasting them with established antiviral medicines.

The lead compound can be used as Testosterone 17beta-dehydrogenase (NADP+) inhibitor, Sugar-phosphatase inhibitor, NADPH-cytochrome-c2 reductase inhibitor, Fatty-acyl-CoA synthase inhibitor, Methylenetetrahydrofolate reductase (NADPH) inhibitor, Pullulanase inhibitor, Glucose oxidase inhibitor, Aspartate-phenylpyruvate transaminase inhibitor, Sulfite oxidase inhibitor, Acetylesterase inhibitor, Cholestanetriol 26-monooxygenase inhibitor, Alkylacetylglycerophosphatase inhibitor, Threonine aldolase inhibitor, Exoribonuclease II inhibitor, Kidney function stimulant, Chymosin inhibitor, Acrocylindropepsin inhibitor, Saccharopepsin inhibitor which are found from PASS prediction using structural activity relationship analysis online tool (https://www.way2drug.com/passonline/predict.php).

Despite the limitations imposed by resource constraints, further empirical investigations are indispensable for conclusively validating the compound-protein interactions identified in our *in-silico* modelling study. Our computational findings establish an excellent basis for future research, providing invaluable insights into potential therapeutic targets. The in silico data presented herein can serve as a valuable resource for researchers seeking to explore the antiviral potential of these compounds. By capitalizing on our findings, subsequent in vitro and in vivo studies can be conducted with greater precision and efficiency, potentially expediting the development of innovative therapeutics for BDV infections.

## 5. Conclusion

This study explored the potential of *in-silico* drug design to identify novel therapeutic options for Borna disease, an untreatable neurological condition findings from our study highlight the promise of phytochemicals, naturally occurring compounds, as potential antiviral agents found in *Mangifera indica* antiviral agents. Using computer-aided techniques like molecular docking, pharmacokinetics, toxicity, MD simulation, post-docking MM-GBSA, DCCM, and PCA, 3,4-Dihydroxy-5-oxocyclohex-3-ene-1-carboxylic acid (CID 20871246-IMPHY007896) found in *Mangifera indica* was identified as a promising lead for Borna disease. Additionally, these findings illuminate the potential of *in-silico* approaches for rapid and efficient drug discovery. Furthermore, *in vitro* and *in vivo* investigations are crucial to validate this result and establish the safety and effectiveness of this phytochemical compound. This paves the way for the development of much-needed therapies for Borna disease infection inhibiting viral replication and transcription.

## Supporting information

S1 File Similar compounds based on the control ligand Favipiravir, ADME & toxicity properties analysis of 80 phytochemical compounds. (DOCX)

S2 File Docking scores. (CSV)

S3 File MM-GBSA scores. (CSV)

S4 File Protein RMSD. (XLSX)

S5 File Protein RMSF. (XLSX)

S6 File Ligand properties. (XLSX)
