# Supplementary material for: Nature’s defense against emerging neurodegenerative threats: Dynamic simulation, PCA, DCCM identified potential plant-based antiviral lead targeting borna disease virus nucleoprotein
Source: PLoS One. 2024 Dec 30;19(12):e0310802. doi: 10.1371/journal.pone.0310802 (PMC11684711; doi:10.1371/journal.pone.0310802)
Supplement: S1 File — (DOCX) [file pone.0310802.s001.docx]

**Nature's Defense Against Emerging Neurodegenerative Threats: Dynamic Simulation, PCA, DCCM Identified 3,4-Dihydroxy-5-Oxocyclohex-3-Ene-1-Carboxylic Acid as a Potential Plant-Based Antiviral Lead Targeting Borna Disease Virus Nucleoprotein**

**Supplementary Tables:**

**Supplementary Table 1.** Similar compounds based on the control ligand Favipiravir

**Supplementary Table 2.** ADME properties analysis of 80 phytochemical compounds.

**Supplementary Table 3.** Toxicity properties analysis of 80 phytochemical compounds.

1. **Supplementary Table 1:** Similar compounds based on the control ligand Favipiravir

| ***1. Plant Name: Ocimum americanum*** | | | |
| --- | --- | --- | --- |
| **IMPPAT Phytochemical identifier:** | **Phytochemical name:** | **SMILES:** | **CID** |
| IMPHY000099 | Myrtenol | OCC1=CCC2CC1C2(C)C | [CID : 10582](cid:10582) |
| IMPHY000302 | Maalialcohol | C[C@]1(O)CCC[C@@]2([C@@H]1[C@H]1[C@H](C1(C)C)CC2)C | CID:10944069 |
| IMPHY000399 | beta-Bisabolene | CC(=CCCC(=C)[C@H]1CCC(=CC1)C)C | [CID:10104370](cid:10104370) |
| IMPHY001915 | Octadecane | CCCCCCCCCCCCCCCCCC | [CID:11635](https://pubchem.ncbi.nlm.nih.gov/compound/11635) |
| IMPHY002072 | Pinocarvone | C=C1C(=O)CC2CC1C2(C)C | [CID:121719](cid:121719) |
| IMPHY002825 | 2-(4-Methylphenyl)propan-2-ol | Cc1ccc(cc1)C(O)(C)C | CID:14529 |
| IMPHY003179 | 3-Octanone | CCCCCC(=O)CC | CID:246728 |
| IMPHY003485 | Myrcene | C=CC(=C)CCC=C(C)C | CID:31253 |
| IMPHY003536 | Eugenol | C=CCc1ccc(c(c1)OC)O | CID:3314 |
| IMPHY003616 | Bicyclogermacrene | C/C/1=CCC/C(=C/[C@H]2[C@@H](CC1)C2(C)C)/C | CID:13894537 |
| IMPHY003685 | trans-1(7),8-p-Menthadien-2-ol | CC(=C)[C@@H]1CCC(=C)[C@H](C1)O | CID:6428442 |
| IMPHY003695 | (-)-Germacrene A | C/C/1=CCC/C(=C/C[C@H](CC1)C(=C)C)/C | [CID:9548706](cid:9548706) |
| IMPHY003710 | (-)-Isopulegol | C[C@@H]1CC[C@H]([C@@H](C1)O)C(=C)C | CID:170833 |
| IMPHY003719 | beta-Copaene | CC([C@@H]1CC[C@]2([C@@H]3[C@H]1C2C(=C)CC3)C)C | CID:57339298 |
| IMPHY003798 | (Z)-alpha-Bisabolene | CC(=CC/C=C(C1CCC(=CC1)C)/C)C | [CID:5352653](cid:5352653) |
| IMPHY003822 | Cubebol | CC([C@@H]1CC[C@H]([C@]23[C@H]1[C@H]2[C@@](C)(O)CC3)C)C | CID:11276107 |
| IMPHY003977 | (-)-beta-Bourbonene | CC([C@@H]1CC[C@@]2([C@H]1[C@H]1C(=C)CC[C@@H]21)C)C | CID:62566 |
| IMPHY003982 | gamma-Terpinene | CC1=CCC(=CC1)C(C)C | CID:7461 |
| IMPHY004077 | Verbenone | CC1=CC(=O)C2CC1C2(C)C | [CID:29025](cid:29025) |
| IMPHY004281 | Guaiol | C[C@H]1CC[C@H](CC2=C1CC[C@@H]2C)C(O)(C)C | CID:227829 |
| IMPHY004372 | Selina-4(15),7(11)-diene | C=C1CCC[C@]2([C@H]1CC(=C(C)C)CC2)C | CID:10655819 |
| IMPHY004541 | 3-Octyl acetate | CCCCCC(OC(=O)C)CC | CID:521238 |
| IMPHY005345 | 1-Octen-3-OL | CCCCCC(C=C)O | CID:18827 |
| IMPHY006145 | p-Cymene | Cc1ccc(cc1)C(C)C | CID:7463 |
| IMPHY006325 | Myrtenal | O=CC1=CCC2CC1C2(C)C | CID:61130 |
| IMPHY006550 | Thymol | Cc1ccc(c(c1)O)C(C)C | CID:6989 |
| IMPHY006696 | Methyleugenol | C=CCc1ccc(c(c1)OC)OC | CID:7127 |
| IMPHY006709 | Acetyleugenol | C=CCc1ccc(c(c1)OC)OC(=O)C | CID:7136 |
| IMPHY006944 | Estragole | COc1ccc(cc1)CC=C | CID:8815 |
| IMPHY006948 | beta-Terpineol | CC(=C)C1CCC(CC1)(C)O | CID:8748 |
| IMPHY006950 | Tricyclene | CC12C3C1CC(C2(C)C)C3 | CID:79035 |
| IMPHY006951 | Eicosane | CCCCCCCCCCCCCCCCCCCC | CID:8222 |
| IMPHY006965 | alpha,alpha-Dimethyl-4-methylenecyclohexanemethanol | CC(C1CCC(=C)CC1)(O)C | CID:81722 |
| IMPHY007067 | Linalyl acetate | C=CC(OC(=O)C)(CCC=C(C)C)C | [CID:8294](cid:8294) |
| IMPHY007317 | 4-Allylphenol | C=CCc1ccc(cc1)O | CID:68148 |
| IMPHY007376 | beta-Cubebene | CC([C@@H]1CC[C@H]([C@]23[C@H]1[C@H]2C(=C)CC3)C)C | CID:93081 |
| IMPHY007606 | Thymohydroquinone | CC(c1cc(O)c(cc1O)C)C | CID:95779 |
| IMPHY007840 | Spathulenol | C=C1CC[C@@H]2[C@H]([C@H]3[C@H]1CC[C@]3(C)O)C2(C)C | CID:92231 |
| IMPHY009355 | Tetracosane | CCCCCCCCCCCCCCCCCCCCCCCC | CID:12592 |
| IMPHY009359 | Hexacosane | CCCCCCCCCCCCCCCCCCCCCCCCCC | CID:12407 |
| IMPHY009369 | Nonadecane | CCCCCCCCCCCCCCCCCCC | CID:12401 |
| IMPHY009481 | Octacosane | CCCCCCCCCCCCCCCCCCCCCCCCCCCC | CID:12408 |
| IMPHY009739 | 5-Isopropylbicyclo[3.1.0]hexan-2-one | O=C1CCC2(C1C2)C(C)C | CID:92784 |
| IMPHY009743 | beta-Gurjunene | C[C@@H]1CC[C@@H]2[C@H]([C@H]3[C@@H]1CCC3=C)C2(C)C | CID:6450812 |
| IMPHY009840 | Cyclosativene | CC(C1CCC2(C3C1C1C(C21C)C3)C)C | CID:519960 |
| IMPHY009853 | Naphthalene | c1ccc2c(c1)cccc2 | CID:931 |
| IMPHY009874 | Myrtenyl acetate | CC(=O)OCC1=CCC2CC1C2(C)C | CID:61262 |
| IMPHY009961 | Ethyl isovalerate | CCOC(=O)CC(C)C | CID:7945 |
| IMPHY010072 | Eucalyptol | CC12CCC(CC1)C(O2)(C)C | CID:2758 |
| IMPHY010080 | beta-Elemene | C=C[C@]1(C)CC[C@H](C[C@H]1C(=C)C)C(=C)C | CID:6918391 |
| IMPHY010815 | Thujopsene | CC1=CC[C@]2([C@@]3([C@H]1C3)C(C)(C)CCC2)C | CID:442402 |
| IMPHY011070 | 1-Octenyl acetate | CCCCCC/C=C/OC(=O)C | CID:5352540 |
| IMPHY011345 | (1S,4R,5R)-Thujane-4-ol acetate | CC(=O)O[C@]1(C)CC[C@@]2([C@H]1C2)C(C)C | CID:20837672 |
| IMPHY011396 | 4-Carvomenthenol | CC1=CCC(CC1)(O)C(C)C | CID:11230 |
| IMPHY011519 | alpha-Terpinyl acetate | CC(=O)OC(C1CCC(=CC1)C)(C)C | CID:111037 |
| IMPHY011542 | beta-Eudesmol | C=C1CCC[C@]2([C@H]1C[C@@H](CC2)C(O)(C)C)C | CID:91457 |
| IMPHY011552 | (1R)-2-methyl-5-propan-2-ylbicyclo[3.1.0]hex-2-ene | CC1=CCC2([C@@H]1C2)C(C)C | CID:6451618 |
| IMPHY011564 | Germacra-1(10),5-dien-4-ol | C/C/1=C/CC[C@@](C)(O)/C=C[C@@H](CC1)C(C)C | CID:101589676 |
| IMPHY011568 | alpha-Fenchyl acetate | CC(=O)O[C@@H]1[C@]2(C)CC[C@H](C1(C)C)C2 | CID:7530950 |
| IMPHY011581 | alpha-Selinene | CC1=CCC[C@]2([C@H]1C[C@@H](CC2)C(=C)C)C | CID:10856614 |
| IMPHY011586 | (S,1Z,6Z)-8-Isopropyl-1-methyl-5-methylenecyclodeca-1,6-diene | C/C/1=C/CCC(=C)/C=C[C@@H](CC1)C(C)C | CID:91723653 |
| IMPHY011590 | d-Borneol | O[C@@H]1C[C@H]2C([C@@]1(C)CC2)(C)C | CID:61060 |
| IMPHY011599 | Terpinolene | CC1=CCC(=C(C)C)CC1 | CID:11463 |
| IMPHY011643 | alpha-Terpinene | CC1=CC=C(CC1)C(C)C | CID:7462 |
| IMPHY011658 | beta-Farnesene | C=CC(=C)CC/C=C(/CCC=C(C)C)C | CID:5281517 |
| IMPHY011659 | alpha-Muurolene | CC1=C[C@@H]2[C@H](CC1)C(=CC[C@H]2C(C)C)C | CID:12306047 |
| IMPHY011660 | (+)-alpha-Cadinene | CC1=C[C@@H]2[C@@H](CC1)C(=CC[C@H]2C(C)C)C | CID:12306048 |
| IMPHY011667 | alpha-Gurjunene | C[C@@H]1CC[C@@H]2[C@H](C3=C(CC[C@H]13)C)C2(C)C | CID:15560276 |
| IMPHY011687 | trans-3-Hexenyl acetate | CC/C=C/CCOC(=O)C | CID:5352557 |
| IMPHY011714 | Methyl cinnamate | COC(=O)/C=C/c1ccccc1 | [CID:637520](cid:637520) |
| IMPHY011749 | Humulene epoxide II | C/C/1=CCC(C)(C)/C=C/C[C@@]2([C@@H](CC1)O2)C | CID:10704181 |
| IMPHY011761 | Humulene | C/C/1=CCC(C)(C)/C=C/C/C(=C/CC1)/C | [CID:5281520](cid:5281520) |
| IMPHY011763 | Anethole | C/C=C/c1ccc(cc1)OC | CID:637563 |
| IMPHY011789 | Citral | O=C/C=C(/CCC=C(C)C)C | CID:638011 |
| IMPHY011790 | Neral | O=C/C=C(CCC=C(C)C)/C | CID:643779 |
| IMPHY011792 | gamma-Muurolene | CC1=C[C@@H]2[C@H](CC1)C(=C)CC[C@H]2C(C)C | CID:12313020 |
| IMPHY011793 | (+)-gamma-Cadinene | CC1=C[C@@H]2[C@@H](CC1)C(=C)CC[C@H]2C(C)C | CID:6432404 |
| IMPHY011804 | cis-3-Hexenyl acetate | CC/C=CCCOC(=O)C | CID:5363388 |
| IMPHY011811 | (Z)-Methyl cinnamate | COC(=O)/C=Cc1ccccc1 | CID:6428458 |
| IMPHY011817 | alpha-Farnesene | C=C/C(=C/C/C=C(/CCC=C(C)C)C)/C | CID:5281516 |
| IMPHY011890 | Elemol | C=C[C@]1(C)CC[C@H](C[C@H]1C(=C)C)C(O)(C)C | CID:92138 |
| IMPHY011938 | gamma-Eudesmol | CC1=C2C[C@@H](CC[C@]2(CCC1)C)C(O)(C)C | CID:6432005 |
| IMPHY011939 | 10-epi-gamma-Eudesmol | CC1=C2C[C@@H](CC[C@@]2(CCC1)C)C(O)(C)C | CID:6430754 |
| IMPHY011957 | (+)-delta-Cadinene | CC1=C[C@@H]2C(=C(C)CC[C@H]2C(C)C)CC1 | CID:441005 |
| IMPHY012036 | Camphor | O=C1CC2C(C1(C)CC2)(C)C | CID:2537 |
| IMPHY012058 | Linalool | C=CC(CCC=C(C)C)(O)C | CID:6549 |
| IMPHY012061 | alpha-Pinene | CC1=CCC2CC1C2(C)C | CID:6654 |
| IMPHY012147 | beta-Pinene | C=C1CCC2CC1C2(C)C | CID:14896 |
| IMPHY012160 | alpha-Terpineol | CC1=CCC(CC1)C(O)(C)C | CID:17100 |
| IMPHY012165 | Sabinene | C=C1CCC2(C1C2)C(C)C | CID:18818 |
| IMPHY012168 | (1S,2S,6S,7R,8R)-1,3-dimethyl-8-propan-2-yltricyclo[4.4.0.02,7]dec-3-ene | CC([C@H]1CC[C@]2([C@@H]3[C@@H]1[C@H]2C(=CC3)C)C)C | CID:101607926 |
| IMPHY012586 | (-)-alpha-Cadinol | CC1=CC2C(CC1)[C@@](C)(O)CC[C@@H]2C(C)C | CID:6431302 |
| IMPHY012665 | Levomenol | CC(=CCC[C@@]([C@H]1CCC(=CC1)C)(O)C)C | CID:442343 |
| IMPHY012667 | Caryophyllene oxide | C=C1CC[C@H]2O[C@@]2(CC[C@@H]2[C@@H]1CC2(C)C)C | CID:1742210 |
| IMPHY012739 | (Z)-beta-Ocimene | C=C/C(=CCC=C(C)C)/C | CID:5320250 |
| IMPHY012815 | Fokienol | C=CC(CC/C=C(/CCC/C=C/C(=C)C)C)(O)C | CID:5352449 |
| IMPHY012920 | 2-Furanmethanol, 5-ethenyltetrahydro-alpha,alpha,5-trimethyl-, cis- | C=C[C@@]1(C)CC[C@H](O1)C(O)(C)C | CID:11116492 |
| IMPHY012921 | gamma-Elemene | C=C[C@]1(C)CCC(=C(C)C)C[C@H]1C(=C)C | CID:6432312 |
| IMPHY013093 | delta-Elemene | C=C[C@@]1(C)CCC(=C[C@@H]1C(=C)C)C(C)C | CID:12309449 |
| IMPHY013133 | (Z)-p-Menth-2-en-1-ol | CC([C@@H]1CC[C@](C=C1)(C)O)C | CID:13918681 |
| IMPHY014708 | beta-Selinene | C=C1CCC[C@]2([C@H]1C[C@@H](CC2)C(=C)C)C | CID:442393 |
| IMPHY014749 | (E,Z)-2,4-Decadienal | CCCCC/C=CC=CC=O | CID:6427087 |
| IMPHY014805 | Cedr-8-ene | CC1=CC[C@@]23C[C@@H]1C(C)(C)[C@@H]2CC[C@H]3C | CID:6431015 |
| IMPHY014806 | Caswell No. 264AB | CC([C@@H]1CC[C@H]([C@]23[C@H]1[C@H]2C(=CC3)C)C)C | CID:442359 |
| IMPHY014811 | alpha-Phellandrene | CC1=CCC(C=C1)C(C)C | CID:7460 |
| IMPHY014817 | Aromadendrene | CC1CCC2C1C1C(C1(C)C)CCC2=C | CID:91354 |
| IMPHY014831 | beta-Caryophyllene | C/C/1=CCCC(=C)[C@@H]2[C@@H](CC1)C(C2)(C)C | CID:5281515 |
| IMPHY014835 | (E)-beta-ocimene | C=C/C(=C/CC=C(C)C)/C | CID:5281553 |
| IMPHY014847 | Bornyl acetate | CC(=O)OC1CC2C(C1(C)CC2)(C)C | [CID:6448](cid:6448) |
| MPHY014852 | Camphene | C=C1C2CCC(C1(C)C)C2 | [CID:6616](cid:6616) |
| IMPHY014863 | cis-alpha-Bergamotene | CC(=CCCC1(C)[C@@H]2CC=C([C@H]1C2)C)C | CID:91753502 |
| IMPHY014874 | cis-Sabinene hydrate | C[C@@H]1CC[C@@]2(C1C2)C(C)C | CID:101629835 |
| IMPHY014906 | Cedrelanol | CC1=C[C@@H]2[C@@H](CC1)[C@@](C)(O)CC[C@H]2C(C)C | CID:160799 |
| IMPHY014923 | Geraniol | OC/C=C(/CCC=C(C)C)C | CID:637566 |
| IMPHY014988 | Limonene | CC1=CCC(CC1)C(=C)C | CID:22311 |
| IMPHY014989 | trans-Linalool oxide | C=C[C@]1(C)CC[C@H](O1)C(O)(C)C | CID:6432254 |
| IMPHY015016 | alpha-Muurolol | CC1=C[C@@H]2[C@H](CC1)[C@](C)(O)CC[C@@H]2C(C)C | CID:91753440 |
| IMPHY015022 | Nerolidol | C=CC(CC/C=C(/CCC=C(C)C)C)(O)C | CID:5284507 |
| IMPHY015095 | 2-Cyclohexen-1-ol, 1-methyl-4-(1-methylethyl)-, trans- | CC([C@@H]1CC[C@@](C=C1)(C)O)C | CID:122484 |
| IMPHY015123 | alpha-Copaene | CC([C@@H]1CC[C@]2([C@@H]3[C@H]1C2C(=CC3)C)C)C | CID:70678558 |
| IMPHY016027 | trans-Sabinene hydrate | CC([C@@]12CC[C@](C2C1)(C)O)C | CID:12315151 |
| IMPHY016054 | trans-alpha-Bergamotene | CC(=CCC[C@]1(C)[C@H]2CC=C([C@@H]1C2)C)C | CID:6429302 |
| IMPHY000099 | Myrtenol | OCC1=CCC2CC1C2(C)C | CID:10582 |
| IMPHY000399 | beta-Bisabolene | CC(=CCCC(=C)[C@H]1CCC(=CC1)C)C | CID:10104370 |
| IMPHY000795 | Octanal | CCCCCCCC=O | CID:454 |
| IMPHY001711 | Salvigenin | COc1ccc(cc1)c1cc(=O)c2c(o1)cc(c(c2O)OC)OC | [CID:161271](cid:161271) |
| IMPHY001776 | Nevadensin | COc1ccc(cc1)c1cc(=O)c2c(o1)c(OC)c(c(c2O)OC)O | CID:160921 |
| IMPHY001828 | 3-Octanol | CCCCCC(CC)O | CID:11527 |
| IMPHY002825 | 2-(4-Methylphenyl)propan-2-ol | Cc1ccc(cc1)C(O)(C)C | CID:14529 |
| IMPHY003482 | 4-Methoxybenzaldehyde | COc1ccc(cc1)C=O | CID:31244 |
| IMPHY003485 | Myrcene | C=CC(=C)CCC=C(C)C | CID:31253 |
| IMPHY003525 | Nonanal | CCCCCCCCC=O | CID:31289 |
| IMPHY003536 | Eugenol | C=CCc1ccc(c(c1)OC)O | CID:3314 |
| IMPHY003537 | Tetradecanal | CCCCCCCCCCCCCC=O | CID:31291 |
| IMPHY003616 | Bicyclogermacrene | C/C/1=CCC/C(=C/[C@H]2[C@@H](CC1)C2(C)C)/C | CID:13894537 |
| IMPHY003695 | (-)-Germacrene A | C/C/1=CCC/C(=C/C[C@H](CC1)C(=C)C)/C | CID:9548706 |
| IMPHY003710 | (-)-Isopulegol | C[C@@H]1CC[C@H]([C@@H](C1)O)C(=C)C | CID:170833 |
| IMPHY003719 | beta-Copaene | CC([C@@H]1CC[C@]2([C@@H]3[C@H]1C2C(=C)CC3)C)C | CID:57339298 |
| IMPHY003807 | (E)-alpha-bisabolene | CC(=CC/C=C(/C1CCC(=CC1)C)C)C | CID:5315468 |
| IMPHY003822 | Cubebol | CC([C@@H]1CC[C@H]([C@]23[C@H]1[C@H]2[C@@](C)(O)CC3)C)C | CID:11276107 |
| IMPHY003982 | gamma-Terpinene | CC1=CCC(=CC1)C(C)C | CID:7461 |
| IMPHY004077 | Verbenone | CC1=CC(=O)C2CC1C2(C)C | CID:29025 |
| IMPHY004151 | Geranyl formate | O=COC/C=C(/CCC=C(C)C)C | CID:5282109 |
| IMPHY004281 | Guaiol | C[C@H]1CC[C@H](CC2=C1CC[C@@H]2C)C(O)(C)C | CID:227829 |
| IMPHY005345 | 1-Octen-3-OL | CCCCCC(C=C)O | CID:18827 |
| IMPHY005390 | 1-Octen-3-yl acetate | CCCCCC(OC(=O)C)C=C | CID:17121 |
| IMPHY006145 | p-Cymene | Cc1ccc(cc1)C(C)C | CID:7463 |
| IMPHY006177 | Methyl geranate | COC(=O)/C=C(/CCC=C(C)C)C | CID:5365910 |
| IMPHY006696 | Methyleugenol | C=CCc1ccc(c(c1)OC)OC | CID:7127 |
| IMPHY006944 | Estragole | COc1ccc(cc1)CC=C | CID:8815 |
| IMPHY006950 | Tricyclene | CC12C3C1CC(C2(C)C)C3 | CID:79035 |
| IMPHY007067 | Linalyl acetate | C=CC(OC(=O)C)(CCC=C(C)C)C | CID:8294 |
| IMPHY007214 | Octyl acetate | CCCCCCCCOC(=O)C | CID:8164 |
| IMPHY007317 | 4-Allylphenol | C=CCc1ccc(cc1)O | CID:68148 |
| IMPHY007331 | 6-Methyl-5-hepten-2-one | CC(=O)CCC=C(C)C | CID:9862 |
| IMPHY007366 | alpha-Santalene | CC(=CCCC1(C)C2CC3C1(C)C3C2)C | CID:94164 |
| IMPHY007376 | beta-Cubebene | CC([C@@H]1CC[C@H]([C@]23[C@H]1[C@H]2C(=C)CC3)C)C | CID:93081 |
| IMPHY007421 | Citronellyl acetate | CC(CCC=C(C)C)CCOC(=O)C | CID:9017 |
| IMPHY007620 | 1-Octanol | CCCCCCCCO | CID:957 |
| IMPHY007840 | Spathulenol | C=C1CC[C@@H]2[C@H]([C@H]3[C@H]1CC[C@]3(C)O)C2(C)C | CID:92231 |
| IMPHY008936 | alpha-Guaiene | CC(=C)[C@@H]1CC[C@@H](C2=C(C1)[C@@H](C)CC2)C | CID:5317844 |
| IMPHY008946 | delta-Guaiene | CC(=C)[C@@H]1CCC(=C2[C@@H](C1)[C@@H](C)CC2)C | CID:94275 |
| IMPHY009757 | 6,6-Dimethyl-2-methylenebicyclo[3.1.1]hept-3-yl acetate | CC(=O)OC1CC2CC(C1=C)C2(C)C | CID:102553 |
| IMPHY010015 | epi-Longipinanol | C[C@H]1C2CC[C@]([C@H]3C1CCC(C23)(C)C)(C)O | CID:91746617 |
| IMPHY010072 | Eucalyptol | CC12CCC(CC1)C(O2)(C)C | CID:2758 |
| IMPHY010080 | beta-Elemene | C=C[C@]1(C)CC[C@H](C[C@H]1C(=C)C)C(=C)C | CID:6918391 |
| IMPHY010815 | Thujopsene | CC1=CC[C@]2([C@@]3([C@H]1C3)C(C)(C)CCC2)C | CID:442402 |
| IMPHY011396 | 4-Carvomenthenol | CC1=CCC(CC1)(O)C(C)C | CID:11230 |
| IMPHY011451 | cis-2-Pinanol | C[C@]1(O)CC[C@H]2C[C@@H]1C2(C)C | CID:6428289 |
| IMPHY011542 | beta-Eudesmol | C=C1CCC[C@]2([C@H]1C[C@@H](CC2)C(O)(C)C)C | CID:91457 |
| IMPHY011552 | (1R)-2-methyl-5-propan-2-ylbicyclo[3.1.0]hex-2-ene | CC1=CCC2([C@@H]1C2)C(C)C | CID:6451618 |
| IMPHY011564 | Germacra-1(10),5-dien-4-ol | C/C/1=C/CC[C@@](C)(O)/C=C[C@@H](CC1)C(C)C | CID:101589676 |
| IMPHY011581 | alpha-Selinene | CC1=CCC[C@]2([C@H]1C[C@@H](CC2)C(=C)C)C | CID:10856615 |
| IMPHY011586 | (S,1Z,6Z)-8-Isopropyl-1-methyl-5-methylenecyclodeca-1,6-diene | C/C/1=C/CCC(=C)/C=C[C@@H](CC1)C(C)C | CID:91723653 |
| IMPHY011590 | d-Borneol | O[C@@H]1C[C@H]2C([C@@]1(C)CC2)(C)C | CID:61060 |
| IMPHY011599 | Terpinolene | CC1=CCC(=C(C)C)CC1 | CID:11463 |
| IMPHY011632 | Farnesol | OC/C=C(/CC/C=C(/CCC=C(C)C)C)C | CID:445070 |
| IMPHY011643 | alpha-Terpinene | CC1=CC=C(CC1)C(C)C | CID:7462 |
| IMPHY011647 | Geranyl acetate | C/C(=CCOC(=O)C)/CCC=C(C)C | CID:1549026 |
| IMPHY011648 | Neryl acetate | C/C(=C/COC(=O)C)/CCC=C(C)C | CID:1549025 |
| IMPHY011657 | cis-beta-Farnesene | C=CC(=C)CC/C=C(CCC=C(C)C)/C | CID:5317319 |
| IMPHY011658 | beta-Farnesene | C=CC(=C)CC/C=C(/CCC=C(C)C)C | CID:5281517 |
| IMPHY011659 | alpha-Muurolene | CC1=C[C@@H]2[C@H](CC1)C(=CC[C@H]2C(C)C)C | CID:12306047 |
| IMPHY011660 | (+)-alpha-Cadinene | CC1=C[C@@H]2[C@@H](CC1)C(=CC[C@H]2C(C)C)C | CID:12306048 |
| IMPHY011667 | alpha-Gurjunene | C[C@@H]1CC[C@@H]2[C@H](C3=C(CC[C@H]13)C)C2(C)C | CID:15560276 |
| IMPHY011687 | trans-3-Hexenyl acetate | CC/C=C/CCOC(=O)C | CID:5352557 |
| IMPHY011692 | trans-3-Hexen-1-ol | OCC/C=C/CC | CID:5284503 |
| IMPHY011709 | alpha-Eudesmol | CC1=CCC[C@]2([C@H]1C[C@@H](CC2)C(O)(C)C)C | CID:92762 |
| IMPHY011714 | Methyl cinnamate | COC(=O)/C=C/c1ccccc1 | CID:637520 |
| IMPHY011749 | Humulene epoxide II | C/C/1=CCC(C)(C)/C=C/C[C@@]2([C@@H](CC1)O2)C | CID:10704181 |
| IMPHY011761 | Humulene | C/C/1=CCC(C)(C)/C=C/C/C(=C/CC1)/C | CID:5281520 |
| IMPHY011789 | Citral | O=C/C=C(/CCC=C(C)C)C | CID:638011 |
| IMPHY011790 | Neral | O=C/C=C(CCC=C(C)C)/C | CID:643779 |
| IMPHY011792 | gamma-Muurolene | CC1=C[C@@H]2[C@H](CC1)C(=C)CC[C@H]2C(C)C | CID:12313020 |
| IMPHY011793 | (+)-gamma-Cadinene | CC1=C[C@@H]2[C@@H](CC1)C(=C)CC[C@H]2C(C)C | CID:6432404 |
| IMPHY011826 | Oleanolic acid | O[C@H]1CC[C@]2([C@H](C1(C)C)CC[C@@]1([C@@H]2CC=C2[C@@]1(C)CC[C@@]1([C@H]2CC(C)(C)CC1)C(=O)O)C)C | CID:10494 |
| IMPHY011880 | Ursolic acid | C[C@@H]1CC[C@]2([C@@H]([C@H]1C)C1=CC[C@H]3[C@@]([C@@]1(CC2)C)(C)CC[C@@H]1[C@]3(C)CC[C@@H](C1(C)C)O)C(=O)O | CID:64945 |
| IMPHY011890 | Elemol | C=C[C@]1(C)CC[C@H](C[C@H]1C(=C)C)C(O)(C)C | CID:92138 |
| IMPHY011894 | (-)-7-Epi-alpha-selinene | CC1=CCC[C@]2([C@H]1C[C@H](CC2)C(=C)C)C | CID:10726905 |
| IMPHY011939 | 10-epi-gamma-Eudesmol | CC1=C2C[C@@H](CC[C@@]2(CCC1)C)C(O)(C)C | [CID:6430754](cid:6430754) |
| IMPHY011957 | (+)-delta-Cadinene | CC1=C[C@@H]2C(=C(C)CC[C@H]2C(C)C)CC1 | CID:441005 |
| IMPHY012036 | Camphor | O=C1CC2C(C1(C)CC2)(C)C | CID:2537 |
| IMPHY012058 | Linalool | C=CC(CCC=C(C)C)(O)C | CID:6549 |
| IMPHY012061 | alpha-Pinene | CC1=CCC2CC1C2(C)C | CID:6654 |
| IMPHY012086 | Citronellal | O=CCC(CCC=C(C)C)C | CID:7794 |
| IMPHY012104 | Citronellol (other chemical name- beta-citronellol) | OCCC(CCC=C(C)C)C | CID:8842 |
| IMPHY012147 | beta-Pinene | C=C1CCC2CC1C2(C)C | CID:14896 |
| IMPHY012160 | alpha-Terpineol | CC1=CCC(CC1)C(O)(C)C | CID:17100 |
| IMPHY012165 | Sabinene | C=C1CCC2(C1C2)C(C)C | CID:18818 |
| IMPHY012279 | alpha-Curcumene | CC(=CCCC(c1ccc(cc1)C)C)C | CID:92139 |
| IMPHY012586 | (-)-alpha-Cadinol | CC1=CC2C(CC1)[C@@](C)(O)CC[C@@H]2C(C)C | CID:6431302 |
| IMPHY012587 | tau-Cadinol | CC1=C[C@H]2[C@H](CC1)[C@](C)(O)CC[C@H]2C(C)C | CID:12302222 |
| IMPHY012589 | 3-(1,5-Dimethyl-4-hexenyl)-6-methylene-1-cyclohexene | CC(C1CCC(=C)C=C1)CCC=C(C)C | CID:519764 |
| IMPHY012596 | Selin-11-en-4alpha-ol | CC(=C)[C@@H]1CC[C@@]2([C@@H](C1)[C@](C)(O)CCC2)C | CID:15560330 |
| IMPHY012654 | Nerol | OC/C=C(CCC=C(C)C)/C | CID:643820 |
| IMPHY012667 | Caryophyllene oxide | C=C1CC[C@H]2O[C@@]2(CC[C@@H]2[C@@H]1CC2(C)C)C | CID:1742210 |
| IMPHY012737 | (1S,4E,9S)-4,11,11-trimethyl-8-methylidenebicyclo[7.2.0]undec-4-ene | C/C/1=CCCC(=C)[C@@H]2[C@H](CC1)C(C2)(C)C | CID:6429301 |
| IMPHY012739 | (Z)-beta-Ocimene | C=C/C(=CCC=C(C)C)/C | [CID:5320250](cid:5320250) |
| IMPHY012815 | Fokienol | C=CC(CC/C=C(/CCC/C=C/C(=C)C)C)(O)C | CID:5352449 |
| IMPHY012920 | 2-Furanmethanol, 5-ethenyltetrahydro-alpha,alpha,5-trimethyl-, cis- | C=C[C@@]1(C)CC[C@H](O1)C(O)(C)C | CID:11116492 |
| IMPHY012921 | gamma-Elemene | C=C[C@]1(C)CCC(=C(C)C)C[C@H]1C(=C)C | CID:6432313 |
| IMPHY013133 | (Z)-p-Menth-2-en-1-ol | CC([C@@H]1CC[C@](C=C1)(C)O)C | CID:13918681 |
| IMPHY013836 | Fenchone | O=C1C2(C)CCC(C1(C)C)C2 | CID:14525 |
| IMPHY013972 | 1,10-Di-epcubenol | CC1=C[C@@H]2[C@](CC1)(O)[C@H](C)CC[C@@H]2C(C)C | CID:91748749 |
| IMPHY014690 | (-)-Globulol | C[C@@H]1CC[C@@H]2[C@@H]1[C@H]1[C@H](C1(C)C)CC[C@@]2(C)O | CID:12304985 |
| IMPHY014708 | beta-Selinene | C=C1CCC[C@]2([C@H]1C[C@@H](CC2)C(=C)C)C | CID:442393 |
| IMPHY014806 | Caswell No. 264AB | CC([C@@H]1CC[C@H]([C@]23[C@H]1[C@H]2C(=CC3)C)C)C | CID:442359 |
| IMPHY014811 | alpha-Phellandrene | CC1=CCC(C=C1)C(C)C | CID:7460 |
| IMPHY014831 | beta-Caryophyllene | C/C/1=CCCC(=C)[C@@H]2[C@@H](CC1)C(C2)(C)C | CID:5281515 |
| IMPHY014835 | (E)-beta-ocimene | C=C/C(=C/CC=C(C)C)/C | CID:5281553 |
| IMPHY014847 | Bornyl acetate | CC(=O)OC1CC2C(C1(C)CC2)(C)C | CID:6448 |
| IMPHY014852 | Camphene | C=C1C2CCC(C1(C)C)C2 | CID:6616 |
| IMPHY014863 | cis-alpha-Bergamotene | CC(=CCCC1(C)[C@@H]2CC=C([C@H]1C2)C)C | CID:91753502 |
| IMPHY014870 | cis-Muurola-4(14),5-diene | C=C1CC[C@@H]2C(=C1)[C@H](CC[C@H]2C)C(C)C | CID:51351709 |
| IMPHY014874 | cis-Sabinene hydrate | C[C@@H]1CC[C@@]2(C1C2)C(C)C | CID:101629835 |
| IMPHY014876 | 7-epi-cis-Sesquisabinene hydrate | CC(=CCCC([C@]12CC[C@@](C2C1)(C)O)C)C | CID:6428435 |
| IMPHY014877 | (S)-cis-Verbenol | CC1=C[C@H](O)[C@H]2C[C@@H]1C2(C)C | CID:87839 |
| IMPHY014906 | Cedrelanol | CC1=C[C@@H]2[C@@H](CC1)[C@@](C)(O)CC[C@H]2C(C)C | CID:160799 |
| IMPHY014907 | 6-Epi-beta-bisabolol | CC(=CCC[C@@H]([C@@]1(O)CCC(=CC1)C)C)C | CID:12300148 |
| IMPHY014923 | Geraniol | OC/C=C(/CCC=C(C)C)C | CID:637566 |
| IMPHY014988 | Limonene | CC1=CCC(CC1)C(=C)C | CID:22311 |
| IMPHY014989 | trans-Linalool oxide | C=C[C@]1(C)CC[C@H](O1)C(O)(C)C | CID:6432254 |
| IMPHY015016 | alpha-Muurolol | CC1=C[C@@H]2[C@H](CC1)[C@](C)(O)CC[C@@H]2C(C)C | CID:91753440 |
| IMPHY015022 | Nerolidol | C=CC(CC/C=C(/CCC=C(C)C)C)(O)C | CID:5284507 |
| IMPHY015095 | 2-Cyclohexen-1-ol, 1-methyl-4-(1-methylethyl)-, trans- | CC([C@@H]1CC[C@@](C=C1)(C)O)C | CID:122484 |
| IMPHY015098 | trans-Verbenol | CC1=C[C@H](O)[C@@H]2C[C@H]1C2(C)C | CID:89664 |
| IMPHY015123 | alpha-Copaene | CC([C@@H]1CC[C@]2([C@@H]3[C@H]1C2C(=CC3)C)C)C | CID:70678558 |
| IMPHY015128 | T-Muurolol | CC1=C[C@@H]2[C@H](CC1)[C@@](C)(O)CC[C@H]2C(C)C | CID:3084331 |
| IMPHY015168 | 14-Hydroxy-alpha-humulene | OCC1(C)/C=C/C/C(=C/CC/C(=C/C1)/C)/C | CID:5352485 |
| IMPHY015210 | 2,2-Dimethyl-3,4-octadienal | CCCC=C=CC(C=O)(C)C | CID:253228 |
| IMPHY016015 | Oct-3-en-1-ol | CCCCC=CCCO | CID:28937 |
| IMPHY016027 | trans-Sabinene hydrate | CC([C@@]12CC[C@](C2C1)(C)O)C | CID:12315151 |
| IMPHY016054 | trans-alpha-Bergamotene | CC(=CCC[C@]1(C)[C@H]2CC=C([C@@H]1C2)C)C | CID:6429302 |
| IMPHY017693 | Menthyl chavicol | C=CCc1ccc(c(c1)C1CC(C)CCC1C(C)C)O | CID:70235324 |
| IMPHY000061 | Patchouli alcohol | C[C@H]1CC[C@@]2([C@@]3([C@H]1C[C@H](C2(C)C)CC3)C)O | CID:10955174 |
| IMPHY000099 | Myrtenol | OCC1=CCC2CC1C2(C)C | CID:10582 |
| IMPHY000399 | beta-Bisabolene | CC(=CCCC(=C)[C@H]1CCC(=CC1)C)C | CID:10104370 |
| IMPHY000795 | Octanal | CCCCCCCC=O | CID:454 |
| IMPHY001084 | Citronellic acid | CC(CC(=O)O)CCC=C(C)C | CID:10402 |
| IMPHY001246 | Carvacrol | CC(c1ccc(c(c1)O)C)C | CID:10364 |
| IMPHY001711 | Salvigenin | COc1ccc(cc1)c1cc(=O)c2c(o1)cc(c(c2O)OC)OC | CID:161271 |
| IMPHY001776 | Nevadensin | COc1ccc(cc1)c1cc(=O)c2c(o1)c(OC)c(c(c2O)OC)O | CID:160921 |
| IMPHY001828 | 3-Octanol | CCCCCC(CC)O | CID:11527 |
| IMPHY001931 | Vanillin | COc1cc(C=O)ccc1O | CID:1183 |
| IMPHY002072 | Pinocarvone | C=C1C(=O)CC2CC1C2(C)C | CID:121719 |
| IMPHY002825 | 2-(4-Methylphenyl)propan-2-ol | Cc1ccc(cc1)C(O)(C)C | CID:14529 |
| IMPHY003482 | 4-Methoxybenzaldehyde | COc1ccc(cc1)C=O | CID:31244 |
| IMPHY003485 | Myrcene | C=CC(=C)CCC=C(C)C | CID:31253 |
| IMPHY003536 | Eugenol | C=CCc1ccc(c(c1)OC)O | CID:3314 |
| IMPHY003545 | 4-Isopropylbenzaldehyde | O=Cc1ccc(cc1)C(C)C | CID:326 |
| IMPHY003616 | Bicyclogermacrene | C/C/1=CCC/C(=C/[C@H]2[C@@H](CC1)C2(C)C)/C | CID:13894537 |
| IMPHY003710 | (-)-Isopulegol | C[C@@H]1CC[C@H]([C@@H](C1)O)C(=C)C | CID:170833 |
| IMPHY003719 | beta-Copaene | CC([C@@H]1CC[C@]2([C@@H]3[C@H]1C2C(=C)CC3)C)C | CID:57339298 |
| IMPHY003807 | (E)-alpha-bisabolene | CC(=CC/C=C(/C1CCC(=CC1)C)C)C | CID:5315468 |
| IMPHY003822 | Cubebol | CC([C@@H]1CC[C@H]([C@]23[C@H]1[C@H]2[C@@](C)(O)CC3)C)C | CID:11276107 |
| IMPHY003955 | (R)-4-Isopropylcyclohex-2-enone | CC([C@H]1CCC(=O)C=C1)C | CID:642520 |
| IMPHY003977 | (-)-beta-Bourbonene | CC([C@@H]1CC[C@@]2([C@H]1[C@H]1C(=C)CC[C@@H]21)C)C | CID:62566 |
| MPHY003982 | gamma-Terpinene | CC1=CCC(=CC1)C(C)C | CID:7461 |
| IMPHY004077 | Verbenone | CC1=CC(=O)C2CC1C2(C)C | CID:29025 |
| IMPHY004151 | Geranyl formate | O=COC/C=C(/CCC=C(C)C)C | CID:5282109 |
| IMPHY004281 | Guaiol | C[C@H]1CC[C@H](CC2=C1CC[C@@H]2C)C(O)(C)C | CID:227829 |
| IMPHY004549 | Safrole | C=CCc1ccc2c(c1)OCO2 | CID:5144 |
| IMPHY005345 | 1-Octen-3-OL | CCCCCC(C=C)O | CID:18827 |
| IMPHY005371 | Cirsilineol | COc1cc2oc(cc(=O)c2c(c1OC)O)c1ccc(c(c1)OC)O | CID:162464 |
| IMPHY005390 | 1-Octen-3-yl acetate | CCCCCC(OC(=O)C)C=C | CID:17121 |
| IMPHY005618 | Germacrene B | C/C/1=CCC/C(=C/CC(=C(C)C)CC1)/C | CID:5281519 |
| IMPHY006145 | p-Cymene | Cc1ccc(cc1)C(C)C | CID:7463 |
| IMPHY006325 | Myrtenal | O=CC1=CCC2CC1C2(C)C | CID:61130 |
| IMPHY006417 | 2,6-Dimethyl-2,4,6-octatriene | C/C=C(/C=C/C=C(C)C)C | CID:5368821 |
| IMPHY006550 | Thymol | Cc1ccc(c(c1)O)C(C)C | CID:6989 |
| IMPHY006696 | Methyleugenol | C=CCc1ccc(c(c1)OC)OC | CID:7127 |
| IMPHY006709 | Acetyleugenol | C=CCc1ccc(c(c1)OC)OC(=O)C | [CID:7136](cid:7136) |
| IMPHY006944 | Estragole | COc1ccc(cc1)CC=C | CID:8815 |
| IMPHY006948 | beta-Terpineol | CC(=C)C1CCC(CC1)(C)O | CID:8748 |
| IMPHY006950 | Tricyclene | CC12C3C1CC(C2(C)C)C3 | CID:79035 |
| IMPHY006965 | alpha,alpha-Dimethyl-4-methylenecyclohexanemethanol | CC(C1CCC(=C)CC1)(O)C | CID:81722 |
| IMPHY007067 | Linalyl acetate | C=CC(OC(=O)C)(CCC=C(C)C)C | CID:8294 |
| IMPHY007275 | 1,6-Germacradien-5-ol | C/C/1=C/CC(C(C)C)C(/C=C(CCC1)/C)O | CID:91748908 |
| IMPHY007317 | 4-Allylphenol | C=CCc1ccc(cc1)O | CID:68148 |
| IMPHY007331 | 6-Methyl-5-hepten-2-one | CC(=O)CCC=C(C)C | CID:9862 |
| IMPHY007376 | beta-Cubebene | CC([C@@H]1CC[C@H]([C@]23[C@H]1[C@H]2C(=C)CC3)C)C | CID:93081 |
| IMPHY007421 | Citronellyl acetate | CC(CCC=C(C)C)CCOC(=O)C | CID:9017 |
| IMPHY007620 | 1-Octanol | CCCCCCCCO | CID:957 |
| IMPHY007840 | Spathulenol | C=C1CC[C@@H]2[C@H]([C@H]3[C@H]1CC[C@]3(C)O)C2(C)C | CID:92231 |
| IMPHY008150 | 1-Methyl-4-(prop-1-en-2-yl)benzene | Cc1ccc(cc1)C(=C)C | CID:62385 |
| IMPHY008936 | alpha-Guaiene | CC(=C)[C@@H]1CC[C@@H](C2=C(C1)[C@@H](C)CC2)C | CID:5317844 |
| IMPHY008946 | delta-Guaiene | CC(=C)[C@@H]1CCC(=C2[C@@H](C1)[C@@H](C)CC2)C | [CID:94275](cid:94275) |
| IMPHY009389 | Pentadecane | CCCCCCCCCCCCCCC | CID:12391 |
| IMPHY009848 | Pogostol | CC1CCC2C1CC(CCC2(C)O)C(=C)C | CID:5320651 |
| IMPHY009874 | Myrtenyl acetate | CC(=O)OCC1=CCC2CC1C2(C)C | CID:61262 |
| IMPHY009955 | Cyclohexane | C1CCCCC1 | CID:8078 |
| IMPHY010072 | Eucalyptol | CC12CCC(CC1)C(O2)(C)C | CID:2758 |
| IMPHY010080 | beta-Elemene | C=C[C@]1(C)CC[C@H](C[C@H]1C(=C)C)C(=C)C | CID:6918391 |
| IMPHY010097 | Benzyl benzoate | O=C(c1ccccc1)OCc1ccccc1 | CID:2345 |
| IMPHY010815 | Thujopsene | CC1=CC[C@]2([C@@]3([C@H]1C3)C(C)(C)CCC2)C | CID:442402 |
| IMPHY011070 | 1-Octenyl acetate | CCCCCC/C=C/OC(=O)C | CID:5352540 |
| IMPHY011392 | 3-Carene | CC1=CCC2C(C1)C2(C)C | CID:26049 |
| IMPHY011396 | 4-Carvomenthenol | CC1=CCC(CC1)(O)C(C)C | CID:11230 |
| IMPHY011519 | alpha-Terpinyl acetate | CC(=O)OC(C1CCC(=CC1)C)(C)C | CID:111037 |
| IMPHY011542 | beta-Eudesmol | C=C1CCC[C@]2([C@H]1C[C@@H](CC2)C(O)(C)C)C | CID:91457 |
| IMPHY011552 | (1R)-2-methyl-5-propan-2-ylbicyclo[3.1.0]hex-2-ene | CC1=CCC2([C@@H]1C2)C(C)C | CID:6451618 |
| IMPHY011564 | Germacra-1(10),5-dien-4-ol | C/C/1=C/CC[C@@](C)(O)/C=C[C@@H](CC1)C(C)C | CID:101589676 |
| IMPHY011568 | alpha-Fenchyl acetate | CC(=O)O[C@@H]1[C@]2(C)CC[C@H](C1(C)C)C2 | CID:7530950 |
| IMPHY011581 | alpha-Selinene | CC1=CCC[C@]2([C@H]1C[C@@H](CC2)C(=C)C)C | CID:10856614 |
| IMPHY011586 | (S,1Z,6Z)-8-Isopropyl-1-methyl-5-methylenecyclodeca-1,6-diene | C/C/1=C/CCC(=C)/C=C[C@@H](CC1)C(C)C | CID:91723653 |
| IMPHY011590 | d-Borneol | O[C@@H]1C[C@H]2C([C@@]1(C)CC2)(C)C | CID:61060 |
| IMPHY011599 | Terpinolene | CC1=CCC(=C(C)C)CC1 | CID:11463 |
| IMPHY011632 | Farnesol | OC/C=C(/CC/C=C(/CCC=C(C)C)C)C | CID:445070 |
| IMPHY011643 | alpha-Terpinene | CC1=CC=C(CC1)C(C)C | CID:7462 |
| IMPHY011647 | Geranyl acetate | C/C(=CCOC(=O)C)/CCC=C(C)C | CID:1549026 |
| IMPHY011648 | Neryl acetate | C/C(=C/COC(=O)C)/CCC=C(C)C | CID:1549025 |
| IMPHY011657 | cis-beta-Farnesene | C=CC(=C)CC/C=C(CCC=C(C)C)/C | CID:5317319 |
| IMPHY011658 | beta-Farnesene | C=CC(=C)CC/C=C(/CCC=C(C)C)C | CID:5281517 |
| IMPHY011659 | alpha-Muurolene | CC1=C[C@@H]2[C@H](CC1)C(=CC[C@H]2C(C)C)C | CID:12306047 |
| IMPHY011660 | (+)-alpha-Cadinene | CC1=C[C@@H]2[C@@H](CC1)C(=CC[C@H]2C(C)C)C | CID:12306048 |
| IMPHY011667 | alpha-Gurjunene | C[C@@H]1CC[C@@H]2[C@H](C3=C(CC[C@H]13)C)C2(C)C | CID:15560276 |
| IMPHY011687 | trans-3-Hexenyl acetate | CC/C=C/CCOC(=O)C | CID:5352557 |
| IMPHY011692 | trans-3-Hexen-1-ol | OCC/C=C/CC | CID:5284503 |
| IMPHY011709 | alpha-Eudesmol | CC1=CCC[C@]2([C@H]1C[C@@H](CC2)C(O)(C)C)C | CID:92762 |
| IMPHY011714 | Methyl cinnamate | COC(=O)/C=C/c1ccccc1 | CID:637520 |
| IMPHY011749 | Humulene epoxide II | C/C/1=CCC(C)(C)/C=C/C[C@@]2([C@@H](CC1)O2)C | CID:10704181 |
| IMPHY011761 | Humulene | C/C/1=CCC(C)(C)/C=C/C/C(=C/CC1)/C | CID:5281520 |
| IMPHY011789 | Citral | O=C/C=C(/CCC=C(C)C)C | CID:638011 |
| IMPHY011790 | Neral | O=C/C=C(CCC=C(C)C)/C | CID:643779 |
| IMPHY011792 | gamma-Muurolene | CC1=C[C@@H]2[C@H](CC1)C(=C)CC[C@H]2C(C)C | CID:12313020 |
| IMPHY011793 | (+)-gamma-Cadinene | CC1=C[C@@H]2[C@@H](CC1)C(=C)CC[C@H]2C(C)C | CID:6432404 |
| IMPHY011811 | (Z)-Methyl cinnamate | COC(=O)/C=Cc1ccccc1 | CID:6428458 |
| IMPHY011817 | alpha-Farnesene | C=C/C(=C/C/C=C(/CCC=C(C)C)C)/C | CID:5281516 |
| IMPHY011826 | Oleanolic acid | O[C@H]1CC[C@]2([C@H](C1(C)C)CC[C@@]1([C@@H]2CC=C2[C@@]1(C)CC[C@@]1([C@H]2CC(C)(C)CC1)C(=O)O)C)C | CID:10494 |
| IMPHY011880 | Ursolic acid | C[C@@H]1CC[C@]2([C@@H]([C@H]1C)C1=CC[C@H]3[C@@]([C@@]1(CC2)C)(C)CC[C@@H]1[C@]3(C)CC[C@@H](C1(C)C)O)C(=O)O | CID:64945 |
| IMPHY011890 | Elemol | C=C[C@]1(C)CC[C@H](C[C@H]1C(=C)C)C(O)(C)C | CID:92138 |
| IMPHY011894 | (-)-7-Epi-alpha-selinene | CC1=CCC[C@]2([C@H]1C[C@H](CC2)C(=C)C)C | CID:10726905 |
| IMPHY011938 | gamma-Eudesmol | CC1=C2C[C@@H](CC[C@]2(CCC1)C)C(O)(C)C | CID:6432005 |
| IMPHY011939 | 10-epi-gamma-Eudesmol | CC1=C2C[C@@H](CC[C@@]2(CCC1)C)C(O)(C)C | CID:6430754 |
| IMPHY011957 | (+)-delta-Cadinene | CC1=C[C@@H]2C(=C(C)CC[C@H]2C(C)C)CC1 | CID:441005 |
| IMPHY011965 | (+)-beta-Phellandrene | CC([C@@H]1CCC(=C)C=C1)C | CID:442484 |
| IMPHY011973 | (-)-cis-Carveol | CC(=C)[C@@H]1CC=C([C@@H](C1)O)C | CID:330573 |
| IMPHY011988 | (-)-trans-Carveol | CC(=C)[C@@H]1CC=C([C@H](C1)O)C | CID:94221 |
| IMPHY012003 | Betulinic acid | CC(=C)[C@@H]1CC[C@]2([C@H]1[C@H]1CC[C@H]3[C@@]([C@]1(C)CC2)(C)CC[C@@H]1[C@]3(C)CC[C@@H](C1(C)C)O)C(=O)O | CID:64971 |
| IMPHY012036 | Camphor | O=C1CC2C(C1(C)CC2)(C)C | CID:2537 |
| IMPHY012058 | Linalool | C=CC(CCC=C(C)C)(O)C | CID:6549 |
| IMPHY012061 | alpha-Pinene | CC1=CCC2CC1C2(C)C | CID:6654 |
| IMPHY012075 | Carvone | CC(=C)C1CC=C(C(=O)C1)C | CID:7439 |
| IMPHY012086 | Citronellal | O=CCC(CCC=C(C)C)C | CID:7794 |
| IMPHY012104 | Citronellol | OCCC(CCC=C(C)C)C | CID:8842 |
| IMPHY012147 | beta-Pinene | C=C1CCC2CC1C2(C)C | CID:14896 |
| IMPHY012160 | alpha-Terpineol | CC1=CCC(CC1)C(O)(C)C | CID:17100 |
| IMPHY012165 | Sabinene | C=C1CCC2(C1C2)C(C)C | CID:18818 |
| IMPHY012261 | alpha-Bergamotene | CC(=CCCC1(C)C2CC=C(C1C2)C)C | CID:86608 |
| IMPHY012279 | alpha-Curcumene | CC(=CCCC(c1ccc(cc1)C)C)C | CID:92139 |
| IMPHY012586 | (-)-alpha-Cadinol | CC1=CC2C(CC1)[C@@](C)(O)CC[C@@H]2C(C)C | CID:6431302 |
| IMPHY012587 | tau-Cadinol | CC1=C[C@H]2[C@H](CC1)[C@](C)(O)CC[C@H]2C(C)C | CID:12302222 |
| IMPHY012589 | 3-(1,5-Dimethyl-4-hexenyl)-6-methylene-1-cyclohexene | CC(C1CCC(=C)C=C1)CCC=C(C)C | CID:519764 |
| IMPHY012654 | Nerol | OC/C=C(CCC=C(C)C)/C | CID:643820 |
| IMPHY012667 | Caryophyllene oxide | C=C1CC[C@H]2O[C@@]2(CC[C@@H]2[C@@H]1CC2(C)C)C | CID:1742210 |
| IMPHY012739 | (Z)-beta-Ocimene | C=C/C(=CCC=C(C)C)/C | CID:5320250 |
| IMPHY012815 | Fokienol | C=CC(CC/C=C(/CCC/C=C/C(=C)C)C)(O)C | CID:5352449 |
| IMPHY012920 | 2-Furanmethanol, 5-ethenyltetrahydro-alpha,alpha,5-trimethyl-, cis- | C=C[C@@]1(C)CC[C@H](O1)C(O)(C)C | CID:11116492 |
| IMPHY013093 | delta-Elemene | C=C[C@@]1(C)CCC(=C[C@@H]1C(=C)C)C(C)C | CID:12309449 |
| IMPHY013133 | (Z)-p-Menth-2-en-1-ol | CC([C@@H]1CC[C@](C=C1)(C)O)C | CID:13918681 |
| IMPHY013836 | Fenchone | O=C1C2(C)CCC(C1(C)C)C2 | CID:14525 |
| IMPHY013972 | 1,10-Di-epcubenol | CC1=C[C@@H]2[C@](CC1)(O)[C@H](C)CC[C@@H]2C(C)C | CID:91748749 |
| IMPHY014690 | (-)-Globulol | C[C@@H]1CC[C@@H]2[C@@H]1[C@H]1[C@H](C1(C)C)CC[C@@]2(C)O | CID:12304985 |
| IMPHY014708 | beta-Selinene | C=C1CCC[C@]2([C@H]1C[C@@H](CC2)C(=C)C)C | CID:442393 |
| IMPHY014806 | Caswell No. 264AB | CC([C@@H]1CC[C@H]([C@]23[C@H]1[C@H]2C(=CC3)C)C)C | CID:442359 |
| IMPHY014811 | alpha-Phellandrene | CC1=CCC(C=C1)C(C)C | CID:7460 |
| IMPHY014831 | beta-Caryophyllene | C/C/1=CCCC(=C)[C@@H]2[C@@H](CC1)C(C2)(C)C | CID:5281515 |
| IMPHY014835 | (E)-beta-ocimene | C=C/C(=C/CC=C(C)C)/C | CID:5281553 |
| IMPHY014836 | beta-Sitosterol | CC[C@@H](C(C)C)CC[C@H]([C@H]1CC[C@@H]2[C@]1(C)CC[C@H]1[C@H]2CC=C2[C@]1(C)CC[C@@H](C2)O)C | CID:222284 |
| IMPHY014847 | Bornyl acetate | CC(=O)OC1CC2C(C1(C)CC2)(C)C | CID:6448 |
| IMPHY014852 | Camphene | C=C1C2CCC(C1(C)C)C2 | CID:6616 |
| IMPHY014863 | cis-alpha-Bergamotene | CC(=CCCC1(C)[C@@H]2CC=C([C@H]1C2)C)C | CID:91753502 |
| IMPHY014874 | cis-Sabinene hydrate | C[C@@H]1CC[C@@]2(C1C2)C(C)C | CID:101629835 |
| IMPHY014877 | (S)-cis-Verbenol | CC1=C[C@H](O)[C@H]2C[C@@H]1C2(C)C | CID:87839 |
| IMPHY014906 | Cedrelanol | CC1=C[C@@H]2[C@@H](CC1)[C@@](C)(O)CC[C@H]2C(C)C | CID:160799 |
| IMPHY014907 | 6-Epi-beta-bisabolol | CC(=CCC[C@@H]([C@@]1(O)CCC(=CC1)C)C)C | CID:12300148 |
| IMPHY014923 | Geraniol | OC/C=C(/CCC=C(C)C)C | CID:637566 |
| IMPHY014988 | Limonene | CC1=CCC(CC1)C(=C)C | CID:22311 |
| IMPHY015004 | Menthone | C[C@@H]1CC[C@H](C(=O)C1)C(C)C | CID:26447 |
| IMPHY015016 | alpha-Muurolol | CC1=C[C@@H]2[C@H](CC1)[C@](C)(O)CC[C@@H]2C(C)C | CID:91753440 |
| IMPHY015022 | Nerolidol | C=CC(CC/C=C(/CCC=C(C)C)C)(O)C | CID:5284507 |
| IMPHY015095 | 2-Cyclohexen-1-ol, 1-methyl-4-(1-methylethyl)-, trans- | CC([C@@H]1CC[C@@](C=C1)(C)O)C | CID:122484 |
| IMPHY015098 | trans-Verbenol | CC1=C[C@H](O)[C@@H]2C[C@H]1C2(C)C | CID:89664 |
| IMPHY015123 | alpha-Copaene | CC([C@@H]1CC[C@]2([C@@H]3[C@H]1C2C(=CC3)C)C)C | CID:70678558, |
| IMPHY015128 | T-Muurolol | CC1=C[C@@H]2[C@H](CC1)[C@@](C)(O)CC[C@H]2C(C)C | CID:3084331 |
| IMPHY016012 | Allo-Aromadendrene | C[C@@H]1CC[C@H]2[C@@H]1C1C(C1(C)C)CCC2=C | CID:42608158 |
| IMPHY016027 | trans-Sabinene hydrate | CC([C@@]12CC[C@](C2C1)(C)O)C | CID:12315151 |
| IMPHY016046 | trans-4-Thujanol | CC([C@@]12CCC([C@@H]2C1)(C)O)C | CID:12315149 |
| IMPHY016054 | trans-alpha-Bergamotene | CC(=CCC[C@]1(C)[C@H]2CC=C([C@@H]1C2)C)C | CID:6429302 |
| IMPHY016502 | Aciphyllene | CC(=C)C1CCC(C2C(=C(C)CC2)C1)C | CID:565709 |
| IMPHY017693 | Menthyl chavicol | C=CCc1ccc(c(c1)C1CC(C)CCC1C(C)C)O | CID:70235324 |
| IMPHY003536 | Eugenol | C=CCc1ccc(c(c1)OC)O | CID:3314 |
| IMPHY011070 | 1-Octenyl acetate | CCCCCC/C=C/OC(=O)C | CID:5352540 |
| IMPHY011714 | Methyl cinnamate | COC(=O)/C=C/c1ccccc1 | CID:637520 |
| IMPHY011811 | (Z)-Methyl cinnamate | COC(=O)/C=Cc1ccccc1 | CID:6428458 |
| IMPHY012058 | Linalool | C=CC(CCC=C(C)C)(O)C | CID:6549 |
| IMPHY012261 | alpha-Bergamotene | CC(=CCCC1(C)C2CC=C(C1C2)C)C | CID:86608 |
| IMPHY012667 | Caryophyllene oxide | C=C1CC[C@H]2O[C@@]2(CC[C@@H]2[C@@H]1CC2(C)C)C | CID:1742210 |
| IMPHY014831 | beta-Caryophyllene | C/C/1=CCCC(=C)[C@@H]2[C@@H](CC1)C(C2)(C)C | CID:5281515 |
| IMPHY000060 | Myristic acid | CCCCCCCCCCCCCC(=O)O | CID:11005 |
| IMPHY003016 | Lauric acid | CCCCCCCCCCCC(=O)O | CID:3893 |
| IMPHY003104 | Decanoic acid | CCCCCCCCCC(=O)O | CID:2969 |
| IMPHY004187 | L-(+)-Arabinose | OC[C@@H]([C@@H]([C@H](C=O)O)O)O | CID:5460291 |
| IMPHY004631 | Stearic acid | CCCCCCCCCCCCCCCCCC(=O)O | CID:5281 |
| IMPHY007327 | Palmitic acid | CCCCCCCCCCCCCCCC(=O)O | CID:985 |
| IMPHY011394 | Arachidic acid | CCCCCCCCCCCCCCCCCCCC(=O)O | CID:10467 |
| IMPHY011797 | Oleic acid | CCCCCCCC/C=CCCCCCCCC(=O)O | CID:445639 |
| IMPHY012050 | D-Galactose | OC[C@H]1OC(O)[C@@H]([C@H]([C@H]1O)O)O | CID:6036 |
| IMPHY014893 | D-Glucose | OC[C@H]1OC(O)[C@@H]([C@H]([C@@H]1O)O)O | CID:5793 |
| IMPHY014919 | D-Galacturonic Acid | OC1O[C@H](C(=O)O)[C@@H]([C@@H]([C@H]1O)O)O | CID:439215 |
| IMPHY014990 | Linoleic acid | CCCCC/C=CC/C=CCCCCCCCC(=O)O | CID:5280450 |
| IMPHY015056 | L-Rhamnose | O[C@H]1[C@H](C)OC([C@@H]([C@@H]1O)O)O | CID:25310 |
| IMPHY015116 | D-Xylose | O[C@@H]1COC([C@@H]([C@H]1O)O)O | CID:135191 |
| IMPHY007331 | 6-Methyl-5-hepten-2-one | CC(=O)CCC=C(C)C | CID:9862 |
| IMPHY011789 | Citral | O=C/C=C(/CCC=C(C)C)C | CID:638011 |
| IMPHY012058 | Linalool | C=CC(CCC=C(C)C)(O)C | CID:6549 |
| IMPHY012086 | Citronellal | O=CCC(CCC=C(C)C)C | CID:7794 |
| IMPHY012104 | Citronellol | OCCC(CCC=C(C)C)C | CID:8842 |
| IMPHY014923 | Geraniol | OC/C=C(/CCC=C(C)C)C | CID:637566 |
| IMPHY000399 | beta-Bisabolene | CC(=CCCC(=C)[C@H]1CCC(=CC1)C)C | CID:10104370 |
| IMPHY001246 | Carvacrol | CC(c1ccc(c(c1)O)C)C | CID:10364 |
| IMPHY001828 | 3-Octanol | CCCCCC(CC)O | CID:11527 |
| IMPHY002551 | Ishwarane | CC1CCCC23C1(C)CC1C(C2)C1(C3)C | CID:14619932 |
| IMPHY002913 | Sesquisabinene | CC(=CCCC(C12CCC(=C)C2C1)C)C | CID:25202482 |
| IMPHY003398 | Myristicin | C=CCc1cc(OC)c2c(c1)OCO2 | CID:4276 |
| IMPHY003485 | Myrcene | C=CC(=C)CCC=C(C)C | CID:31253 |
| IMPHY003536 | Eugenol | C=CCc1ccc(c(c1)OC)O | CID:3315 |
| IMPHY003616 | Bicyclogermacrene | C/C/1=CCC/C(=C/[C@H]2[C@@H](CC1)C2(C)C)/C | CID:13894537 |
| IMPHY003719 | beta-Copaene | CC([C@@H]1CC[C@]2([C@@H]3[C@H]1C2C(=C)CC3)C)C | CID:57339298 |
| IMPHY003798 | (Z)-alpha-Bisabolene | CC(=CC/C=C(C1CCC(=CC1)C)/C)C | CID:5352653 |
| IMPHY003977 | (-)-beta-Bourbonene | CC([C@@H]1CC[C@@]2([C@H]1[C@H]1C(=C)CC[C@@H]21)C)C | CID:62566 |
| IMPHY003982 | gamma-Terpinene | CC1=CCC(=CC1)C(C)C | CID:7461 |
| IMPHY004077 | Verbenone | CC1=CC(=O)C2CC1C2(C)C | CID:29025 |
| IMPHY004281 | Guaiol | C[C@H]1CC[C@H](CC2=C1CC[C@@H]2C)C(O)(C)C | CID:227829 |
| IMPHY004549 | Safrole | C=CCc1ccc2c(c1)OCO2 | CID:5144 |
| IMPHY005345 | 1-Octen-3-OL | CCCCCC(C=C)O | CID:18827 |
| IMPHY005618 | Germacrene B | C/C/1=CCC/C(=C/CC(=C(C)C)CC1)/C | CID:5281519 |
| IMPHY006145 | p-Cymene | Cc1ccc(cc1)C(C)C | CID:7463 |
| IMPHY006550 | Thymol | Cc1ccc(c(c1)O)C(C)C | CID:6989 |
| IMPHY006696 | Methyleugenol | C=CCc1ccc(c(c1)OC)OC | CID:7127 |
| IMPHY006944 | Estragole | COc1ccc(cc1)CC=C | CID:8815 |
| IMPHY007331 | 6-Methyl-5-hepten-2-one | CC(=O)CCC=C(C)C | CID:9862 |
| IMPHY007376 | beta-Cubebene | CC([C@@H]1CC[C@H]([C@]23[C@H]1[C@H]2C(=C)CC3)C)C | CID:93081 |
| IMPHY007840 | Spathulenol | C=C1CC[C@@H]2[C@H]([C@H]3[C@H]1CC[C@]3(C)O)C2(C)C | CID:92231 |
| IMPHY008936 | alpha-Guaiene | CC(=C)[C@@H]1CC[C@@H](C2=C(C1)[C@@H](C)CC2)C | CID:5317844 |
| IMPHY008946 | delta-Guaiene | CC(=C)[C@@H]1CCC(=C2[C@@H](C1)[C@@H](C)CC2)C | CID:94275 |
| IMPHY009718 | Bulnesol | CC1CCC2=C(C)CCC(CC12)C(O)(C)C | CID:90785 |
| IMPHY009743 | beta-Gurjunene | C[C@@H]1CC[C@@H]2[C@H]([C@H]3[C@@H]1CCC3=C)C2(C)C | CID:6450812 |
| IMPHY010072 | Eucalyptol | CC12CCC(CC1)C(O2)(C)C | CID:2758 |
| IMPHY010080 | beta-Elemene | C=C[C@]1(C)CC[C@H](C[C@H]1C(=C)C)C(=C)C | CID:6918391 |
| IMPHY011392 | 3-Carene | CC1=CCC2C(C1)C2(C)C | CID:26049 |
| IMPHY011396 | 4-Carvomenthenol | CC1=CCC(CC1)(O)C(C)C | CID:11230 |
| IMPHY011455 | Cadina-1,4-diene | CC1=CC2C(=CC1)[C@@H](C)CCC2C(C)C | CID:6427091 |
| IMPHY011484 | (+)-delta-Selinene | CC1=C2C=C(CC[C@]2(CCC1)C)C(C)C | CID:12308846 |
| IMPHY011542 | beta-Eudesmol | C=C1CCC[C@]2([C@H]1C[C@@H](CC2)C(O)(C)C)C | CID:91457 |
| IMPHY011552 | (1R)-2-methyl-5-propan-2-ylbicyclo[3.1.0]hex-2-ene | CC1=CCC2([C@@H]1C2)C(C)C | CID:6451618 |
| IMPHY011581 | alpha-Selinene | CC1=CCC[C@]2([C@H]1C[C@@H](CC2)C(=C)C)C | CID:10856614 |
| IMPHY011586 | (S,1Z,6Z)-8-Isopropyl-1-methyl-5-methylenecyclodeca-1,6-diene | C/C/1=C/CCC(=C)/C=C[C@@H](CC1)C(C)C | CID:91723653 |
| IMPHY011590 | d-Borneol | O[C@@H]1C[C@H]2C([C@@]1(C)CC2)(C)C | CID:61060 |
| IMPHY011599 | Terpinolene | CC1=CCC(=C(C)C)CC1 | CID:11463 |
| IMPHY011643 | alpha-Terpinene | CC1=CC=C(CC1)C(C)C | CID:7462 |
| IMPHY011647 | Geranyl acetate | C/C(=CCOC(=O)C)/CCC=C(C)C | CID:1549026 |
| IMPHY011648 | Neryl acetate | C/C(=C/COC(=O)C)/CCC=C(C)C | CID:1549025 |
| IMPHY011658 | beta-Farnesene | C=CC(=C)CC/C=C(/CCC=C(C)C)C | CID:5281517 |
| IMPHY011667 | alpha-Gurjunene | C[C@@H]1CC[C@@H]2[C@H](C3=C(CC[C@H]13)C)C2(C)C | CID:15560276 |
| IMPHY011709 | alpha-Eudesmol | CC1=CCC[C@]2([C@H]1C[C@@H](CC2)C(O)(C)C)C | CID:92762 |
| IMPHY011714 | Methyl cinnamate | COC(=O)/C=C/c1ccccc1 | CID:637520 |
| IMPHY011745 | Zingiberene | CC(=CCC[C@@H]([C@H]1CC=C(C=C1)C)C)C | CID:92776 |
| IMPHY011749 | Humulene epoxide II | C/C/1=CCC(C)(C)/C=C/C[C@@]2([C@@H](CC1)O2)C | CID:10704181 |
| IMPHY011761 | Humulene | C/C/1=CCC(C)(C)/C=C/C/C(=C/CC1)/C | CID:5281520 |
| IMPHY011789 | Citral | O=C/C=C(/CCC=C(C)C)C | CID:638011 |
| IMPHY011790 | Neral | O=C/C=C(CCC=C(C)C)/C | CID:643779 |
| IMPHY011792 | gamma-Muurolene | CC1=C[C@@H]2[C@H](CC1)C(=C)CC[C@H]2C(C)C | CID:12313020 |
| IMPHY011793 | (+)-gamma-Cadinene | CC1=C[C@@H]2[C@@H](CC1)C(=C)CC[C@H]2C(C)C | CID:6432404 |
| IMPHY011811 | (Z)-Methyl cinnamate | COC(=O)/C=Cc1ccccc1 | CID:6428458 |
| IMPHY011817 | alpha-Farnesene | C=C/C(=C/C/C=C(/CCC=C(C)C)C)/C | CID:5281516 |
| IMPHY011839 | (Z)-gamma-bisabolene | CC(=CCC/C(=C1/CCC(=CC1)C)/C)C | CID:3033866 |
| IMPHY011873 | Humulene epoxide | C/C/1=CCCC2(C)OC2CC(/C=C/C1)(C)C | CID:5352470 |
| IMPHY011890 | Elemol | C=C[C@]1(C)CC[C@H](C[C@H]1C(=C)C)C(O)(C)C | CID:92138 |
| IMPHY011896 | Valencene | CC(=C)[C@@H]1CCC2=CCC[C@H]([C@@]2(C1)C)C | CID:9855795 |
| IMPHY011938 | gamma-Eudesmol | CC1=C2C[C@@H](CC[C@]2(CCC1)C)C(O)(C)C | CID:6432005 |
| IMPHY011957 | (+)-delta-Cadinene | CC1=C[C@@H]2C(=C(C)CC[C@H]2C(C)C)CC1 | CID:441005 |
| IMPHY011965 | (+)-beta-Phellandrene | CC([C@@H]1CCC(=C)C=C1)C | CID:442484 |
| IMPHY011988 | (-)-trans-Carveol | CC(=C)[C@@H]1CC=C([C@H](C1)O)C | CID:94221 |
| IMPHY012036 | Camphor | O=C1CC2C(C1(C)CC2)(C)C | CID:2537 |
| IMPHY012058 | Linalool | C=CC(CCC=C(C)C)(O)C | CID:6549 |
| IMPHY012061 | alpha-Pinene | CC1=CCC2CC1C2(C)C | CID:6654 |
| IMPHY012075 | Carvone | CC(=C)C1CC=C(C(=O)C1)C | CID:7439 |
| IMPHY012147 | beta-Pinene | C=C1CCC2CC1C2(C)C | CID:14896 |
| IMPHY012160 | alpha-Terpineol | CC1=CCC(CC1)C(O)(C)C | CID:17100 |
| IMPHY012165 | Sabinene | C=C1CCC2(C1C2)C(C)C | CID:18818 |
| IMPHY012279 | alpha-Curcumene | CC(=CCCC(c1ccc(cc1)C)C)C | CID:92139 |
| IMPHY012585 | delta-Cadinol | CC1=C[C@@H]2[C@H](CC1)[C@](C)(O)CC[C@H]2C(C)C | CID:3084311 |
| IMPHY012586 | (-)-alpha-Cadinol | CC1=CC2C(CC1)[C@@](C)(O)CC[C@@H]2C(C)C | CID:6431302 |
| IMPHY012654 | Nerol | OC/C=C(CCC=C(C)C)/C | CID:643820 |
| IMPHY012665 | Levomenol | CC(=CCC[C@@]([C@H]1CCC(=CC1)C)(O)C)C | CID:442343 |
| IMPHY012667 | Caryophyllene oxide | C=C1CC[C@H]2O[C@@]2(CC[C@@H]2[C@@H]1CC2(C)C)C | CID:1742210 |
| IMPHY012739 | (Z)-beta-Ocimene | C=C/C(=CCC=C(C)C)/C | CID:5320250 |
| IMPHY012920 | 2-Furanmethanol, 5-ethenyltetrahydro-alpha,alpha,5-trimethyl-, cis- | C=C[C@@]1(C)CC[C@H](O1)C(O)(C)C | CID:11116492 |
| IMPHY013093 | delta-Elemene | C=C[C@@]1(C)CCC(=C[C@@H]1C(=C)C)C(C)C | CID:12309449 |
| IMPHY013836 | Fenchone | O=C1C2(C)CCC(C1(C)C)C2 | CID:14525 |
| IMPHY014708 | beta-Selinene | C=C1CCC[C@]2([C@H]1C[C@@H](CC2)C(=C)C)C | CID:442393 |
| IMPHY014801 | Zizanene | CC1=C[C@@H]2[C@H](CC1)C(=CC[C@@H]2C(C)C)C | CID:12306046 |
| IMPHY014806 | Caswell No. 264AB | CC([C@@H]1CC[C@H]([C@]23[C@H]1[C@H]2C(=CC3)C)C)C | CID:442359 |
| IMPHY014811 | alpha-Phellandrene | CC1=CCC(C=C1)C(C)C | CID:7460 |
| IMPHY014817 | Aromadendrene | CC1CCC2C1C1C(C1(C)C)CCC2=C | CID:91354 |
| IMPHY014831 | beta-Caryophyllene | C/C/1=CCCC(=C)[C@@H]2[C@@H](CC1)C(C2)(C)C | CID:5281515 |
| IMPHY014835 | (E)-beta-ocimene | C=C/C(=C/CC=C(C)C)/C | CID:5281553 |
| IMPHY014847 | Bornyl acetate | CC(=O)OC1CC2C(C1(C)CC2)(C)C | CID:6448 |
| IMPHY014852 | Camphene | C=C1C2CCC(C1(C)C)C2 | CID:6616 |
| IMPHY014885 | 1-Isopropyl-4,7-dimethyl-1,3,4,5,6,8a-hexahydro-4a(2H)-naphthalenol | CC1=CC2C(CC1)(O)C(C)CCC2C(C)C | CID:519857 |
| IMPHY014906 | Cedrelanol | CC1=C[C@@H]2[C@@H](CC1)[C@@](C)(O)CC[C@H]2C(C)C | CID:160799 |
| IMPHY014907 | 6-Epi-beta-bisabolol | CC(=CCC[C@@H]([C@@]1(O)CCC(=CC1)C)C)C | CID:12300148 |
| IMPHY014914 | Fenchol | OC1C2(C)CCC(C1(C)C)C2 | CID:15406 |
| IMPHY014923 | Geraniol | OC/C=C(/CCC=C(C)C)C | CID:637566 |
| IMPHY014988 | Limonene | CC1=CCC(CC1)C(=C)C | CID:22311 |
| IMPHY014989 | trans-Linalool oxide | C=C[C@]1(C)CC[C@H](O1)C(O)(C)C | CID:6432254 |
| IMPHY015016 | alpha-Muurolol | CC1=C[C@@H]2[C@H](CC1)[C@](C)(O)CC[C@@H]2C(C)C | CID:91753440 |
| IMPHY015022 | Nerolidol | C=CC(CC/C=C(/CCC=C(C)C)C)(O)C | CID:5284507 |
| IMPHY015123 | alpha-Copaene | CC([C@@H]1CC[C@]2([C@@H]3[C@H]1C2C(=CC3)C)C)C | CID:70678558 |
| IMPHY015128 | T-Muurolol | CC1=C[C@@H]2[C@H](CC1)[C@@](C)(O)CC[C@H]2C(C)C | CID:3084331 |
| IMPHY016012 | Allo-Aromadendrene | C[C@@H]1CC[C@H]2[C@@H]1C1C(C1(C)C)CCC2=C | CID:42608158 |
| IMPHY016054 | trans-alpha-Bergamotene | CC(=CCC[C@]1(C)[C@H]2CC=C([C@@H]1C2)C)C | CID:6429302 |
| IMPHY000099 | Myrtenol | OCC1=CCC2CC1C2(C)C | CID:10582 |
| IMPHY000399 | beta-Bisabolene | CC(=CCCC(=C)[C@H]1CCC(=CC1)C)C | CID:10104370 |
| IMPHY001776 | Nevadensin | COc1ccc(cc1)c1cc(=O)c2c(o1)c(OC)c(c(c2O)OC)O | CID:160921 |
| IMPHY003485 | Myrcene | C=CC(=C)CCC=C(C)C | CID:31253 |
| IMPHY003536 | Eugenol | C=CCc1ccc(c(c1)OC)O | CID:3314 |
| IMPHY003616 | Bicyclogermacrene | C/C/1=CCC/C(=C/[C@H]2[C@@H](CC1)C2(C)C)/C | CID:13894537 |
| IMPHY003977 | (-)-beta-Bourbonene | CC([C@@H]1CC[C@@]2([C@H]1[C@H]1C(=C)CC[C@@H]21)C)C | CID:62566 |
| IMPHY003982 | gamma-Terpinene | CC1=CCC(=CC1)C(C)C | CID:7461 |
| IMPHY004187 | L-(+)-Arabinose | OC[C@@H]([C@@H]([C@H](C=O)O)O)O | CID:5460291 |
| IMPHY004235 | D-Glucuronic Acid | OC1O[C@H](C(=O)O)[C@H]([C@@H]([C@H]1O)O)O | CID:94715 |
| IMPHY004372 | Selina-4(15),7(11)-diene | C=C1CCC[C@]2([C@H]1CC(=C(C)C)CC2)C | CID:10655819 |
| IMPHY004388 | Kaempferol | Oc1ccc(cc1)c1oc2cc(O)cc(c2c(=O)c1O)O | CID:5280863 |
| IMPHY004543 | gamma-Patchoulene | CC1CCC23C1CC(C3(C)C)CCC2=C | CID:521302 |
| IMPHY004661 | Apigenin | Oc1ccc(cc1)c1cc(=O)c2c(o1)cc(cc2O)O | CID:5280443 |
| IMPHY006138 | Anisole | COc1ccccc1 | CID:7519 |
| IMPHY006145 | p-Cymene | Cc1ccc(cc1)C(C)C | CID:7463 |
| IMPHY006362 | Ascorbic acid | OC[C@@H]([C@H]1OC(=O)C(=C1O)O)O | CID:54670067 |
| IMPHY006550 | Thymol | Cc1ccc(c(c1)O)C(C)C | CID:6989 |
| IMPHY006696 | Methyleugenol | C=CCc1ccc(c(c1)OC)OC | CID:7127 |
| IMPHY006709 | Acetyleugenol | C=CCc1ccc(c(c1)OC)OC(=O)C | CID:7136 |
| IMPHY006944 | Estragole | COc1ccc(cc1)CC=C | CID:8815 |
| IMPHY007214 | Octyl acetate | CCCCCCCCOC(=O)C | CID:8164 |
| IMPHY007267 | beta-Terpinene | C=C1CCC(=CC1)C(C)C | CID:66841 |
| IMPHY007331 | 6-Methyl-5-hepten-2-one | CC(=O)CCC=C(C)C | CID:9862 |
| IMPHY007840 | Spathulenol | C=C1CC[C@@H]2[C@H]([C@H]3[C@H]1CC[C@]3(C)O)C2(C)C | CID:92231 |
| IMPHY007925 | Ellipticine | Cc1c2cnccc2c(c2c1c1ccccc1[nH]2)C | CID:3213 |
| IMPHY008092 | Epoxytagetone | C=CC1(C)OC1C(=O)CC(C)C | CID:91748342 |
| IMPHY008296 | Gratissimene | CC(C/C=C/C(C1=CCC(CC1)C)C)C | CID:101316466 |
| IMPHY008458 | (1E,6Z)-gamma-humulene | C=C1CC/C=C(C)/CCCC(/C=C1)(C)C | CID:24798697 |
| IMPHY009047 | Oleuropeic acid | OC(=O)C1=CC[C@H](CC1)C(O)(C)C | CID:188320 |
| IMPHY010072 | Eucalyptol | CC12CCC(CC1)C(O2)(C)C | CID:2758 |
| IMPHY011070 | 1-Octenyl acetate | CCCCCC/C=C/OC(=O)C | CID:5352540 |
| IMPHY011392 | 3-Carene | CC1=CCC2C(C1)C2(C)C | CID:26049 |
| IMPHY011396 | 4-Carvomenthenol | CC1=CCC(CC1)(O)C(C)C | CID:11230 |
| IMPHY011521 | 2-Undecanone | CCCCCCCCCC(=O)C | CID:8163 |
| IMPHY011579 | Eremophilene | CC(=C)[C@@H]1CCC2=CCC[C@@H]([C@]2(C1)C)C | CID:12309744 |
| IMPHY011586 | (S,1Z,6Z)-8-Isopropyl-1-methyl-5-methylenecyclodeca-1,6-diene | C/C/1=C/CCC(=C)/C=C[C@@H](CC1)C(C)C | CID:91723653 |
| IMPHY011590 | d-Borneol | O[C@@H]1C[C@H]2C([C@@]1(C)CC2)(C)C | CID:61060 |
| IMPHY011599 | Terpinolene | CC1=CCC(=C(C)C)CC1 | CID:11463 |
| IMPHY011640 | Isoeugenol | C/C=C/c1ccc(c(c1)OC)O | CID:853433 |
| IMPHY011658 | beta-Farnesene | C=CC(=C)CC/C=C(/CCC=C(C)C)C | CID:5281517 |
| IMPHY011714 | Methyl cinnamate | COC(=O)/C=C/c1ccccc1 | CID:637520 |
| IMPHY011761 | Humulene | C/C/1=CCC(C)(C)/C=C/C/C(=C/CC1)/C | CID:5281520 |
| IMPHY011789 | Citral | O=C/C=C(/CCC=C(C)C)C | CID:638011 |
| IMPHY011811 | (Z)-Methyl cinnamate | COC(=O)/C=Cc1ccccc1 | CID:6428458 |
| IMPHY011826 | Oleanolic acid | O[C@H]1CC[C@]2([C@H](C1(C)C)CC[C@@]1([C@@H]2CC=C2[C@@]1(C)CC[C@@]1([C@H]2CC(C)(C)CC1)C(=O)O)C)C | CID:10494 |
| IMPHY011880 | Ursolic acid | C[C@@H]1CC[C@]2([C@@H]([C@H]1C)C1=CC[C@H]3[C@@]([C@@]1(CC2)C)(C)CC[C@@H]1[C@]3(C)CC[C@@H](C1(C)C)O)C(=O)O | CID:64945 |
| IMPHY011919 | Afzelin | Oc1ccc(cc1)c1oc2cc(O)cc(c2c(=O)c1O[C@@H]1O[C@@H](C)[C@@H]([C@H]([C@H]1O)O)O)O | CID:5316673 |
| IMPHY011957 | (+)-delta-Cadinene | CC1=C[C@@H]2C(=C(C)CC[C@H]2C(C)C)CC1 | CID:441005 |
| IMPHY012003 | Betulinic acid | CC(=C)[C@@H]1CC[C@]2([C@H]1[C@H]1CC[C@H]3[C@@]([C@]1(C)CC2)(C)CC[C@@H]1[C@]3(C)CC[C@@H](C1(C)C)O)C(=O)O | CID:64971 |
| IMPHY012036 | Camphor | O=C1CC2C(C1(C)CC2)(C)C | CID:2537 |
| IMPHY012050 | D-Galactose | OC[C@H]1OC(O)[C@@H]([C@H]([C@H]1O)O)O | CID:6036 |
| IMPHY012058 | Linalool | C=CC(CCC=C(C)C)(O)C | CID:6549 |
| IMPHY012061 | alpha-Pinene | CC1=CCC2CC1C2(C)C | CID:6654 |
| IMPHY012086 | Citronellal | O=CCC(CCC=C(C)C)C | CID:7794 |
| IMPHY012147 | beta-Pinene | C=C1CCC2CC1C2(C)C | CID:14896 |
| IMPHY012160 | alpha-Terpineol | CC1=CCC(CC1)C(O)(C)C | CID:17100 |
| IMPHY012165 | Sabinene | C=C1CCC2(C1C2)C(C)C | CID:18818 |
| IMPHY012205 | Sabinene hydrate | CC(C12CCC(C2C1)(C)O)C | CID:62367 |
| IMPHY012261 | alpha-Bergamotene | CC(=CCCC1(C)C2CC=C(C1C2)C)C | CID:86608 |
| IMPHY012585 | delta-Cadinol | CC1=C[C@@H]2[C@H](CC1)[C@](C)(O)CC[C@H]2C(C)C | CID:3084311 |
| IMPHY012654 | Nerol | OC/C=C(CCC=C(C)C)/C | CID:643820 |
| IMPHY012667 | Caryophyllene oxide | C=C1CC[C@H]2O[C@@]2(CC[C@@H]2[C@@H]1CC2(C)C)C | CID:1742210 |
| IMPHY014708 | beta-Selinene | C=C1CCC[C@]2([C@H]1C[C@@H](CC2)C(=C)C)C | CID:442393 |
| IMPHY014824 | Astragalin | OC[C@H]1O[C@@H](Oc2c(oc3c(c2=O)c(O)cc(c3)O)c2ccc(cc2)O)[C@@H]([C@H]([C@@H]1O)O)O | CID:5282102 |
| IMPHY014830 | Bergamotene | CC(=CCCC1(C)C2CCC(=C)C1C2)C | CID:521569 |
| IMPHY014831 | beta-Caryophyllene | C/C/1=CCCC(=C)[C@@H]2[C@@H](CC1)C(C2)(C)C | CID:5281515 |
| IMPHY014835 | (E)-beta-ocimene | C=C/C(=C/CC=C(C)C)/C | CID:5281553 |
| IMPHY014836 | beta-Sitosterol | CC[C@@H](C(C)C)CC[C@H]([C@H]1CC[C@@H]2[C@]1(C)CC[C@H]1[C@H]2CC=C2[C@]1(C)CC[C@@H](C2)O)C | CID:222284 |
| IMPHY014852 | Camphene | C=C1C2CCC(C1(C)C)C2 | CID:6616 |
| IMPHY014919 | D-Galacturonic Acid | OC1O[C@H](C(=O)O)[C@@H]([C@@H]([C@H]1O)O)O | CID:439215 |
| IMPHY014923 | Geraniol | OC/C=C(/CCC=C(C)C)C | CID:637566 |
| IMPHY014988 | Limonene | CC1=CCC(CC1)C(=C)C | CID:22311 |
| IMPHY015004 | Menthone | C[C@@H]1CC[C@H](C(=O)C1)C(C)C | CID:26447 |
| IMPHY015056 | L-Rhamnose | O[C@H]1[C@H](C)OC([C@@H]([C@@H]1O)O)O | CID:25310 |
| IMPHY015116 | D-Xylose | O[C@@H]1COC([C@@H]([C@H]1O)O)O | CID:135191 |
| IMPHY016012 | Allo-Aromadendrene | C[C@@H]1CC[C@H]2[C@@H]1C1C(C1(C)C)CCC2=C | CID:42608158 |
| IMPHY016054 | trans-alpha-Bergamotene | CC(=CCC[C@]1(C)[C@H]2CC=C([C@@H]1C2)C)C | CID:6429302 |
|  |  |  |  |
|  |  |  |  |
| ***2. Plant name: Ocimum africanum*** | | | |
| **IMPPAT Phytochemical identifier:** | **Phytochemical name:** | **SMILES:** | **CID** |
| IMPHY000399 | beta-Bisabolene | CC(=CCCC(=C)[C@H]1CCC(=CC1)C)C | CID:10104370 |
| IMPHY003956 | (+)-gamma-Gurjunene | C[C@@H]1CC[C@H]2C1=C[C@@H](CC[C@H]2C)C(=C)C | CID:15560285 |
| IMPHY004150 | Neryl formate | O=COC/C=C(CCC=C(C)C)/C | CID:5354882 |
| IMPHY007331 | 6-Methyl-5-hepten-2-one | CC(=O)CCC=C(C)C | CID:9862 |
| IMPHY007376 | beta-Cubebene | CC([C@@H]1CC[C@H]([C@]23[C@H]1[C@H]2C(=C)CC3)C)C | CID:93081 |
| IMPHY007840 | Spathulenol | C=C1CC[C@@H]2[C@H]([C@H]3[C@H]1CC[C@]3(C)O)C2(C)C | CID:92231 |
| IMPHY010072 | Eucalyptol | CC12CCC(CC1)C(O2)(C)C | CID:2758 |
| IMPHY011586 | (S,1Z,6Z)-8-Isopropyl-1-methyl-5-methylenecyclodeca-1,6-diene | C/C/1=C/CCC(=C)/C=C[C@@H](CC1)C(C)C | CID:91723653 |
| IMPHY011647 | Geranyl acetate | C/C(=CCOC(=O)C)/CCC=C(C)C | CID:1549026 |
| IMPHY011648 | Neryl acetate | C/C(=C/COC(=O)C)/CCC=C(C)C | CID:1549025 |
| IMPHY011657 | cis-beta-Farnesene | C=CC(=C)CC/C=C(CCC=C(C)C)/C | CID:5317319 |
| IMPHY011658 | beta-Farnesene | C=CC(=C)CC/C=C(/CCC=C(C)C)C | CID:5281517 |
| IMPHY011761 | Humulene | C/C/1=CCC(C)(C)/C=C/C/C(=C/CC1)/C | CID:5281520 |
| IMPHY011789 | Citral | O=C/C=C(/CCC=C(C)C)C | CID:638011 |
| IMPHY011790 | Neral | O=C/C=C(CCC=C(C)C)/C | CID:643779 |
| IMPHY012058 | Linalool | C=CC(CCC=C(C)C)(O)C | CID:6549 |
| IMPHY012086 | Citronellal | O=CCC(CCC=C(C)C)C | CID:7794 |
| IMPHY012147 | beta-Pinene | C=C1CCC2CC1C2(C)C | CID:14896 |
| IMPHY012654 | Nerol | OC/C=C(CCC=C(C)C)/C | CID:643820 |
| IMPHY012667 | Caryophyllene oxide | C=C1CC[C@H]2O[C@@]2(CC[C@@H]2[C@@H]1CC2(C)C)C | CID:1742210 |
| IMPHY014831 | beta-Caryophyllene | C/C/1=CCCC(=C)[C@@H]2[C@@H](CC1)C(C2)(C)C | CID:5281515 |
| IMPHY014923 | Geraniol | OC/C=C(/CCC=C(C)C)C | CID:637566 |
| IMPHY014988 | Limonene | CC1=CCC(CC1)C(=C)C | CID:22311 |
| IMPHY015123 | alpha-Copaene | CC([C@@H]1CC[C@]2([C@@H]3[C@H]1C2C(=CC3)C)C)C | CID:70678558 |
| IMPHY016054 | trans-alpha-Bergamotene | CC(=CCC[C@]1(C)[C@H]2CC=C([C@@H]1C2)C)C | CID:6429302 |
| IMPHY006696 | Methyleugenol | C=CCc1ccc(c(c1)OC)OC | CID:7127 |
| IMPHY006944 | Estragole | COc1ccc(cc1)CC=C | CID:8815 |
| IMPHY011647 | Geranyl acetate | C/C(=CCOC(=O)C)/CCC=C(C)C | CID:1549026 |
| IMPHY011714 | Methyl cinnamate | COC(=O)/C=C/c1ccccc1 | CID:637520 |
| IMPHY012058 | Linalool | C=CC(CCC=C(C)C)(O)C | CID:6549 |
| IMPHY014923 | Geraniol | OC/C=C(/CCC=C(C)C)C | CID:637566 |
| IMPHY015011 | Methylisoeugenol | C/C=C/c1ccc(c(c1)OC)OC | CID:637776 |
|  |  |  |  |
|  |  |  |  |
| ***3. Plant name: Ocimum carnosum*** | | | |
| **IMPPAT Phytochemical identifier:** | **Phytochemical name:** | **SMILES:** | **CID** |
| IMPHY001351 | Elemicin | C=CCc1cc(OC)c(c(c1)OC)OC | CID:10248 |
| IMPHY003536 | Eugenol | C=CCc1ccc(c(c1)OC)O | CID:3314 |
|  |  |  |  |
|  |  |  |  |
|  |  |  |  |
|  |  |  |  |
|  |  |  |  |
|  |  |  |  |
|  |  |  |  |
| **4. Plant name: Artocarpus altilis** | | | |
| **IMPPAT Phytochemical identifier:** | **Phytochemical name:** | **SMILES:** | **CID** |
| IMPHY000132 | Cycloartomunin | COc1cc2OC(C=C(C)C)c3c(-c2cc1O)oc1c(c3=O)c(O)cc2c1C=CC(O2)(C)C | CID:10456313 |
| IMPHY000674 | Dihydrocycloartomunin | COc1cc(O)c2c(c1CC=C(C)C)oc1-c3cc(O)c(cc3OC(c1c2=O)C=C(C)C)O | CID:10072454 |
| IMPHY002830 | artonin F | CC(=CCc1c2OC(C)(C)C=Cc2c2c(c1O)c(=O)c1c(-c3c(O)cc(c4c3C(C1)C(C)(C)O4)O)o2)C | CID:14680593 |
| IMPHY005903 | Artonin E | CC(=CCc1c(oc2c(c1=O)c(O)cc1c2C=CC(O1)(C)C)c1cc(O)c(cc1O)O)C | CID:5481962 |
| IMPHY011619 | alpha-Amyrin | C[C@@H]1CC[C@]2([C@@H]([C@H]1C)C1=CC[C@H]3[C@@]([C@@]1(CC2)C)(C)CC[C@@H]1[C@]3(C)CC[C@@H](C1(C)C)O)C | [CID:73170](cid:73170) |
| IMPHY011642 | Cycloartenol | CC(=CCC[C@H]([C@H]1CC[C@@]2([C@]1(C)CC[C@@]13[C@H]2CC[C@@H]2[C@]3(C1)CC[C@@H](C2(C)C)O)C)C)C | CID:92110 |
| IMPHY000132 | Cycloartomunin | COc1cc2OC(C=C(C)C)c3c(-c2cc1O)oc1c(c3=O)c(O)cc2c1C=CC(O2)(C)C | CID:10456312 |
| IMPHY000674 | Dihydrocycloartomunin | COc1cc(O)c2c(c1CC=C(C)C)oc1-c3cc(O)c(cc3OC(c1c2=O)C=C(C)C)O | CID:10072454 |
| IMPHY001627 | Artomunoxanthone | COc1cc(O)c2-c3oc4c5C=CC(Oc5cc(c4c(=O)c3CC(c2c1O)C(=C)C)O)(C)C | CID:15725887 |
| IMPHY001629 | Artomunoxanthentrione | COC1=CC(=O)c2c(C1=O)c(cc1c2oc2c3C=CC(Oc3cc(c2c1=O)O)(C)C)C(=C)C | CID:15725888 |
| IMPHY001678 | Cycloartomunoxanthone | COc1cc(O)c2-c3oc4c5C=CC(Oc5cc(c4c(=O)c3CC3c2c1OC3(C)C)O)(C)C | CID:14841185 |
| IMPHY001910 | Cyclomulberrin | CC(=CCc1c(O)cc(c2c1oc1-c3ccc(cc3OC(c1c2=O)C=C(C)C)O)O)C | CID:11742872 |
| IMPHY005875 | Cyclomorusin | CC(=CC1Oc2cc(O)ccc2-c2c1c(=O)c1c(o2)c2C=CC(Oc2cc1O)(C)C)C | CID:5481969 |
| IMPHY007854 | Cyclocommunol | CC(=CC1Oc2cc(O)ccc2-c2c1c(=O)c1c(o2)cc(cc1O)O)C | CID:10315987 |
| IMPHY007897 | Dihydroisocycloartomunin | COc1cc2-c3oc4c(CC=C(C)C)c(O)cc(c4c(=O)c3C(Oc2cc1O)C=C(C)C)O | CID:10095293 |
| IMPHY014836 | beta-Sitosterol | CC[C@@H](C(C)C)CC[C@H]([C@H]1CC[C@@H]2[C@]1(C)CC[C@H]1[C@H]2CC=C2[C@]1(C)CC[C@@H](C2)O)C | CID:222284 |
| IMPHY014991 | Lupeol acetate | CC(=O)O[C@H]1CC[C@]2([C@H](C1(C)C)CC[C@@]1([C@@H]2CC[C@H]2[C@@]1(C)CC[C@@]1([C@@H]2[C@@H](CC1)C(=C)C)C)C)C | CID:92157 |
| IMPHY001910 | Cyclomulberrin | CC(=CCc1c(O)cc(c2c1oc1-c3ccc(cc3OC(c1c2=O)C=C(C)C)O)O)C | CID:11742872 |
| IMPHY003888 | Engeletin | Oc1ccc(cc1)[C@H]1Oc2cc(O)cc(c2C(=O)[C@@H]1O[C@@H]1O[C@@H](C)[C@@H]([C@H]([C@H]1O)O)O)O | CID:6453452 |
| IMPHY004952 | cudraflavone A | CC(=CC1Oc2cc(O)ccc2-c2c1c(=O)c1c(o2)cc2c(c1O)C=CC(O2)(C)C)C | CID:5316261 |
| IMPHY005875 | Cyclomorusin | CC(=CC1Oc2cc(O)ccc2-c2c1c(=O)c1c(o2)c2C=CC(Oc2cc1O)(C)C)C | CID:5481969 |
| IMPHY012782 | Isocyclomulberrin | CC(=CC1Oc2cc(O)ccc2-c2c1c(=O)c1c(o2)cc(c(c1O)CC=C(C)C)O)C | CID:5316260 |
| IMPHY013421 | Cycloaltilisin | COc1cc2OC(C=C(C)C)c3c(-c2cc1O)oc1c(c3=O)c(O)cc(c1CC=C(C)C)O | CID:44258301 |
| IMPHY005463 | Morin | Oc1ccc(c(c1)O)c1oc2cc(O)cc(c2c(=O)c1O)O | CID:5281670 |
| IMPHY005874 | Norartocarpetin | Oc1ccc(c(c1)O)c1cc(=O)c2c(o1)cc(cc2O)O | CID:5481970 |
| IMPHY006382 | 3,8-dihydroxy-10-methoxy-9-[(E)-3-methylbut-1-enyl]-6-(2-methylprop-1-enyl)-6H-chromeno[4,3-b]chromen-7-one | COc1cc2oc3-c4ccc(cc4OC(c3c(=O)c2c(c1/C=C/C(C)C)O)C=C(C)C)O | CID:5458462 |
| IMPHY006383 | Artocarpin | COc1cc2oc(c3ccc(cc3O)O)c(c(=O)c2c(c1/C=C/C(C)C)O)CC=C(C)C | CID:5458461 |
| IMPHY001232 | Cycloartobiloxanthone | Oc1cc(O)c2c3c1-c1oc4c5C=CC(Oc5cc(c4c(=O)c1CC3C(O2)(C)C)O)(C)C | CID:10342859 |
| IMPHY001678 | Cycloartomunoxanthone | COc1cc(O)c2-c3oc4c5C=CC(Oc5cc(c4c(=O)c3CC3c2c1OC3(C)C)O)(C)C | CID:14841185 |
| IMPHY004952 | cudraflavone A | CC(=CC1Oc2cc(O)ccc2-c2c1c(=O)c1c(o2)cc2c(c1O)C=CC(O2)(C)C)C | CID:5316261 |
| IMPHY014141 | 2-Geranyl-2',3,4,4'-tetrahydroxydihydrochalcone | C/C(=CCc1c(CCC(=O)c2ccc(cc2O)O)ccc(c1O)O)/CCC=C(C)C | CID:6449829 |
| IMPHY014836 | beta-Sitosterol | CC[C@@H](C(C)C)CC[C@H]([C@H]1CC[C@@H]2[C@]1(C)CC[C@H]1[C@H]2CC=C2[C@]1(C)CC[C@@H](C2)O)C | CID:222284 |
| IMPHY014991 | Lupeol acetate | CC(=O)O[C@H]1CC[C@]2([C@H](C1(C)C)CC[C@@]1([C@@H]2CC[C@H]2[C@@]1(C)CC[C@@]1([C@@H]2[C@@H](CC1)C(=C)C)C)C)C | CID:92157 |
|  |  |  |  |
|  |  |  |  |
|  |  |  |  |
| ***5. Plant name: Phyllanthus niruri*** | | | |
| **IMPPAT Phytochemical identifier:** | **Phytochemical name:** | **SMILES:** | **CID** |
| IMPHY010965 | Corilagin | O[C@@H]1[C@H]2COC(=O)c3cc(O)c(c(c3-c3c(C(=O)O[C@@H]1[C@H]([C@@H](O2)OC(=O)c1cc(O)c(c(c1)O)O)O)cc(O)c(c3O)O)O)O | CID:73568 |
| IMPHY003390 | Hypophyllanthin | COC[C@@H]1Cc2cc(OC)c3c(c2[C@@H]([C@H]1COC)c1ccc(c(c1)OC)OC)OCO3 | CID:182140 |
| IMPHY003246 | Phyllanthin | COC[C@H]([C@H](Cc1ccc(c(c1)OC)OC)COC)Cc1ccc(c(c1)OC)OC | CID:358901 |
| IMPHY003390 | Hypophyllanthin | COC[C@@H]1Cc2cc(OC)c3c(c2[C@@H]([C@H]1COC)c1ccc(c(c1)OC)OC)OCO3 | CID:182140 |
|  |  |  |  |
|  |  |  |  |
| ***6. Plant name: Azadirachta indica*** | | | |
| **IMPPAT Phytochemical identifier:** | **Phytochemical name:** | **SMILES:** | **CID** |
| IMPHY000093 | Nimbiol | O=C1C[C@H]2C(C)(C)CCC[C@@]2(c2c1cc(C)c(c2)O)C | 11119228 |
| IMPHY000150 | 6-Deacetylnimbin | COC(=O)C[C@H]1[C@@]2(C)[C@H](O[C@H]3C2=C(C)[C@@H](C3)c2cocc2)[C@@H]([C@@H]2[C@]1(C)C(=O)C=C[C@@]2(C)C(=O)OC)O | 10505484 |
| IMPHY001446 | Kulinone | CC(=CCC[C@H]([C@@H]1[C@@H](O)C[C@]2([C@@]1(C)CC[C@H]1C2=CC[C@@H]2[C@]1(C)CCC(=O)C2(C)C)C)C)C | 44567124 |
| IMPHY001448 | Methyl kulonate | COC(=O)[C@@H]([C@@H]1[C@@H](O)C[C@]2([C@@]1(C)CC[C@H]1C2=CC[C@@H]2[C@]1(C)CCC(=O)C2(C)C)C)CCC=C(C)C | 44567123 |
| IMPHY001600 | Kulactone | CC(=CCC[C@H]1C(=O)O[C@@H]2[C@@H]1[C@]1(C)CC[C@H]3C(=CC[C@@H]4[C@]3(C)CCC(=O)C4(C)C)[C@]1(C2)C)C | 15560423 |
| IMPHY002006 | Gedunin | CC(=O)O[C@@H]1C[C@H]2C(C)(C)C(=O)C=C[C@@]2([C@@H]2[C@]1(C)[C@@]13O[C@@H]1C(=O)O[C@H]([C@@]3(CC2)C)c1ccoc1)C | 12004512 |
| IMPHY002171 | Kulolactone | CC(=CCC[C@H]1C(=O)O[C@@H]2[C@@H]1[C@]1(C)CC[C@H]3C(=CC[C@@H]4[C@]3(C)CC[C@H](C4(C)C)O)[C@]1(C2)C)C | 101289844 |
| IMPHY004200 | 6beta-Hydroxystigmast-4-en-3-one | CC[C@@H](C(C)C)CC[C@H]([C@H]1CC[C@@H]2[C@]1(C)CC[C@H]1[C@H]2C[C@H](C2=CC(=O)CC[C@]12C)O)C | 9823926 |
| IMPHY004432 | Methyl 2,5-dihydroxycinnamate | COC(=O)/C=C/c1cc(O)ccc1O | 5353609 |
| IMPHY005303 | (4bS,8aR)-2,4b,8,8-tetramethyl-7,10-dioxo-5,6,8a,9-tetrahydrophenanthrene-3-carboxylic Acid | O=C1CC[C@]2([C@H](C1(C)C)CC(=O)c1c2cc(C(=O)O)c(c1)C)C | 189727 |
| IMPHY005310 | (4aS,10aR)-7-hydroxy-1,1,4a,6-tetramethyl-3,4,10,10a-tetrahydrophenanthrene-2,9-dione | O=C1CC[C@]2([C@H](C1(C)C)CC(=O)c1c2cc(C)c(c1)O)C | 189404 |
| IMPHY005328 | (4aS,10aR)-6-hydroxy-1,1,4a,7-tetramethyl-3,4,10,10a-tetrahydrophenanthrene-2,9-dione | O=C1CC[C@]2([C@H](C1(C)C)CC(=O)c1c2cc(O)c(c1)C)C | 189403 |
| IMPHY005337 | Nimbionol | COc1cc2C(=O)C[C@@H]3[C@](c2cc1O)(C)CC[C@@H](C3(C)C)O | 189704 |
| IMPHY007375 | Sugiol | O=C1C[C@H]2C(C)(C)CCC[C@@]2(c2c1cc(C(C)C)c(c2)O)C | 94162 |
| IMPHY009443 | Nimbionone | COc1cc2C(=O)C[C@@H]3[C@](c2cc1O)(C)CCC(=O)C3(C)C | 189706 |
| IMPHY011387 | Epoxyazadiradione | CC(=O)O[C@@H]1C[C@H]2C(C)(C)C(=O)C=C[C@@]2([C@@H]2[C@]1(C)[C@@]13O[C@@H]1C(=O)[C@H]([C@@]3(CC2)C)c1ccoc1)C | 49863985 |
| IMPHY012310 | Nimbin | COC(=O)C[C@H]1[C@@]2(C)[C@H](O[C@H]3C2=C(C)[C@@H](C3)c2cocc2)[C@@H]([C@@H]2[C@]1(C)C(=O)C=C[C@@]2(C)C(=O)OC)OC(=O)C | 108058 |
| IMPHY012408 | Nimbosone | COc1cc2c(cc1C(=O)C)CC[C@@H]1[C@]2(C)CCCC1(C)C | 177090 |
| IMPHY013917 | (4aS,10aR)-6,7-dimethoxy-1,1,4a-trimethyl-3,4,10,10a-tetrahydrophenanthrene-2,9-dione | COc1cc2c(cc1OC)C(=O)C[C@@H]1[C@]2(C)CCC(=O)C1(C) | 189660 |
| IMPHY013919 | Margolonone | O=C1CC[C@]2([C@H](C1(C)C)CC(=O)c1c2cc(C)c(c1)C(=O)O)C | 189726 |
| IMPHY013920 | (4bS,8aS)-3,4b,8,8-tetramethyl-10-oxo-6,7,8a,9-tetrahydro-5H-phenanthrene-2-carboxylic Acid | O=C1C[C@H]2C(C)(C)CCC[C@@]2(c2c1cc(C(=O)O)c(c2)C)C | 189728 |
| IMPHY013953 | 6-Hydroxycyclohexa-2,4-dien-1-one | OC1C=CC=CC1=O | 440554 |
| IMPHY014771 | Deacetylgedunin | O=C1O[C@@H](c2ccoc2)[C@]2([C@]3([C@@H]1O3)[C@]1(C)[C@H](O)C[C@@H]3[C@]([C@H]1CC2)(C)C=CC(=O)C3(C)C)C | 3034112 |
| IMPHY014836 | beta-Sitosterol | CC[C@@H](C(C)C)CC[C@H]([C@H]1CC[C@@H]2[C@]1(C)CC[C@H]1[C@H]2CC=C2[C@]1(C)CC[C@@H](C2)O)C | 222284 |
| IMPHY002915 | Benzyl Alcohol | OCc1ccccc1 | 244 |
| IMPHY004388 | Kaempferol | Oc1ccc(cc1)c1oc2cc(O)cc(c2c(=O)c1O)O | 5280863 |
| IMPHY004619 | Quercetin | Oc1cc(O)c2c(c1)oc(c(c2=O)O)c1ccc(c(c1)O)O | 5280343 |
| IMPHY004631 | Stearic acid | CCCCCCCCCCCCCCCCCC(=O)O | 5281 |
| IMPHY005471 | Myricetin | Oc1cc(O)c2c(c1)oc(c(c2=O)O)c1cc(O)c(c(c1)O)O | 5281672 |
| IMPHY006300 | Cholesterol | CC(CCC[C@H]([C@H]1CC[C@@H]2[C@]1(C)CC[C@H]1[C@H]2CC=C2[C@]1(C)CC[C@@H](C2)O)C)C | 5997 |
| IMPHY007212 | Docosanoic acid | CCCCCCCCCCCCCCCCCCCCCC(=O)O | 8215 |
| IMPHY008991 | Benzyl acetate | CC(=O)OCc1ccccc1 | 8785 |
| IMPHY009482 | Nonacosane | CCCCCCCCCCCCCCCCCCCCCCCCCCCCC | 12409 |
| IMPHY010637 | Melicitrin | Oc1cc(O)c2c(c1)oc(c(c2=O)O)c1cc(O[C@@H]2OC[C@@H]([C@@H]([C@H]2O)O)O)c(c(c1)O)O | 5319346 |
| IMPHY011394 | Arachidic acid | CCCCCCCCCCCCCCCCCCCC(=O)O | 10467 |
| IMPHY011797 | Oleic acid | CCCCCCCC/C=CCCCCCCCC(=O)O | 445639 |
| IMPHY012310 | Nimbin | COC(=O)C[C@H]1[C@@]2(C)[C@H](O[C@H]3C2=C(C)[C@@H](C3)c2cocc2)[C@@H]([C@@H]2[C@]1(C)C(=O)C=C[C@@]2(C)C(=O)OC)OC(=O)C | 108058 |
| IMPHY014824 | Astragalin | OC[C@H]1O[C@@H](Oc2c(oc3c(c2=O)c(O)cc(c3)O)c2ccc(cc2)O)[C@@H]([C@H]([C@@H]1O)O)O | 5282102 |
| IMPHY014935 | Hyperoside | OC[C@H]1O[C@@H](Oc2c(oc3c(c2=O)c(O)cc(c3)O)c2ccc(c(c2)O)O)[C@@H]([C@H]([C@H]1O)O)O | 5281643 |
| IMPHY014990 | Linoleic acid | CCCCC/C=CC/C=CCCCCCCCC(=O)O | 5280450 |
| IMPHY015023 | Nimbosterin | CC[C@@H](C(C)C)CC[C@H]([C@H]1CC[C@@H]2[C@]1(C)CC[C@H]1[C@H]2CC=C2[C@]1(C)CC[C@@H](C2)OC1O[C@H](CO)[C@H]([C@@H]([C@H]1O)O)O)C | 6602509 |
| IMPHY000155 | Azadirone | CC(=O)O[C@@H]1C[C@@H]2[C@]([C@@H]3[C@]1(C)C1=CC[C@H]([C@@]1(CC3)C)c1ccoc1)(C)C=CC(=O)C2(C)C | 10906239 |
| IMPHY000668 | Nimbochalcin | CCOCc1c(CCC(=O)c2c(C)cc(cc2C(=O)OCC)O)c(O)c(c(c1O)O)O | 163114683 |
| IMPHY000812 | Triterpenoid | O[C@H]1CC[C@]2([C@H]([C@]1(C)COS(=O)(=O)O)CC[C@@]1([C@@H]2CC=C2[C@@]1(C)CC[C@@]1([C@H]2CC(C)(C)CC1)C(=O)O)C)C | 451674 |
| IMPHY001139 | Azadirolic acid | OC(=O)CC/C=C([C@@H]1CC=C2[C@@]1(C)CC[C@H]1[C@@]2(C)[C@H](O)[C@H]([C@@H]2[C@]1(C)C=CC(=O)C2(C)C)OC(=O)C)/C | 102316534 |
| IMPHY001140 | Azadiradionol | CC(CC[C@@H]([C@@H]1C(=O)C=C2[C@@]1(C)CC[C@H]1[C@@]2(C)[C@H](OC(=O)C)C[C@@H]2[C@]1(C)C=CC(=O)C2(C)C)C)O | 102316535 |
| IMPHY001415 | Azadirol | OC[C@H]([C@@H]1CC=C2[C@@]1(C)CC[C@H]1[C@@]2(C)[C@H](OC(=O)C)C[C@@H]2[C@]1(C)C=CC(=O)C2(C)C)C[C@@H](C(=O)C(O)(C)C)O | 44567142 |
| IMPHY001600 | Kulactone | CC(=CCC[C@H]1C(=O)O[C@@H]2[C@@H]1[C@]1(C)CC[C@H]3C(=CC[C@@H]4[C@]3(C)CCC(=O)C4(C)C)[C@]1(C2)C)C | 15560423 |
| IMPHY001746 | Salimuzzalin | CC(=O)OC1OC(C=C1[C@@H]1CC=C2[C@@]1(C)CC[C@H]1[C@@]2(C)[C@H](O)C[C@@H]2[C@]1(C)C=CC(=O)C2(C)C)OC(=O)C | 15840157 |
| IMPHY002006 | Gedunin | CC(=O)O[C@@H]1C[C@H]2C(C)(C)C(=O)C=C[C@@]2([C@@H]2[C@]1(C)[C@@]13O[C@@H]1C(=O)O[C@H]([C@@]3(CC2)C)c1ccoc1)C | 12004512 |
| IMPHY002653 | Nimbocinol | O=C1C=C2[C@@]([C@@H]1c1ccoc1)(C)CC[C@H]1[C@@]2(C)[C@H](O)C[C@@H]2[C@]1(C)C=CC(=O)C2(C)C | 13875741 |
| IMPHY002655 | 1-Cinnamoylmelianolone | OC[C@@]12[C@@H](OC(=O)/C=C/c3ccccc3)C[C@H]([C@@]3([C@@H]1[C@H](OC3)[C@H]([C@]([C@@H]2C(=O)C)(C)[C@@]12O[C@@]2(C)[C@@H]2C[C@@H]1O[C@@H]1[C@]2(O)C=CO1)O)C) | 13819214 |
| IMPHY003174 | 5-Hydroxymethylfurfural | OCc1ccc(o1)C=O | 237332 |
| IMPHY003825 | Azadiradione | CC(=O)O[C@@H]1C[C@@H]2[C@]([C@@H]3[C@]1(C)C1=CC(=O)[C@H]([C@@]1(CC3)C)c1ccoc1)(C)C=CC(=O)C2(C)C | 12308714 |
| IMPHY004014 | Azadirachtin | C/C=C(/C(=O)O[C@H]1C[C@@H](OC(=O)C)[C@@]2([C@H]3[C@@]41CO[C@]([C@H]4[C@@](C)([C@@H]([C@@H]3OC2)O)[C@@]12O[C@@]2(C)[C@H]2C[C@@H]1O[C@H]1[C@]2(O)C=CO1)(O)C(=O)OC)C(=O)OC)C | 5281303 |
| IMPHY004015 | dimethyl (1S,4S,5R,6S,7S,8R,11S,12R,14S,15R)-12-acetyloxy-4,7-dihydroxy-6-[(1R,2S,6S,8R,9R,11S)-2-hydroxy-11-methyl-5,7,10-trioxatetracyclo[6.3.1.02,6.09,11]dodec-3-en-9-yl]-6-methyl-14-[(E)-2-methylb | C/C=C(/C(=O)O[C@H]1C[C@@H](OC(=O)C)[C@@]2([C@H]3[C@@]41CO[C@]([C@H]4[C@@](C)([C@@H]([C@@H]3OC2)O)[C@@]12O[C@@]2(C)[C@@H]2C[C@H]1O[C@H]1[C@]2(O)C=CO1)(O)C(=O)OC)C(=O)OC)C | 44584063 |
| IMPHY004388 | Kaempferol | Oc1ccc(cc1)c1oc2cc(O)cc(c2c(=O)c1O)O | 5280863 |
| IMPHY005039 | 6-Acetylnimbandiol | COC(=O)C[C@H]1[C@@]2(C)[C@H](O[C@H]3C2=C(C)[C@@H](C3)c2cocc2)[C@@H]([C@@H]2[C@]1(C)C(=O)C=C[C@@]2(C)O)OC(=O)C | 52952216 |
| IMPHY005199 | Azadirachtol | COC(=O)[C@H]1OC[C@]23[C@@H]1[C@@](C)([C@@H]([C@H]1[C@H]3[C@@]([C@@H](C[C@@H]2O)O)(CO1)C(=O)OC)O)[C@@]12O[C@@]2(C)[C@H]2C[C@@H]1O[C@H]1[C@]2(O)C=CO1 | 23256847 |
| IMPHY005274 | [(5R,6R,7S,9R,10R,13S,17R)-6-hydroxy-17-(2-hydroxy-5-oxo-2H-furan-3-yl)-4,4,8,10,13-pentamethyl-3-oxo-5,6,7,9,11,12,16,17-octahydrocyclopenta[a]phenanthren-7-yl] acetate | CC(=O)O[C@@H]1[C@H](O)[C@@H]2[C@]([C@@H]3C1(C)C1=CC[C@H]([C@]1(C)CC3)C1=CC(=O)OC1O)(C)C=CC(=O)C2(C)C | 184310 |
| IMPHY005293 | Nimolicinol | CC(=O)O[C@@H]1C[C@@H]2[C@]([C@@H]3[C@]1(C)C1=CC(=O)O[C@]([C@@]1(CC3)C)(O)c1ccoc1)(C)C=CC(=O)C2(C)C | 184937 |
| IMPHY005957 | Nimolinone | C=C[C@@H]1OC(=O)[C@@H](C1)[C@@H]1CC[C@]2([C@@]1(C)CC=C1C2=CC[C@@H]2[C@]1(C)CCC(=O)C2(C)C)C | 56841069 |
| IMPHY006063 | 1-Docosene | CCCCCCCCCCCCCCCCCCCCC=C | 74138 |
| IMPHY006237 | Melianoninol | O=C/C=C/c1cc(OC)c(cc1O)[C@@H]1Oc2c([C@@H]1CO)cc(cc2)OC | 6438693 |
| IMPHY006837 | (5R,9R,10R,13S,14S,17S)-17-[(2R,3S,5R)-5-[(2S)-3,3-dimethyloxiran-2-yl]-2-hydroxyoxolan-3-yl]-4,4,10,13,14-pentamethyl-1,2,5,6,9,11,12,15,16,17-decahydrocyclopenta[a]phenanthren-3-one | O[C@@H]1O[C@H](C[C@H]1[C@@H]1CC[C@]2([C@@]1(C)CC[C@H]1C2=CC[C@@H]2[C@]1(C)CCC(=O)C2(C)C)C)[C@@H]1OC1(C)C | 44575793 |
| IMPHY006850 | (2R,3S,5R)-5-[(2S)-3,3-dimethyloxiran-2-yl]-3-[(3S,5R,9R,10R,13S,14S,17S)-3-hydroxy-4,4,10,13,14-pentamethyl-2,3,5,6,9,11,12,15,16,17-decahydro-1H-cyclopenta[a]phenanthren-17-yl]oxolan-2-ol | O[C@@H]1O[C@H](C[C@H]1[C@@H]1CC[C@]2([C@@]1(C)CC[C@H]1C2=CC[C@@H]2[C@]1(C)CC[C@@H](C2(C)C)O)C)[C@@H]1OC1(C)C | 101289709 |
| IMPHY006854 | (1R,2R,5R,6S,10R,11S,12R,15R,16R,18S,19R)-6-(furan-3-yl)-11,16,18-trihydroxy-1,5,10,15-tetramethyl-13-oxapentacyclo[10.6.1.02,10.05,9.015,19]nonadec-8-en-4-one | O[C@H]1C[C@@H](O)[C@@]2([C@H]3[C@@]1(C)[C@H]1CC(=O)[C@]4(C(=CC[C@H]4c4ccoc4)[C@]1(C)[C@@H]([C@@H]3OC2)O)C)C | 101306757 |
| IMPHY007084 | Vanillic acid | COc1cc(ccc1O)C(=O)O | 8468 |
| IMPHY007327 | Palmitic acid | CCCCCCCCCCCCCCCC(=O)O | 985 |
| IMPHY009375 | Docosane | CCCCCCCCCCCCCCCCCCCCCC | 12405 |
| IMPHY009552 | Naheedin | CCC([C@H]1O[C@@H]([C@H](C1)[C@@H]1CC=C2[C@@]1(C)C[C@@H](O)[C@H]1[C@@]2(C)[C@H](OC(=O)C)C[C@@H]2[C@]1(C)C=CC(=O)C2(C)C)O)C | 129754 |
| IMPHY010341 | 17-Hydroxyazadiradione | CC(=O)O[C@@H]1C[C@@H]2[C@]([C@@H]3[C@]1(C)C1=CC(=O)[C@@]([C@@]1(CC3)C)(O)c1ccoc1)(C)C=CC(=O)C2(C)C | 52951892 |
| IMPHY010454 | Vilasinin | O[C@H]1C[C@@H](O)[C@@]2([C@H]3[C@@]1(C)[C@H]1CC[C@@]4(C(=CC[C@H]4c4ccoc4)[C@]1(C)[C@@H]([C@@H]3OC2)O)C)C | 102090424 |
| IMPHY010554 | Salannin | COC(=O)C[C@@H]1[C@@]2(C)[C@@H](OC(=O)/C(=C/C)/C)C[C@H]([C@@]3([C@@H]2[C@H]([C@@H]2[C@@]1(C)C1=C(C)[C@@H](C[C@H]1O2)c1ccoc1)OC3)C)OC(=O)C | 6437066 |
| IMPHY011387 | Epoxyazadiradione | CC(=O)O[C@@H]1C[C@H]2C(C)(C)C(=O)C=C[C@@]2([C@@H]2[C@]1(C)[C@@]13O[C@@H]1C(=O)[C@H]([C@@]3(CC2)C)c1ccoc1)C | 49863985 |
| IMPHY011498 | 3-Deacetylsalannin | COC(=O)C[C@@H]1[C@@]2(C)[C@@H](OC(=O)/C(=C/C)/C)C[C@H]([C@@]3([C@@H]2[C@H]([C@@H]2[C@@]1(C)C1=C(C)[C@@H](C[C@H]1O2)c1ccoc1)OC3)C)O | 14458886 |
| IMPHY011741 | Tannic acid | O=C(c1cc(O)c(c(c1)OC(=O)c1cc(O)c(c(c1)O)O)O)O[C@@H]1[C@@H](COC(=O)c2cc(O)c(c(c2)OC(=O)c2cc(O)c(c(c2)O)O)O)O[C@H]([C@@H]([C@H]1OC(=O)c1cc(O)c(c(c1)OC(=O)c1cc(O)c(c(c1)O)O)O)OC(=O)c1cc(O)c(c(c1)OC(=O)c1cc(O)c(c(c1)O)O)O)OC(=O)c1cc(O)c(c(c1)OC(=O)c1cc(O)c(c(c1)O)O)O | 16129778 |
| IMPHY011797 | Oleic acid | CCCCCCCC/C=CCCCCCCCC(=O)O | 445639 |
| IMPHY012310 | Nimbin | COC(=O)C[C@H]1[C@@]2(C)[C@H](O[C@H]3C2=C(C)[C@@H](C3)c2cocc2)[C@@H]([C@@H]2[C@]1(C)C(=O)C=C[C@@]2(C)C(=O)OC)OC(=O)C | 108058 |
| IMPHY012479 | 2-Methyltricosane | CCCCCCCCCCCCCCCCCCCCCC(C)C | 283510 |
| IMPHY012723 | Linolenic acid | CC/C=CC/C=CC/C=CCCCCCCCC(=O)O | 5280934 |
| IMPHY013522 | Triterpenoids | O[C@H]1C[C@@]2(C)C(=CC[C@@]3([C@@H]2CC=C2[C@@]3(C)CC[C@@]3([C@H]2CC(C)(C)CC3)C(=O)O)C)[C@]([C@H]1O)(C)O | 71597391 |
| IMPHY000150 | 6-Deacetylnimbin | COC(=O)C[C@H]1[C@@]2(C)[C@H](O[C@H]3C2=C(C)[C@@H](C3)c2cocc2)[C@@H]([C@@H]2[C@]1(C)C(=O)C=C[C@@]2(C)C(=O)OC)O | 10505484 |
| IMPHY000193 | Azadirachtanin | CC(=O)O[C@@H]1OC[C@@]23C([C@@]1(C)[C@H](OC(=O)C)C[C@@H]2O)C[C@H]([C@@]1([C@@H]3C(=O)[C@H](OC(=O)C)[C@@]2(C1=CC[C@H]2c1ccoc1)C)C)O | 102146586 |
| IMPHY000464 | Isomargosinolide | COC(=O)C[C@H]1[C@]2(C)C3=C(C)[C@@H](C[C@H]3O[C@@H]2[C@@H]2[C@@H]3[C@]1(C)C=CC(=O)[C@@]3(C)CO2)C1=CC(=O)OC1O | 0 |
| IMPHY000663 | Methyl 2-[6-(furan-3-yl)-7,9,11,15-tetramethyl-12,16-dioxo-3,17-dioxapentacyclo[9.6.1.02,9.04,8.015,18]octadeca-7,13-dien-10-yl]acetate | COC(=O)CC1C2(C)C(OC3C2=C(C)C(C3)c2cocc2)C2C3C1(C)C(=O)C=CC3(C)C(=O)O2 | 100017 |
| IMPHY001280 | Nimbinene | COC(=O)C[C@H]1[C@@]2(C)[C@H](OC3C2=C(C)[C@@H](C3)c2cocc2)[C@@H](C2[C@]1(C)C(=O)CC=C2C)OC(=O)C | 44715635 |
| IMPHY002244 | Isoazadirolide | COC(=O)CC1C2(C)C(OC3C2=C(C)C(C3)C2=CC(=O)OC2O)C2C3C1(C)C(OC(=O)C=C(C)C)CC(C3(C)CO2)O | 101425842 |
| IMPHY002654 | Nimbocinolide | CC(=O)O[C@@H]1C[C@@H]2[C@]([C@@H]3[C@]1(C)C1=CC[C@H]([C@@]1([C@H]([C@H]3OC(=O)C(O)(C)C)O)C)C1=CC(OC1=O)O)(C)C=CC(=O)C2(C)C | 13875774 |
| IMPHY002788 | 5,7-Dihydroxy-4'-methoxy-8,3'-di-C-prenylflavanone | COc1ccc(cc1CC=C(C)C)C1CC(=O)c2c(O1)c(CC=C(C)C)c(cc2O)O | 14492795 |
| IMPHY002857 | 28-Deoxonimbolide | COC(=O)C[C@H]1[C@@]2(C)[C@H](O[C@H]3C2=C(C)[C@@H](C3)c2cocc2)[C@H]2[C@@H]3[C@]1(C)C(=O)C=C[C@@]3(C)CO2 | 14467538 |
| IMPHY002935 | 1-Nonacosanol | CCCCCCCCCCCCCCCCCCCCCCCCCCCCCO | 243696 |
| IMPHY003537 | Tetradecanal | CCCCCCCCCCCCCC=O | 31291 |
| IMPHY004014 | Azadirachtin | C/C=C(/C(=O)O[C@H]1C[C@@H](OC(=O)C)[C@@]2([C@H]3[C@@]41CO[C@]([C@H]4[C@@](C)([C@@H]([C@@H]3OC2)O)[C@@]12O[C@@]2(C)[C@H]2C[C@@H]1O[C@H]1[C@]2(O)C=CO1)(O)C(=O)OC)C(=O)OC)C | 5281303 |
| IMPHY004521 | alpha-Patchoulene | CC1CCC23C1CC(C3(C)C)CC=C2C | 521710 |
| IMPHY004619 | Quercetin | Oc1cc(O)c2c(c1)oc(c(c2=O)O)c1ccc(c(c1)O)O | 5280343 |
| IMPHY005267 | (9R,10R,13S,14S,17R)-17-[(2R)-2-[(1R,2S)-1,3-dihydroxy-2-methylpropyl]-2,3-dihydrofuran-4-yl]-4,4,10,13,14-pentamethyl-1,2,5,6,9,11,12,15,16,17-decahydrocyclopenta[a]phenanthren-3-one | OC[C@@H]([C@H]([C@@H]1OC=C(C1)[C@@H]1CC[C@]2([C@@]1(C)CC[C@H]1C2=CCC2[C@]1(C)CCC(=O)C2(C)C)C)O)C | 184503 |
| IMPHY005274 | [(5R,6R,7S,9R,10R,13S,17R)-6-hydroxy-17-(2-hydroxy-5-oxo-2H-furan-3-yl)-4,4,8,10,13-pentamethyl-3-oxo-5,6,7,9,11,12,16,17-octahydrocyclopenta[a]phenanthren-7-yl] acetate | CC(=O)O[C@@H]1[C@H](O)[C@@H]2[C@]([C@@H]3C1(C)C1=CC[C@H]([C@]1(C)CC3)C1=CC(=O)OC1O)(C)C=CC(=O)C2(C)C | 184310 |
| IMPHY005618 | Germacrene B | C/C/1=CCC/C(=C/CC(=C(C)C)CC1)/C | 5281519 |
| IMPHY006017 | Isomeldenin | CC(=O)OC1[C@H](O)[C@@H]2[C@]([C@@H]3[C@]1(C)C1=CC[C@H]([C@@]1(CC3)C)c1ccoc1)(C)CCC(=O)C2(C)C | 76316558 |
| IMPHY006238 | [(4S,5R,6R,11R,12R,15S,16R,17Z)-17-(2,2-dihydroxyethylidene)-5-hydroxy-3,7,7,11,15-pentamethyl-8,18-dioxo-19-oxapentacyclo[14.3.1.02,15.03,12.06,11]icosa-1,9-dien-4-yl] acetate | CC(=O)O[C@@H]1[C@H](O)[C@@H]2[C@]([C@@H]3C1(C)C1=C4OC(=O)/C(=CC(O)O)/[C@@H]([C@@]1(CC3)C)C4)(C)C=CC(=O)C2(C)C | 6442906 |
| IMPHY006612 | Corosolic acid | C[C@@H]1CC[C@]2([C@@H]([C@H]1C)C1=CC[C@H]3[C@@]([C@@]1(CC2)C)(C)CC[C@@H]1[C@]3(C)C[C@@H](O)[C@@H](C1(C)C)O)C(=O)O | 6918774 |
| IMPHY006786 | Margosinolide | COC(=O)C[C@H]1[C@@]2(C)[C@H](O[C@H]3C2=C(C)[C@@H](C3)C2=CC(OC2=O)O)[C@H]2[C@@H]3[C@]1(C)C=CC(=O)[C@@]3(C)CO2 | 21681049 |
| IMPHY006799 | Meldenin | CC(=O)O[C@H]1[C@@H](O)[C@]2(C)[C@@H]([C@@]3([C@@H]1C(C)(C)C(=O)CC3)C)CC[C@@]1(C2=CC[C@H]1c1ccoc1)C | 101289833 |
| IMPHY006827 | (1S)-1-[(2R,4S,5R)-5-hydroxy-4-[(3S,5R,9R,10R,13S,14S,17S)-3-hydroxy-4,4,10,13,14-pentamethyl-2,3,5,6,9,11,12,15,16,17-decahydro-1H-cyclopenta[a]phenanthren-17-yl]oxolan-2-yl]-2-methylpropane-1,2-diol | O[C@@H]1O[C@H](C[C@H]1[C@@H]1CC[C@]2([C@@]1(C)CC[C@H]1C2=CC[C@@H]2[C@]1(C)CC[C@@H](C2(C)C)O)C)[C@@H](C(O)(C)C)O | 101650343 |
| IMPHY006853 | 2H-Cyclopenta(b)naphtho(2,3-d)furan-10-carboxylic acid,2-(3-furanyl)-3,3a,4a,5,5a,6,9,9a,10,10a-decahydro-5,6-dihydroxy-1,6,9a,10a-tetramethyl-9-oxo-, methyl ester, (2R,3aS,4aS,5R,5aS,6R,9aR,10S,10aR | COC(=O)C[C@H]1[C@@]2(C)[C@H](O[C@@H]3C2=C(C)[C@@H](C3)c2cocc2)[C@@H]([C@@H]2[C@@]1(C)C(=O)C=C[C@@]2(C)O)O | 157277 |
| IMPHY007273 | 1-Hexacosanol | CCCCCCCCCCCCCCCCCCCCCCCCCCO | 68171 |
| IMPHY007392 | 2',3'-Dehydrosalannol | COC(=O)C[C@@H]1[C@@]2(C)[C@@H](OC(=O)C=C(C)C)C[C@H]([C@@]3([C@@H]2[C@H]([C@@H]2[C@@]1(C)C1=C(C)[C@@H](C[C@H]1O2)c1ccoc1)OC3)C)O | 91886694 |
| IMPHY008724 | Isorhamnetin | COc1cc(ccc1O)c1oc2cc(O)cc(c2c(=O)c1O)O | 5281654 |
| IMPHY007467 | Hexadecanal | CCCCCCCCCCCCCCCC=O | 984 |
| IMPHY009482 | Nonacosane | CCCCCCCCCCCCCCCCCCCCCCCCCCCCC | 12409 |
| IMPHY010080 | beta-Elemene | C=C[C@]1(C)CC[C@H](C[C@H]1C(=C)C)C(=C)C | 6918391 |
| IMPHY010224 | Nimocinol | CC(=O)O[C@@H]1[C@H](O)[C@@H]2[C@]([C@@H]3C1(C)C1=CC[C@H]([C@@]1(CC3)C)c1ccoc1)(C)C=CC(=O)C2(C)C | 178770 |
| IMPHY010454 | Vilasinin | O[C@H]1C[C@@H](O)[C@@]2([C@H]3[C@@]1(C)[C@H]1CC[C@@]4(C(=CC[C@H]4c4ccoc4)[C@]1(C)[C@@H]([C@@H]3OC2)O)C)C | 102090424 |
| IMPHY011498 | 3-Deacetylsalannin | COC(=O)C[C@@H]1[C@@]2(C)[C@@H](OC(=O)/C(=C/C)/C)C[C@H]([C@@]3([C@@H]2[C@H]([C@@H]2[C@@]1(C)C1=C(C)[C@@H](C[C@H]1O2)c1ccoc1)OC3)C)O | 14458886 |
| IMPHY011541 | Scopoletin | COc1cc2ccc(=O)oc2cc1O | 5280460 |
| IMPHY011586 | (S,1Z,6Z)-8-Isopropyl-1-methyl-5-methylenecyclodeca-1,6-diene | C/C/1=C/CCC(=C)/C=C[C@@H](CC1)C(C)C | 91723653 |
| IMPHY011620 | Lutein | C/C(=CC=CC=C(C=CC=C(C=CC1=C(C)C[C@H](CC1(C)C)O)/C)/C)/C=C/C=C(/C=C/[C@H]1C(=C[C@@H](CC1(C)C)O)C)C | 5281243 |
| IMPHY011707 | beta-Carotene | C/C(=CC=CC=C(C=CC=C(C=CC1=C(C)CCCC1(C)C)/C)/C)/C=C/C=C(/C=C/C1=C(C)CCCC1(C)C)C | 5280489 |
| IMPHY011761 | Humulene | C/C/1=CCC(C)(C)/C=C/C/C(=C/CC1)/C | 5281520 |
| IMPHY011792 | gamma-Muurolene | CC1=C[C@@H]2[C@H](CC1)C(=C)CC[C@H]2C(C)C | 12313020 |
| IMPHY011985 | Nicotiflorin | Oc1ccc(cc1)c1oc2cc(O)cc(c2c(=O)c1O[C@@H]1O[C@H](CO[C@@H]2O[C@@H](C)[C@@H]([C@H]([C@H]2O)O)O)[C@H]([C@@H]([C@H]1O)O)O)O | 5318767 |
| IMPHY012029 | Sterol | OC1CCC2C(C1)CCC1C2CCC2C1CCC2 | 1107 |
| IMPHY012921 | gamma-Elemene | C=C[C@]1(C)CCC(=C(C)C)C[C@H]1C(=C)C | 6432312 |
| IMPHY013093 | delta-Elemene | C=C[C@@]1(C)CCC(=C[C@@H]1C(=C)C)C(C)C | 12309449 |
| IMPHY013522 | Triterpenoids | O[C@H]1C[C@@]2(C)C(=CC[C@@]3([C@@H]2CC=C2[C@@]3(C)CC[C@@]3([C@H]2CC(C)(C)CC3)C(=O)O)C)[C@]([C@H]1O)(C)O | 71597391 |
| IMPHY014396 | Quercetin-3-glucoside | OC[C@H]1O[C@@H](Oc2c(oc3c(c2=O)c(O)cc(c3)O)c2ccc(c(c2)O)[O-])[C@@H]([C@H]([C@@H]1O)O)O | 25203368 |
| IMPHY014831 | beta-Caryophyllene | C/C/1=CCCC(=C)[C@@H]2[C@@H](CC1)C(C2)(C)C | 5281515 |
| IMPHY014836 | beta-Sitosterol | CC[C@@H](C(C)C)CC[C@H]([C@H]1CC[C@@H]2[C@]1(C)CC[C@H]1[C@H]2CC=C2[C@]1(C)CC[C@@H](C2)O)C | 222284 |
| IMPHY014838 | Daucosterol | CC[C@@H](C(C)C)CC[C@H]([C@H]1CC[C@@H]2[C@]1(C)CC[C@H]1[C@H]2CC=C2[C@]1(C)CC[C@@H](C2)O[C@@H]1O[C@H](CO)[C@H]([C@@H]([C@H]1O)O)O)C | 5742590 |
| IMPHY014842 | Stigmasterol | CC[C@@H](C(C)C)/C=C/[C@H]([C@H]1CC[C@@H]2[C@]1(C)CC[C@H]1[C@H]2CC=C2[C@]1(C)CC[C@@H](C2)O)C | 5280794 |
| IMPHY014935 | Hyperoside | OC[C@H]1O[C@@H](Oc2c(oc3c(c2=O)c(O)cc(c3)O)c2ccc(c(c2)O)O)[C@@H]([C@H]([C@H]1O)O)O | 5281643 |
| IMPHY015047 | Rutin | Oc1cc(O)c2c(c1)oc(c(c2=O)O[C@@H]1O[C@H](CO[C@@H]2O[C@@H](C)[C@@H]([C@H]([C@H]2O)O)O)[C@H]([C@@H]([C@H]1O)O)O)c1ccc(c(c1)O)O | 5280805 |
| IMPHY015054 | Quercitrin | Oc1cc(O)c2c(c1)oc(c(c2=O)O[C@@H]1O[C@@H](C)[C@@H]([C@H]([C@H]1O)O)O)c1ccc(c(c1)O)O | 5280459 |
| IMPHY015072 | beta-Sitosterol-beta-D-glucoside | CC[C@@H](C(C)C)CC[C@H]([C@H]1CC[C@@H]2[C@]1(C)CC[C@H]1[C@@H]2CC=C2[C@@]1(C)CC[C@@H](C2)O[C@H]1O[C@H](CO)[C@H]([C@@H]([C@H]1O)O)O)C | 12309055 |
| IMPHY015123 | alpha-Copaene | CC([C@@H]1CC[C@]2([C@@H]3[C@H]1C2C(=CC3)C)C)C | 70678558 |
| IMPHY016012 | Allo-Aromadendrene | C[C@@H]1CC[C@H]2[C@@H]1C1C(C1(C)C)CCC2=C | 42608158 |
| IMPHY004187 | L-(+)-Arabinose | OC[C@@H]([C@@H]([C@H](C=O)O)O)O | 5460291 |
| IMPHY012050 | D-Galactose | OC[C@H]1OC(O)[C@@H]([C@H]([C@H]1O)O)O | 6036 |
| IMPHY014892 | D-Glucosamine | OC[C@H]1OC(O)[C@@H]([C@H]([C@@H]1O)O)N | 439213 |
| IMPHY000016 | Nimbidiol | O=C1C[C@H]2C(C)(C)CCC[C@@]2(c2c1cc(O)c(c2)O)C | 11334829 |
| IMPHY001121 | Azadirinin | CC(=O)O[C@@H]1C2C(C)(C)[C@H](OC(=O)C)C[C@@H]([C@]2(C)[C@@H]2[C@]([C@@H]1O)(C)C1=CC(=O)O[C@H]([C@@]1(CC2)C)c1ccoc1)OC(=O)/C=C/c1ccccc1 | 102275331 |
| IMPHY001931 | Vanillin | COc1cc(C=O)ccc1O | 1183 |
| IMPHY002183 | Margocinin | OCC(c1cc2C(=O)C[C@@H]3[C@](c2cc1O)(C)CCC(=O)C3(C)C)C | 101529197 |
| IMPHY002184 | Margocilin | CC(c1cc2C(=O)C[C@@H]3[C@](c2cc1O)(C)CC[C@@H](C3(C)C)O)C | 101529198 |
| IMPHY002229 | Cycloeucalenol | C=C(C(C)C)CC[C@H]([C@H]1CC[C@@]2([C@]1(C)CC[C@@]13[C@H]2CC[C@@H]2[C@]3(C1)CC[C@@H]([C@H]2C)O)C)C | 101690 |
| IMPHY002557 | Nimbolicin | OC1OC2CC(C(=C2C2(C(C1)C1(C)C(OC(=O)C(=C)C)CC(C3(C1C(C2OC(=O)/C=C/c1ccccc1)OC3)C)OC(=O)C)C)C)c1ccoc1 | 14563366 |
| IMPHY003342 | Nimolinin | O=C1C[C@@]2(O)C(C)(C)CCC[C@@]2(c2c1cc(C(C)C)c(c2)O)C | 180429 |
| IMPHY003804 | Cycloeucalenone | C=C(C(C)C)CC[C@H]([C@H]1CC[C@@]2([C@]1(C)CC[C@@]13[C@H]2CC[C@@H]2[C@]3(C1)CCC(=O)[C@H]2C)C)C | 21594790 |
| IMPHY003979 | beta-Sitostenone | CC[C@@H](C(C)C)CC[C@H](C1CCC2[C@]1(C)CCC1C2CCC2=CC(=O)CC[C@]12C)C | 60123241 |
| IMPHY005184 | Margocin | O=C1CC[C@]2([C@H](C1(C)C)CC(=O)c1c2ccc(c1)C(C)C)C | 21632833 |
| IMPHY005977 | Campestan-3-one | O=C1CC[C@]2(C(C1)CC[C@@H]1[C@@H]2CC[C@]2([C@H]1CC[C@@H]2[C@@H](CC[C@H](C(C)C)C)C)C)C | 53754390 |
| IMPHY006558 | 1-Triacontanol | CCCCCCCCCCCCCCCCCCCCCCCCCCCCCCO | 68972 |
| IMPHY007084 | Vanillic acid | COc1cc(ccc1O)C(=O)O | 8468 |
| IMPHY007657 | Azedarachin C | CC(=O)O[C@@H]1C[C@H](O)[C@]23[C@H]([C@@]1(C)[C@@H](OC2)OC(=O)C(C)C)C[C@H]([C@@]1([C@@H]3C(=O)C[C@@]2([C@]31O[C@@H]3C[C@H]2c1ccoc1)C)C)O | 10348330 |
| IMPHY007664 | Azedarachin A | CCC(C(=O)O[C@@H]1OC[C@@]23[C@H]([C@@]1(C)[C@H](OC(=O)C)C[C@@H]2O)C[C@H]([C@@]1([C@@H]3C(=O)[C@H](O)[C@@]2([C@]31O[C@@H]3C[C@H]2c1ccoc1)C)C)O)C | 101930511 |
| IMPHY007672 | Azedarachin B | CC(=O)O[C@@H]1C[C@H](O)[C@]23[C@H]([C@]1(C)[C@@H](OC2)OC(=O)C(C)C)C[C@H]([C@@]1([C@@H]3C(=O)[C@H](O)[C@@]2([C@]31O[C@@H]3C[C@H]2c1ccoc1)C)C)O | 102586008 |
| IMPHY009726 | Nimbilin | C/C=C(/C(=O)OC1CC(OC(=O)C)C2(C3C1(C)C1CC(O)OC4C(=C(C)C(C4)c4ccoc4)C1(C)C(C3OC2)OC(=O)/C=C/c1ccccc1)C)C | 6442484 |
| IMPHY011393 | 24-Methylenecycloartanol | C=C(C(C)C)CC[C@H]([C@H]1CC[C@@]2([C@]1(C)CC[C@@]13[C@H]2CC[C@@H]2[C@]3(C1)CC[C@@H](C2(C)C)O)C)C | 94204 |
| IMPHY011960 | Cinnamic acid | OC(=O)/C=C/c1ccccc1 | 444539 |
| IMPHY012310 | Nimbin | COC(=O)C[C@H]1[C@@]2(C)[C@H](O[C@H]3C2=C(C)[C@@H](C3)c2cocc2)[C@@H]([C@@H]2[C@]1(C)C(=O)C=C[C@@]2(C)C(=O)OC)OC(=O)C | 108058 |
| IMPHY000016 | Nimbidiol | O=C1C[C@H]2C(C)(C)CCC[C@@]2(c2c1cc(O)c(c2)O)C | 11334829 |
| IMPHY000060 | Myristic acid | CCCCCCCCCCCCCC(=O)O | 11005 |
| IMPHY000150 | 6-Deacetylnimbin | COC(=O)C[C@H]1[C@@]2(C)[C@H](O[C@H]3C2=C(C)[C@@H](C3)c2cocc2)[C@@H]([C@@H]2[C@]1(C)C(=O)C=C[C@@]2(C)C(=O)OC)O | 10505484 |
| IMPHY000155 | Azadirone | CC(=O)O[C@@H]1C[C@@H]2[C@]([C@@H]3[C@]1(C)C1=CC[C@H]([C@@]1(CC3)C)c1ccoc1)(C)C=CC(=O)C2(C)C | 10906239 |
| IMPHY000812 | Triterpenoid | O[C@H]1CC[C@]2([C@H]([C@]1(C)COS(=O)(=O)O)CC[C@@]1([C@@H]2CC=C2[C@@]1(C)CC[C@@]1([C@H]2CC(C)(C)CC1)C(=O)O)C)C | 451674 |
| IMPHY001280 | Nimbinene | COC(=O)C[C@H]1[C@@]2(C)[C@H](OC3C2=C(C)[C@@H](C3)c2cocc2)[C@@H](C2[C@]1(C)C(=O)CC=C2C)OC(=O)C | 44715635 |
| IMPHY001341 | Limbocidin | C/C=C(/C(=O)O[C@H]1C[C@@H](OC(=O)C)[C@]2([C@H]3[C@@]1(C)[C@H]1[C@H](O)[C@H](O)[C@]4([C@]5([C@]1(C)[C@@H]([C@H]3OC2)O)O[C@@H]5C(=O)[C@H]4C1=CC(=O)OC1=O)C)C)C | 102588495 |
| IMPHY001415 | Azadirol | OC[C@H]([C@@H]1CC=C2[C@@]1(C)CC[C@H]1[C@@]2(C)[C@H](OC(=O)C)C[C@@H]2[C@]1(C)C=CC(=O)C2(C)C)C[C@@H](C(=O)C(O)(C)C)O | 44567142 |
| IMPHY002006 | Gedunin | CC(=O)O[C@@H]1C[C@H]2C(C)(C)C(=O)C=C[C@@]2([C@@H]2[C@]1(C)[C@@]13O[C@@H]1C(=O)O[C@H]([C@@]3(CC2)C)c1ccoc1)C | 12004512 |
| IMPHY002620 | Nimbanal | COC(=O)C[C@H]1[C@@]2(C)[C@H](O[C@H]3C2=C(C)[C@@H](C3)c2cocc2)[C@@H]([C@@H]2[C@]1(C)C(=O)C=C[C@@]2(C)C=O)OC(=O)C | 14194023 |
| IMPHY003237 | 2,4-Dimethylthiophene | Cc1scc(c1)C | 34296 |
| IMPHY003824 | 17-Epiazadiradione | CC(=O)O[C@@H]1C[C@@H]2[C@]([C@@H]3[C@]1(C)C1=CC(=O)[C@@H]([C@@]1(CC3)C)c1ccoc1)(C)C=CC(=O)C2(C)C | 12308716 |
| IMPHY003825 | Azadiradione | CC(=O)O[C@@H]1C[C@@H]2[C@]([C@@H]3[C@]1(C)C1=CC(=O)[C@H]([C@@]1(CC3)C)c1ccoc1)(C)C=CC(=O)C2(C)C | 12308714 |
| IMPHY004014 | Azadirachtin | C/C=C(/C(=O)O[C@H]1C[C@@H](OC(=O)C)[C@@]2([C@H]3[C@@]41CO[C@]([C@H]4[C@@](C)([C@@H]([C@@H]3OC2)O)[C@@]12O[C@@]2(C)[C@H]2C[C@@H]1O[C@H]1[C@]2(O)C=CO1)(O)C(=O)OC)C(=O)OC)C | 5281303 |
| IMPHY004015 | dimethyl (1S,4S,5R,6S,7S,8R,11S,12R,14S,15R)-12-acetyloxy-4,7-dihydroxy-6-[(1R,2S,6S,8R,9R,11S)-2-hydroxy-11-methyl-5,7,10-trioxatetracyclo[6.3.1.02,6.09,11]dodec-3-en-9-yl]-6-methyl-14-[(E)-2-methylb | C/C=C(/C(=O)O[C@H]1C[C@@H](OC(=O)C)[C@@]2([C@H]3[C@@]41CO[C@]([C@H]4[C@@](C)([C@@H]([C@@H]3OC2)O)[C@@]12O[C@@]2(C)[C@@H]2C[C@H]1O[C@H]1[C@]2(O)C=CO1)(O)C(=O)OC)C(=O)OC)C | 44584063 |
| IMPHY004024 | dimethyl (2aR,3S,4S,4aR,5S,7aS,8R,10R,10aS,10bR)-10-acetoxy-3,5-dihydroxy-4-[(1aR,2S,3aS,6aS,7S,7aS)-6a-hydroxy-7a-methyl-3a,6a,7,7a-tetrahydro-2,7-methanofuro[2,3-b]oxireno[e]oxepin-1a(2H)-yl]-4-meth | C/C=C(/C(=O)O[C@@H]1C[C@@H](OC(=O)C)[C@@]2([C@H]3[C@@]41CO[C@]([C@H]4[C@@](C)([C@@H]([C@@H]3OC2)O)[C@@]12O[C@@]2(C)[C@H]2C[C@@H]1O[C@H]1[C@]2(O)C=CO1)(O)C(=O)OC)C(=O)OC)C | 16126804 |
| IMPHY004631 | Stearic acid | CCCCCCCCCCCCCCCCCC(=O)O | 5281 |
| IMPHY005037 | 1,3-Diacetylvilasinin | CC(=O)O[C@H]1C[C@@H](OC(=O)C)[C@@]2([C@H]3[C@@]1(C)[C@H]1CC[C@@]4(C(=CC[C@H]4c4ccoc4)[C@]1(C)[C@@H]([C@@H]3OC2)O)C)C | 52952013 |
| IMPHY005039 | 6-Acetylnimbandiol | COC(=O)C[C@H]1[C@@]2(C)[C@H](O[C@H]3C2=C(C)[C@@H](C3)c2cocc2)[C@@H]([C@@H]2[C@]1(C)C(=O)C=C[C@@]2(C)O)OC(=O)C | 52952216 |
| IMPHY005723 | Ohchinolide B | C/C=C(/C(=O)O[C@@H]1[C@@H]2OC[C@]3([C@H]2[C@]([C@@H]2[C@]1(C)C1=C(C)[C@@H](C[C@@H]1OC(=O)C2)c1ccoc1)(C)[C@H](C[C@H]3OC(=O)C)OC(=O)C)C)C | 21581584 |
| IMPHY005975 | 11beta-Azadirachtin H | C/C=C(/C(=O)O[C@H]1C[C@@H](OC(=O)C)[C@@]2([C@H]3[C@@]41CO[C@H]([C@H]4[C@@](C)([C@@H]([C@@H]3OC2)O)[C@@]12O[C@@]2(C)[C@H]2C[C@@H]1O[C@H]1[C@]2(O)C=CO1)O)C(=O)OC)C | 76327056 |
| IMPHY006221 | Tiglic acid | C/C(=CC)/C(=O)O | 125468 |
| IMPHY006286 | [(4R,5R,6S,7S,8R,11R,12R,14S,15S)-12-acetyloxy-4,7-dihydroxy-6-[(1R,2S,6S,9R,11S)-2-hydroxy-11-methyl-5,7,10-trioxatetracyclo[6.3.1.02,6.09,11]dodec-3-en-9-yl]-6,11-dimethyl-3,9-dioxatetracyclo[6.6.1. | C/C=C(/C(=O)O[C@H]1C[C@@H](OC(=O)C)[C@@]2([C@H]3C41CO[C@H]([C@H]4[C@@](C)([C@@H]([C@@H]3OC2)O)[C@@]12O[C@@]2(C)[C@@H]2CC1O[C@H]1[C@]2(O)C=CO1)O)C)C | 6442369 |
| IMPHY006799 | Meldenin | CC(=O)O[C@H]1[C@@H](O)[C@]2(C)[C@@H]([C@@]3([C@@H]1C(C)(C)C(=O)CC3)C)CC[C@@]1(C2=CC[C@H]1c1ccoc1)C | 101289833 |
| IMPHY006827 | (1S)-1-[(2R,4S,5R)-5-hydroxy-4-[(3S,5R,9R,10R,13S,14S,17S)-3-hydroxy-4,4,10,13,14-pentamethyl-2,3,5,6,9,11,12,15,16,17-decahydro-1H-cyclopenta[a]phenanthren-17-yl]oxolan-2-yl]-2-methylpropane-1,2-diol | O[C@@H]1O[C@H](C[C@H]1[C@@H]1CC[C@]2([C@@]1(C)CC[C@H]1C2=CC[C@@H]2[C@]1(C)CC[C@@H](C2(C)C)O)C)[C@@H](C(O)(C)C)O | 101650343 |
| IMPHY006853 | 2H-Cyclopenta(b)naphtho(2,3-d)furan-10-carboxylic acid,2-(3-furanyl)-3,3a,4a,5,5a,6,9,9a,10,10a-decahydro-5,6-dihydroxy-1,6,9a,10a-tetramethyl-9-oxo-, methyl ester, (2R,3aS,4aS,5R,5aS,6R,9aR,10S,10aR) | COC(=O)C[C@H]1[C@@]2(C)[C@H](O[C@@H]3C2=C(C)[C@@H](C3)c2cocc2)[C@@H]([C@@H]2[C@@]1(C)C(=O)C=C[C@@]2(C)O)O | 157277 |
| IMPHY006854 | (1R,2R,5R,6S,10R,11S,12R,15R,16R,18S,19R)-6-(furan-3-yl)-11,16,18-trihydroxy-1,5,10,15-tetramethyl-13-oxapentacyclo[10.6.1.02,10.05,9.015,19]nonadec-8-en-4-one | O[C@H]1C[C@@H](O)[C@@]2([C@H]3[C@@]1(C)[C@H]1CC(=O)[C@]4(C(=CC[C@H]4c4ccoc4)[C@]1(C)[C@@H]([C@@H]3OC2)O)C)C | 101306757 |
| IMPHY007327 | Palmitic acid | CCCCCCCCCCCCCCCC(=O)O | 985 |
| IMPHY008697 | 3,4-Dimethylthiophene | Cc1cscc1C | 79089 |
| IMPHY008937 | Vitamin E | C[C@@H](CCC[C@]1(C)CCc2c(O1)c(C)c(c(c2C)O)C)CCC[C@@H](CCCC(C)C)C | 14985 |
| IMPHY009514 | Salannol | COC(=O)C[C@@H]1[C@@]2(C)[C@@H](OC(=O)CC(C)C)C[C@H]([C@@]3([C@@H]2[C@H]([C@@H]2[C@@]1(C)C1=C(C)[C@@H](C[C@H]1O2)c1ccoc1)OC3)C)O | 12874816 |
| IMPHY009593 | Dipropyl disulfide | CCCSSCCC | 12377 |
| IMPHY009957 | 7-Deacetoxy-7-hydroxygedunin | O=C1OC(c2ccoc2)C2(C3(C1O3)C1(C)C(O)CC3C(C1CC2)(C)C=CC(=O)C3(C)C)C | 1885 |
| IMPHY010341 | 17-Hydroxyazadiradione | CC(=O)O[C@@H]1C[C@@H]2[C@]([C@@H]3[C@]1(C)C1=CC(=O)[C@@]([C@@]1(CC3)C)(O)c1ccoc1)(C)C=CC(=O)C2(C)C | 52951892 |
| IMPHY010454 | Vilasinin | O[C@H]1C[C@@H](O)[C@@]2([C@H]3[C@@]1(C)[C@H]1CC[C@@]4(C(=CC[C@H]4c4ccoc4)[C@]1(C)[C@@H]([C@@H]3OC2)O)C)C | 102090424 |
| IMPHY010554 | Salannin | COC(=O)C[C@@H]1[C@@]2(C)[C@@H](OC(=O)/C(=C/C)/C)C[C@H]([C@@]3([C@@H]2[C@H]([C@@H]2[C@@]1(C)C1=C(C)[C@@H](C[C@H]1O2)c1ccoc1)OC3)C)OC(=O)C | 6437066 |
| IMPHY010582 | 6-Deacetylnimbinene | COC(=O)C[C@H]1[C@@]2(C)[C@H](OC3C2=C(C)[C@@H](C3)c2cocc2)[C@@H](C2[C@]1(C)C(=O)CC=C2C)O | 102285347 |
| IMPHY010713 | Nimbinol | COC(=O)C[C@H]1[C@@]2(C)[C@H](O[C@H]3C2=C(C)[C@@H](C3)c2cocc2)[C@@H]([C@@H]2[C@]1(C)C(=O)C=C[C@@]2(C)CO)OC(=O)C | 21730094 |
| IMPHY011385 | 2-Methyl-2-pentenal | C/C(=CCC)/C=O | 5319754 |
| IMPHY011387 | Epoxyazadiradione | CC(=O)O[C@@H]1C[C@H]2C(C)(C)C(=O)C=C[C@@]2([C@@H]2[C@]1(C)[C@@]13O[C@@H]1C(=O)[C@H]([C@@]3(CC2)C)c1ccoc1)C | 49863985 |
| IMPHY011394 | Arachidic acid | CCCCCCCCCCCCCCCCCCCC(=O)O | 10467 |
| IMPHY011498 | 3-Deacetylsalannin | COC(=O)C[C@@H]1[C@@]2(C)[C@@H](OC(=O)/C(=C/C)/C)C[C@H]([C@@]3([C@@H]2[C@H]([C@@H]2[C@@]1(C)C1=C(C)[C@@H](C[C@H]1O2)c1ccoc1)OC3)C)O | 14458886 |
| IMPHY011526 | Compositolide | COC(=O)C[C@H]1[C@@]2(C)[C@H](O[C@H]3C2=C(C)[C@@H](C3)C2=CC(OC2=O)O)[C@H]2[C@@H]3[C@]1(C)[C@@H](OC(=O)/C(=C/C)/C)C[C@H]([C@@]3(C)CO2)OC(=O)C | 76309326 |
| IMPHY011547 | Salannic acid | OC(=O)C[C@H]1[C@@]2(C)[C@H](O[C@H]3C2=C(C)[C@@H](C3)c2cocc2)[C@H]2[C@@H]3[C@]1(C)[C@@H](O)C[C@H]([C@@]3(C)CO2)O | 185704 |
| IMPHY011797 | Oleic acid | CCCCCCCC/C=CCCCCCCCC(=O)O | 445639 |
| IMPHY012310 | Nimbin | COC(=O)C[C@H]1[C@@]2(C)[C@H](O[C@H]3C2=C(C)[C@@H](C3)c2cocc2)[C@@H]([C@@H]2[C@]1(C)C(=O)C=C[C@@]2(C)C(=O)OC)OC(=O)C | 108058 |
| IMPHY012436 | Vepinin | CC(=O)O[C@H]1[C@@H]2OC3C4C2(C)[C@@H]([C@@]2([C@@H]1C(C)(C)C(=O)C=C2)C)CC[C@]4([C@@H](C3)c1ccoc1)C | 185552 |
| IMPHY012918 | cis-3,5-Diethyl-1,2,4-trithiolane | CC[C@@H]1SS[C@@H](S1)CC | 6431294 |
| IMPHY014135 | Salannolactam 21 | COC(=O)CC1[C@@]2(C)[C@@H](OC(=O)/C(=C/C)/C)C[C@H]([C@@]3([C@@H]2[C@H]([C@@H]2[C@@]1(C)C1=C(C)[C@@H](C[C@H]1O2)C1=CCNC1=O)OC3)C)OC(=O)C | 6442937 |
| IMPHY014138 | Azadirachtin D | C/C=C(/C(=O)O[C@H]1C[C@@H](OC(=O)C)[C@@]2([C@H]3C41CO[C@]([C@H]4[C@@](C)([C@@H]([C@H]3OC2)O)[C@@]12O[C@@]2(C)[C@@H]2CC1O[C@H]1[C@]2(O)C=CO1)(O)C(=O)OC)C)C | 6443232 |
| IMPHY014139 | Azadirachtin F | COC(=O)[C@H]([C@H]1[C@@](C)([C@H](O)[C@H]2[C@@H]3[C@]1(C)[C@@H](O)C[C@H]([C@]3(CO2)C(=O)OC)OC(=O)/C(=C/C)/C)[C@@]12O[C@@]2(C)[C@@H]2C[C@H]1O[C@H]1[C@]2(O)C=CO1)O | 6443385 |
| IMPHY014597 | 13,14-Desepoxyazadirachtin A | C/C=C(/C(=O)O[C@H]1C[C@@H](OC(=O)C)[C@@]2([C@H]3[C@@]41CO[C@]([C@H]4[C@@](C)([C@@H]([C@@H]3OC2)O)C1=C(C)[C@@H]2C[C@@H]1O[C@H]1[C@@]2(O)C=CO1)(O)C(=O)OC)C(=O)OC)C | 101701146 |
| IMPHY014607 | 11-Hydroxyazadirachtin B | C/C=C(/C(=O)O[C@@H]1C[C@H](O)[C@]23[C@@H]4[C@@]1(CO[C@H]4[C@H]([C@@]([C@@H]3[C@@](OC2)(O)C(=O)OC)(C)[C@@]12O[C@@]2(C)[C@H]2C[C@@H]1O[C@H]1[C@]2(O)C=CO1)O)C(=O)OC)C | 101999884 |
| IMPHY014836 | beta-Sitosterol | CC[C@@H](C(C)C)CC[C@H]([C@H]1CC[C@@H]2[C@]1(C)CC[C@H]1[C@H]2CC=C2[C@]1(C)CC[C@@H](C2)O)C | 222284 |
| IMPHY014990 | Linoleic acid | CCCCC/C=CC/C=CCCCCCCCC(=O)O | 5280450 |
| IMPHY000155 | Azadirone | CC(=O)O[C@@H]1C[C@@H]2[C@]([C@@H]3[C@]1(C)C1=CC[C@H]([C@@]1(CC3)C)c1ccoc1)(C)C=CC(=O)C2(C)C | 10906239 |
| IMPHY002006 | Gedunin | CC(=O)O[C@@H]1C[C@H]2C(C)(C)C(=O)C=C[C@@]2([C@@H]2[C@]1(C)[C@@]13O[C@@H]1C(=O)O[C@H]([C@@]3(CC2)C)c1ccoc1)C | 12004512 |
| IMPHY006612 | Corosolic acid | C[C@@H]1CC[C@]2([C@@H]([C@H]1C)C1=CC[C@H]3[C@@]([C@@]1(CC2)C)(C)CC[C@@H]1[C@]3(C)C[C@@H](O)[C@@H](C1(C)C)O)C(=O)O | 6918774 |
| IMPHY011393 | 24-Methylenecycloartanol | C=C(C(C)C)CC[C@H]([C@H]1CC[C@@]2([C@]1(C)CC[C@@]13[C@H]2CC[C@@H]2[C@]3(C1)CC[C@@H](C2(C)C)O)C)C | 94204 |
| IMPHY012310 | Nimbin | COC(=O)C[C@H]1[C@@]2(C)[C@H](O[C@H]3C2=C(C)[C@@H](C3)c2cocc2)[C@@H]([C@@H]2[C@]1(C)C(=O)C=C[C@@]2(C)C(=O)OC)OC(=O)C | 108058 |
| IMPHY013913 | 7-(Acetyloxy)-21-hydroxy-6-methoxy-4,4,8-trimethyl-3-oxocarda-1,14,20(22)-trienolide | COC1C(OC(=O)C)C2(C)C3=CCC(C3(C)CCC2C2(C1C(C)(C)C(=O)C=C2)C)C1=CC(=O)OC1O | 188754 |
| IMPHY014836 | beta-Sitosterol | CC[C@@H](C(C)C)CC[C@H]([C@H]1CC[C@@H]2[C@]1(C)CC[C@H]1[C@H]2CC=C2[C@]1(C)CC[C@@H](C2)O)C | 222284 |
| IMPHY014893 | D-Glucose | OC[C@H]1OC(O)[C@@H]([C@H]([C@@H]1O)O)O | 5793 |
| IMPHY014916 | D-Fructose | OCC1(O)OC[C@H]([C@H]([C@@H]1O)O)O | 2723872 |
| IMPHY015116 | D-Xylose | O[C@@H]1COC([C@@H]([C@H]1O)O)O | 135191 |
| IMPHY000150 | 6-Deacetylnimbin | COC(=O)C[C@H]1[C@@]2(C)[C@H](O[C@H]3C2=C(C)[C@@H](C3)c2cocc2)[C@@H]([C@@H]2[C@]1(C)C(=O)C=C[C@@]2(C)C(=O)OC)O | 10505484 |
| IMPHY000472 | Isonimolicinolide | CC(=O)O[C@@H]1CC2C(C3C1(C)C1=CC(=O)[C@@]([C@@]1(CC3)C)(OC(=O)C)C1=CC(=O)OC1O)(C)C=CC(=O)C2(C)C | 0 |
| IMPHY005039 | 6-Acetylnimbandiol | COC(=O)C[C@H]1[C@@]2(C)[C@H](O[C@H]3C2=C(C)[C@@H](C3)c2cocc2)[C@@H]([C@@H]2[C@]1(C)C(=O)C=C[C@@]2(C)O)OC(=O)C | 52952216 |
| IMPHY006017 | Isomeldenin | CC(=O)OC1[C@H](O)[C@@H]2[C@]([C@@H]3[C@]1(C)C1=CC[C@H]([C@@]1(CC3)C)c1ccoc1)(C)CCC(=O)C2(C)C | 76316558 |
| IMPHY006551 | Isoscopoletin | COc1cc2oc(=O)ccc2cc1O | 69894 |
| IMPHY010224 | Nimocinol | CC(=O)O[C@@H]1[C@H](O)[C@@H]2[C@]([C@@H]3C1(C)C1=CC[C@H]([C@@]1(CC3)C)c1ccoc1)(C)C=CC(=O)C2(C)C | 178770 |
| IMPHY011518 | Esculetin | O=c1ccc2c(o1)cc(c(c2)O)O | 5281416 |
| IMPHY012402 | Campesterol | O[C@H]1CC[C@]2(C(=CC[C@@H]3[C@@H]2CC[C@]2([C@H]3CC[C@@H]2[C@@H](CC[C@H](C(C)C)C)C)C)C1)C | 173183 |
| IMPHY002310 | Melianin A | CC(=O)O[C@@H]1C[C@H]2C(C)(C)[C@H](OC(=O)c3ccccc3)C[C@@H]([C@@]2([C@@H]2[C@]1(C)C1=CC[C@H]([C@@]1(CC2)C)[C@H]1CO[C@H]([C@@H](C1)O)C(O)(C)C)C)OC(=O)C | 101277363 |
| IMPHY006231 | Nimbolin B | O=C(OC1C2OCC3(C2C(C2C1(C)C1=C(C)C(CC1OC(C2)O)c1cocc1)(C)C(CC3OC(=O)C)OC(=O)C)C)/C=C/c1ccccc1 | 6443005 |
| IMPHY006825 | melianin B | CC(=O)O[C@@H]1C[C@@H]2[C@@]([C@@H]3[C@]1(C)C1=CC[C@H]([C@@]1(CC3)C)C1COC([C@H]([C@@H](C1)O)O)(C)C)(C)[C@@H](OC(=O)C)C[C@H](C2(C)C)OC(=O)c1ccccc1 | 15427646 |
| IMPHY006834 | [(1R,2R,5R,6R,10R,11S,15R,16R,18S)-16,18-diacetyloxy-6-(furan-3-yl)-1,5,10,15-tetramethyl-13-oxapentacyclo[10.6.1.02,10.05,9.015,19]nonadec-8-en-11-yl] (E)-3-phenylprop-2-enoate | O=C(O[C@@H]1C2OC[C@]3(C2[C@]([C@@H]2[C@]1(C)C1=CC[C@H]([C@]1(CC2)C)c1cocc1)(C)[C@H](C[C@H]3OC(=O)C)OC(=O)C)C)/C=C/c1ccccc1 | 101650373 |
| IMPHY011393 | 24-Methylenecycloartanol | C=C(C(C)C)CC[C@H]([C@H]1CC[C@@]2([C@]1(C)CC[C@@]13[C@H]2CC[C@@H]2[C@]3(C1)CC[C@@H](C2(C)C)O)C)C | 94204 |
| IMPHY012769 | 24-Methylenelophenol | C=C(C(C)C)CC[C@H]([C@H]1CC[C@@H]2[C@]1(C)CC[C@H]1C2=CC[C@@H]2[C@]1(C)CC[C@@H]([C@H]2C)O)C | 5283640 |
| IMPHY014836 | beta-Sitosterol | CC[C@@H](C(C)C)CC[C@H]([C@H]1CC[C@@H]2[C@]1(C)CC[C@H]1[C@H]2CC=C2[C@]1(C)CC[C@@H](C2)O)C | 222284 |
| IMPHY000016 | Nimbidiol | O=C1C[C@H]2C(C)(C)CCC[C@@]2(c2c1cc(O)c(c2)O)C | 11334829 |
| IMPHY000093 | Nimbiol | O=C1C[C@H]2C(C)(C)CCC[C@@]2(c2c1cc(C)c(c2)O)C | 11119228 |
| IMPHY000150 | 6-Deacetylnimbin | COC(=O)C[C@H]1[C@@]2(C)[C@H](O[C@H]3C2=C(C)[C@@H](C3)c2cocc2)[C@@H]([C@@H]2[C@]1(C)C(=O)C=C[C@@]2(C)C(=O)OC)O | 10505484 |
| IMPHY000155 | Azadirone | CC(=O)O[C@@H]1C[C@@H]2[C@]([C@@H]3[C@]1(C)C1=CC[C@H]([C@@]1(CC3)C)c1ccoc1)(C)C=CC(=O)C2(C)C | 10906239 |
| IMPHY000165 | Tetracosanoic acid | CCCCCCCCCCCCCCCCCCCCCCCC(=O)O | 11197 |
| IMPHY000193 | Azadirachtanin | CC(=O)O[C@@H]1OC[C@@]23C([C@@]1(C)[C@H](OC(=O)C)C[C@@H]2O)C[C@H]([C@@]1([C@@H]3C(=O)[C@H](OC(=O)C)[C@@]2(C1=CC[C@H]2c1ccoc1)C)C)O | 102146586 |
| IMPHY000464 | Isomargosinolide | COC(=O)C[C@H]1[C@]2(C)C3=C(C)[C@@H](C[C@H]3O[C@@H]2[C@@H]2[C@@H]3[C@]1(C)C=CC(=O)[C@@]3(C)CO2)C1=CC(=O)OC1O | 0 |
| IMPHY000472 | Isonimolicinolide | CC(=O)O[C@@H]1CC2C(C3C1(C)C1=CC(=O)[C@@]([C@@]1(CC3)C)(OC(=O)C)C1=CC(=O)OC1O)(C)C=CC(=O)C2(C)C | 0 |
| IMPHY000663 | Methyl 2-[6-(furan-3-yl)-7,9,11,15-tetramethyl-12,16-dioxo-3,17-dioxapentacyclo[9.6.1.02,9.04,8.015,18]octadeca-7,13-dien-10-yl]acetate | COC(=O)CC1C2(C)C(OC3C2=C(C)C(C3)c2cocc2)C2C3C1(C)C(=O)C=CC3(C)C(=O)O2 | 100017 |
| IMPHY000668 | Nimbochalcin | CCOCc1c(CCC(=O)c2c(C)cc(cc2C(=O)OCC)O)c(O)c(c(c1O)O)O | 163114683 |
| IMPHY001121 | Azadirinin | CC(=O)O[C@@H]1C2C(C)(C)[C@H](OC(=O)C)C[C@@H]([C@]2(C)[C@@H]2[C@]([C@@H]1O)(C)C1=CC(=O)O[C@H]([C@@]1(CC2)C)c1ccoc1)OC(=O)/C=C/c1ccccc1 | 102275331 |
| IMPHY001127 | Desacetylnimbinolide | COC(=O)C[C@H]1[C@@]2(C)[C@H](O[C@H]3C2=C(C)[C@@H](C3)C2=CC(OC2=O)O)[C@@H]([C@@H]2[C@]1(C)C(=O)C=C[C@@]2(C)C(=O)OC)O | 102285346 |
| IMPHY001280 | Nimbinene | COC(=O)C[C@H]1[C@@]2(C)[C@H](OC3C2=C(C)[C@@H](C3)c2cocc2)[C@@H](C2[C@]1(C)C(=O)CC=C2C)OC(=O)C | 44715635 |
| IMPHY001415 | Azadirol | OC[C@H]([C@@H]1CC=C2[C@@]1(C)CC[C@H]1[C@@]2(C)[C@H](OC(=O)C)C[C@@H]2[C@]1(C)C=CC(=O)C2(C)C)C[C@@H](C(=O)C(O)(C)C)O | 44567142 |
| IMPHY001446 | Kulinone | CC(=CCC[C@H]([C@@H]1[C@@H](O)C[C@]2([C@@]1(C)CC[C@H]1C2=CC[C@@H]2[C@]1(C)CCC(=O)C2(C)C)C)C)C | 44567124 |
| IMPHY001448 | Methyl kulonate | COC(=O)[C@@H]([C@@H]1[C@@H](O)C[C@]2([C@@]1(C)CC[C@H]1C2=CC[C@@H]2[C@]1(C)CCC(=O)C2(C)C)C)CCC=C(C)C | 44567123 |
| IMPHY001600 | Kulactone | CC(=CCC[C@H]1C(=O)O[C@@H]2[C@@H]1[C@]1(C)CC[C@H]3C(=CC[C@@H]4[C@]3(C)CCC(=O)C4(C)C)[C@]1(C2)C)C | 15560423 |
| IMPHY002006 | Gedunin | CC(=O)O[C@@H]1C[C@H]2C(C)(C)C(=O)C=C[C@@]2([C@@H]2[C@]1(C)[C@@]13O[C@@H]1C(=O)O[C@H]([C@@]3(CC2)C)c1ccoc1)C | 12004512 |
| IMPHY002050 | Campest-4-en-3-one | CC([C@@H](CC[C@H]([C@H]1CC[C@@H]2[C@]1(C)CC[C@H]1[C@H]2CCC2=CC(=O)CC[C@]12C)C)C)C | 11988279 |
| IMPHY002171 | Kulolactone | CC(=CCC[C@H]1C(=O)O[C@@H]2[C@@H]1[C@]1(C)CC[C@H]3C(=CC[C@@H]4[C@]3(C)CC[C@H](C4(C)C)O)[C@]1(C2)C)C | 101289844 |
| IMPHY002229 | Cycloeucalenol | C=C(C(C)C)CC[C@H]([C@H]1CC[C@@]2([C@]1(C)CC[C@@]13[C@H]2CC[C@@H]2[C@]3(C1)CC[C@@H]([C@H]2C)O)C)C | 101690 |
| IMPHY002244 | Isoazadirolide | COC(=O)CC1C2(C)C(OC3C2=C(C)C(C3)C2=CC(=O)OC2O)C2C3C1(C)C(OC(=O)C=C(C)C)CC(C3(C)CO2)O | 101425842 |
| IMPHY002557 | Nimbolicin | OC1OC2CC(C(=C2C2(C(C1)C1(C)C(OC(=O)C(=C)C)CC(C3(C1C(C2OC(=O)/C=C/c1ccccc1)OC3)C)OC(=O)C)C)C)c1ccoc1 | 14563366 |
| IMPHY002620 | Nimbanal | COC(=O)C[C@H]1[C@@]2(C)[C@H](O[C@H]3C2=C(C)[C@@H](C3)c2cocc2)[C@@H]([C@@H]2[C@]1(C)C(=O)C=C[C@@]2(C)C=O)OC(=O)C | 14194023 |
| IMPHY002653 | Nimbocinol | O=C1C=C2[C@@]([C@@H]1c1ccoc1)(C)CC[C@H]1[C@@]2(C)[C@H](O)C[C@@H]2[C@]1(C)C=CC(=O)C2(C)C | 13875741 |
| IMPHY002654 | Nimbocinolide | CC(=O)O[C@@H]1C[C@@H]2[C@]([C@@H]3[C@]1(C)C1=CC[C@H]([C@@]1([C@H]([C@H]3OC(=O)C(O)(C)C)O)C)C1=CC(OC1=O)O)(C)C=CC(=O)C2(C)C | 13875774 |
| IMPHY002788 | 5,7-Dihydroxy-4'-methoxy-8,3'-di-C-prenylflavanone | COc1ccc(cc1CC=C(C)C)C1CC(=O)c2c(O1)c(CC=C(C)C)c(cc2O)O | 14492795 |
| IMPHY002857 | 28-Deoxonimbolide | COC(=O)C[C@H]1[C@@]2(C)[C@H](O[C@H]3C2=C(C)[C@@H](C3)c2cocc2)[C@H]2[C@@H]3[C@]1(C)C(=O)C=C[C@@]3(C)CO2 | 14467538 |
| IMPHY002896 | 1,2-Dioleoyl-3-palmitoylglycerol | CCCCCCCC/C=CCCCCCCCC(=O)OCC(OC(=O)CCCCCCC/C=CCCCCCCCC)COC(=O)CCCCCCCCCCCCCCC | 25240174 |
| IMPHY002915 | Benzyl Alcohol | OCc1ccccc1 | 244 |
| IMPHY002935 | 1-Nonacosanol | CCCCCCCCCCCCCCCCCCCCCCCCCCCCCO | 243696 |
| IMPHY003099 | N-[[5,8-dimethoxy-6-nitro-2-(trifluoromethyl)quinolin-4-yl]methylidene]hydroxylamine | ON=Cc1cc(nc2c1c(OC)c(cc2OC)[N+](=O)[O-])C(F)(F)F | 298061 |
| IMPHY003230 | Galactoarabinan | COC1C(O)C(COC2OCC(C(C2O)OC)O)OC(C1O)OC1C(O)C(C)OC(C1O)CO | 24847856 |
| IMPHY003237 | 2,4-Dimethylthiophene | Cc1scc(c1)C | 34296 |
| IMPHY003342 | Nimolinin | O=C1C[C@@]2(O)C(C)(C)CCC[C@@]2(c2c1cc(C(C)C)c(c2)O)C | 180429 |
| IMPHY003804 | Cycloeucalenone | C=C(C(C)C)CC[C@H]([C@H]1CC[C@@]2([C@]1(C)CC[C@@]13[C@H]2CC[C@@H]2[C@]3(C1)CCC(=O)[C@H]2C)C)C | 21594790 |
| IMPHY003824 | 17-Epiazadiradione | CC(=O)O[C@@H]1C[C@@H]2[C@]([C@@H]3[C@]1(C)C1=CC(=O)[C@@H]([C@@]1(CC3)C)c1ccoc1)(C)C=CC(=O)C2(C)C | 12308716 |
| IMPHY003825 | Azadiradione | CC(=O)O[C@@H]1C[C@@H]2[C@]([C@@H]3[C@]1(C)C1=CC(=O)[C@H]([C@@]1(CC3)C)c1ccoc1)(C)C=CC(=O)C2(C)C | 12308714 |
| IMPHY003959 | Vepaol | CO[C@H]1O[C@@H]2[C@@](C1)(O)[C@@H]1C[C@H](O2)[C@@]2([C@@]1(C)O2)[C@]1(C)[C@H](O)[C@@H]2OC[C@]3([C@H]2[C@@]2([C@H]1[C@](O)(OC2)C(=O)OC)[C@@H](OC(=O)/C(=C/C)/C)C[C@H]3OC(=O)C)C(=O)OC | 21725519 |
| IMPHY003960 | 23-Epivepaol | CO[C@@H]1O[C@@H]2[C@@](C1)(O)[C@@H]1C[C@H](O2)[C@@]2([C@@]1(C)O2)[C@]1(C)[C@H](O)[C@@H]2OC[C@]3([C@H]2[C@@]2([C@H]1[C@](O)(OC2)C(=O)OC)[C@@H](OC(=O)/C(=C/C)/C)C[C@H]3OC(=O)C)C(=O)OC | 24796982 |
| IMPHY003979 | beta-Sitostenone | CC[C@@H](C(C)C)CC[C@H](C1CCC2[C@]1(C)CCC1C2CCC2=CC(=O)CC[C@]12C)C | 60123241 |
| IMPHY004014 | Azadirachtin | C/C=C(/C(=O)O[C@H]1C[C@@H](OC(=O)C)[C@@]2([C@H]3[C@@]41CO[C@]([C@H]4[C@@](C)([C@@H]([C@@H]3OC2)O)[C@@]12O[C@@]2(C)[C@H]2C[C@@H]1O[C@H]1[C@]2(O)C=CO1)(O)C(=O)OC)C(=O)OC)C | 5281303 |
| IMPHY004015 | dimethyl (1S,4S,5R,6S,7S,8R,11S,12R,14S,15R)-12-acetyloxy-4,7-dihydroxy-6-[(1R,2S,6S,8R,9R,11S)-2-hydroxy-11-methyl-5,7,10-trioxatetracyclo[6.3.1.02,6.09,11]dodec-3-en-9-yl]-6-methyl-14-[(E)-2-methylb | C/C=C(/C(=O)O[C@H]1C[C@@H](OC(=O)C)[C@@]2([C@H]3[C@@]41CO[C@]([C@H]4[C@@](C)([C@@H]([C@@H]3OC2)O)[C@@]12O[C@@]2(C)[C@@H]2C[C@H]1O[C@H]1[C@]2(O)C=CO1)(O)C(=O)OC)C(=O)OC)C | 44584063 |
| IMPHY004024 | dimethyl (2aR,3S,4S,4aR,5S,7aS,8R,10R,10aS,10bR)-10-acetoxy-3,5-dihydroxy-4-[(1aR,2S,3aS,6aS,7S,7aS)-6a-hydroxy-7a-methyl-3a,6a,7,7a-tetrahydro-2,7-methanofuro[2,3-b]oxireno[e]oxepin-1a(2H)-yl]-4-meth | C/C=C(/C(=O)O[C@@H]1C[C@@H](OC(=O)C)[C@@]2([C@H]3[C@@]41CO[C@]([C@H]4[C@@](C)([C@@H]([C@@H]3OC2)O)[C@@]12O[C@@]2(C)[C@H]2C[C@@H]1O[C@H]1[C@]2(O)C=CO1)(O)C(=O)OC)C(=O)OC)C | 16126804 |
| IMPHY004079 | Catechol | Oc1ccccc1O | 289 |
| IMPHY004140 | 7-Deacetoxy-7-oxogedunin | O=C1O[C@@H](c2ccoc2)[C@]2([C@]3([C@@H]1O3)[C@]1(C)C(=O)C[C@@H]3[C@](C1CC2)(C)C=CC(=O)C3(C)C)C | 71300386 |
| IMPHY004235 | D-Glucuronic Acid | OC1O[C@H](C(=O)O)[C@H]([C@@H]([C@H]1O)O)O | 94715 |
| IMPHY004388 | Kaempferol | Oc1ccc(cc1)c1oc2cc(O)cc(c2c(=O)c1O)O | 5280863 |
| IMPHY004619 | Quercetin | Oc1cc(O)c2c(c1)oc(c(c2=O)O)c1ccc(c(c1)O)O | 5280343 |
| IMPHY004631 | Stearic acid | CCCCCCCCCCCCCCCCCC(=O)O | 5281 |
| IMPHY005022 | Isofraxidin | COc1c(O)c(OC)cc2c1oc(=O)cc2 | 5318565 |
| IMPHY005038 | 7-Deacetyl-7-benzoylgedunin | O=C(c1ccccc1)O[C@@H]1C[C@H]2C(C)(C)C(=O)C=C[C@@]2([C@@H]2[C@]1(C)[C@@]13O[C@@H]1C(=O)O[C@H]([C@@]3(CC2)C)c1ccoc1)C | 52952112 |
| IMPHY005039 | 6-Acetylnimbandiol | COC(=O)C[C@H]1[C@@]2(C)[C@H](O[C@H]3C2=C(C)[C@@H](C3)c2cocc2)[C@@H]([C@@H]2[C@]1(C)C(=O)C=C[C@@]2(C)O)OC(=O)C | 52952216 |
| IMPHY005199 | Azadirachtol | COC(=O)[C@H]1OC[C@]23[C@@H]1[C@@](C)([C@@H]([C@H]1[C@H]3[C@@]([C@@H](C[C@@H]2O)O)(CO1)C(=O)OC)O)[C@@]12O[C@@]2(C)[C@H]2C[C@@H]1O[C@H]1[C@]2(O)C=CO1 | 23256847 |
| IMPHY005223 | Deacetylazadirachtinol | COC(=O)[C@H]1OC[C@]23[C@@H]1[C@@](C)([C@H](O)[C@H]1[C@H]3[C@@]([C@@H](C[C@@H]2O)OC(=O)/C(=C/C)/C)(CO1)C(=O)OC)[C@@]12O[C@@]2(C)[C@H]2C[C@@H]1O[C@H]1[C@]2(O)C=CO1 | 21725521 |
| IMPHY005267 | (9R,10R,13S,14S,17R)-17-[(2R)-2-[(1R,2S)-1,3-dihydroxy-2-methylpropyl]-2,3-dihydrofuran-4-yl]-4,4,10,13,14-pentamethyl-1,2,5,6,9,11,12,15,16,17-decahydrocyclopenta[a]phenanthren-3-one | OC[C@@H]([C@H]([C@@H]1OC=C(C1)[C@@H]1CC[C@]2([C@@]1(C)CC[C@H]1C2=CCC2[C@]1(C)CCC(=O)C2(C)C)C)O)C | 184503 |
| IMPHY005274 | [(5R,6R,7S,9R,10R,13S,17R)-6-hydroxy-17-(2-hydroxy-5-oxo-2H-furan-3-yl)-4,4,8,10,13-pentamethyl-3-oxo-5,6,7,9,11,12,16,17-octahydrocyclopenta[a]phenanthren-7-yl] acetate | CC(=O)O[C@@H]1[C@H](O)[C@@H]2[C@]([C@@H]3C1(C)C1=CC[C@H]([C@]1(C)CC3)C1=CC(=O)OC1O)(C)C=CC(=O)C2(C)C | 184310 |
| IMPHY005293 | Nimolicinol | CC(=O)O[C@@H]1C[C@@H]2[C@]([C@@H]3[C@]1(C)C1=CC(=O)O[C@]([C@@]1(CC3)C)(O)c1ccoc1)(C)C=CC(=O)C2(C)C | 184937 |
| IMPHY005303 | (4bS,8aR)-2,4b,8,8-tetramethyl-7,10-dioxo-5,6,8a,9-tetrahydrophenanthrene-3-carboxylic Acid | O=C1CC[C@]2([C@H](C1(C)C)CC(=O)c1c2cc(C(=O)O)c(c1)C)C | 189727 |
| IMPHY005310 | (4aS,10aR)-7-hydroxy-1,1,4a,6-tetramethyl-3,4,10,10a-tetrahydrophenanthrene-2,9-dione | O=C1CC[C@]2([C@H](C1(C)C)CC(=O)c1c2cc(C)c(c1)O)C | 189404 |
| IMPHY005328 | (4aS,10aR)-6-hydroxy-1,1,4a,7-tetramethyl-3,4,10,10a-tetrahydrophenanthrene-2,9-dione | O=C1CC[C@]2([C@H](C1(C)C)CC(=O)c1c2cc(O)c(c1)C)C | 189403 |
| IMPHY005337 | Nimbionol | COc1cc2C(=O)C[C@@H]3[C@](c2cc1O)(C)CC[C@@H](C3(C)C)O | 189704 |
| IMPHY005471 | Myricetin | Oc1cc(O)c2c(c1)oc(c(c2=O)O)c1cc(O)c(c(c1)O)O | 5281672 |
| IMPHY005900 | Dipropyl trisulfide | CCCSSSCCC | 22383 |
| IMPHY005957 | Nimolinone | C=C[C@@H]1OC(=O)[C@@H](C1)[C@@H]1CC[C@]2([C@@]1(C)CC=C1C2=CC[C@@H]2[C@]1(C)CCC(=O)C2(C)C)C | 56841069 |
| IMPHY005975 | 11beta-Azadirachtin H | C/C=C(/C(=O)O[C@H]1C[C@@H](OC(=O)C)[C@@]2([C@H]3[C@@]41CO[C@H]([C@H]4[C@@](C)([C@@H]([C@@H]3OC2)O)[C@@]12O[C@@]2(C)[C@H]2C[C@@H]1O[C@H]1[C@]2(O)C=CO1)O)C(=O)OC)C | 76327056 |
| IMPHY005977 | Campestan-3-one | O=C1CC[C@]2(C(C1)CC[C@@H]1[C@@H]2CC[C@]2([C@H]1CC[C@@H]2[C@@H](CC[C@H](C(C)C)C)C)C)C | 53754390 |
| IMPHY006017 | Isomeldenin | CC(=O)OC1[C@H](O)[C@@H]2[C@]([C@@H]3[C@]1(C)C1=CC[C@H]([C@@]1(CC3)C)c1ccoc1)(C)CCC(=O)C2(C)C | 76316558 |
| IMPHY006063 | 1-Docosene | CCCCCCCCCCCCCCCCCCCCC=C | 74138 |
| IMPHY006221 | Tiglic acid | C/C(=CC)/C(=O)O | 125468 |
| IMPHY006231 | Nimbolin B | O=C(OC1C2OCC3(C2C(C2C1(C)C1=C(C)C(CC1OC(C2)O)c1cocc1)(C)C(CC3OC(=O)C)OC(=O)C)C)/C=C/c1ccccc1 | 6443005 |
| IMPHY006238 | [(4S,5R,6R,11R,12R,15S,16R,17Z)-17-(2,2-dihydroxyethylidene)-5-hydroxy-3,7,7,11,15-pentamethyl-8,18-dioxo-19-oxapentacyclo[14.3.1.02,15.03,12.06,11]icosa-1,9-dien-4-yl] acetate | CC(=O)O[C@@H]1[C@H](O)[C@@H]2[C@]([C@@H]3C1(C)C1=C4OC(=O)/C(=CC(O)O)/[C@@H]([C@@]1(CC3)C)C4)(C)C=CC(=O)C2(C)C | 6442906 |
| IMPHY006286 | [(4R,5R,6S,7S,8R,11R,12R,14S,15S)-12-acetyloxy-4,7-dihydroxy-6-[(1R,2S,6S,9R,11S)-2-hydroxy-11-methyl-5,7,10-trioxatetracyclo[6.3.1.02,6.09,11]dodec-3-en-9-yl]-6,11-dimethyl-3,9-dioxatetracyclo[6.6.1. | C/C=C(/C(=O)O[C@H]1C[C@@H](OC(=O)C)[C@@]2([C@H]3C41CO[C@H]([C@H]4[C@@](C)([C@@H]([C@@H]3OC2)O)[C@@]12O[C@@]2(C)[C@@H]2CC1O[C@H]1[C@]2(O)C=CO1)O)C)C | 6442369 |
| IMPHY006300 | Cholesterol | CC(CCC[C@H]([C@H]1CC[C@@H]2[C@]1(C)CC[C@H]1[C@H]2CC=C2[C@]1(C)CC[C@@H](C2)O)C)C | 5997 |
| IMPHY006551 | Isoscopoletin | COc1cc2oc(=O)ccc2cc1O | 69894 |
| IMPHY006652 | Norvaline | CCC[C@@H](C(=O)O)N | 65908 |
| IMPHY006659 | Obtusifoliol | CC(C(=C)CC[C@H]([C@H]1CC[C@@]2([C@]1(C)CCC1=C2CC[C@@H]2[C@]1(C)CC[C@@H]([C@H]2C)O)C)C)C | 65252 |
| IMPHY006786 | Margosinolide | COC(=O)C[C@H]1[C@@]2(C)[C@H](O[C@H]3C2=C(C)[C@@H](C3)C2=CC(OC2=O)O)[C@H]2[C@@H]3[C@]1(C)C=CC(=O)[C@@]3(C)CO2 | 21681049 |
| IMPHY006799 | Meldenin | CC(=O)O[C@H]1[C@@H](O)[C@]2(C)[C@@H]([C@@]3([C@@H]1C(C)(C)C(=O)CC3)C)CC[C@@]1(C2=CC[C@H]1c1ccoc1)C | 101289833 |
| IMPHY006827 | (1S)-1-[(2R,4S,5R)-5-hydroxy-4-[(3S,5R,9R,10R,13S,14S,17S)-3-hydroxy-4,4,10,13,14-pentamethyl-2,3,5,6,9,11,12,15,16,17-decahydro-1H-cyclopenta[a]phenanthren-17-yl]oxolan-2-yl]-2-methylpropane-1,2-diol | O[C@@H]1O[C@H](C[C@H]1[C@@H]1CC[C@]2([C@@]1(C)CC[C@H]1C2=CC[C@@H]2[C@]1(C)CC[C@@H](C2(C)C)O)C)[C@@H](C(O)(C)C)O | 101650343 |
| IMPHY006834 | [(1R,2R,5R,6R,10R,11S,15R,16R,18S)-16,18-diacetyloxy-6-(furan-3-yl)-1,5,10,15-tetramethyl-13-oxapentacyclo[10.6.1.02,10.05,9.015,19]nonadec-8-en-11-yl] (E)-3-phenylprop-2-enoate | O=C(O[C@@H]1C2OC[C@]3(C2[C@]([C@@H]2[C@]1(C)C1=CC[C@H]([C@]1(CC2)C)c1cocc1)(C)[C@H](C[C@H]3OC(=O)C)OC(=O)C)C)/C=C/c1ccccc1 | 101650373 |
| IMPHY006837 | (5R,9R,10R,13S,14S,17S)-17-[(2R,3S,5R)-5-[(2S)-3,3-dimethyloxiran-2-yl]-2-hydroxyoxolan-3-yl]-4,4,10,13,14-pentamethyl-1,2,5,6,9,11,12,15,16,17-decahydrocyclopenta[a]phenanthren-3-one | O[C@@H]1O[C@H](C[C@H]1[C@@H]1CC[C@]2([C@@]1(C)CC[C@H]1C2=CC[C@@H]2[C@]1(C)CCC(=O)C2(C)C)C)[C@@H]1OC1(C)C | 44575793 |
| IMPHY006853 | 2H-Cyclopenta(b)naphtho(2,3-d)furan-10-carboxylic acid,2-(3-furanyl)-3,3a,4a,5,5a,6,9,9a,10,10a-decahydro-5,6-dihydroxy-1,6,9a,10a-tetramethyl-9-oxo-, methyl ester, (2R,3aS,4aS,5R,5aS,6R,9aR,10S,10aR) | COC(=O)C[C@H]1[C@@]2(C)[C@H](O[C@@H]3C2=C(C)[C@@H](C3)c2cocc2)[C@@H]([C@@H]2[C@@]1(C)C(=O)C=C[C@@]2(C)O)O | 157277 |
| IMPHY006854 | (1R,2R,5R,6S,10R,11S,12R,15R,16R,18S,19R)-6-(furan-3-yl)-11,16,18-trihydroxy-1,5,10,15-tetramethyl-13-oxapentacyclo[10.6.1.02,10.05,9.015,19]nonadec-8-en-4-one | O[C@H]1C[C@@H](O)[C@@]2([C@H]3[C@@]1(C)[C@H]1CC(=O)[C@]4(C(=CC[C@H]4c4ccoc4)[C@]1(C)[C@@H]([C@@H]3OC2)O)C)C | 101306757 |
| IMPHY007084 | Vanillic acid | COc1cc(ccc1O)C(=O)O | 8468 |
| IMPHY007203 | 1-Pentanethiol | CCCCCS | 8067 |
| IMPHY007212 | Docosanoic acid | CCCCCCCCCCCCCCCCCCCCCC(=O)O | 8215 |
| IMPHY007273 | 1-Hexacosanol | CCCCCCCCCCCCCCCCCCCCCCCCCCO | 68171 |
| IMPHY007327 | Palmitic acid | CCCCCCCCCCCCCCCC(=O)O | 985 |
| IMPHY007375 | Sugiol | O=C1C[C@H]2C(C)(C)CCC[C@@]2(c2c1cc(C(C)C)c(c2)O)C | 94162 |
| IMPHY007385 | Nicotinamide | NC(=O)c1cccnc1 | 936 |
| IMPHY007392 | 2',3'-Dehydrosalannol | COC(=O)C[C@@H]1[C@@]2(C)[C@@H](OC(=O)C=C(C)C)C[C@H]([C@@]3([C@@H]2[C@H]([C@@H]2[C@@]1(C)C1=C(C)[C@@H](C[C@H]1O2)c1ccoc1)OC3)C)O | 91886694 |
| IMPHY008412 | Limbonin | CO/C=C(/C1OC(=O)C=C2C1(C)CCC1C2(C)C(O)C2C3C1(C)C(OC(=O)/C(=C/C)/C)CC(C3(CO2)CO)OC(=O)C)CC(C(=O)O)O | 102276189 |
| IMPHY008697 | 3,4-Dimethylthiophene | Cc1cscc1C | 79089 |
| IMPHY008724 | Isorhamnetin | COc1cc(ccc1O)c1oc2cc(O)cc(c2c(=O)c1O)O | 5281654 |
| IMPHY009353 | Mahmoodin | OCCO[C@]1(OC(=O)C=C2[C@@]1(C)CC[C@H]1[C@@]2(C)[C@H](OC(=O)C)C[C@@H]2[C@]1(C)C=CC(=O)C2(C)C)c1cocc1 | 126566 |
| IMPHY009373 | Fraxinellone | CC1=C2C(=O)O[C@H]([C@@]2(CCC1)C)c1cocc1 | 124039 |
| IMPHY009375 | Docosane | CCCCCCCCCCCCCCCCCCCCCC | 12405 |
| IMPHY009443 | Nimbionone | COc1cc2C(=O)C[C@@H]3[C@](c2cc1O)(C)CCC(=O)C3(C)C | 189706 |
| IMPHY009445 | 4alpha-Methylfecosterol | C=C(C(C)C)CC[C@H]([C@H]1CC[C@@H]2[C@]1(C)CCC1=C2CC[C@@H]2[C@]1(C)CC[C@@H]([C@H]2C)O)C | 193524 |
| IMPHY009514 | Salannol | COC(=O)C[C@@H]1[C@@]2(C)[C@@H](OC(=O)CC(C)C)C[C@H]([C@@]3([C@@H]2[C@H]([C@@H]2[C@@]1(C)C1=C(C)[C@@H](C[C@H]1O2)c1ccoc1)OC3)C)O | 12874816 |
| IMPHY009552 | Naheedin | CCC([C@H]1O[C@@H]([C@H](C1)[C@@H]1CC=C2[C@@]1(C)C[C@@H](O)[C@H]1[C@@]2(C)[C@H](OC(=O)C)C[C@@H]2[C@]1(C)C=CC(=O)C2(C)C)O)C | 129754 |
| IMPHY009593 | Dipropyl disulfide | CCCSSCCC | 12377 |
| IMPHY009642 | 2-Nonanone | CCCCCCCC(=O)C | 13187 |
| IMPHY009726 | Nimbilin | C/C=C(/C(=O)OC1CC(OC(=O)C)C2(C3C1(C)C1CC(O)OC4C(=C(C)C(C4)c4ccoc4)C1(C)C(C3OC2)OC(=O)/C=C/c1ccccc1)C)C | 6442484 |
| IMPHY009957 | 7-Deacetoxy-7-hydroxygedunin | O=C1OC(c2ccoc2)C2(C3(C1O3)C1(C)C(O)CC3C(C1CC2)(C)C=CC(=O)C3(C)C)C | 1885 |
| IMPHY010224 | Nimocinol | CC(=O)O[C@@H]1[C@H](O)[C@@H]2[C@]([C@@H]3C1(C)C1=CC[C@H]([C@@]1(CC3)C)c1ccoc1)(C)C=CC(=O)C2(C)C | 178770 |
| IMPHY010313 | 17-epi-17-Hydroxyazadiradione | CC(=O)O[C@@H]1C[C@@H]2[C@]([C@@H]3[C@]1(C)C1=CC(=O)[C@]([C@@]1(CC3)C)(O)c1ccoc1)(C)C=CC(=O)C2(C)C | 52951894 |
| IMPHY010341 | 17-Hydroxyazadiradione | CC(=O)O[C@@H]1C[C@@H]2[C@]([C@@H]3[C@]1(C)C1=CC(=O)[C@@]([C@@]1(CC3)C)(O)c1ccoc1)(C)C=CC(=O)C2(C)C | 52951892 |
| IMPHY010454 | Vilasinin | O[C@H]1C[C@@H](O)[C@@]2([C@H]3[C@@]1(C)[C@H]1CC[C@@]4(C(=CC[C@H]4c4ccoc4)[C@]1(C)[C@@H]([C@@H]3OC2)O)C)C | 102090424 |
| IMPHY010554 | Salannin | COC(=O)C[C@@H]1[C@@]2(C)[C@@H](OC(=O)/C(=C/C)/C)C[C@H]([C@@]3([C@@H]2[C@H]([C@@H]2[C@@]1(C)C1=C(C)[C@@H](C[C@H]1O2)c1ccoc1)OC3)C)OC(=O)C | 6437066 |
| IMPHY010582 | 6-Deacetylnimbinene | COC(=O)C[C@H]1[C@@]2(C)[C@H](OC3C2=C(C)[C@@H](C3)c2cocc2)[C@@H](C2[C@]1(C)C(=O)CC=C2C)O | 102285347 |
| IMPHY010637 | Melicitrin | Oc1cc(O)c2c(c1)oc(c(c2=O)O)c1cc(O[C@@H]2OC[C@@H]([C@@H]([C@H]2O)O)O)c(c(c1)O)O | 5319346 |
| IMPHY010713 | Nimbinol | COC(=O)C[C@H]1[C@@]2(C)[C@H](O[C@H]3C2=C(C)[C@@H](C3)c2cocc2)[C@@H]([C@@H]2[C@]1(C)C(=O)C=C[C@@]2(C)CO)OC(=O)C | 21730094 |
| IMPHY010893 | Trichilin H | CC(=O)O[C@@H]1[C@H](O)[C@]23COC([C@]([C@@H]1OC(=O)C)([C@@H]3C[C@H]([C@@]1([C@@H]2C(=O)[C@H](OC(=O)C)[C@@]2([C@]31O[C@@H]3C[C@H]2c1ccoc1)C)C)O)C)OC(=O)C(C)C | 15966088 |
| IMPHY011314 | 24-Methylenecycloartanone | C=C(C(C)C)CC[C@H]([C@H]1CC[C@@]2([C@]1(C)CC[C@@]13[C@H]2CC[C@@H]2[C@]3(C1)CCC(=O)C2(C)C)C)C | 14635659 |
| IMPHY011385 | 2-Methyl-2-pentenal | C/C(=CCC)/C=O | 5319754 |
| IMPHY011387 | Epoxyazadiradione | CC(=O)O[C@@H]1C[C@H]2C(C)(C)C(=O)C=C[C@@]2([C@@H]2[C@]1(C)[C@@]13O[C@@H]1C(=O)[C@H]([C@@]3(CC2)C)c1ccoc1)C | 49863985 |
| IMPHY011393 | 24-Methylenecycloartanol | C=C(C(C)C)CC[C@H]([C@H]1CC[C@@]2([C@]1(C)CC[C@@]13[C@H]2CC[C@@H]2[C@]3(C1)CC[C@@H](C2(C)C)O)C)C | 94204 |
| IMPHY011394 | Arachidic acid | CCCCCCCCCCCCCCCCCCCC(=O)O | 10467 |
| IMPHY011498 | 3-Deacetylsalannin | COC(=O)C[C@@H]1[C@@]2(C)[C@@H](OC(=O)/C(=C/C)/C)C[C@H]([C@@]3([C@@H]2[C@H]([C@@H]2[C@@]1(C)C1=C(C)[C@@H](C[C@H]1O2)c1ccoc1)OC3)C)O | 14458886 |
| IMPHY011518 | Esculetin | O=c1ccc2c(o1)cc(c(c2)O)O | 5281416 |
| IMPHY011521 | 2-Undecanone | CCCCCCCCCC(=O)C | 8163 |
| IMPHY011526 | Compositolide | COC(=O)C[C@H]1[C@@]2(C)[C@H](O[C@H]3C2=C(C)[C@@H](C3)C2=CC(OC2=O)O)[C@H]2[C@@H]3[C@]1(C)[C@@H](OC(=O)/C(=C/C)/C)C[C@H]([C@@]3(C)CO2)OC(=O)C | 76309326 |
| IMPHY011541 | Scopoletin | COc1cc2ccc(=O)oc2cc1O | 5280460 |
| IMPHY011547 | Salannic acid | OC(=O)C[C@H]1[C@@]2(C)[C@H](O[C@H]3C2=C(C)[C@@H](C3)c2cocc2)[C@H]2[C@@H]3[C@]1(C)[C@@H](O)C[C@H]([C@@]3(C)CO2)O | 185704 |
| IMPHY011707 | beta-Carotene | C/C(=CC=CC=C(C=CC=C(C=CC1=C(C)CCCC1(C)C)/C)/C)/C=C/C=C(/C=C/C1=C(C)CCCC1(C)C)C | 5280489 |
| IMPHY011735 | (+)-Gallocatechin | Oc1cc2O[C@H](c3cc(O)c(c(c3)O)O)[C@H](Cc2c(c1)O)O | 65084 |
| IMPHY011741 | Tannic acid | O=C(c1cc(O)c(c(c1)OC(=O)c1cc(O)c(c(c1)O)O)O)O[C@@H]1[C@@H](COC(=O)c2cc(O)c(c(c2)OC(=O)c2cc(O)c(c(c2)O)O)O)O[C@H]([C@@H]([C@H]1OC(=O)c1cc(O)c(c(c1)OC(=O)c1cc(O)c(c(c1)O)O)O)OC(=O)c1cc(O)c(c(c1)OC(=O)c1cc(O)c(c(c1)O)O)O)OC(=O)c1cc(O)c(c(c1)OC(=O)c1cc(O)c(c(c1)O)O)O | 16129778 |
| IMPHY011797 | Oleic acid | CCCCCCCC/C=CCCCCCCCC(=O)O | 445639 |
| IMPHY011844 | Chlorogenic acid | O=C(O[C@@H]1C[C@@](O)(C[C@H]([C@H]1O)O)C(=O)O)/C=C/c1ccc(c(c1)O)O | 1794427 |
| IMPHY011960 | Cinnamic acid | OC(=O)/C=C/c1ccccc1 | 444539 |
| IMPHY011985 | Nicotiflorin | Oc1ccc(cc1)c1oc2cc(O)cc(c2c(=O)c1O[C@@H]1O[C@H](CO[C@@H]2O[C@@H](C)[C@@H]([C@H]([C@H]2O)O)O)[C@H]([C@@H]([C@H]1O)O)O)O | 5318767 |
| IMPHY012021 | Gallic acid | OC(=O)c1cc(O)c(c(c1)O)O | 370 |
| IMPHY012050 | D-Galactose | OC[C@H]1OC(O)[C@@H]([C@H]([C@H]1O)O)O | 6036 |
| IMPHY012223 | beta-Amyrin | O[C@H]1CC[C@]2([C@H](C1(C)C)CC[C@@]1([C@@H]2CC=C2[C@@]1(C)CC[C@@]1([C@H]2CC(C)(C)CC1)C)C)C | 73145 |
| IMPHY012310 | Nimbin | COC(=O)C[C@H]1[C@@]2(C)[C@H](O[C@H]3C2=C(C)[C@@H](C3)c2cocc2)[C@@H]([C@@H]2[C@]1(C)C(=O)C=C[C@@]2(C)C(=O)OC)OC(=O)C | 108058 |
| IMPHY012367 | Trichilin D | CCC(C(=O)OC1OC[C@@]23[C@H]([C@@]1(C)[C@H](OC(=O)C)[C@@H]([C@@H]2O)OC(=O)C)C[C@H]([C@@]1([C@@H]3C(=O)C[C@@]2([C@]31O[C@@H]3C[C@H]2c1ccoc1)C)C)O)C | 101665324 |
| IMPHY012402 | Campesterol | O[C@H]1CC[C@]2(C(=CC[C@@H]3[C@@H]2CC[C@]2([C@H]3CC[C@@H]2[C@@H](CC[C@H](C(C)C)C)C)C)C1)C | 173183 |
| IMPHY012408 | Nimbosone | COc1cc2c(cc1C(=O)C)CC[C@@H]1[C@]2(C)CCCC1(C)C | 177090 |
| IMPHY012473 | Lupeol | CC(=C)[C@@H]1CC[C@]2([C@H]1[C@H]1CC[C@H]3[C@@]([C@]1(C)CC2)(C)CC[C@@H]1[C@]3(C)CC[C@@H](C1(C)C)O)C | 259846 |
| IMPHY012479 | 2-Methyltricosane | CCCCCCCCCCCCCCCCCCCCCC(C)C | 283510 |
| IMPHY012513 | Glycoprotein | OC[C@H]1OC(NC(=O)C[C@@H](C(=O)N)NC(=O)C)[C@@H]([C@H]([C@@H]1O[C@@H]1O[C@H](CO)[C@H]([C@@H]([C@H]1NC(=O)C)O)O[C@@H]1O[C@H](CO)[C@H]([C@@H]([C@@H]1O)O)O)O)NC(=O)C | 439212 |
| IMPHY012721 | Isoquercitrin | OC[C@H]1O[C@@H](Oc2c(oc3c(c2=O)c(O)cc(c3)O)c2ccc(c(c2)O)O)[C@@H]([C@H]([C@@H]1O)O)O | 5280804 |
| IMPHY012769 | 24-Methylenelophenol | C=C(C(C)C)CC[C@H]([C@H]1CC[C@@H]2[C@]1(C)CC[C@H]1C2=CC[C@@H]2[C@]1(C)CC[C@@H]([C@H]2C)O)C | 5283640 |
| IMPHY012918 | cis-3,5-Diethyl-1,2,4-trithiolane | CC[C@@H]1SS[C@@H](S1)CC | 6431294 |
| IMPHY013913 | 7-(Acetyloxy)-21-hydroxy-6-methoxy-4,4,8-trimethyl-3-oxocarda-1,14,20(22)-trienolide | COC1C(OC(=O)C)C2(C)C3=CCC(C3(C)CCC2C2(C1C(C)(C)C(=O)C=C2)C)C1=CC(=O)OC1O | 188754 |
| IMPHY013917 | (4aS,10aR)-6,7-dimethoxy-1,1,4a-trimethyl-3,4,10,10a-tetrahydrophenanthrene-2,9-dione | COc1cc2c(cc1OC)C(=O)C[C@@H]1[C@]2(C)CCC(=O)C1(C)C | 189660 |
| IMPHY013919 | Margolonone | O=C1CC[C@]2([C@H](C1(C)C)CC(=O)c1c2cc(C)c(c1)C(=O)O)C | 189726 |
| IMPHY013920 | (4bS,8aS)-3,4b,8,8-tetramethyl-10-oxo-6,7,8a,9-tetrahydro-5H-phenanthrene-2-carboxylic Acid | O=C1C[C@H]2C(C)(C)CCC[C@@]2(c2c1cc(C(=O)O)c(c2)C)C | 189728 |
| IMPHY014135 | Salannolactam 21 | COC(=O)CC1[C@@]2(C)[C@@H](OC(=O)/C(=C/C)/C)C[C@H]([C@@]3([C@@H]2[C@H]([C@@H]2[C@@]1(C)C1=C(C)[C@@H](C[C@H]1O2)C1=CCNC1=O)OC3)C)OC(=O)C | 6442937 |
| IMPHY014138 | Azadirachtin D | C/C=C(/C(=O)O[C@H]1C[C@@H](OC(=O)C)[C@@]2([C@H]3C41CO[C@]([C@H]4[C@@](C)([C@@H]([C@H]3OC2)O)[C@@]12O[C@@]2(C)[C@@H]2CC1O[C@H]1[C@]2(O)C=CO1)(O)C(=O)OC)C)C | 6443232 |
| IMPHY014824 | Astragalin | OC[C@H]1O[C@@H](Oc2c(oc3c(c2=O)c(O)cc(c3)O)c2ccc(cc2)O)[C@@H]([C@H]([C@@H]1O)O)O | 5282102 |
| IMPHY014836 | beta-Sitosterol | CC[C@@H](C(C)C)CC[C@H]([C@H]1CC[C@@H]2[C@]1(C)CC[C@H]1[C@H]2CC=C2[C@]1(C)CC[C@@H](C2)O)C | 222284 |
| IMPHY014838 | Daucosterol | CC[C@@H](C(C)C)CC[C@H]([C@H]1CC[C@@H]2[C@]1(C)CC[C@H]1[C@H]2CC=C2[C@]1(C)CC[C@@H](C2)O[C@@H]1O[C@H](CO)[C@H]([C@@H]([C@H]1O)O)O)C | 5742590 |
| IMPHY014842 | Stigmasterol | CC[C@@H](C(C)C)/C=C/[C@H]([C@H]1CC[C@@H]2[C@]1(C)CC[C@H]1[C@H]2CC=C2[C@]1(C)CC[C@@H](C2)O)C | 5280794 |
| IMPHY014854 | Cianidanol | Oc1cc2O[C@H](c3ccc(c(c3)O)O)[C@H](Cc2c(c1)O)O | 9064 |
| IMPHY014908 | (-)-Epicatechin | Oc1cc2O[C@H](c3ccc(c(c3)O)O)[C@@H](Cc2c(c1)O)O | 72276 |
| IMPHY014925 | beta-D-Glucose | OC[C@H]1O[C@@H](O)[C@@H]([C@H]([C@@H]1O)O)O | 64689 |
| IMPHY014935 | Hyperoside | OC[C@H]1O[C@@H](Oc2c(oc3c(c2=O)c(O)cc(c3)O)c2ccc(c(c2)O)O)[C@@H]([C@H]([C@H]1O)O)O | 5281643 |
| IMPHY014983 | L-Arabinose | O[C@H]1COC([C@@H]([C@H]1O)O)O | 439195 |
| IMPHY014990 | Linoleic acid | CCCCC/C=CC/C=CCCCCCCCC(=O)O | 5280450 |
| IMPHY015047 | Rutin | Oc1cc(O)c2c(c1)oc(c(c2=O)O[C@@H]1O[C@H](CO[C@@H]2O[C@@H](C)[C@@H]([C@H]([C@H]2O)O)O)[C@H]([C@@H]([C@H]1O)O)O)c1ccc(c(c1)O)O | 5280805 |
| IMPHY015054 | Quercitrin | Oc1cc(O)c2c(c1)oc(c(c2=O)O[C@@H]1O[C@@H](C)[C@@H]([C@H]([C@H]1O)O)O)c1ccc(c(c1)O)O | 5280459 |
| IMPHY015056 | L-Rhamnose | O[C@H]1[C@H](C)OC([C@@H]([C@@H]1O)O)O | 25310 |
| IMPHY015492 | 3-Tigloylazadirachtin | C/C=C(/C(=O)O[C@H]1C[C@@H](OC(=O)C)[C@@]2([C@H]3[C@@]41CO[C@]([C@H]4[C@]([C@@H]([C@@H]3OC2)OC(=O)/C(=C/C)/C)(C)[C@@]12O[C@@]2(C)[C@H]2C[C@@H]1O[C@H]1[C@]2(O)C=CO1)(O)C(=O)OC)C(=O)OC)C | 122232601 |
| IMPHY015493 | 3-Desacetylazadirachtin | C/C=C(/C(=O)O[C@H]1C[C@@H](O)[C@@]2([C@H]3C41CO[C@]([C@H]4[C@@](C)([C@@H]([C@H]3OC2)O)[C@@]12O[C@@]2(C)[C@@H]2CC1O[C@H]1[C@]2(O)C=CO1)(O)C(=O)OC)C(=O)OC)C | 6442561 |
| IMPHY015625 | Dipropyl tetrasulfide | CCCSSSSCCC | 104285 |
| IMPHY015706 | Glycopeptide | NCCCC[C@H](C(=O)N[C@H](C(=O)O)C)NC(=O)CC[C@H](C(=O)N)NC(=O)[C@H](NC(=O)C(O[C@@H]1[C@@H](NCC)C(O)O[C@@H]([C@H]1O[C@@H]1O[C@H](CO)[C@H]([C@@H]([C@H]1NC(=O)C)O)O)CO)C)C | 56928060 |
| IMPHY015796 | Limocinin | COC1OCC(C1)(O)[C@H]1CC=C2[C@@]1(C)CC[C@H]1[C@@]2(C)[C@@H](C[C@@H]2[C@]1(C)C=CC(=O)C2(C)C)OC(=O)c1ccccc1 | 14845550 |
| IMPHY015797 | Limocinol | CC(=CCC[C@@H]([C@@H]1[C@@H](O)C[C@]2([C@@]1(C)CC[C@H]1C2=CC[C@@H]2[C@]1(C)CCCC2(C)C)C)C)C | 14845542 |
| IMPHY015811 | Melatonin | COc1ccc2c(c1)c(CCNC(=O)C)c[nH]2 | 896 |
| IMPHY015812 | 6-Methoxymellein | COc1cc2C[C@@H](C)OC(=O)c2c(c1)O | 83412 |
| IMPHY015829 | Methyl butyl disulfide | CCCCSSC | 521941 |
| IMPHY015844 | Methyl propyl tetrasulphide | CCCSSSSC | 528709 |
| IMPHY015861 | Isonimbinolide | COC(=O)C[C@H]1[C@@]2(C)[C@H](O[C@H]3C2=C(C)[C@@H](C3)C2=CC(=O)OC2O)[C@@H]([C@@H]2[C@]1(C)C(=O)C=C[C@@]2(C)C(=O)OC)OC(=O)C | 14136864 |
| IMPHY016049 | trans-3,5-Diethyl-1,2,4-trithiolane | CC[C@H]1SS[C@@H](S1)CC | 6432398 |
|  |  |  |  |
|  |  |  |  |
|  |  |  |  |
|  |  |  |  |
| ***7.Plant name : Ficus religiosa*** | | | |
| **IMPPAT Phytochemical identifier:** | **Phytochemical name:** | **SMILES:** | **CID** |
| IMPHY004534 | Bergaptol | O=c1ccc2c(o1)cc1c(c2O)cco1 | 5280371 |
| IMPHY005428 | Bergapten | COc1c2ccc(=O)oc2cc2c1cco2 | 2355 |
| IMPHY006972 | 1-Octacosanol | CCCCCCCCCCCCCCCCCCCCCCCCCCCCO | 68406 |
| IMPHY011461 | Methyl oleanolate | COC(=O)[C@@]12CCC(C[C@H]2C2=CC[C@H]3[C@@]([C@@]2(CC1)C)(C)CC[C@@H]1[C@]3(C)CC[C@@H](C1(C)C)O)(C)C | 92900 |
| IMPHY011471 | Lupenone | CC(=C)[C@@H]1CC[C@]2([C@H]1[C@H]1CC[C@H]3[C@@]([C@]1(C)CC2)(C)CC[C@@H]1[C@]3(C)CCC(=O)C1(C)C)C | 92158 |
| IMPHY011607 | Bergenin | OC[C@H]1O[C@@H]2[C@@H]([C@H]([C@@H]1O)O)OC(=O)c1c2c(O)c(c(c1)O)OC | 66065 |
| IMPHY011857 | Lanosterol | CC(=CCC[C@H]([C@H]1CC[C@@]2([C@]1(C)CCC1=C2CC[C@@H]2[C@]1(C)CC[C@@H](C2(C)C)O)C)C)C | 246983 |
| IMPHY011933 | Caffeic acid | OC(=O)/C=C/c1ccc(c(c1)O)O | 689043 |
| IMPHY014836 | beta-Sitosterol | CC[C@@H](C(C)C)CC[C@H]([C@H]1CC[C@@H]2[C@]1(C)CC[C@H]1[C@H]2CC=C2[C@]1(C)CC[C@@H](C2)O)C | 222284 |
| IMPHY014841 | beta-Sitosterol-d-glucoside | CC[C@@H](C(C)C)CC[C@H]([C@H]1CC[C@@H]2[C@]1(C)CC[C@H]1[C@H]2CC=C2[C@]1(C)CCC(C2)O[C@@H]1O[C@H](CO)[C@H]([C@@H]([C@H]1O)O)O)C | 12309060 |
| IMPHY014842 | Stigmasterol | CC[C@@H](C(C)C)/C=C/[C@H]([C@H]1CC[C@@H]2[C@]1(C)CC[C@H]1[C@H]2CC=C2[C@]1(C)CC[C@@H](C2)O)C | 5280794 |
| IMPHY006972 | 1-Octacosanol | CCCCCCCCCCCCCCCCCCCCCCCCCCCCO | 68406 |
| IMPHY011461 | Methyl oleanolate | COC(=O)[C@@]12CCC(C[C@H]2C2=CC[C@H]3[C@@]([C@@]2(CC1)C)(C)CC[C@@H]1[C@]3(C)CC[C@@H](C1(C)C)O)(C)C | 92900 |
| IMPHY011857 | Lanosterol | CC(=CCC[C@H]([C@H]1CC[C@@]2([C@]1(C)CCC1=C2CC[C@@H]2[C@]1(C)CC[C@@H](C2(C)C)O)C)C)C | 246983 |
| IMPHY012473 | Lupeol | CC(=C)[C@@H]1CC[C@]2([C@H]1[C@H]1CC[C@H]3[C@@]([C@]1(C)CC2)(C)CC[C@@H]1[C@]3(C)CC[C@@H](C1(C)C)O)C | 259846 |
| IMPHY014836 | beta-Sitosterol | CC[C@@H](C(C)C)CC[C@H]([C@H]1CC[C@@H]2[C@]1(C)CC[C@H]1[C@H]2CC=C2[C@]1(C)CC[C@@H](C2)O)C | 222284 |
| IMPHY014842 | Stigmasterol | CC[C@@H](C(C)C)/C=C/[C@H]([C@H]1CC[C@@H]2[C@]1(C)CC[C@H]1[C@H]2CC=C2[C@]1(C)CC[C@@H](C2)O)C | 5280794 |
|  |  |  |  |
|  |  |  |  |
| ***8. Plant name : Artocarpus integer*** | | | |
| **IMPPAT Phytochemical identifier:** | **Phytochemical name:** | **SMILES:** | **CID** |
| IMPHY002120 | Cycloartenone | CC(=CCC[C@H]([C@H]1CC[C@@]2([C@]1(C)CC[C@@]13[C@H]2CC[C@@H]2[C@]3(C1)CCC(=O)C2(C)C)C)C)C | 12305360 |
| IMPHY011642 | Cycloartenol | CC(=CCC[C@H]([C@H]1CC[C@@]2([C@]1(C)CC[C@@]13[C@H]2CC[C@@H]2[C@]3(C1)CC[C@@H](C2(C)C)O)C)C)C | 92110 |
| IMPHY014836 | beta-Sitosterol | CC[C@@H](C(C)C)CC[C@H]([C@H]1CC[C@@H]2[C@]1(C)CC[C@H]1[C@H]2CC=C2[C@]1(C)CC[C@@H](C2)O)C | 222284 |
| IMPHY003450 | Artocarpesin | CC(=CCc1c(O)cc2c(c1O)c(=O)cc(o2)c1ccc(cc1O)O)C | 399491 |
|  |  |  |  |
|  |  |  |  |
|  |  |  |  |
|  |  |  |  |
|  |  |  |  |
| ***9.Plant name : Artocarpus heterophyllus*** | | | |
| **IMPPAT Phytochemical identifier:** | **Phytochemical name:** | **SMILES:** | **CID** |
| IMPHY002120 | Cycloartenone | CC(=CCC[C@H]([C@H]1CC[C@@]2([C@]1(C)CC[C@@]13[C@H]2CC[C@@H]2[C@]3(C1)CCC(=O)C2(C)C)C)C)C | 12305360 |
| IMPHY002555 | Heterophyllin | CC(=CCc1c(oc2c(c1=O)c(O)c1c(c2CC=C(C)C)OC(C=C1)(C)C)c1cc(O)c(cc1O)O)C | 14557105 |
| IMPHY004689 | 9,19-Cyclolanost-24-en-3-ol, acetate, (3beta)- | CC(=CCCC(C1CCC2(C1(C)CCC13C2CCC2C3(C1)CCC(C2(C)C)OC(=O)C)C)C)C | 518616 |
| IMPHY004951 | Cycloheterophyllin | CC(=CCc1c2OC(C)(C)C=Cc2c(c2c1oc1-c3cc(O)c(cc3OC(c1c2=O)C=C(C)C)O)O)C | 5316250 |
| IMPHY006633 | Isocycloheterophyllin | CC(=CCc1c2OC(C)(C)C=Cc2c2c(c1O)c(=O)c1c(-c3cc(O)c(cc3OC(=C(C)C)C1)O)o2)C | 71437947 |
| IMPHY011642 | Cycloartenol | CC(=CCC[C@H]([C@H]1CC[C@@]2([C@]1(C)CC[C@@]13[C@H]2CC[C@@H]2[C@]3(C1)CC[C@@H](C2(C)C)O)C)C)C | 92110 |
| IMPHY011741 | Tannic acid | O=C(c1cc(O)c(c(c1)OC(=O)c1cc(O)c(c(c1)O)O)O)O[C@@H]1[C@@H](COC(=O)c2cc(O)c(c(c2)OC(=O)c2cc(O)c(c(c2)O)O)O)O[C@H]([C@@H]([C@H]1OC(=O)c1cc(O)c(c(c1)OC(=O)c1cc(O)c(c(c1)O)O)O)OC(=O)c1cc(O)c(c(c1)OC(=O)c1cc(O)c(c(c1)O)O)O)OC(=O)c1cc(O)c(c(c1)OC(=O)c1cc(O)c(c(c1)O)O)O | 16129778 |
| IMPHY012003 | Betulinic acid | CC(=C)[C@@H]1CC[C@]2([C@H]1[C@H]1CC[C@H]3[C@@]([C@]1(C)CC2)(C)CC[C@@H]1[C@]3(C)CC[C@@H](C1(C)C)O)C(=O)O | 64971 |
| IMPHY000795 | Octanal | CCCCCCCC=O | 454 |
| IMPHY001109 | Isovaleric acid | CC(CC(=O)O)C | 10430 |
| IMPHY001215 | Propanol | CCCO | 1031 |
| IMPHY002120 | Cycloartenone | CC(=CCC[C@H]([C@H]1CC[C@@]2([C@]1(C)CC[C@@]13[C@H]2CC[C@@H]2[C@]3(C1)CCC(=O)C2(C)C)C)C)C | 12305360 |
| IMPHY002139 | (-)-Butyrospermol | CC(=CCC[C@H]([C@@H]1CC[C@]2([C@@]1(C)CC[C@H]1C2=CC[C@@H]2[C@]1(C)CC[C@@H](C2(C)C)O)C)C)C | 12302182 |
| IMPHY002915 | Benzyl Alcohol | OCc1ccccc1 | 244 |
| IMPHY002949 | 1-Butanol | CCCCO | 263 |
| IMPHY003301 | Octanoic acid | CCCCCCCC(=O)O | 379 |
| IMPHY003340 | Acetoin | CC(=O)C(O)C | 179 |
| IMPHY003486 | Diacetone alcohol | CC(=O)CC(O)(C)C | 31256 |
| IMPHY003519 | Isoamyl acetate | CC(CCOC(=O)C)C | 31276 |
| IMPHY004038 | Eriodictyol | Oc1cc2O[C@@H](CC(=O)c2c(c1)O)c1ccc(c(c1)O)O | 440735 |
| IMPHY004235 | D-Glucuronic Acid | OC1O[C@H](C(=O)O)[C@H]([C@@H]([C@H]1O)O)O | 94715 |
| IMPHY004388 | Kaempferol | Oc1ccc(cc1)c1oc2cc(O)cc(c2c(=O)c1O)O | 5280863 |
| IMPHY004624 | 2-Acetyl-1-pyrroline | CC(=O)C1=NCCC1 | 522834 |
| IMPHY004751 | 3-(Methylthio)propionaldehyde | CSCCC=O | 18635 |
| IMPHY004934 | Tiglic aldehyde | C/C=C(/C=O)C | 5321950 |
| IMPHY006279 | 2-Phenylethanol | OCCc1ccccc1 | 6054 |
| IMPHY006383 | Artocarpin | COc1cc2oc(c3ccc(cc3O)O)c(c(=O)c2c(c1/C=C/C(C)C)O)CC=C(C)C | 5458461 |
| IMPHY006666 | 3-(Methylthio)-1-hexanol | CCCC(CCO)SC | 65413 |
| IMPHY006674 | Isopentyl benzoate | CC(CCOC(=O)c1ccccc1)C | 7193 |
| IMPHY007041 | Furfural | O=Cc1ccco1 | 7362 |
| IMPHY007354 | Hexanoic acid | CCCCCC(=O)O | 8892 |
| IMPHY007539 | Phenylacetaldehyde | O=CCc1ccccc1 | 998 |
| IMPHY007620 | 1-Octanol | CCCCCCCCO | 957 |
| IMPHY008673 | 3-Phenyl-1-propanol | OCCCc1ccccc1 | 31234 |
| IMPHY008726 | 3-Phenylpropanal | O=CCCc1ccccc1 | 7707 |
| IMPHY009642 | 2-Nonanone | CCCCCCCC(=O)C | 13187 |
| IMPHY009846 | Methyl isovalerate | COC(=O)CC(C)C | 11160 |
| IMPHY009946 | Benzaldehyde | O=Cc1ccccc1 | 240 |
| IMPHY009961 | Ethyl isovalerate | CCOC(=O)CC(C)C | 7945 |
| IMPHY009990 | Isopentyl isobutyrate | CC(CCOC(=O)C(C)C)C | 519786 |
| IMPHY011033 | Phenethyl isovalerate | CC(CC(=O)OCCc1ccccc1)C | 8792 |
| IMPHY011562 | 2-Hexenal | CCC/C=C/C=O | 5281168 |
| IMPHY011642 | Cycloartenol | CC(=CCC[C@H]([C@H]1CC[C@@]2([C@]1(C)CC[C@@]13[C@H]2CC[C@@H]2[C@]3(C1)CC[C@@H](C2(C)C)O)C)C)C | 92110 |
| IMPHY011707 | beta-Carotene | C/C(=CC=CC=C(C=CC=C(C=CC1=C(C)CCCC1(C)C)/C)/C)/C=C/C=C(/C=C/C1=C(C)CCCC1(C)C)C | 5280489 |
| IMPHY011729 | Mannitol | OC[C@H]([C@H]([C@@H]([C@@H](CO)O)O)O)O | 6251 |
| IMPHY012028 | Propionic acid | CCC(=O)O | 1032 |
| IMPHY012050 | D-Galactose | OC[C@H]1OC(O)[C@@H]([C@H]([C@H]1O)O)O | 6036 |
| IMPHY012058 | Linalool | C=CC(CCC=C(C)C)(O)C | 6549 |
| IMPHY012059 | Isobutanol | OCC(C)C | 6560 |
| IMPHY012089 | Valeric acid | CCCCC(=O)O | 7991 |
| IMPHY013770 | Propyl isovalerate | CCCOC(=O)CC(C)C | 11176 |
| IMPHY014836 | beta-Sitosterol | CC[C@@H](C(C)C)CC[C@H]([C@H]1CC[C@@H]2[C@]1(C)CC[C@H]1[C@H]2CC=C2[C@]1(C)CC[C@@H](C2)O)C | 222284 |
| IMPHY014893 | D-Glucose | OC[C@H]1OC(O)[C@@H]([C@H]([C@@H]1O)O)O | 5793 |
| IMPHY015000 | D-Mannose | OC[C@H]1OC(O)[C@H]([C@H]([C@@H]1O)O)O | 18950 |
| IMPHY015223 | 2,3-Pentanedione | CCC(=O)C(=O)C | 11747 |
| IMPHY015288 | Isobutyl isovalerate | CC(COC(=O)CC(C)C)C | 11514 |
| IMPHY015522 | Butyl isovalerate | CCCCOC(=O)CC(C)C | 7981 |
| IMPHY015824 | Methyl 3-hydroxy-3-methylbutanoate | COC(=O)CC(O)(C)C | 521979 |
| IMPHY015867 | Propyl acetate | CCCOC(=O)C | 7997 |
| IMPHY002120 | Cycloartenone | CC(=CCC[C@H]([C@H]1CC[C@@]2([C@]1(C)CC[C@@]13[C@H]2CC[C@@H]2[C@]3(C1)CCC(=O)C2(C)C)C)C)C | 12305360 |
| IMPHY011642 | Cycloartenol | CC(=CCC[C@H]([C@H]1CC[C@@]2([C@]1(C)CC[C@@]13[C@H]2CC[C@@H]2[C@]3(C1)CC[C@@H](C2(C)C)O)C)C)C | 92110 |
| IMPHY011741 | Tannic acid | O=C(c1cc(O)c(c(c1)OC(=O)c1cc(O)c(c(c1)O)O)O)O[C@@H]1[C@@H](COC(=O)c2cc(O)c(c(c2)OC(=O)c2cc(O)c(c(c2)O)O)O)O[C@H]([C@@H]([C@H]1OC(=O)c1cc(O)c(c(c1)OC(=O)c1cc(O)c(c(c1)O)O)O)OC(=O)c1cc(O)c(c(c1)OC(=O)c1cc(O)c(c(c1)O)O)O)OC(=O)c1cc(O)c(c(c1)OC(=O)c1cc(O)c(c(c1)O)O)O | 16129778 |
| IMPHY011871 | Procyanidin B3 | Oc1cc(O)c2c(c1)O[C@@H]([C@H]([C@@H]2c1c(O)cc(c2c1O[C@@H]([C@H](C2)O)c1ccc(c(c1)O)O)O)O)c1ccc(c(c1)O)O | 146798 |
| IMPHY014836 | beta-Sitosterol | CC[C@@H](C(C)C)CC[C@H]([C@H]1CC[C@@H]2[C@]1(C)CC[C@H]1[C@H]2CC=C2[C@]1(C)CC[C@@H](C2)O)C | 222284 |
| IMPHY014854 | Cianidanol | Oc1cc2O[C@H](c3ccc(c(c3)O)O)[C@H](Cc2c(c1)O)O | 9064 |
| IMPHY015045 | Procyanidin C1 | Oc1cc(O)c2c(c1)O[C@@H]([C@@H]([C@H]2c1c(O)cc(c2c1O[C@H](c1ccc(c(c1)O)O)[C@@H]([C@H]2c1c(O)cc(c2c1O[C@@H]([C@@H](C2)O)c1ccc(c(c1)O)O)O)O)O)O)c1ccc(c(c1)O)O | 169853 |
| IMPHY002120 | Cycloartenone | CC(=CCC[C@H]([C@H]1CC[C@@]2([C@]1(C)CC[C@@]13[C@H]2CC[C@@H]2[C@]3(C1)CCC(=O)C2(C)C)C)C)C | 12305360 |
| IMPHY011642 | Cycloartenol | CC(=CCC[C@H]([C@H]1CC[C@@]2([C@]1(C)CC[C@@]13[C@H]2CC[C@@H]2[C@]3(C1)CC[C@@H](C2(C)C)O)C)C)C | 92110 |
| IMPHY014794 | 9,19-Cyclolanost-23-ene-3,25-diol | C[C@@H]([C@H]1CC[C@@]2([C@]1(C)CC[C@@]13[C@H]2CC[C@H]2[C@]3(C1)CCC(C2(C)C)O)C)C/C=C/C(O)(C)C | 129681941 |
| IMPHY001451 | Artonin K | COc1cc(O)c2c(c1)oc1-c3c(O)cc(c4c3C(Cc1c2=O)C(O4)(C)C)O | 15340661 |
| IMPHY001499 | Heterophylol | COc1cc(OC)c(c2c1CC1c3ccc(cc3OC(C1C2)(C)C)O)CC=C(C)C | 15227962 |
| IMPHY001986 | Artonin B | CC(=CCc1c2OC(C)(C)C=Cc2c(c2c1oc1-c3c(O)cc(c(c3C(Cc1c2=O)C(=C)C)O)O)O)C | 11964501 |
| IMPHY002120 | Cycloartenone | CC(=CCC[C@H]([C@H]1CC[C@@]2([C@]1(C)CC[C@@]13[C@H]2CC[C@@H]2[C@]3(C1)CCC(=O)C2(C)C)C)C)C | 12305360 |
| IMPHY002554 | artonin A | CC(=CCc1c2OC(C)(C)C=Cc2c(c2c1oc1-c3c(O)cc(c4c3C(Cc1c2=O)C(O4)(C)C)O)O)C | 14557102 |
| IMPHY002555 | Heterophyllin | CC(=CCc1c(oc2c(c1=O)c(O)c1c(c2CC=C(C)C)OC(C=C1)(C)C)c1cc(O)c(cc1O)O) | 14557105 |
| IMPHY002831 | (E)-1-[3-[(1S,5S,6R)-6-[2,4-dihydroxy-3-[(E)-3-methylbut-1-enyl]benzoyl]-5-(2,4-dihydroxyphenyl)-3-methylcyclohex-2-en-1-yl]-2,4-dihydroxyphenyl]-3-(2,4-dihydroxyphenyl)prop-2-en-1-one | CC(/C=C/c1c(O)ccc(c1O)C(=O)[C@H]1[C@H](C=C(C[C@@H]1c1ccc(cc1O)O)C)c1c(O)ccc(c1O)C(=O)/C=C/c1ccc(cc1O)O)C | 14681572 |
| IMPHY002832 | (E)-3-(2,4-dihydroxyphenyl)-1-[3-[(1S,5S,6R)-5-(2,4-dihydroxyphenyl)-6-(5-hydroxy-2,2-dimethylchromene-6-carbonyl)-3-methylcyclohex-2-en-1-yl]-2,4-dihydroxyphenyl]prop-2-en-1-one | CC1=C[C@H](c2c(O)ccc(c2O)C(=O)/C=C/c2ccc(cc2O)O)[C@@H]([C@H](C1)c1ccc(cc1O)O)C(=O)c1ccc2c(c1O)C=CC(O2)(C)C | 14681574 |
| IMPHY003430 | Heteroflavanone C | COc1cc(OC)c(c(c1)OC)C1CC(=O)c2c(O1)c(CC=C(C)C)c(cc2O)O | 42608025 |
| IMPHY004271 | Betulin | OC[C@@]12CC[C@H]([C@@H]2[C@@H]2[C@](CC1)(C)[C@]1(C)CC[C@@H]3[C@]([C@H]1CC2)(C)CC[C@@H](C3(C)C)O)C(=C)C | 72326 |
| IMPHY004747 | cycloartocarpin A | COc1ccc2-c3oc4cc(O)c(c(c4c(=O)c3C(Oc2c1)C=C(C)C)O)/C=C/C(C)C | 44258302 |
| IMPHY004778 | Artonin J | CC(=CCc1c(O)c2-c3oc4cc(O)cc(c4c(=O)c3CC3c2c(c1O)OC3(C)C)O)C | 44258663 |
| IMPHY004781 | Artonin L | COc1cc(O)c2c3c1-c1oc4cc(OC)cc(c4c(=O)c1CC3C(O2)(C)C)O | 44258662 |
| IMPHY004951 | Cycloheterophyllin | CC(=CCc1c2OC(C)(C)C=Cc2c(c2c1oc1-c3cc(O)c(cc3OC(c1c2=O)C=C(C)C)O)O)C | 5316250 |
| IMPHY008266 | Heteroflavanone A | COc1cc(OC)c(c(c1)OC)C1CC(=O)c2c(O1)cc(cc2O)OC | 42608055 |
| IMPHY008269 | Heteroflavanone B | COc1cc(OC)cc(c1C1CC(=O)c2c(O1)c(CC=C(C)C)c(cc2O)OC)OC | 42608026 |
| IMPHY010242 | (2S)-5-hydroxy-7-methoxy-6-(3-methylbut-2-enyl)-2-(3,4,5-trimethoxyphenyl)-2,3-dihydrochromen-4-one | COc1cc2O[C@@H](CC(=O)c2c(c1CC=C(C)C)O)c1cc(OC)c(c(c1)OC)OC | 90473423 |
| IMPHY011642 | Cycloartenol | CC(=CCC[C@H]([C@H]1CC[C@@]2([C@]1(C)CC[C@@]13[C@H]2CC[C@@H]2[C@]3(C1)CC[C@@H](C2(C)C)O)C)C)C | 92110 |
| IMPHY011880 | Ursolic acid | C[C@@H]1CC[C@]2([C@@H]([C@H]1C)C1=CC[C@H]3[C@@]([C@@]1(CC2)C)(C)CC[C@@H]1[C@]3(C)CC[C@@H](C1(C)C)O)C(=O)O | 64945 |
| IMPHY012003 | Betulinic acid | CC(=C)[C@@H]1CC[C@]2([C@H]1[C@H]1CC[C@H]3[C@@]([C@]1(C)CC2)(C)CC[C@@H]1[C@]3(C)CC[C@@H](C1(C)C)O)C(=O)O | 64971 |
| IMPHY013663 | 9-Hydroxytridecyl docosanoate | CCCCCCCCCCCCCCCCCCCCCC(=O)OCCCCCCCCC(CCCC)O | 129824367 |
| IMPHY014836 | beta-Sitosterol | CC[C@@H](C(C)C)CC[C@H]([C@H]1CC[C@@H]2[C@]1(C)CC[C@H]1[C@H]2CC=C2[C@]1(C)CC[C@@H](C2)O)C | 222284 |
| IMPHY015100 | Tridecyl behenate | CCCCCCCCCCCCCCCCCCCCCC(=O)OCCCCCCCCCCCCC | 22287906 |
| IMPHY006466 | Ricinoleic acid | CCCCCC[C@H](C/C=CCCCCCCCC(=O)O)O | 643684 |
| IMPHY007327 | Palmitic acid | CCCCCCCCCCCCCCCC(=O)O | 985 |
| IMPHY010589 | Asperglaucide | CC(=O)OC[C@@H](NC(=O)[C@@H](NC(=O)c1ccccc1)Cc1ccccc1)Cc1ccccc1 | 10026486 |
| IMPHY012050 | D-Galactose | OC[C@H]1OC(O)[C@@H]([C@H]([C@H]1O)O)O | 6036 |
| IMPHY012161 | L-Fucose | O[C@@H]1[C@H](C)OC([C@H]([C@@H]1O)O)O | 17106 |
| IMPHY014390 | (2s)-2-[[(2s)-2-Acetamido-5-[[n-(Methylcarbamoyl)carbamimidoyl]amino]pentanoyl]-Methyl-Amino]-3-Phenyl-Propanoic Acid | CNC(=O)N/C(=N/CCC[C@@H](C(=O)N([C@H](C(=O)O)Cc1ccccc1)C)NC(=O)C)/N | 24755478 |
| IMPHY014892 | D-Glucosamine | OC[C@H]1OC(O)[C@@H]([C@H]([C@@H]1O)O)N | 439213 |
| IMPHY014893 | D-Glucose | OC[C@H]1OC(O)[C@@H]([C@H]([C@@H]1O)O)O | 5793 |
| IMPHY014990 | Linoleic acid | CCCCC/C=CC/C=CCCCCCCCC(=O)O | 5280450 |
| IMPHY015000 | D-Mannose | OC[C@H]1OC(O)[C@H]([C@H]([C@@H]1O)O)O | 18950 |
| IMPHY015116 | D-Xylose | O[C@@H]1COC([C@@H]([C@H]1O)O)O | 135191 |
| IMPHY001403 | Artocarpanone | COc1cc2O[C@@H](CC(=O)c2c(c1)O)c1ccc(cc1O)O | 15298902 |
| IMPHY001568 | Cycloartocarpesin | Oc1ccc(c(c1)O)c1cc(=O)c2c(o1)cc1c(c2O)C=CC(O1)(C)C | 15224382 |
| IMPHY002120 | Cycloartenone | CC(=CCC[C@H]([C@H]1CC[C@@]2([C@]1(C)CC[C@@]13[C@H]2CC[C@@H]2[C@]3(C1)CCC(=O)C2(C)C)C)C)C | 12305360 |
| IMPHY003217 | Artocarpin dimethyl ether | COc1cc2oc(c3ccc(cc3OC)OC)c(c(=O)c2c(c1/C=C/C(C)C)O)CC=C(C)C | 24850643 |
| IMPHY003259 | 2-(2,4-Dihydroxyphenyl)-3,5,7-trihydroxy-2,3-dihydrochromen-4-one | Oc1ccc(c(c1)O)C1Oc2cc(O)cc(c2C(=O)C1O)O | 362637 |
| IMPHY003450 | Artocarpesin | CC(=CCc1c(O)cc2c(c1O)c(=O)cc(o2)c1ccc(cc1O)O)C | 399491 |
| IMPHY004699 | Cyanomaclurin | Oc1ccc2c(c1)O[C@@H]1C([C@@H]2Oc2c1c(O)cc(c2)O)O | 44257130 |
| IMPHY004784 | Isoartocarpin | COc1cc2oc3-c4ccc(cc4OC(c3c(=O)c2c(c1CC=C(C)C)O)C=C(C)C)O | 44258300 |
| IMPHY005463 | Morin | Oc1ccc(c(c1)O)c1oc2cc(O)cc(c2c(=O)c1O)O | 5281670 |
| IMPHY005537 | Ellagic acid | Oc1cc2c(=O)oc3c4c2c(c1O)oc(=O)c4cc(c3O)O | 5281855 |
| IMPHY005874 | Norartocarpetin | Oc1ccc(c(c1)O)c1cc(=O)c2c(o1)cc(cc2O)O | 5481970 |
| IMPHY006382 | 3,8-dihydroxy-10-methoxy-9-[(E)-3-methylbut-1-enyl]-6-(2-methylprop-1-enyl)-6H-chromeno[4,3-b]chromen-7-one | COc1cc2oc3-c4ccc(cc4OC(c3c(=O)c2c(c1/C=C/C(C)C)O)C=C(C)C)O | 5458462 |
| IMPHY006383 | Artocarpin | COc1cc2oc(c3ccc(cc3O)O)c(c(=O)c2c(c1/C=C/C(C)C)O)CC=C(C)C | 5458461 |
| IMPHY009341 | Artocarpetin | COc1cc(O)c2c(c1)oc(cc2=O)c1ccc(cc1O)O | 12308618 |
| IMPHY012053 | Sucrose | OC[C@H]1O[C@@]([C@H]([C@@H]1O)O)(CO)O[C@H]1O[C@H](CO)[C@H]([C@@H]([C@H]1O)O)O | 5988 |
| IMPHY013429 | Oxyisocyclointegrin | COc1cc(O)c2c(c1)oc1-c3ccc(cc3OC(Cc1c2=O)C(O)(C)C)O | 44258671 |
| IMPHY013430 | Cyclointegrin | COc1cc(O)c2c(c1)oc1-c3ccc(cc3OC(CCc1c2=O)(C)C)O | 44258672 |
| IMPHY001109 | Isovaleric acid | CC(CC(=O)O)C | 10430 |
| IMPHY001403 | Artocarpanone | COc1cc2O[C@@H](CC(=O)c2c(c1)O)c1ccc(cc1O)O | 15298902 |
| IMPHY002120 | Cycloartenone | CC(=CCC[C@H]([C@H]1CC[C@@]2([C@]1(C)CC[C@@]13[C@H]2CC[C@@H]2[C@]3(C1)CCC(=O)C2(C)C)C)C)C | 12305360 |
| IMPHY002555 | Heterophyllin | CC(=CCc1c(oc2c(c1=O)c(O)c1c(c2CC=C(C)C)OC(C=C1)(C)C)c1cc(O)c(cc1O)O)C | 14557105 |
| IMPHY002949 | 1-Butanol | CCCCO | 263 |
| IMPHY003450 | Artocarpesin | CC(=CCc1c(O)cc2c(c1O)c(=O)cc(o2)c1ccc(cc1O)O)C | 399491 |
| IMPHY003519 | Isoamyl acetate | CC(CCOC(=O)C)C | 31276 |
| IMPHY004235 | D-Glucuronic Acid | OC1O[C@H](C(=O)O)[C@H]([C@@H]([C@H]1O)O)O | 94715 |
| IMPHY004271 | Betulin | OC[C@@]12CC[C@H]([C@@H]2[C@@H]2[C@](CC1)(C)[C@]1(C)CC[C@@H]3[C@]([C@H]1CC2)(C)CC[C@@H](C3(C)C)O)C(=C)C | 72326 |
| IMPHY004951 | Cycloheterophyllin | CC(=CCc1c2OC(C)(C)C=Cc2c(c2c1oc1-c3cc(O)c(cc3OC(c1c2=O)C=C(C)C)O)O)C | 5316250 |
| IMPHY006362 | Ascorbic acid | OC[C@@H]([C@H]1OC(=O)C(=C1O)O)O | 54670067 |
| IMPHY006383 | Artocarpin | COc1cc2oc(c3ccc(cc3O)O)c(c(=O)c2c(c1/C=C/C(C)C)O)CC=C(C)C | 5458461 |
| IMPHY009341 | Artocarpetin | COc1cc(O)c2c(c1)oc(cc2=O)c1ccc(cc1O)O | 12308618 |
| IMPHY009846 | Methyl isovalerate | COC(=O)CC(C)C | 11160 |
| IMPHY010242 | (2S)-5-hydroxy-7-methoxy-6-(3-methylbut-2-enyl)-2-(3,4,5-trimethoxyphenyl)-2,3-dihydrochromen-4-one | COc1cc2O[C@@H](CC(=O)c2c(c1CC=C(C)C)O)c1cc(OC)c(c(c1)OC)OC | 90473423 |
| IMPHY010589 | Asperglaucide | CC(=O)OC[C@@H](NC(=O)[C@@H](NC(=O)c1ccccc1)Cc1ccccc1)Cc1ccccc1 | 10026486 |
| IMPHY011707 | beta-Carotene | C/C(=CC=CC=C(C=CC=C(C=CC1=C(C)CCCC1(C)C)/C)/C)/C=C/C=C(/C=C/C1=C(C)CCCC1(C)C)C | 5280489 |
| IMPHY011729 | Mannitol | OC[C@H]([C@H]([C@@H]([C@@H](CO)O)O)O)O | 6251 |
| IMPHY011880 | Ursolic acid | C[C@@H]1CC[C@]2([C@@H]([C@H]1C)C1=CC[C@H]3[C@@]([C@@]1(CC2)C)(C)CC[C@@H]1[C@]3(C)CC[C@@H](C1(C)C)O)C(=O)O | 64945 |
| IMPHY012003 | Betulinic acid | CC(=C)[C@@H]1CC[C@]2([C@H]1[C@H]1CC[C@H]3[C@@]([C@]1(C)CC2)(C)CC[C@@H]1[C@]3(C)CC[C@@H](C1(C)C)O)C(=O)O | 64971 |
| IMPHY012020 | Acetylcholine | CC(=O)OCC[N+](C)(C)C | 187 |
| IMPHY012050 | D-Galactose | OC[C@H]1OC(O)[C@@H]([C@H]([C@H]1O)O)O | 6036 |
| IMPHY013770 | Propyl isovalerate | CCCOC(=O)CC(C)C | 11176 |
| IMPHY014836 | beta-Sitosterol | CC[C@@H](C(C)C)CC[C@H]([C@H]1CC[C@@H]2[C@]1(C)CC[C@H]1[C@H]2CC=C2[C@]1(C)CC[C@@H](C2)O)C | 222284 |
| IMPHY014893 | D-Glucose | OC[C@H]1OC(O)[C@@H]([C@H]([C@@H]1O)O)O | 5793 |
| IMPHY015000 | D-Mannose | OC[C@H]1OC(O)[C@H]([C@H]([C@@H]1O)O)O | 18950 |
|  |  |  |  |
|  |  |  |  |
|  |  |  |  |
|  |  |  |  |
| ***10. Plant name : Cynodon dactylon*** | | | |
| **IMPPAT Phytochemical identifier:** | **Phytochemical name:** | **SMILES:** | **CID** |
| IMPHY000687 | Syringic acid | COc1cc(cc(c1O)OC)C(=O)O | CID:10742 |
| IMPHY001801 | Isoorientin | OC[C@H]1O[C@H]([C@@H]([C@H]([C@@H]1O)O)O)c1c(O)cc2c(c1O)c(=O)cc(o2)c1ccc(c(c1)O)O | CID:114776 |
| IMPHY004252 | Arundoin | CO[C@H]1CC[C@]2([C@H](C1(C)C)CC[C@H]1C2=CC[C@@]2([C@]1(C)CC[C@]1([C@H]2CC[C@@H]1C(C)C)C)C)C | CID:12308619 |
| IMPHY004660 | Luteolin | Oc1cc(O)c2c(c1)oc(cc2=O)c1ccc(c(c1)O)O | CID:5280445 |
| IMPHY004661 | Apigenin | Oc1ccc(cc1)c1cc(=O)c2c(o1)cc(cc2O)O | CID:5280443 |
| IMPHY005455 | Orientin | OC[C@H]1O[C@H]([C@@H]([C@H]([C@@H]1O)O)O)c1c(O)cc(c2c1oc(cc2=O)c1ccc(c(c1)O)O)O | CID:5281675 |
| IMPHY006362 | Ascorbic acid | OC[C@@H]([C@H]1OC(=O)C(=C1O)O)O | CID:54670067 |
| IMPHY006485 | beta-Ionone | CC(=O)/C=C/C1=C(C)CCCC1(C)C | CID:638014 |
| IMPHY007006 | Furfuryl alcohol | OCc1ccco1 | CID:7361 |
| IMPHY007041 | Furfural | O=Cc1ccco1 | CID:7362 |
| IMPHY007084 | Vanillic acid | COc1cc(ccc1O)C(=O)O | CID:8468 |
| IMPHY007212 | Docosanoic acid | CCCCCCCCCCCCCCCCCCCCCC(=O)O | CID:8215 |
| IMPHY007327 | Palmitic acid | CCCCCCCCCCCCCCCC(=O)O | CID:985 |
| IMPHY007539 | Phenylacetaldehyde | O=CCc1ccccc1 | CID:998 |
| IMPHY008689 | Isovitexin | OC[C@H]1O[C@H]([C@@H]([C@H]([C@@H]1O)O)O)c1c(O)cc2c(c1O)c(=O)cc(o2)c1ccc(cc1)O | CID:162350 |
| IMPHY009483 | Tritriacontane | CCCCCCCCCCCCCCCCCCCCCCCCCCCCCCCCC | CID:12411 |
| IMPHY010083 | 4-Hydroxybenzoic acid | Oc1ccc(cc1)C(=O)O | CID:135 |
| IMPHY011436 | Triglochinin | OC[C@H]1O[C@@H](O/C(=C(CC(=O)O)/C=CC(=O)O)/C#N)[C@@H]([C@H]([C@@H]1O)O)O | CID:5281124 |
| IMPHY011688 | Friedelin | O=C1CC[C@@H]2[C@]([C@H]1C)(C)CC[C@H]1[C@@]2(C)CC[C@@]2([C@]1(C)CC[C@@]1([C@H]2CC(C)(C)CC1)C)C | CID:91472 |
| IMPHY011707 | beta-Carotene | C/C(=CC=CC=C(C=CC=C(C=CC1=C(C)CCCC1(C)C)/C)/C)/C=C/C=C(/C=C/C1=C(C)CCCC1(C)C)C | CID:5280489 |
| IMPHY011751 | Ergometrine | OC[C@@H](NC(=O)[C@H]1CN(C)[C@H]2C(=C1)c1cccc3c1c(C2)c[nH]3)C | CID:443884 |
| IMPHY011760 | Ergometrinine | OC[C@@H](NC(=O)[C@@H]1CN(C)[C@H]2C(=C1)c1cccc3c1c(C2)c[nH]3)C | CID:5486180 |
| IMPHY011802 | Ferulic acid | COc1cc(/C=C/C(=O)O)ccc1O | CID:445858 |
| IMPHY011974 | 4-Hydroxycinnamic acid | OC(=O)/C=C/c1ccc(cc1)O | CID:637542 |
| IMPHY012713 | Vitexin | OC[C@H]1O[C@H]([C@@H]([C@H]([C@@H]1O)O)O)c1c(O)cc(c2c1oc(cc2=O)c1ccc(cc1)O)O | CID:5280441 |
| IMPHY014836 | beta-Sitosterol | CC[C@@H](C(C)C)CC[C@H]([C@H]1CC[C@@H]2[C@]1(C)CC[C@H]1[C@H]2CC=C2[C@]1(C)CC[C@@H](C2)O)C | CID:222284 |
| IMPHY005601 | Tricin | COc1cc(cc(c1O)OC)c1cc(=O)c2c(o1)cc(cc2O)O | CID:5281702 |
| IMPHY006362 | Ascorbic acid | OC[C@@H]([C@H]1OC(=O)C(=C1O)O)O | CID:54670067 |
| IMPHY000687 | Syringic acid | COc1cc(cc(c1O)OC)C(=O)O | CID:10742 |
| IMPHY001135 | 6,10,14-Trimethylpentadecan-2-one | CC(CCCC(C)C)CCCC(CCCC(=O)C)C | CID:10408 |
| IMPHY004119 | Stigmasterol acetate | CC[C@@H](C(C)C)/C=C/[C@H]([C@H]1CC[C@@H]2[C@]1(C)CC[C@H]1[C@H]2CC=C2[C@]1(C)CC[C@@H](C2)OC(=O)C)C | CID:6437330 |
| IMPHY004252 | Arundoin | CO[C@H]1CC[C@]2([C@H](C1(C)C)CC[C@H]1C2=CC[C@@]2([C@]1(C)CC[C@]1([C@H]2CC[C@@H]1C(C)C)C)C)C | CID:12308619 |
| IMPHY006485 | beta-Ionone | CC(=O)/C=C/C1=C(C)CCCC1(C)C | CID:638014 |
| IMPHY007041 | Furfural | O=Cc1ccco1 | CID:7362 |
| IMPHY007084 | Vanillic acid | COc1cc(ccc1O)C(=O)O | CID:8468 |
| IMPHY010083 | 4-Hydroxybenzoic acid | Oc1ccc(cc1)C(=O)O | CID:135 |
| IMPHY011802 | Ferulic acid | COc1cc(/C=C/C(=O)O)ccc1O | CID:445858 |
| IMPHY011974 | 4-Hydroxycinnamic acid | OC(=O)/C=C/c1ccc(cc1)O | CID:637542 |
| IMPHY012712 | Phytol | OC/C=C(/CCC[C@@H](CCC[C@@H](CCCC(C)C)C)C)C | CID:5280435 |
| IMPHY014841 | beta-Sitosterol-d-glucoside | CC[C@@H](C(C)C)CC[C@H]([C@H]1CC[C@@H]2[C@]1(C)CC[C@H]1[C@H]2CC=C2[C@]1(C)CCC(C2)O[C@@H]1O[C@H](CO)[C@H]([C@@H]([C@H]1O)O)O)C | CID:12309060 |
| IMPHY000687 | Syringic acid | COc1cc(cc(c1O)OC)C(=O)O | CID:10742 |
| IMPHY004119 | Stigmasterol acetate | CC[C@@H](C(C)C)/C=C/[C@H]([C@H]1CC[C@@H]2[C@]1(C)CC[C@H]1[C@H]2CC=C2[C@]1(C)CC[C@@H](C2)OC(=O)C)C | CID:6437330 |
| IMPHY004660 | Luteolin | Oc1cc(O)c2c(c1)oc(cc2=O)c1ccc(c(c1)O)O | CID:5280445 |
| IMPHY004661 | Apigenin | Oc1ccc(cc1)c1cc(=O)c2c(o1)cc(cc2O)O | CID:5280443 |
| IMPHY006485 | beta-Ionone | CC(=O)/C=C/C1=C(C)CCCC1(C)C | CID:638014 |
| IMPHY007084 | Vanillic acid | COc1cc(ccc1O)C(=O)O | CID:8468 |
| IMPHY010083 | 4-Hydroxybenzoic acid | Oc1ccc(cc1)C(=O)O | CID:135 |
| IMPHY011802 | Ferulic acid | COc1cc(/C=C/C(=O)O)ccc1O | CID:445858 |
| IMPHY011974 | 4-Hydroxycinnamic acid | OC(=O)/C=C/c1ccc(cc1)O | CID:637542 |
| IMPHY012712 | Phytol | OC/C=C(/CCC[C@@H](CCC[C@@H](CCCC(C)C)C)C)C | CID:5280435 |
| IMPHY013858 | 2-(4-Hydroxyphenyl)propanoic acid | OC(=O)C(c1ccc(cc1)O)C | CID:102526 |
| IMPHY014836 | beta-Sitosterol | CC[C@@H](C(C)C)CC[C@H]([C@H]1CC[C@@H]2[C@]1(C)CC[C@H]1[C@H]2CC=C2[C@]1(C)CC[C@@H](C2)O)C | CID:222284 |
| IMPHY014841 | beta-Sitosterol-d-glucoside | CC[C@@H](C(C)C)CC[C@H]([C@H]1CC[C@@H]2[C@]1(C)CC[C@H]1[C@H]2CC=C2[C@]1(C)CCC(C2)O[C@@H]1O[C@H](CO)[C@H]([C@@H]([C@H]1O)O)O)C | CID:12309060 |
|  |  |  |  |
|  |  |  |  |
| ***11. Plant name : Piper longum*** | | | |
| **IMPPAT Phytochemical identifier:** | **Phytochemical name:** | **SMILES:** | **CID** |
| IMPHY004341 | Piperlonguminine | CC(CNC(=O)/C=C/C=C/c1ccc2c(c1)OCO2)C | 5320621 |
| IMPHY006342 | Piperlongumine | COc1cc(/C=C/C(=O)N2CCC=CC2=O)cc(c1OC)OC | 637858 |
| IMPHY014895 | 5-[3-(1,3-Benzodioxol-5-yl)-1,3,3a,4,6,6a-hexahydrofuro[3,4-c]furan-6-yl]-1,3-benzodioxole | C1Oc2c(O1)cc(cc2)C1OCC2C1COC2c1ccc2c(c1)OCO2 | 5204 |
| IMPHY000308 | Hexadecane | CCCCCCCCCCCCCCCC | 11006 |
| IMPHY001313 | Piperundecalidine | O=C(N1CCCCC1)/C=C/C=C/CCCC/C=C/c1ccc2c(c1)OCO2 | 44453654 |
| IMPHY001881 | 2-Tridecanone | CCCCCCCCCCCC(=O)C | 11622 |
| IMPHY001915 | Octadecane | CCCCCCCCCCCCCCCCCC | 11635 |
| IMPHY003053 | 1-Pentadecene | CCCCCCCCCCCCCC=C | 25913 |
| IMPHY003329 | Norcepharadione B | COc1cc2C(=O)C(=O)Nc3c2c(c1OC)c1ccccc1c3 | 189168 |
| IMPHY003485 | Myrcene | C=CC(=C)CCC=C(C)C | 31253 |
| IMPHY004192 | Piperine | O=C(N1CCCCC1)/C=C/C=C/c1ccc2c(c1)OCO2 | 638024 |
| IMPHY005260 | Aristolodione | COc1c(O)cc2c3c1c1ccccc1cc3N(C(=O)C2=O)C | 184116 |
| IMPHY005360 | Aristolactam BII | COc1c(OC)cc2c3c1c1ccccc1cc3NC2=O | 162739 |
| IMPHY006145 | p-Cymene | Cc1ccc(cc1)C(C)C | 7463 |
| IMPHY006149 | 4'-Methoxyacetophenone | COc1ccc(cc1)C(=O)C | 7476 |
| IMPHY006184 | Dehydropipernonaline | O=C(N1CCCCC1)/C=C/C=C/CC/C=C/c1ccc2c(c1)OCO2 | 6439947 |
| IMPHY006266 | Guineensine | CC(CNC(=O)/C=C/C=C/CCCCCC/C=C/c1ccc2c(c1)OCO2)C | 6442405 |
| IMPHY006279 | 2-Phenylethanol | OCCc1ccccc1 | 6054 |
| IMPHY006342 | Piperlongumine | COc1cc(/C=C/C(=O)N2CCC=CC2=O)cc(c1OC)OC | 637858 |
| IMPHY006624 | Pluviatilol | COc1cc(ccc1O)[C@H]1OC[C@@H]2[C@H]1CO[C@H]2c1ccc2c(c1)OCO2 | 70695727 |
| IMPHY006951 | Eicosane | CCCCCCCCCCCCCCCCCCCC | 8222 |
| IMPHY006970 | Decanal | CCCCCCCCCC=O | 8175 |
| IMPHY006992 | 1-Methylhexyl acetate | CCCCCC(OC(=O)C)C | 80018 |
| IMPHY007204 | Dodecanal | CCCCCCCCCCCC=O | 8194 |
| IMPHY007376 | beta-Cubebene | CC([C@@H]1CC[C@H]([C@]23[C@H]1[C@H]2C(=C)CC3)C)C | 93081 |
| IMPHY007528 | Cadinane | C[C@H]1CC[C@@H]2[C@@H](C1)[C@@H](CC[C@@H]2C)C(C)C | 9548708 |
| IMPHY007559 | Pipernonaline | O=C(N1CCCCC1)/C=C/CCCC/C=C/c1ccc2c(c1)OCO2 | 9974595 |
| IMPHY007632 | Aristololactam | COc1cccc2c1cc1NC(=O)c3c1c2c1OCOc1c3 | 96710 |
| IMPHY009368 | Heptadecane | CCCCCCCCCCCCCCCCC | 12398 |
| IMPHY009369 | Nonadecane | CCCCCCCCCCCCCCCCCCC | 12401 |
| IMPHY009382 | Heneicosane | CCCCCCCCCCCCCCCCCCCCC | 12403 |
| IMPHY009389 | Pentadecane | CCCCCCCCCCCCCCC | 12391 |
| IMPHY009419 | Tridecane | CCCCCCCCCCCCC | 12388 |
| IMPHY009642 | 2-Nonanone | CCCCCCCC(=O)C | 13187 |
| IMPHY010072 | Eucalyptol | CC12CCC(CC1)C(O2)(C)C | 2758 |
| IMPHY010080 | beta-Elemene | C=C[C@]1(C)CC[C@H](C[C@H]1C(=C)C)C(=C)C | 6918391 |
| IMPHY011464 | Isobutyramide | CC(C(=O)N)C | 68424 |
| IMPHY011519 | alpha-Terpinyl acetate | CC(=O)OC(C1CCC(=CC1)C)(C)C | 111037 |
| IMPHY011521 | 2-Undecanone | CCCCCCCCCC(=O)C | 8163 |
| IMPHY011542 | beta-Eudesmol | C=C1CCC[C@]2([C@H]1C[C@@H](CC2)C(O)(C)C)C | 91457 |
| IMPHY011552 | (1R)-2-methyl-5-propan-2-ylbicyclo[3.1.0]hex-2-ene | CC1=CCC2([C@@H]1C2)C(C)C | 6451618 |
| IMPHY011567 | Fargesin | COc1cc(ccc1OC)[C@@H]1OC[C@H]2[C@@H]1CO[C@@H]2c1ccc2c(c1)OCO2 | 10926754 |
| IMPHY011593 | Pellitorine | CCCCC/C=C/C=C/C(=O)NCC(C)C | 5318516 |
| IMPHY011599 | Terpinolene | CC1=CCC(=C(C)C)CC1 | 11463 |
| IMPHY011641 | (+)-alpha-Gurjunene | C[C@H]1CC[C@H]2[C@@H](C3=C(CC[C@@H]13)C)C2(C)C | 15560275 |
| IMPHY011659 | alpha-Muurolene | CC1=C[C@@H]2[C@H](CC1)C(=CC[C@H]2C(C)C)C | 12306047 |
| IMPHY011709 | alpha-Eudesmol | CC1=CCC[C@]2([C@H]1C[C@@H](CC2)C(O)(C)C)C | 92762 |
| IMPHY011745 | Zingiberene | CC(=CCC[C@@H]([C@H]1CC=C(C=C1)C)C)C | 92776 |
| IMPHY011761 | Humulene | C/C/1=CCC(C)(C)/C=C/C/C(=C/CC1)/C | 5281520 |
| IMPHY011792 | gamma-Muurolene | CC1=C[C@@H]2[C@H](CC1)C(=C)CC[C@H]2C(C)C | 12313020 |
| IMPHY011839 | (Z)-gamma-bisabolene | CC(=CCC/C(=C1/CCC(=CC1)C)/C)C | 3033866 |
| IMPHY011890 | Elemol | C=C[C@]1(C)CC[C@H](C[C@H]1C(=C)C)C(O)(C)C | 92138 |
| IMPHY011965 | (+)-beta-Phellandrene | CC([C@@H]1CCC(=C)C=C1)C | 442484 |
| IMPHY012058 | Linalool | C=CC(CCC=C(C)C)(O)C | 6549 |
| IMPHY012061 | alpha-Pinene | CC1=CCC2CC1C2(C)C | 6654 |
| IMPHY012130 | Dihydrocarveol | CC(=C)C1CCC(C(C1)O)C | 12072 |
| IMPHY012147 | beta-Pinene | C=C1CCC2CC1C2(C)C | 14896 |
| IMPHY012160 | alpha-Terpineol | CC1=CCC(CC1)C(O)(C)C | 17100 |
| IMPHY012305 | beta-Patchoulene | CC1CCC2=C1CC1CCC2(C1(C)C)C | 101731 |
| IMPHY012353 | Aristolactam AII | COc1c(O)cc2c3c1c1ccccc1cc3NC2=O | 148657 |
| IMPHY012585 | delta-Cadinol | CC1=C[C@@H]2[C@H](CC1)[C@](C)(O)CC[C@H]2C(C)C | 3084311 |
| IMPHY012586 | (-)-alpha-Cadinol | CC1=CC2C(CC1)[C@@](C)(O)CC[C@@H]2C(C)C | 6431302 |
| IMPHY012615 | 9-Eicosyne | CCCCCCCCCCC#CCCCCCCCC | 557019 |
| IMPHY012667 | Caryophyllene oxide | C=C1CC[C@H]2O[C@@]2(CC[C@@H]2[C@@H]1CC2(C)C)C | 1742210 |
| IMPHY012739 | (Z)-beta-Ocimene | C=C/C(=CCC=C(C)C)/C | 5320250 |
| IMPHY012921 | gamma-Elemene | C=C[C@]1(C)CCC(=C(C)C)C[C@H]1C(=C)C | 6432312 |
| IMPHY013093 | delta-Elemene | C=C[C@@]1(C)CCC(=C[C@@H]1C(=C)C)C(C)C | 12309449 |
| IMPHY014811 | alpha-Phellandrene | CC1=CCC(C=C1)C(C)C | 7460 |
| IMPHY014831 | beta-Caryophyllene | C/C/1=CCCC(=C)[C@@H]2[C@@H](CC1)C(C2)(C)C | 5281515 |
| IMPHY014835 | (E)-beta-ocimene | C=C/C(=C/CC=C(C)C)/C | 5281553 |
| IMPHY014847 | Bornyl acetate | CC(=O)OC1CC2C(C1(C)CC2)(C)C | 6448 |
| IMPHY014852 | Camphene | C=C1C2CCC(C1(C)C)C2 | 6616 |
| IMPHY014881 | Copaene | CC(C1CCC2(C3C1C2C(=CC3)C)C)C | 19725 |
| IMPHY014895 | 5-[3-(1,3-Benzodioxol-5-yl)-1,3,3a,4,6,6a-hexahydrofuro[3,4-c]furan-6-yl]-1,3-benzodioxole | C1Oc2c(O1)cc(cc2)C1OCC2C1COC2c1ccc2c(c1)OCO2 | 5204 |
| IMPHY014988 | Limonene | CC1=CCC(CC1)C(=C)C | 22311 |
| IMPHY015022 | Nerolidol | C=CC(CC/C=C(/CCC=C(C)C)C)(O)C | 5284507 |
| IMPHY001881 | 2-Tridecanone | CCCCCCCCCCCC(=O)C | 11622 |
| IMPHY003053 | 1-Pentadecene | CCCCCCCCCCCCCC=C | 25913 |
| IMPHY003485 | Myrcene | C=CC(=C)CCC=C(C)C | 31253 |
| IMPHY006970 | Decanal | CCCCCCCCCC=O | 8175 |
| IMPHY006992 | 1-Methylhexyl acetate | CCCCCC(OC(=O)C)C | 80018 |
| IMPHY007204 | Dodecanal | CCCCCCCCCCCC=O | 8194 |
| IMPHY007376 | beta-Cubebene | CC([C@@H]1CC[C@H]([C@]23[C@H]1[C@H]2C(=C)CC3)C)C | 93081 |
| IMPHY007528 | Cadinane | C[C@H]1CC[C@@H]2[C@@H](C1)[C@@H](CC[C@@H]2C)C(C)C | 9548708 |
| IMPHY009368 | Heptadecane | CCCCCCCCCCCCCCCCC | 12398 |
| IMPHY009389 | Pentadecane | CCCCCCCCCCCCCCC | 12391 |
| IMPHY009419 | Tridecane | CCCCCCCCCCCCC | 12388 |
| IMPHY009642 | 2-Nonanone | CCCCCCCC(=O)C | 13187 |
| IMPHY010072 | Eucalyptol | CC12CCC(CC1)C(O2)(C)C | 2758 |
| IMPHY010080 | beta-Elemene | C=C[C@]1(C)CC[C@H](C[C@H]1C(=C)C)C(=C)C | 6918391 |
| IMPHY011519 | alpha-Terpinyl acetate | CC(=O)OC(C1CCC(=CC1)C)(C)C | 111037 |
| IMPHY011521 | 2-Undecanone | CCCCCCCCCC(=O)C | 8163 |
| IMPHY011542 | beta-Eudesmol | C=C1CCC[C@]2([C@H]1C[C@@H](CC2)C(O)(C)C)C | 91457 |
| IMPHY011599 | Terpinolene | CC1=CCC(=C(C)C)CC1 | 11463 |
| IMPHY011641 | (+)-alpha-Gurjunene | C[C@H]1CC[C@H]2[C@@H](C3=C(CC[C@@H]13)C)C2(C)C | 15560275 |
| IMPHY011659 | alpha-Muurolene | CC1=C[C@@H]2[C@H](CC1)C(=CC[C@H]2C(C)C)C | 12306047 |
| IMPHY011709 | alpha-Eudesmol | CC1=CCC[C@]2([C@H]1C[C@@H](CC2)C(O)(C)C)C | 92762 |
| IMPHY011761 | Humulene | C/C/1=CCC(C)(C)/C=C/C/C(=C/CC1)/C | 5281520 |
| IMPHY011792 | gamma-Muurolene | CC1=C[C@@H]2[C@H](CC1)C(=C)CC[C@H]2C(C)C | 12313020 |
| IMPHY011839 | (Z)-gamma-bisabolene | CC(=CCC/C(=C1/CCC(=CC1)C)/C)C | 3033866 |
| IMPHY011890 | Elemol | C=C[C@]1(C)CC[C@H](C[C@H]1C(=C)C)C(O)(C)C | 92138 |
| IMPHY011965 | (+)-beta-Phellandrene | CC([C@@H]1CCC(=C)C=C1)C | 442484 |
| IMPHY012058 | Linalool | C=CC(CCC=C(C)C)(O)C | 6549 |
| IMPHY012061 | alpha-Pinene | CC1=CCC2CC1C2(C)C | 6654 |
| IMPHY012147 | beta-Pinene | C=C1CCC2CC1C2(C)C | 14896 |
| IMPHY012160 | alpha-Terpineol | CC1=CCC(CC1)C(O)(C)C | 17100 |
| IMPHY012305 | beta-Patchoulene | CC1CCC2=C1CC1CCC2(C1(C)C)C | 101731 |
| IMPHY012585 | delta-Cadinol | CC1=C[C@@H]2[C@H](CC1)[C@](C)(O)CC[C@H]2C(C)C | 3084311 |
| IMPHY012586 | (-)-alpha-Cadinol | CC1=CC2C(CC1)[C@@](C)(O)CC[C@@H]2C(C)C | 6431302 |
| IMPHY012615 | 9-Eicosyne | CCCCCCCCCCC#CCCCCCCCC | 557019 |
| IMPHY012667 | Caryophyllene oxide | C=C1CC[C@H]2O[C@@]2(CC[C@@H]2[C@@H]1CC2(C)C)C | 1742210 |
| IMPHY012739 | (Z)-beta-Ocimene | C=C/C(=CCC=C(C)C)/C | 5320250 |
| IMPHY012921 | gamma-Elemene | C=C[C@]1(C)CCC(=C(C)C)C[C@H]1C(=C)C | 6432312 |
| IMPHY013093 | delta-Elemene | C=C[C@@]1(C)CCC(=C[C@@H]1C(=C)C)C(C)C | 12309449 |
| IMPHY014811 | alpha-Phellandrene | CC1=CCC(C=C1)C(C)C | 7460 |
| IMPHY014831 | beta-Caryophyllene | C/C/1=CCCC(=C)[C@@H]2[C@@H](CC1)C(C2)(C)C | 5281515 |
| IMPHY014835 | (E)-beta-ocimene | C=C/C(=C/CC=C(C)C)/C | 5281553 |
| IMPHY014847 | Bornyl acetate | CC(=O)OC1CC2C(C1(C)CC2)(C)C | 6448 |
| IMPHY014852 | Camphene | C=C1C2CCC(C1(C)C)C2 | 6616 |
| IMPHY014881 | Copaene | CC(C1CCC2(C3C1C2C(=CC3)C)C)C | 19725 |
| IMPHY014988 | Limonene | CC1=CCC(CC1)C(=C)C | 22311 |
| IMPHY015022 | Nerolidol | C=CC(CC/C=C(/CCC=C(C)C)C)(O)C | 5284507 |
| IMPHY001881 | 2-Tridecanone | CCCCCCCCCCCC(=O)C | 11622 |
| IMPHY003053 | 1-Pentadecene | CCCCCCCCCCCCCC=C | 25913 |
| IMPHY003328 | Cepharadione B | COc1cc2C(=O)C(=O)N(c3c2c(c1OC)c1ccccc1c3)C | 189151 |
| IMPHY003329 | Norcepharadione B | COc1cc2C(=O)C(=O)Nc3c2c(c1OC)c1ccccc1c3 | 189168 |
| IMPHY003485 | Myrcene | C=CC(=C)CCC=C(C)C | 31253 |
| IMPHY004192 | Piperine | O=C(N1CCCCC1)/C=C/C=C/c1ccc2c(c1)OCO2 | 638024 |
| IMPHY004341 | Piperlonguminine | CC(CNC(=O)/C=C/C=C/c1ccc2c(c1)OCO2)C | 5320621 |
| IMPHY005260 | Aristolodione | COc1c(O)cc2c3c1c1ccccc1cc3N(C(=O)C2=O)C | 184116 |
| IMPHY005360 | Aristolactam BII | COc1c(OC)cc2c3c1c1ccccc1cc3NC2=O | 162739 |
| IMPHY006342 | Piperlongumine | COc1cc(/C=C/C(=O)N2CCC=CC2=O)cc(c1OC)OC | 637858 |
| IMPHY006970 | Decanal | CCCCCCCCCC=O | 8175 |
| IMPHY006992 | 1-Methylhexyl acetate | CCCCCC(OC(=O)C)C | 80018 |
| IMPHY007017 | Methyl 3,4,5-trimethoxycinnamate | COC(=O)/C=C/c1cc(OC)c(c(c1)OC)OC | 735846 |
| IMPHY007204 | Dodecanal | CCCCCCCCCCCC=O | 8194 |
| IMPHY007371 | Cepharadione A | O=C1N(C)c2cc3ccccc3c3c2c(C1=O)cc1c3OCO1 | 94577 |
| IMPHY007376 | beta-Cubebene | CC([C@@H]1CC[C@H]([C@]23[C@H]1[C@H]2C(=C)CC3)C)C | 93081 |
| IMPHY007528 | Cadinane | C[C@H]1CC[C@@H]2[C@@H](C1)[C@@H](CC[C@@H]2C)C(C)C | 9548708 |
| IMPHY009368 | Heptadecane | CCCCCCCCCCCCCCCCC | 12398 |
| IMPHY009389 | Pentadecane | CCCCCCCCCCCCCCC | 12391 |
| IMPHY009413 | Triacontane | CCCCCCCCCCCCCCCCCCCCCCCCCCCCCC | 12535 |
| IMPHY009419 | Tridecane | CCCCCCCCCCCCC | 12388 |
| IMPHY009642 | 2-Nonanone | CCCCCCCC(=O)C | 13187 |
| IMPHY010072 | Eucalyptol | CC12CCC(CC1)C(O2)(C)C | 2758 |
| IMPHY010080 | beta-Elemene | C=C[C@]1(C)CC[C@H](C[C@H]1C(=C)C)C(=C)C | 6918391 |
| IMPHY011519 | alpha-Terpinyl acetate | CC(=O)OC(C1CCC(=CC1)C)(C)C | 111037 |
| IMPHY011521 | 2-Undecanone | CCCCCCCCCC(=O)C | 8163 |
| IMPHY011542 | beta-Eudesmol | C=C1CCC[C@]2([C@H]1C[C@@H](CC2)C(O)(C)C)C | 91457 |
| IMPHY011599 | Terpinolene | CC1=CCC(=C(C)C)CC1 | 11463 |
| IMPHY011641 | (+)-alpha-Gurjunene | C[C@H]1CC[C@H]2[C@@H](C3=C(CC[C@@H]13)C)C2(C)C | 15560275 |
| IMPHY011659 | alpha-Muurolene | CC1=C[C@@H]2[C@H](CC1)C(=CC[C@H]2C(C)C)C | 12306047 |
| IMPHY011709 | alpha-Eudesmol | CC1=CCC[C@]2([C@H]1C[C@@H](CC2)C(O)(C)C)C | 92762 |
| IMPHY011761 | Humulene | C/C/1=CCC(C)(C)/C=C/C/C(=C/CC1)/C | 5281520 |
| IMPHY011792 | gamma-Muurolene | CC1=C[C@@H]2[C@H](CC1)C(=C)CC[C@H]2C(C)C | 12313020 |
| IMPHY011839 | (Z)-gamma-bisabolene | CC(=CCC/C(=C1/CCC(=CC1)C)/C)C | 3033866 |
| IMPHY011890 | Elemol | C=C[C@]1(C)CC[C@H](C[C@H]1C(=C)C)C(O)(C)C | 92138 |
| IMPHY011965 | (+)-beta-Phellandrene | CC([C@@H]1CCC(=C)C=C1)C | 442484 |
| IMPHY012058 | Linalool | C=CC(CCC=C(C)C)(O)C | 6549 |
| IMPHY012061 | alpha-Pinene | CC1=CCC2CC1C2(C)C | 6654 |
| IMPHY012147 | beta-Pinene | C=C1CCC2CC1C2(C)C | 14896 |
| IMPHY012160 | alpha-Terpineol | CC1=CCC(CC1)C(O)(C)C | 17100 |
| IMPHY012305 | beta-Patchoulene | CC1CCC2=C1CC1CCC2(C1(C)C)C | 101731 |
| IMPHY012585 | delta-Cadinol | CC1=C[C@@H]2[C@H](CC1)[C@](C)(O)CC[C@H]2C(C)C | 3084311 |
| IMPHY012586 | (-)-alpha-Cadinol | CC1=CC2C(CC1)[C@@](C)(O)CC[C@@H]2C(C)C | 6431302 |
| IMPHY012615 | 9-Eicosyne | CCCCCCCCCCC#CCCCCCCCC | 557019 |
| IMPHY012667 | Caryophyllene oxide | C=C1CC[C@H]2O[C@@]2(CC[C@@H]2[C@@H]1CC2(C)C)C | 1742210 |
| IMPHY012679 | Piperolactam A | COc1cc2C(=O)Nc3c2c(c1O)c1ccccc1c3 | 3081016 |
| IMPHY012739 | (Z)-beta-Ocimene | C=C/C(=CCC=C(C)C)/C | 5320250 |
| IMPHY012921 | gamma-Elemene | C=C[C@]1(C)CCC(=C(C)C)C[C@H]1C(=C)C | 6432312 |
| IMPHY013093 | delta-Elemene | C=C[C@@]1(C)CCC(=C[C@@H]1C(=C)C)C(C)C | 12309449 |
| IMPHY014811 | alpha-Phellandrene | CC1=CCC(C=C1)C(C)C | 7460 |
| IMPHY014831 | beta-Caryophyllene | C/C/1=CCCC(=C)[C@@H]2[C@@H](CC1)C(C2)(C)C | 5281515 |
| IMPHY014835 | (E)-beta-ocimene | C=C/C(=C/CC=C(C)C)/C | 5281553 |
| IMPHY014847 | Bornyl acetate | CC(=O)OC1CC2C(C1(C)CC2)(C)C | 6448 |
| IMPHY014852 | Camphene | C=C1C2CCC(C1(C)C)C2 | 6616 |
| IMPHY014881 | Copaene | CC(C1CCC2(C3C1C2C(=CC3)C)C)C | 19725 |
| IMPHY014895 | 5-[3-(1,3-Benzodioxol-5-yl)-1,3,3a,4,6,6a-hexahydrofuro[3,4-c]furan-6-yl]-1,3-benzodioxole | C1Oc2c(O1)cc(cc2)C1OCC2C1COC2c1ccc2c(c1)OCO2 | 5204 |
| IMPHY014988 | Limonene | CC1=CCC(CC1)C(=C)C | 22311 |
| IMPHY015022 | Nerolidol | C=CC(CC/C=C(/CCC=C(C)C)C)(O)C | 5284507 |
| IMPHY011462 | Sylvatine | CC(CCC/C=C/CCCCNC(=O)/C=C/C=C/c1ccc2c(c1)OCO2)C | 90472536 |
| IMPHY012967 | Diaeudesmin | COc1cc(ccc1OC)[C@@H]1OC[C@H]2[C@@H]1CO[C@H]2c1ccc(c(c1)OC)OC | 6992053 |
| IMPHY014895 | 5-[3-(1,3-Benzodioxol-5-yl)-1,3,3a,4,6,6a-hexahydrofuro[3,4-c]furan-6-yl]-1,3-benzodioxole | C1Oc2c(O1)cc(cc2)C1OCC2C1COC2c1ccc2c(c1)OCO2 | 5204 |
| IMPHY001881 | 2-Tridecanone | CCCCCCCCCCCC(=O)C | 11622 |
| IMPHY003053 | 1-Pentadecene | CCCCCCCCCCCCCC=C | 25913 |
| IMPHY003485 | Myrcene | C=CC(=C)CCC=C(C)C | 31253 |
| IMPHY004192 | Piperine | O=C(N1CCCCC1)/C=C/C=C/c1ccc2c(c1)OCO2 | 638024 |
| IMPHY004341 | Piperlonguminine | CC(CNC(=O)/C=C/C=C/c1ccc2c(c1)OCO2)C | 5320621 |
| IMPHY006342 | Piperlongumine | COc1cc(/C=C/C(=O)N2CCC=CC2=O)cc(c1OC)OC | 637858 |
| IMPHY006970 | Decanal | CCCCCCCCCC=O | 8175 |
| IMPHY006992 | 1-Methylhexyl acetate | CCCCCC(OC(=O)C)C | 80018 |
| IMPHY007204 | Dodecanal | CCCCCCCCCCCC=O | 8194 |
| IMPHY007376 | beta-Cubebene | CC([C@@H]1CC[C@H]([C@]23[C@H]1[C@H]2C(=C)CC3)C)C | 93081 |
| IMPHY007528 | Cadinane | C[C@H]1CC[C@@H]2[C@@H](C1)[C@@H](CC[C@@H]2C)C(C)C | 9548708 |
| IMPHY009368 | Heptadecane | CCCCCCCCCCCCCCCCC | 12398 |
| IMPHY009389 | Pentadecane | CCCCCCCCCCCCCCC | 12391 |
| IMPHY009419 | Tridecane | CCCCCCCCCCCCC | 12388 |
| IMPHY009642 | 2-Nonanone | CCCCCCCC(=O)C | 13187 |
| IMPHY010072 | Eucalyptol | CC12CCC(CC1)C(O2)(C)C | 2758 |
| IMPHY010080 | beta-Elemene | C=C[C@]1(C)CC[C@H](C[C@H]1C(=C)C)C(=C)C | 6918391 |
| IMPHY011519 | alpha-Terpinyl acetate | CC(=O)OC(C1CCC(=CC1)C)(C)C | 111037 |
| IMPHY011521 | 2-Undecanone | CCCCCCCCCC(=O)C | 8163 |
| IMPHY011542 | beta-Eudesmol | C=C1CCC[C@]2([C@H]1C[C@@H](CC2)C(O)(C)C)C | 91457 |
| IMPHY011599 | Terpinolene | CC1=CCC(=C(C)C)CC1 | 11463 |
| IMPHY011641 | (+)-alpha-Gurjunene | C[C@H]1CC[C@H]2[C@@H](C3=C(CC[C@@H]13)C)C2(C)C | 15560275 |
| IMPHY011659 | alpha-Muurolene | CC1=C[C@@H]2[C@H](CC1)C(=CC[C@H]2C(C)C)C | 12306047 |
| IMPHY011709 | alpha-Eudesmol | CC1=CCC[C@]2([C@H]1C[C@@H](CC2)C(O)(C)C)C | 92762 |
| IMPHY011761 | Humulene | C/C/1=CCC(C)(C)/C=C/C/C(=C/CC1)/C | 5281520 |
| IMPHY011792 | gamma-Muurolene | CC1=C[C@@H]2[C@H](CC1)C(=C)CC[C@H]2C(C)C | 12313020 |
| IMPHY011839 | (Z)-gamma-bisabolene | CC(=CCC/C(=C1/CCC(=CC1)C)/C)C | 3033866 |
| IMPHY011890 | Elemol | C=C[C@]1(C)CC[C@H](C[C@H]1C(=C)C)C(O)(C)C | 92138 |
| IMPHY011965 | (+)-beta-Phellandrene | CC([C@@H]1CCC(=C)C=C1)C | 442484 |
| IMPHY012058 | Linalool | C=CC(CCC=C(C)C)(O)C | 6549 |
| IMPHY012061 | alpha-Pinene | CC1=CCC2CC1C2(C)C | 6654 |
| IMPHY012147 | beta-Pinene | C=C1CCC2CC1C2(C)C | 14896 |
| IMPHY012160 | alpha-Terpineol | CC1=CCC(CC1)C(O)(C)C | 17100 |
| IMPHY012305 | beta-Patchoulene | CC1CCC2=C1CC1CCC2(C1(C)C)C | 101731 |
| IMPHY012585 | delta-Cadinol | CC1=C[C@@H]2[C@H](CC1)[C@](C)(O)CC[C@H]2C(C)C | 3084311 |
| IMPHY012586 | (-)-alpha-Cadinol | CC1=CC2C(CC1)[C@@](C)(O)CC[C@@H]2C(C)C | 6431302 |
| IMPHY012615 | 9-Eicosyne | CCCCCCCCCCC#CCCCCCCCC | 557019 |
| IMPHY012667 | Caryophyllene oxide | C=C1CC[C@H]2O[C@@]2(CC[C@@H]2[C@@H]1CC2(C)C)C | 1742210 |
| IMPHY012739 | (Z)-beta-Ocimene | C=C/C(=CCC=C(C)C)/C | 5320250 |
| IMPHY012921 | gamma-Elemene | C=C[C@]1(C)CCC(=C(C)C)C[C@H]1C(=C)C | 6432312 |
| IMPHY013093 | delta-Elemene | C=C[C@@]1(C)CCC(=C[C@@H]1C(=C)C)C(C)C | 12309449 |
| IMPHY014811 | alpha-Phellandrene | CC1=CCC(C=C1)C(C)C | 7460 |
| IMPHY014831 | beta-Caryophyllene | C/C/1=CCCC(=C)[C@@H]2[C@@H](CC1)C(C2)(C)C | 5281515 |
| IMPHY014835 | (E)-beta-ocimene | C=C/C(=C/CC=C(C)C)/C | 5281553 |
| IMPHY014847 | Bornyl acetate | CC(=O)OC1CC2C(C1(C)CC2)(C)C | 6448 |
| IMPHY014852 | Camphene | C=C1C2CCC(C1(C)C)C2 | 6616 |
| IMPHY014881 | Copaene | CC(C1CCC2(C3C1C2C(=CC3)C)C)C | 19725 |
| IMPHY014988 | Limonene | CC1=CCC(CC1)C(=C)C | 22311 |
| IMPHY015022 | Nerolidol | C=CC(CC/C=C(/CCC=C(C)C)C)(O)C | 5284507 |
| IMPHY000399 | beta-Bisabolene | CC(=CCCC(=C)[C@H]1CCC(=CC1)C)C | 10104370 |
| IMPHY001313 | Piperundecalidine | O=C(N1CCCCC1)/C=C/C=C/CCCC/C=C/c1ccc2c(c1)OCO2 | 44453654 |
| IMPHY003328 | Cepharadione B | COc1cc2C(=O)C(=O)N(c3c2c(c1OC)c1ccccc1c3)C | 189151 |
| IMPHY003329 | Norcepharadione B | COc1cc2C(=O)C(=O)Nc3c2c(c1OC)c1ccccc1c3 | 189168 |
| IMPHY004192 | Piperine | O=C(N1CCCCC1)/C=C/C=C/c1ccc2c(c1)OCO2 | 638024 |
| IMPHY004217 | Chavicine | O=C(N1CCCCC1)/C=CC=C/c1ccc2c(c1)OCO2 | 1548912 |
| IMPHY004341 | Piperlonguminine | CC(CNC(=O)/C=C/C=C/c1ccc2c(c1)OCO2)C | 5320621 |
| IMPHY005360 | Aristolactam BII | COc1c(OC)cc2c3c1c1ccccc1cc3NC2=O | 162739 |
| IMPHY005815 | Tetrahydropiperine | O=C(N1CCCCC1)CCCCc1ccc2c(c1)OCO2 | 581676 |
| IMPHY005944 | Pipercide | CC(CNC(=O)/C=C/C=C/CCCC/C=C/c1ccc2c(c1)OCO2)C | 5372162 |
| IMPHY006266 | Guineensine | CC(CNC(=O)/C=C/C=C/CCCCCC/C=C/c1ccc2c(c1)OCO2)C | 6442405 |
| IMPHY006342 | Piperlongumine | COc1cc(/C=C/C(=O)N2CCC=CC2=O)cc(c1OC)OC | 637858 |
| IMPHY006624 | Pluviatilol | COc1cc(ccc1O)[C@H]1OC[C@@H]2[C@H]1CO[C@H]2c1ccc2c(c1)OCO2 | 70695727 |
| IMPHY007269 | Trichostachine | O=C(N1CCCC1)/C=C/C=C/c1ccc2c(c1)OCO2 | 636537 |
| IMPHY007371 | Cepharadione A | O=C1N(C)c2cc3ccccc3c3c2c(C1=O)cc1c3OCO1 | 94577 |
| IMPHY007559 | Pipernonaline | O=C(N1CCCCC1)/C=C/CCCC/C=C/c1ccc2c(c1)OCO2 | 9974595 |
| IMPHY009389 | Pentadecane | CCCCCCCCCCCCCCC | 12391 |
| IMPHY011593 | Pellitorine | CCCCC/C=C/C=C/C(=O)NCC(C)C | 5318516 |
| IMPHY012058 | Linalool | C=CC(CCC=C(C)C)(O)C | 6549 |
| IMPHY012165 | Sabinene | C=C1CCC2(C1C2)C(C)C | 18818 |
| IMPHY013823 | Bamipine | CN1CCC(CC1)N(c1ccccc1)Cc1ccccc1 | 72075 |
| IMPHY014811 | alpha-Phellandrene | CC1=CCC(C=C1)C(C)C | 7460 |
| IMPHY014831 | beta-Caryophyllene | C/C/1=CCCC(=C)[C@@H]2[C@@H](CC1)C(C2)(C)C | 5281515 |
| IMPHY014836 | beta-Sitosterol | CC[C@@H](C(C)C)CC[C@H]([C@H]1CC[C@@H]2[C@]1(C)CC[C@H]1[C@H]2CC=C2[C@]1(C)CC[C@@H](C2)O)C | 222284 |
| IMPHY014895 | 5-[3-(1,3-Benzodioxol-5-yl)-1,3,3a,4,6,6a-hexahydrofuro[3,4-c]furan-6-yl]-1,3-benzodioxole | C1Oc2c(O1)cc(cc2)C1OCC2C1COC2c1ccc2c(c1)OCO2 | 5204 |
| IMPHY014988 | Limonene | CC1=CCC(CC1)C(=C)C | 22311 |
| IMPHY015047 | Rutin | Oc1cc(O)c2c(c1)oc(c(c2=O)O[C@@H]1O[C@H](CO[C@@H]2O[C@@H](C)[C@@H]([C@H]([C@H]2O)O)O)[C@H]([C@@H]([C@H]1O)O)O)c1ccc(c(c1)O)O | 5280805 |
|  |  |  |  |
|  |  |  |  |
|  |  |  |  |
|  |  |  |  |
|  |  |  |  |
|  |  |  |  |
| ***12. Plant name : Piper nigrum*** | | | |
| **IMPPAT Phytochemical identifier:** | **Phytochemical name:** | **SMILES:** | **CID** |
| IMPHY000022 | Myrcenol | C=CC(=C)CCCC(O)(C)C | CID: 10975 |
| IMPHY000027 | (E)-Piperolein A | O=C(N1CCCCC1)CCCC/C=C/c1ccc2c(c1)OCO2 | CID: 11141599 |
| IMPHY000099 | Myrtenol | OCC1=CCC2CC1C2(C)C | CID: 10582 |
| IMPHY000399 | beta-Bisabolene | CC(=CCCC(=C)[C@H]1CCC(=CC1)C)C | CID: 10104370 |
| IMPHY000402 | 1,4-Cineole | CC(C12CCC(O2)(CC1)C)C | CID: 10106 |
| IMPHY000491 | Pinene | CC1CCC2CC1C2(C)C | CID: 10129 |
| IMPHY000602 | M-Cymene | Cc1cccc(c1)C(C)C | CID: 10812 |
| IMPHY000711 | Bornylene | CC1(C)C2CCC1(C)C=C2 | CID: 10047 |
| IMPHY001316 | (2E,4E,6E)-7-(2H-1,3-benzodioxol-5-yl)-1-(piperidin-1-yl)hepta-2,4,6-trien-1-one | O=C(N1CCCCC1)/C=C/C=C/C=C/c1ccc2c(c1)OCO2 | CID: 10244953 |
| IMPHY001351 | Elemicin | C=CCc1cc(OC)c(c(c1)OC)OC | CID: 10248 |
| IMPHY002263 | Pipericide | CC(NC(=O)/C=C/C=C/CCCC/C=C/c1ccc2c(c1)OCO2)C | CID: 101422868 |
| IMPHY002825 | 2-(4-Methylphenyl)propan-2-ol | Cc1ccc(cc1)C(O)(C)C | CID: 14529 |
| IMPHY002915 | Benzyl Alcohol | OCc1ccccc1 | CID: 244 |
| IMPHY003398 | Myristicin | C=CCc1cc(OC)c2c(c1)OCO2 | CID: 4276 |
| IMPHY003485 | Myrcene | C=CC(=C)CCC=C(C)C | CID: 31253 |
| IMPHY003513 | Isoamyl alcohol | OCCC(C)C | CID: 31260 |
| IMPHY003525 | Nonanal | CCCCCCCCC=O | CID: 31289 |
| IMPHY003536 | Eugenol | C=CCc1ccc(c(c1)OC)O | CID: 3314 |
| IMPHY003616 | Bicyclogermacrene | C/C/1=CCC/C(=C/[C@H]2[C@@H](CC1)C2(C)C)/C | CID: 13894537 |
| IMPHY003694 | Germacrene a | C/C/1=CCC/C(=C/C[C@@H](CC1)C(=C)C)/C | CID: 9548705 |
| IMPHY003719 | beta-Copaene | CC([C@@H]1CC[C@]2([C@@H]3[C@H]1C2C(=C)CC3)C)C | CID: 57339298 |
| IMPHY003798 | (Z)-alpha-Bisabolene | CC(=CC/C=C(C1CCC(=CC1)C)/C)C | CID: 5352653 |
| IMPHY003807 | (E)-alpha-bisabolene | CC(=CC/C=C(/C1CCC(=CC1)C)C)C | CID: 5315468 |
| IMPHY003955 | (R)-4-Isopropylcyclohex-2-enone | CC([C@H]1CCC(=O)C=C1)C | CID: 642520 |
| IMPHY003956 | (+)-gamma-Gurjunene | C[C@@H]1CC[C@H]2C1=C[C@@H](CC[C@H]2C)C(=C)C | CID: 15560285 |
| IMPHY003962 | beta-Guaiene | CC(=C1CC[C@@H](C2=C(C1)[C@@H](C)CC2)C)C | CID: 15560252 |
| IMPHY003977 | (-)-beta-Bourbonene | CC([C@@H]1CC[C@@]2([C@H]1[C@H]1C(=C)CC[C@@H]21)C)C | CID: 62566 |
| IMPHY003982 | gamma-Terpinene | CC1=CCC(=CC1)C(C)C | CID: 7461 |
| IMPHY004022 | Tricyclo(6.3.1.02,5)dodecan-1-ol, 4,4,8-trimethyl-, (1R,2S,5R,8S)- | C[C@]12CCC[C@](C2)(O)[C@@H]2[C@@H](CC1)C(C2)(C)C | CID: 11746218 |
| IMPHY004135 | Cyclohexene, 6-ethenyl-6-methyl-1-(1-methylethyl)-3-(1-methylethylidene)-, (S)- | C=C[C@@]1(C)CCC(=C(C)C)C=C1C(C)C | CID: 11019992 |
| IMPHY004151 | Geranyl formate | O=COC/C=C(/CCC=C(C)C)C | CID: 5282109 |
| IMPHY004184 | Cinnamyl acetate | CC(=O)OC/C=C/c1ccccc1 | CID: 5282110 |
| IMPHY004192 | Piperine | O=C(N1CCCCC1)/C=C/C=C/c1ccc2c(c1)OCO2 | CID: 638024 |
| IMPHY004217 | Chavicine | O=C(N1CCCCC1)/C=CC=C/c1ccc2c(c1)OCO2 | CID: 1548912 |
| IMPHY004536 | Geranic acid | CC(=CCC/C(=C/C(=O)O)/C)C | CID: 5275520 |
| IMPHY004646 | Vomifoliol | C[C@H](/C=C/[C@@]1(O)C(=CC(=O)CC1(C)C)C)O | CID: 5280462 |
| IMPHY004889 | Cinnamyl alcohol | OC/C=C/c1ccccc1 | CID: 5315892 |
| IMPHY005345 | 1-Octen-3-OL | CCCCCC(C=C)O | CID: 18827 |
| IMPHY005653 | 2-Octanol | CCCCCCC(O)C | CID: 20083 |
| IMPHY005720 | Piperolein B | O=C(N1CCCCC1)CCCCCC/C=C/c1ccc2c(c1)OCO2 | CID: 21580213 |
| IMPHY005836 | Methyl caffeic acid | OC(=O)/C(=C/c1ccc(c(c1)O)O)/C | CID: 55255958 |
| IMPHY005944 | Pipercide | CC(CNC(=O)/C=C/C=C/CCCC/C=C/c1ccc2c(c1)OCO2)C | CID: 5372162 |
| IMPHY006007 | Citronellyl formate | O=COCCC(CCC=C(C)C)C | CID: 7778 |
| IMPHY006145 | p-Cymene | Cc1ccc(cc1)C(C)C | CID: 7463 |
| IMPHY006266 | Guineensine | CC(CNC(=O)/C=C/C=C/CCCCCC/C=C/c1ccc2c(c1)OCO2)C | CID: 6442405 |
| IMPHY006324 | Linalyl propionate | CCC(=O)OC(CCC=C(C)C)(C=C)C | CID: 61098 |
| IMPHY006414 | Geranyl tiglate | C/C=C(/C(=O)OC/C=C(/CCC=C(C)C)C)C | CID: 5367785 |
| IMPHY006516 | 3,4-Methylenedioxycinnamic acid | OC(=O)/C=C/c1ccc2c(c1)OCO2 | CID: 643181 |
| IMPHY006550 | Thymol | Cc1ccc(c(c1)O)C(C)C | CID: 6989 |
| IMPHY006696 | Methyleugenol | C=CCc1ccc(c(c1)OC)OC | CID: 7127 |
| IMPHY006719 | (1aR,7S,7aS,7bR)-1,1,4,7-tetramethyl-2,3,5,6,7a,7b-hexahydro-1aH-cyclopropa[h]azulen-7-ol | CC1=C2CC[C@]([C@H]2[C@H]2[C@@H](CC1)C2(C)C)(C)O | CID: 102303030 |
| IMPHY006932 | Cuparene | Cc1ccc(cc1)[C@]1(C)CCCC1(C)C | CID: 86895 |
| IMPHY006944 | Estragole | COc1ccc(cc1)CC=C | CID: 8815 |
| IMPHY006948 | beta-Terpineol | CC(=C)C1CCC(CC1)(C)O | CID: 8748 |
| IMPHY006965 | alpha,alpha-Dimethyl-4-methylenecyclohexanemethanol | CC(C1CCC(=C)CC1)(O)C | CID: 81722 |
| IMPHY007055 | Piperonal | O=Cc1ccc2c(c1)OCO2 | CID: 8438 |
| IMPHY007171 | 1-Hexanol | CCCCCCO | CID: 8103 |
| IMPHY007188 | Piperidine | C1CCCNC1 | CID: 8082 |
| IMPHY007202 | Nonane | CCCCCCCCC | CID: 8141 |
| IMPHY007269 | Trichostachine | O=C(N1CCCC1)/C=C/C=C/c1ccc2c(c1)OCO2 | CID: 636537 |
| IMPHY007307 | (1S,2S,3S,6S)-3-ethenyl-3,7,7-trimethyl-2-prop-1-en-2-ylbicyclo[4.1.0]heptane | C=C[C@]1(C)CC[C@H]2[C@@H]([C@H]1C(=C)C)C2(C)C | CID: 91746561 |
| IMPHY007327 | Palmitic acid | CCCCCCCCCCCCCCCC(=O)O | CID: 985 |
| IMPHY007376 | beta-Cubebene | CC([C@@H]1CC[C@H]([C@]23[C@H]1[C@H]2C(=C)CC3)C)C | CID: 93081 |
| IMPHY007421 | Citronellyl acetate | CC(CCC=C(C)C)CCOC(=O)C | CID: 9017 |
| IMPHY007445 | (Z)-beta-Ocimenol | C=C/C(=CC(C=C(C)C)O)/C | CID: 91753567 |
| IMPHY007528 | Cadinane | C[C@H]1CC[C@@H]2[C@@H](C1)[C@@H](CC[C@@H]2C)C(C)C | CID: 9548708 |
| IMPHY007840 | Spathulenol | C=C1CC[C@@H]2[C@H]([C@H]3[C@H]1CC[C@]3(C)O)C2(C)C | CID: 92231 |
| IMPHY007852 | Azulene | c1ccc2-c(cc1)ccc2 | CID: 9231 |
| IMPHY008146 | 1-Pentanol | CCCCCO | CID: 6276 |
| IMPHY008303 | 1-Hepten-3-OL | CCCCC(C=C)O | CID: 21057 |
| IMPHY008773 | (2E,4E)-N-(2-methylpropyl)octadeca-2,4-dienamide | CCCCCCCCCCCCC/C=C/C=C/C(=O)NCC(C)C | CID: 9974234 |
| IMPHY008936 | alpha-Guaiene | CC(=C)[C@@H]1CC[C@@H](C2=C(C1)[C@@H](C)CC2)C | CID: 5317844 |
| IMPHY008946 | delta-Guaiene | CC(=C)[C@@H]1CCC(=C2[C@@H](C1)[C@@H](C)CC2)C | CID: 94275 |
| IMPHY009604 | Dihydroferuperine | COC1=CC(=CCC1O)/C=CC=CC(=O)N1CCCCC1 | CID: 131752910 |
| IMPHY009605 | Feruperine | COc1cc(/C=CC=CC(=O)N2CCCCC2)ccc1O | CID: 131752909 |
| IMPHY009618 | Zingiberenol | CC(=CCCC(C1CCC(C=C1)(C)O)C)C | CID: 13213649 |
| IMPHY009626 | 2-Nonanol | CCCCCCCC(O)C | CID: 12367 |
| IMPHY009840 | Cyclosativene | CC(C1CCC2(C3C1C1C(C21C)C3)C)C | CID: 519960 |
| IMPHY009853 | Naphthalene | c1ccc2c(c1)cccc2 | CID: 931 |
| IMPHY009946 | Benzaldehyde | O=Cc1ccccc1 | CID: 240 |
| IMPHY009966 | Eucarvone | CC1=CC=CC(CC1=O)(C)C | CID: 136330 |
| IMPHY010072 | Eucalyptol | CC12CCC(CC1)C(O2)(C)C | CID: 2758 |
| IMPHY010080 | beta-Elemene | C=C[C@]1(C)CC[C@H](C[C@H]1C(=C)C)C(=C)C | CID: 6918391 |
| IMPHY010097 | Benzyl benzoate | O=C(c1ccccc1)OCc1ccccc1 | CID: 2345 |
| IMPHY010603 | beta-Cadinene | CC1=CC[C@@H]2[C@@H](C1)[C@@H](CC=C2C)C(C)C | CID: 10657 |
| IMPHY010986 | 2-Methyl cinnamyl alcohol | OCC=Cc1ccccc1C | CID: 54031279 |
| IMPHY011050 | 3-Buten-2-OL | CC(C=C)O | CID: 11716 |
| IMPHY011058 | 3-Cyclohexen-1-ol, 4-methyl-1-(1-methylethyl)-, acetate | CC(=O)OC1(CCC(=CC1)C)C(C)C | CID: 20960 |
| IMPHY011392 | 3-Carene | CC1=CCC2C(C1)C2(C)C | CID: 26049 |
| IMPHY011396 | 4-Carvomenthenol | CC1=CCC(CC1)(O)C(C)C | CID: 11230 |
| IMPHY011409 | Guaiacol | COc1ccccc1O | CID: 460 |
| IMPHY011519 | alpha-Terpinyl acetate | CC(=O)OC(C1CCC(=CC1)C)(C)C | CID: 111037 |
| IMPHY011542 | beta-Eudesmol | C=C1CCC[C@]2([C@H]1C[C@@H](CC2)C(O)(C)C)C | CID: 91457 |
| IMPHY011552 | (1R)-2-methyl-5-propan-2-ylbicyclo[3.1.0]hex-2-ene | CC1=CCC2([C@@H]1C2)C(C)C | CID: 6451618 |
| IMPHY011557 | 4-Isopropylbenzyl alcohol | OCc1ccc(cc1)C(C)C | CID: 325 |
| IMPHY011562 | 2-Hexenal | CCC/C=C/C=O | CID: 5281168 |
| IMPHY011581 | alpha-Selinene | CC1=CCC[C@]2([C@H]1C[C@@H](CC2)C(=C)C)C | CID: 10856614 |
| IMPHY011586 | (S,1Z,6Z)-8-Isopropyl-1-methyl-5-methylenecyclodeca-1,6-diene | C/C/1=C/CCC(=C)/C=C[C@@H](CC1)C(C)C | CID: 91723653 |
| IMPHY011588 | cis-3-Hexen-1-ol | OCC/C=CCC | CID: 5281167 |
| IMPHY011590 | d-Borneol | O[C@@H]1C[C@H]2C([C@@]1(C)CC2)(C)C | CID: 61060 |
| IMPHY011593 | Pellitorine | CCCCC/C=C/C=C/C(=O)NCC(C)C | CID: 5318516 |
| IMPHY011599 | Terpinolene | CC1=CCC(=C(C)C)CC1 | CID: 11463 |
| IMPHY011630 | cis,cis-Farnesol | OC/C=C(CC/C=C(CCC=C(C)C)/C)/C | CID: 1549107 |
| IMPHY011631 | (E,Z)-farnesol | OC/C=C(/CC/C=C(CCC=C(C)C)/C)C | CID: 1549109 |
| IMPHY011632 | Farnesol | OC/C=C(/CC/C=C(/CCC=C(C)C)C)C | CID: 445070 |
| IMPHY011633 | (2Z,6E)-Farnesol | OC/C=C(CC/C=C(/CCC=C(C)C)C)/C | CID: 1549108 |
| IMPHY011643 | alpha-Terpinene | CC1=CC=C(CC1)C(C)C | CID: 7462 |
| IMPHY011647 | Geranyl acetate | C/C(=CCOC(=O)C)/CCC=C(C)C | CID: 1549026 |
| IMPHY011648 | Neryl acetate | C/C(=C/COC(=O)C)/CCC=C(C)C | CID: 1549025 |
| IMPHY011658 | beta-Farnesene | C=CC(=C)CC/C=C(/CCC=C(C)C)C | CID: 5281517 |
| IMPHY011659 | alpha-Muurolene | CC1=C[C@@H]2[C@H](CC1)C(=CC[C@H]2C(C)C)C | CID: 12306047 |
| IMPHY011667 | alpha-Gurjunene | C[C@@H]1CC[C@@H]2[C@H](C3=C(CC[C@H]13)C)C2(C)C | CID: 15560276 |
| IMPHY011709 | alpha-Eudesmol | CC1=CCC[C@]2([C@H]1C[C@@H](CC2)C(O)(C)C)C | CID: 92762 |
| IMPHY011745 | Zingiberene | CC(=CCC[C@@H]([C@H]1CC=C(C=C1)C)C)C | CID: 92776 |
| IMPHY011749 | Humulene epoxide II | C/C/1=CCC(C)(C)/C=C/C[C@@]2([C@@H](CC1)O2)C | CID: 10704181 |
| IMPHY011761 | Humulene | C/C/1=CCC(C)(C)/C=C/C/C(=C/CC1)/C | CID: 5281520 |
| IMPHY011789 | Citral | O=C/C=C(/CCC=C(C)C)C | CID: 638011 |
| IMPHY011790 | Neral | O=C/C=C(CCC=C(C)C)/C | CID: 643779 |
| IMPHY011792 | gamma-Muurolene | CC1=C[C@@H]2[C@H](CC1)C(=C)CC[C@H]2C(C)C | CID: 12313020 |
| IMPHY011793 | (+)-gamma-Cadinene | CC1=C[C@@H]2[C@@H](CC1)C(=C)CC[C@H]2C(C)C | CID: 6432404 |
| IMPHY011817 | alpha-Farnesene | C=C/C(=C/C/C=C(/CCC=C(C)C)C)/C | CID: 5281516 |
| IMPHY011872 | (4E,7E)-1,5,9,9-Tetramethyl-12-oxabicyclo[9.1.0]dodeca-4,7-diene | C/C/1=C/CCC2(C)OC2CC(/C=CC1)(C)C | CID: 22559443 |
| IMPHY011879 | Moupinamide | COc1cc(/C=C/C(=O)NCCc2ccc(cc2)O)ccc1O | CID: 5280537 |
| IMPHY011882 | Cinnamaldehyde | O=C/C=C/c1ccccc1 | CID: 637511 |
| IMPHY011890 | Elemol | C=C[C@]1(C)CC[C@H](C[C@H]1C(=C)C)C(O)(C)C | CID: 92138 |
| IMPHY011901 | Thujone | O=C1C[C@]2([C@@H]([C@H]1C)C2)C(C)C | CID: 261491 |
| IMPHY011902 | beta-Thujone | O=C1C[C@]2([C@@H]([C@@H]1C)C2)C(C)C | CID: 91456 |
| IMPHY011938 | gamma-Eudesmol | CC1=C2C[C@@H](CC[C@]2(CCC1)C)C(O)(C)C | CID: 6432005 |
| IMPHY011939 | 10-epi-gamma-Eudesmol | CC1=C2C[C@@H](CC[C@@]2(CCC1)C)C(O)(C)C | CID: 6430754 |
| IMPHY011957 | (+)-delta-Cadinene | CC1=C[C@@H]2C(=C(C)CC[C@H]2C(C)C)CC1 | CID: 441005 |
| IMPHY011965 | (+)-beta-Phellandrene | CC([C@@H]1CCC(=C)C=C1)C | CID: 442484 |
| IMPHY011973 | (-)-cis-Carveol | CC(=C)[C@@H]1CC=C([C@@H](C1)O)C | CID: 330573 |
| IMPHY011983 | cis-Cinnamaldehyde | O=C/C=Cc1ccccc1 | CID: 6428995 |
| IMPHY011988 | (-)-trans-Carveol | CC(=C)[C@@H]1CC=C([C@H](C1)O)C | CID: 94221 |
| IMPHY012036 | Camphor | O=C1CC2C(C1(C)CC2)(C)C | CID: 2537 |
| IMPHY012058 | Linalool | C=CC(CCC=C(C)C)(O)C | CID: 6549 |
| IMPHY012061 | alpha-Pinene | CC1=CCC2CC1C2(C)C | CID: 6654 |
| IMPHY012086 | Citronellal | O=CCC(CCC=C(C)C)C | CID: 7794 |
| IMPHY012095 | Cyclohexene | C1CCC=CC1 | CID: 8079 |
| IMPHY012104 | Citronellol | OCCC(CCC=C(C)C)C | CID: 8842 |
| IMPHY012107 | Adamantane | C1C2CC3CC1CC(C2)C3 | CID: 9238 |
| IMPHY012130 | Dihydrocarveol | CC(=C)C1CCC(C(C1)O)C | CID: 12072 |
| IMPHY012147 | beta-Pinene | C=C1CCC2CC1C2(C)C | CID: 14896 |
| IMPHY012160 | alpha-Terpineol | CC1=CCC(CC1)C(O)(C)C | CID: 17100 |
| IMPHY012165 | Sabinene | C=C1CCC2(C1C2)C(C)C | CID: 18818 |
| IMPHY012168 | (1S,2S,6S,7R,8R)-1,3-dimethyl-8-propan-2-yltricyclo[4.4.0.02,7]dec-3-ene | CC([C@H]1CC[C@]2([C@@H]3[C@@H]1[C@H]2C(=CC3)C)C)C | CID: 101607926 |
| IMPHY012178 | p-Menthan-3-one | CC1CCC(C(=O)C1)C(C)C | CID: 6986 |
| IMPHY012279 | alpha-Curcumene | CC(=CCCC(c1ccc(cc1)C)C)C | CID: 92139 |
| IMPHY012305 | beta-Patchoulene | CC1CCC2=C1CC1CCC2(C1(C)C)C | CID: 101731 |
| IMPHY012585 | delta-Cadinol | CC1=C[C@@H]2[C@H](CC1)[C@](C)(O)CC[C@H]2C(C)C | CID: 3084311 |
| IMPHY012586 | (-)-alpha-Cadinol | CC1=CC2C(CC1)[C@@](C)(O)CC[C@@H]2C(C)C | CID: 6431302 |
| IMPHY012589 | 3-(1,5-Dimethyl-4-hexenyl)-6-methylene-1-cyclohexene | CC(C1CCC(=C)C=C1)CCC=C(C)C | CID: 519764 |
| IMPHY012654 | Nerol | OC/C=C(CCC=C(C)C)/C | CID: 643820 |
| IMPHY012667 | Caryophyllene oxide | C=C1CC[C@H]2O[C@@]2(CC[C@@H]2[C@@H]1CC2(C)C)C | CID: 1742210 |
| IMPHY012739 | (Z)-beta-Ocimene | C=C/C(=CCC=C(C)C)/C | CID: 5320250 |
| IMPHY012823 | 1,6-Cyclodecadiene | C1C/C=CCCC/C=CC1 | CID: 5365639 |
| IMPHY012920 | 2-Furanmethanol, 5-ethenyltetrahydro-alpha,alpha,5-trimethyl-, cis- | C=C[C@@]1(C)CC[C@H](O1)C(O)(C)C | CID: 11116492 |
| IMPHY012921 | gamma-Elemene | C=C[C@]1(C)CCC(=C(C)C)C[C@H]1C(=C)C | CID: 6432312 |
| IMPHY013093 | delta-Elemene | C=C[C@@]1(C)CCC(=C[C@@H]1C(=C)C)C(C)C | CID: 12309449 |
| IMPHY013133 | (Z)-p-Menth-2-en-1-ol | CC([C@@H]1CC[C@](C=C1)(C)O)C | CID: 13918681 |
| IMPHY014161 | Coumaperine | Oc1ccc(cc1)/C=C/C=C/C(=O)N1CCCCC1 | CID: 10131321 |
| IMPHY014708 | beta-Selinene | C=C1CCC[C@]2([C@H]1C[C@@H](CC2)C(=C)C)C | CID: 442393 |
| IMPHY014801 | Zizanene | CC1=C[C@@H]2[C@H](CC1)C(=CC[C@@H]2C(C)C)C | CID: 12306046 |
| IMPHY014806 | Caswell No. 264AB | CC([C@@H]1CC[C@H]([C@]23[C@H]1[C@H]2C(=CC3)C)C)C | CID: 442359 |
| IMPHY014811 | alpha-Phellandrene | CC1=CCC(C=C1)C(C)C | CID: 7460 |
| IMPHY014817 | Aromadendrene | CC1CCC2C1C1C(C1(C)C)CCC2=C | CID: 91354 |
| IMPHY014831 | beta-Caryophyllene | C/C/1=CCCC(=C)[C@@H]2[C@@H](CC1)C(C2)(C)C | CID: 5281515 |
| IMPHY014835 | (E)-beta-ocimene | C=C/C(=C/CC=C(C)C)/C | CID: 5281553 |
| IMPHY014852 | Camphene | C=C1C2CCC(C1(C)C)C2 | CID: 6616 |
| IMPHY014857 | Cedrol | C[C@@H]1CC[C@@H]2[C@@]31CC[C@@]([C@H](C3)C2(C)C)(C)O | CID: 65575 |
| IMPHY014871 | cis-Nerolidol | C=CC(CC/C=C(CCC=C(C)C)/C)(O)C | CID: 5320128 |
| IMPHY014881 | Copaene | CC(C1CCC2(C3C1C2C(=CC3)C)C)C | CID: 19725 |
| IMPHY014884 | Cubebene | CC([C@H]1CC[C@@H](C23[C@@H]1C2C(=CC3)C)C)C | CID: 91747196 |
| IMPHY014906 | Cedrelanol | CC1=C[C@@H]2[C@@H](CC1)[C@@](C)(O)CC[C@H]2C(C)C | CID: 160799 |
| IMPHY014907 | 6-Epi-beta-bisabolol | CC(=CCC[C@@H]([C@@]1(O)CCC(=CC1)C)C)C | CID: 12300148 |
| IMPHY014923 | Geraniol | OC/C=C(/CCC=C(C)C)C | CID: 637566 |
| IMPHY014988 | Limonene | CC1=CCC(CC1)C(=C)C | CID: 22311 |
| IMPHY014989 | trans-Linalool oxide | C=C[C@]1(C)CC[C@H](O1)C(O)(C)C | CID: 6432254 |
| IMPHY015003 | Menthol | CC1CCC(C(C1)O)C(C)C | CID: 1254 |
| IMPHY015004 | Menthone | C[C@@H]1CC[C@H](C(=O)C1)C(C)C | CID: 26447 |
| IMPHY015016 | alpha-Muurolol | CC1=C[C@@H]2[C@H](CC1)[C@](C)(O)CC[C@@H]2C(C)C | CID: 91753440 |
| IMPHY015022 | Nerolidol | C=CC(CC/C=C(/CCC=C(C)C)C)(O)C | CID: 5284507 |
| IMPHY015042 | Piperitone | CC1=CC(=O)C(CC1)C(C)C | CID: 6987 |
| IMPHY015094 | (+)-trans-Limonene oxide | CC(=C)[C@@H]1CC[C@]2([C@@H](C1)O2)C | CID: 449290 |
| IMPHY015095 | 2-Cyclohexen-1-ol, 1-methyl-4-(1-methylethyl)-, trans- | CC([C@@H]1CC[C@@](C=C1)(C)O)C | CID: 122484 |
| IMPHY015123 | alpha-Copaene | CC([C@@H]1CC[C@]2([C@@H]3[C@H]1C2C(=CC3)C)C)C | CID: 70678558 |
| IMPHY016027 | trans-Sabinene hydrate | CC([C@@]12CC[C@](C2C1)(C)O)C | CID: 12315151 |
| IMPHY016054 | trans-alpha-Bergamotene | CC(=CCC[C@]1(C)[C@H]2CC=C([C@@H]1C2)C)C | CID: 6429302 |
| IMPHY000022 | Myrcenol | C=CC(=C)CCCC(O)(C)C | CID: 10975 |
| IMPHY000399 | beta-Bisabolene | CC(=CCCC(=C)[C@H]1CCC(=CC1)C)C | CID: 10104370 |
| IMPHY000402 | 1,4-Cineole | CC(C12CCC(O2)(CC1)C)C | CID: 10106 |
| IMPHY001246 | Carvacrol | CC(c1ccc(c(c1)O)C)C | CID: 10364 |
| IMPHY001351 | Elemicin | C=CCc1cc(OC)c(c(c1)OC)OC | CID: 10248 |
| IMPHY001516 | Decane | CCCCCCCCCC | CID: 15600 |
| IMPHY001912 | Cubebin | O[C@H]1OC[C@@H]([C@H]1Cc1ccc2c(c1)OCO2)Cc1ccc2c(c1)OCO2 | CID: 117443 |
| IMPHY003296 | Piperitenone | CC1=CC(=O)C(=C(C)C)CC1 | CID: 381152 |
| IMPHY003485 | Myrcene | C=CC(=C)CCC=C(C)C | CID: 31253 |
| IMPHY003719 | beta-Copaene | CC([C@@H]1CC[C@]2([C@@H]3[C@H]1C2C(=C)CC3)C)C | CID: 57339298 |
| IMPHY003822 | Cubebol | CC([C@@H]1CC[C@H]([C@]23[C@H]1[C@H]2[C@@](C)(O)CC3)C)C | CID: 11276107 |
| IMPHY003977 | (-)-beta-Bourbonene | CC([C@@H]1CC[C@@]2([C@H]1[C@H]1C(=C)CC[C@@H]21)C)C | CID: 62566 |
| IMPHY003982 | gamma-Terpinene | CC1=CCC(=CC1)C(C)C | [CID:7461](cid:7461) |
| IMPHY004067 | Dihydrocarvyl acetate | CC(=O)OC1CC(CCC1C)C(=C)C | [CID:30248](https://pubchem.ncbi.nlm.nih.gov/compound/30248) |
| IMPHY004281 | Guaiol | C[C@H]1CC[C@H](CC2=C1CC[C@@H]2C)C(O)(C)C | [CID:227829](https://pubchem.ncbi.nlm.nih.gov/compound/227829) |
| IMPHY004549 | Safrole | C=CCc1ccc2c(c1)OCO2 | [CID:5144](https://pubchem.ncbi.nlm.nih.gov/compound/5144) |
| IMPHY005521 | 1-Hexen-3-OL | CCCC(C=C)O | [CID:20928](cid:20928) |
| IMPHY005618 | Germacrene B | C/C/1=CCC/C(=C/CC(=C(C)C)CC1)/C | [CID:5281519](https://pubchem.ncbi.nlm.nih.gov/compound/5281519) |
| IMPHY006145 | p-Cymene | Cc1ccc(cc1)C(C)C | [CID:7463](https://pubchem.ncbi.nlm.nih.gov/compound/7463) |
| IMPHY006177 | Methyl geranate | COC(=O)/C=C(/CCC=C(C)C)C | [CID:5365910](https://pubchem.ncbi.nlm.nih.gov/compound/5365910) |
| IMPHY006550 | Thymol | Cc1ccc(c(c1)O)C(C)C | [CID:6989](https://pubchem.ncbi.nlm.nih.gov/compound/6989) |
| IMPHY006696 | Methyleugenol | C=CCc1ccc(c(c1)OC)OC | [CID:7127](https://pubchem.ncbi.nlm.nih.gov/compound/7127) |
| IMPHY006948 | beta-Terpineol | CC(=C)C1CCC(CC1)(C)O | [CID:8748](https://pubchem.ncbi.nlm.nih.gov/compound/8748) |
| IMPHY007041 | Furfural | O=Cc1ccco1 | [CID:7362](https://pubchem.ncbi.nlm.nih.gov/compound/7362) |
| IMPHY007376 | beta-Cubebene | CC([C@@H]1CC[C@H]([C@]23[C@H]1[C@H]2C(=C)CC3)C)C | [CID:93081](cid:93081) |
| IMPHY007421 | Citronellyl acetate | CC(CCC=C(C)C)CCOC(=O)C | [CID:9017](https://pubchem.ncbi.nlm.nih.gov/compound/9017) |
| IMPHY007840 | Spathulenol | C=C1CC[C@@H]2[C@H]([C@H]3[C@H]1CC[C@]3(C)O)C2(C)C | [CID:92231](https://pubchem.ncbi.nlm.nih.gov/compound/92231) |
| IMPHY008150 | 1-Methyl-4-(prop-1-en-2-yl)benzene | Cc1ccc(cc1)C(=C)C | [CID:62385](cid:62385) |
| IMPHY008303 | 1-Hepten-3-OL | CCCCC(C=C)O | [CID:21057](https://pubchem.ncbi.nlm.nih.gov/compound/21057) |
| IMPHY008369 | Methyl isobutyl ketone | CC(CC(=O)C)C | [CID:7909](https://pubchem.ncbi.nlm.nih.gov/compound/7909) |
| IMPHY008936 | alpha-Guaiene | CC(=C)[C@@H]1CC[C@@H](C2=C(C1)[C@@H](C)CC2)C | [CID:5317844](https://pubchem.ncbi.nlm.nih.gov/compound/5317844) |
| IMPHY008946 | delta-Guaiene | CC(=C)[C@@H]1CCC(=C2[C@@H](C1)[C@@H](C)CC2)C | [CID:94275](https://pubchem.ncbi.nlm.nih.gov/compound/94275) |
| IMPHY009642 | 2-Nonanone | CCCCCCCC(=O)C | [CID:13187](https://pubchem.ncbi.nlm.nih.gov/compound/13187) |
| IMPHY010080 | beta-Elemene | C=C[C@]1(C)CC[C@H](C[C@H]1C(=C)C)C(=C)C | [CID:6918391](https://pubchem.ncbi.nlm.nih.gov/compound/6918391) |
| IMPHY011002 | 3-Methyl-2-pentanol | CCC(C(O)C)C | [CID:11261](https://pubchem.ncbi.nlm.nih.gov/compound/11261) |
| IMPHY011100 | alpha-Cedrene epoxide | CC1CCC2C31CC1OC1(C(C3)C2(C)C)C | [CID:122510](https://pubchem.ncbi.nlm.nih.gov/compound/122510) |
| IMPHY011392 | 3-Carene | CC1=CCC2C(C1)C2(C)C | [CID:26049](https://pubchem.ncbi.nlm.nih.gov/compound/26049) |
| IMPHY011396 | 4-Carvomenthenol | CC1=CCC(CC1)(O)C(C)C | [CID:11230](https://pubchem.ncbi.nlm.nih.gov/compound/11230) |
| IMPHY011455 | Cadina-1,4-diene | CC1=CC2C(=CC1)[C@@H](C)CCC2C(C)C | [CID:6427091](https://pubchem.ncbi.nlm.nih.gov/compound/6427091) |
| IMPHY011519 | alpha-Terpinyl acetate | CC(=O)OC(C1CCC(=CC1)C)(C)C | [CID:111037](https://pubchem.ncbi.nlm.nih.gov/compound/111037) |
| IMPHY011521 | 2-Undecanone | CCCCCCCCCC(=O)C | [CID:8163](https://pubchem.ncbi.nlm.nih.gov/compound/8163) |
| IMPHY011542 | beta-Eudesmol | C=C1CCC[C@]2([C@H]1C[C@@H](CC2)C(O)(C)C)C | [CID:91457](https://pubchem.ncbi.nlm.nih.gov/compound/91457) |
| IMPHY011552 | (1R)-2-methyl-5-propan-2-ylbicyclo[3.1.0]hex-2-ene | CC1=CCC2([C@@H]1C2)C(C)C | [CID:6451618](https://pubchem.ncbi.nlm.nih.gov/compound/6451618) |
| IMPHY011581 | alpha-Selinene | CC1=CCC[C@]2([C@H]1C[C@@H](CC2)C(=C)C)C | [CID:10856614](https://pubchem.ncbi.nlm.nih.gov/compound/10856614) |
| IMPHY011586 | (S,1Z,6Z)-8-Isopropyl-1-methyl-5-methylenecyclodeca-1,6-diene | C/C/1=C/CCC(=C)/C=C[C@@H](CC1)C(C)C | [CID:91723653](https://pubchem.ncbi.nlm.nih.gov/compound/91723653) |
| IMPHY011588 | cis-3-Hexen-1-ol | OCC/C=CCC | [CID:5281167](https://pubchem.ncbi.nlm.nih.gov/compound/5281167) |
| IMPHY011590 | d-Borneol | O[C@@H]1C[C@H]2C([C@@]1(C)CC2)(C)C | [CID:61060](https://pubchem.ncbi.nlm.nih.gov/compound/61060) |
| IMPHY011599 | Terpinolene | CC1=CCC(=C(C)C)CC1 | [CID:11463](https://pubchem.ncbi.nlm.nih.gov/compound/11463) |
| IMPHY011614 | beta-Cedrene | C=C1CC[C@@]23C[C@@H]1C(C)(C)[C@@H]2CC[C@H]3C | [CID:11106485](cid:11106485) |
| IMPHY011630 | cis,cis-Farnesol | OC/C=C(CC/C=C(CCC=C(C)C)/C)/C | [CID:1549107](https://pubchem.ncbi.nlm.nih.gov/compound/1549107) |
| IMPHY011631 | (E,Z)-farnesol | OC/C=C(/CC/C=C(CCC=C(C)C)/C)C | [CID:1549109](https://pubchem.ncbi.nlm.nih.gov/compound/1549109) |
| IMPHY011632 | Farnesol | OC/C=C(/CC/C=C(/CCC=C(C)C)C)C | [CID:445070](https://pubchem.ncbi.nlm.nih.gov/compound/445070) |
| IMPHY011633 | (2Z,6E)-Farnesol | OC/C=C(CC/C=C(/CCC=C(C)C)C)/C | [CID:1549108](https://pubchem.ncbi.nlm.nih.gov/compound/1549108) |
| IMPHY011643 | alpha-Terpinene | CC1=CC=C(CC1)C(C)C | [CID:7462](https://pubchem.ncbi.nlm.nih.gov/compound/7462) |
| IMPHY011659 | alpha-Muurolene | CC1=C[C@@H]2[C@H](CC1)C(=CC[C@H]2C(C)C)C | [CID:12306047](https://pubchem.ncbi.nlm.nih.gov/compound/12306047) |
| IMPHY011761 | Humulene | C/C/1=CCC(C)(C)/C=C/C/C(=C/CC1)/C | [CID:5281520](https://pubchem.ncbi.nlm.nih.gov/compound/5281520) |
| IMPHY011789 | Citral | O=C/C=C(/CCC=C(C)C)C | [CID:638011](https://pubchem.ncbi.nlm.nih.gov/compound/638011) |
| IMPHY011790 | Neral | O=C/C=C(CCC=C(C)C)/C | [CID:643779](cid:643779) |
| IMPHY011890 | Elemol | C=C[C@]1(C)CC[C@H](C[C@H]1C(=C)C)C(O)(C)C | [CID:92138](https://pubchem.ncbi.nlm.nih.gov/compound/92138) |
| IMPHY011957 | (+)-delta-Cadinene | CC1=C[C@@H]2C(=C(C)CC[C@H]2C(C)C)CC1 | [CID:441005](cid:441005) |
| IMPHY011965 | (+)-beta-Phellandrene | CC([C@@H]1CCC(=C)C=C1)C | [CID:442484](https://pubchem.ncbi.nlm.nih.gov/compound/442484) |
| IMPHY011988 | (-)-trans-Carveol | CC(=C)[C@@H]1CC=C([C@H](C1)O)C | [CID:94221](https://pubchem.ncbi.nlm.nih.gov/compound/94221) |
| IMPHY012036 | Camphor | O=C1CC2C(C1(C)CC2)(C)C | [CID:2537](cid:2537) |
| IMPHY012058 | Linalool | C=CC(CCC=C(C)C)(O)C | [CID:6549](https://pubchem.ncbi.nlm.nih.gov/compound/6549) |
| IMPHY012061 | alpha-Pinene | CC1=CCC2CC1C2(C)C | [CID:6654](https://pubchem.ncbi.nlm.nih.gov/compound/6654) |
| IMPHY012086 | Citronellal | O=CCC(CCC=C(C)C)C | [CID:7794](https://pubchem.ncbi.nlm.nih.gov/compound/7794) |
| IMPHY012104 | Citronellol | OCCC(CCC=C(C)C)C | [CID:8842](https://pubchem.ncbi.nlm.nih.gov/compound/8842) |
| IMPHY012147 | beta-Pinene | C=C1CCC2CC1C2(C)C | [CID:14896](https://pubchem.ncbi.nlm.nih.gov/compound/14896) |
| IMPHY012160 | alpha-Terpineol | CC1=CCC(CC1)C(O)(C)C | [CID:17100](https://pubchem.ncbi.nlm.nih.gov/compound/17100) |
| IMPHY012165 | Sabinene | C=C1CCC2(C1C2)C(C)C | [CID:18818](https://pubchem.ncbi.nlm.nih.gov/compound/18818) |
| IMPHY012168 | (1S,2S,6S,7R,8R)-1,3-dimethyl-8-propan-2-yltricyclo[4.4.0.02,7]dec-3-ene | CC([C@H]1CC[C@]2([C@@H]3[C@@H]1[C@H]2C(=CC3)C)C)C | [CID:101607926](https://pubchem.ncbi.nlm.nih.gov/compound/101607926) |
| IMPHY012255 | (+)-trans-Piperitenol | CC1=C[C@@H]([C@H](CC1)C(C)C)O | [CID:85568](https://pubchem.ncbi.nlm.nih.gov/compound/85568) |
| IMPHY012261 | alpha-Bergamotene | CC(=CCCC1(C)C2CC=C(C1C2)C)C | [CID:86608](https://pubchem.ncbi.nlm.nih.gov/compound/86608) |
| IMPHY012279 | alpha-Curcumene | CC(=CCCC(c1ccc(cc1)C)C)C | [CID:92139](https://pubchem.ncbi.nlm.nih.gov/compound/92139) |
| IMPHY012586 | (-)-alpha-Cadinol | CC1=CC2C(CC1)[C@@](C)(O)CC[C@@H]2C(C)C | [CID:6431302](https://pubchem.ncbi.nlm.nih.gov/compound/6431302) |
| IMPHY012654 | Nerol | OC/C=C(CCC=C(C)C)/C | [CID:643820](https://pubchem.ncbi.nlm.nih.gov/compound/643820) |
| IMPHY012667 | Caryophyllene oxide | C=C1CC[C@H]2O[C@@]2(CC[C@@H]2[C@@H]1CC2(C)C)C | [CID:1742210](https://pubchem.ncbi.nlm.nih.gov/compound/1742210) |
| IMPHY012910 | trans-Calamenene | CC([C@H]1CC[C@@H](c2c1cc(C)cc2)C)C | [CID:6429022](https://pubchem.ncbi.nlm.nih.gov/compound/6429022) |
| IMPHY013080 | alpha-Calacorene | CC([C@@H]1CC=C(c2c1cc(C)cc2)C)C | [CID:12302243](https://pubchem.ncbi.nlm.nih.gov/compound/12302243) |
| IMPHY013093 | delta-Elemene | C=C[C@@]1(C)CCC(=C[C@@H]1C(=C)C)C(C)C | [CID:12309449](https://pubchem.ncbi.nlm.nih.gov/compound/12309449) |
| IMPHY013836 | Fenchone | O=C1C2(C)CCC(C1(C)C)C2 | [CID:14525](https://pubchem.ncbi.nlm.nih.gov/compound/14525) |
| IMPHY013838 | 3,7-Dimethyloct-6-en-3-ol | CCC(CCC=C(C)C)(O)C | [CID:86749](https://pubchem.ncbi.nlm.nih.gov/compound/86749) |
| IMPHY014690 | (-)-Globulol | C[C@@H]1CC[C@@H]2[C@@H]1[C@H]1[C@H](C1(C)C)CC[C@@]2(C)O | [CID:12304985](cid:12304985) |
| IMPHY014708 | beta-Selinene | C=C1CCC[C@]2([C@H]1C[C@@H](CC2)C(=C)C)C | [CID:442393](https://pubchem.ncbi.nlm.nih.gov/compound/442393) |
| IMPHY014805 | Cedr-8-ene | CC1=CC[C@@]23C[C@@H]1C(C)(C)[C@@H]2CC[C@H]3C | [CID:6431015](https://pubchem.ncbi.nlm.nih.gov/compound/6431015) |
| IMPHY014806 | Caswell No. 264AB | CC([C@@H]1CC[C@H]([C@]23[C@H]1[C@H]2C(=CC3)C)C)C | [CID:442359](cid:442359) |
| IMPHY014811 | alpha-Phellandrene | CC1=CCC(C=C1)C(C)C | [CID:7460](https://pubchem.ncbi.nlm.nih.gov/compound/7460) |
| IMPHY014831 | beta-Caryophyllene | C/C/1=CCCC(=C)[C@@H]2[C@@H](CC1)C(C2)(C)C | [CID:5281515](https://pubchem.ncbi.nlm.nih.gov/compound/5281515) |
| IMPHY014835 | (E)-beta-ocimene | C=C/C(=C/CC=C(C)C)/C | [CID:5281553](https://pubchem.ncbi.nlm.nih.gov/compound/5281553) |
| IMPHY014847 | Bornyl acetate | CC(=O)OC1CC2C(C1(C)CC2)(C)C | [CID:6448](https://pubchem.ncbi.nlm.nih.gov/compound/6448) |
| IMPHY014852 | Camphene | C=C1C2CCC(C1(C)C)C2 | [CID:6616](https://pubchem.ncbi.nlm.nih.gov/compound/6616) |
| IMPHY014857 | Cedrol | C[C@@H]1CC[C@@H]2[C@@]31CC[C@@]([C@H](C3)C2(C)C)(C)O | [CID:65575](https://pubchem.ncbi.nlm.nih.gov/compound/65575) |
| IMPHY014865 | Calamenene | CC([C@@H]1CC[C@@H](c2c1cc(C)cc2)C)C | [CID:6429077](https://pubchem.ncbi.nlm.nih.gov/compound/6429077) |
| IMPHY014874 | cis-Sabinene hydrate | C[C@@H]1CC[C@@]2(C1C2)C(C)C | [CID:101629835](https://pubchem.ncbi.nlm.nih.gov/compound/101629835) |
| IMPHY014907 | 6-Epi-beta-bisabolol | CC(=CCC[C@@H]([C@@]1(O)CCC(=CC1)C)C)C | [CID:12300148](https://pubchem.ncbi.nlm.nih.gov/compound/12300148) |
| IMPHY014914 | Fenchol | OC1C2(C)CCC(C1(C)C)C2 | [CID:15406](https://pubchem.ncbi.nlm.nih.gov/compound/15406) |
| IMPHY014923 | Geraniol | OC/C=C(/CCC=C(C)C)C | [CID:637566](https://pubchem.ncbi.nlm.nih.gov/compound/637566) |
| IMPHY014988 | Limonene | CC1=CCC(CC1)C(=C)C | [CID:22311](https://pubchem.ncbi.nlm.nih.gov/compound/22311) |
| IMPHY014989 | trans-Linalool oxide | C=C[C@]1(C)CC[C@H](O1)C(O)(C)C | [CID:6432254](https://pubchem.ncbi.nlm.nih.gov/compound/6432254) |
| IMPHY015062 | (+)-cis-Sabinol | CC([C@]12C[C@@H]2C(=C)[C@@H](C1)O)C | [CID:94147](https://pubchem.ncbi.nlm.nih.gov/compound/94147) |
| IMPHY015063 | Sabinyl acetate | CC(=O)OC1CC2(C(C1=C)C2)C(C)C | [CID:94266](https://pubchem.ncbi.nlm.nih.gov/compound/94266) |
| IMPHY015123 | alpha-Copaene | CC([C@@H]1CC[C@]2([C@@H]3[C@H]1C2C(=CC3)C)C)C | [CID:70678558](https://pubchem.ncbi.nlm.nih.gov/compound/70678558) |
| IMPHY015128 | T-Muurolol | CC1=C[C@@H]2[C@H](CC1)[C@@](C)(O)CC[C@H]2C(C)C | [CID:3084331](https://pubchem.ncbi.nlm.nih.gov/compound/3084331) |
| IMPHY015380 | 4-Methyl-2-pentanol | CC(CC(O)C)C | [CID:7910](https://pubchem.ncbi.nlm.nih.gov/compound/7910) |
| IMPHY016012 | Allo-Aromadendrene | C[C@@H]1CC[C@H]2[C@@H]1C1C(C1(C)C)CCC2=C | [CID:42608158](https://pubchem.ncbi.nlm.nih.gov/compound/42608158) |
| IMPHY017044 | Dihydroocimene | CCC(/C=C/C=C(C)C)C | [CID:129640025](https://pubchem.ncbi.nlm.nih.gov/compound/129640025) |
| IMPHY017124 | beta Farnesene | CCC(=C)CC/C=C(/CCC=C(C)C)C | [CID:15228937](https://pubchem.ncbi.nlm.nih.gov/compound/15228937) |
| IMPHY017645 | Cadina-1,4-diene-3-ol | CC(C1CCC(C2=CC(C(=CC12)C)O)C)C | [CID:6431196](https://pubchem.ncbi.nlm.nih.gov/compound/6431196) |
| IMPHY000491 | Pinene | CC1CCC2CC1C2(C)C | 10129 |
| IMPHY002915 | Benzyl Alcohol | OCc1ccccc1 | [CID:244](https://pubchem.ncbi.nlm.nih.gov/compound/244) |
| IMPHY003694 | Germacrene a | C/C/1=CCC/C(=C/C[C@@H](CC1)C(=C)C)/C | [CID:9548705](https://pubchem.ncbi.nlm.nih.gov/compound/9548705) |
| IMPHY004135 | Cyclohexene, 6-ethenyl-6-methyl-1-(1-methylethyl)-3-(1-methylethylidene)-, (S)- | C=C[C@@]1(C)CCC(=C(C)C)C=C1C(C)C | [CID:11019992](https://pubchem.ncbi.nlm.nih.gov/compound/11019992) |
| IMPHY007840 | Spathulenol | C=C1CC[C@@H]2[C@H]([C@H]3[C@H]1CC[C@]3(C)O)C2(C)C | [CID:92231](https://pubchem.ncbi.nlm.nih.gov/compound/92231) |
| IMPHY007852 | Azulene | c1ccc2-c(cc1)ccc2 | [CID:9231](https://pubchem.ncbi.nlm.nih.gov/compound/9231) |
| IMPHY009853 | Naphthalene | c1ccc2c(c1)cccc2 | [CID:931](https://pubchem.ncbi.nlm.nih.gov/compound/931) |
| IMPHY009966 | Eucarvone | CC1=CC=CC(CC1=O)(C)C | [CID:136330](https://pubchem.ncbi.nlm.nih.gov/compound/136330) |
| IMPHY011392 | 3-Carene | CC1=CCC2C(C1)C2(C)C | [CID:26049](https://pubchem.ncbi.nlm.nih.gov/compound/26049) |
| IMPHY011643 | alpha-Terpinene | CC1=CC=C(CC1)C(C)C | [CID:7462](https://pubchem.ncbi.nlm.nih.gov/compound/7462) |
| IMPHY011761 | Humulene | C/C/1=CCC(C)(C)/C=C/C/C(=C/CC1)/C | [CID:5281520](https://pubchem.ncbi.nlm.nih.gov/compound/5281520) |
| IMPHY012061 | alpha-Pinene | CC1=CCC2CC1C2(C)C | [CID:6654](https://pubchem.ncbi.nlm.nih.gov/compound/6654) |
| IMPHY012095 | Cyclohexene | C1CCC=CC1 | [CID:8079](https://pubchem.ncbi.nlm.nih.gov/compound/8079) |
| IMPHY012107 | Adamantane | C1C2CC3CC1CC(C2)C3 | [CID:9238](https://pubchem.ncbi.nlm.nih.gov/compound/9238) |
| IMPHY012147 | beta-Pinene | C=C1CCC2CC1C2(C)C | [CID:14896](https://pubchem.ncbi.nlm.nih.gov/compound/14896) |
| IMPHY012667 | Caryophyllene oxide | C=C1CC[C@H]2O[C@@]2(CC[C@@H]2[C@@H]1CC2(C)C)C | [CID:1742210](https://pubchem.ncbi.nlm.nih.gov/compound/1742210) |
| IMPHY012823 | 1,6-Cyclodecadiene | C1C/C=CCCC/C=CC1 | [CID:5365639](https://pubchem.ncbi.nlm.nih.gov/compound/5365639) |
| IMPHY014806 | Caswell No. 264AB | CC([C@@H]1CC[C@H]([C@]23[C@H]1[C@H]2C(=CC3)C)C)C | [CID:442359](https://pubchem.ncbi.nlm.nih.gov/compound/442359) |
| IMPHY014831 | beta-Caryophyllene | C/C/1=CCCC(=C)[C@@H]2[C@@H](CC1)C(C2)(C)C | [CID:5281515](https://pubchem.ncbi.nlm.nih.gov/compound/5281515) |
| IMPHY014881 | Copaene | CC(C1CCC2(C3C1C2C(=CC3)C)C)C | [CID:19725](https://pubchem.ncbi.nlm.nih.gov/compound/19725) |
| IMPHY014988 | Limonene | CC1=CCC(CC1)C(=C)C | [CID:22311](https://pubchem.ncbi.nlm.nih.gov/compound/22311) |
| IMPHY003485 | Myrcene | C=CC(=C)CCC=C(C)C | [CID:31253](https://pubchem.ncbi.nlm.nih.gov/compound/31253) |
| IMPHY003982 | gamma-Terpinene | CC1=CCC(=CC1)C(C)C | [CID:7461](https://pubchem.ncbi.nlm.nih.gov/compound/7461) |
| IMPHY004192 | Piperine | O=C(N1CCCCC1)/C=C/C=C/c1ccc2c(c1)OCO2 | [CID:638024](https://pubchem.ncbi.nlm.nih.gov/compound/638024) |
| IMPHY005618 | Germacrene B | C/C/1=CCC/C(=C/CC(=C(C)C)CC1)/C | [CID:5281519](https://pubchem.ncbi.nlm.nih.gov/compound/5281519) |
| IMPHY006145 | p-Cymene | Cc1ccc(cc1)C(C)C | [CID:7463](https://pubchem.ncbi.nlm.nih.gov/compound/7463) |
| IMPHY006177 | Methyl geranate | COC(=O)/C=C(/CCC=C(C)C)C | [CID:5365910](https://pubchem.ncbi.nlm.nih.gov/compound/5365910) |
| IMPHY007840 | Spathulenol | C=C1CC[C@@H]2[C@H]([C@H]3[C@H]1CC[C@]3(C)O)C2(C)C | [CID:92231](https://pubchem.ncbi.nlm.nih.gov/compound/92231) |
| IMPHY008150 | 1-Methyl-4-(prop-1-en-2-yl)benzene | Cc1ccc(cc1)C(=C)C | [CID:62385](https://pubchem.ncbi.nlm.nih.gov/compound/62385) |
| IMPHY008910 | Hentriacontane | CCCCCCCCCCCCCCCCCCCCCCCCCCCCCCC | [CID:12410](https://pubchem.ncbi.nlm.nih.gov/compound/12410) |
| IMPHY008930 | 16-Hentriacontanone | CCCCCCCCCCCCCCCC(=O)CCCCCCCCCCCCCCC | [CID:94741](https://pubchem.ncbi.nlm.nih.gov/compound/94741) |
| IMPHY008936 | alpha-Guaiene | CC(=C)[C@@H]1CC[C@@H](C2=C(C1)[C@@H](C)CC2)C | [CID:5317844](https://pubchem.ncbi.nlm.nih.gov/compound/5317844) |
| IMPHY008946 | delta-Guaiene | CC(=C)[C@@H]1CCC(=C2[C@@H](C1)[C@@H](C)CC2)C | [CID:94275](cid:94275) |
| IMPHY009642 | 2-Nonanone | CCCCCCCC(=O)C | [CID:13187](https://pubchem.ncbi.nlm.nih.gov/compound/13187) |
| IMPHY010080 | beta-Elemene | C=C[C@]1(C)CC[C@H](C[C@H]1C(=C)C)C(=C)C | [CID:6918391](https://pubchem.ncbi.nlm.nih.gov/compound/6918391) |
| IMPHY011392 | 3-Carene | CC1=CCC2C(C1)C2(C)C | [CID:26049](https://pubchem.ncbi.nlm.nih.gov/compound/26049) |
| IMPHY011396 | 4-Carvomenthenol | CC1=CCC(CC1)(O)C(C)C | [CID:11230](cid:11230) |
| IMPHY011455 | Cadina-1,4-diene | CC1=CC2C(=CC1)[C@@H](C)CCC2C(C)C | [CID:6427091](https://pubchem.ncbi.nlm.nih.gov/compound/6427091) |
| IMPHY011521 | 2-Undecanone | CCCCCCCCCC(=O)C | [CID:8163](https://pubchem.ncbi.nlm.nih.gov/compound/8163) |
| IMPHY011586 | (S,1Z,6Z)-8-Isopropyl-1-methyl-5-methylenecyclodeca-1,6-diene | C/C/1=C/CCC(=C)/C=C[C@@H](CC1)C(C)C | [CID:91723653](https://pubchem.ncbi.nlm.nih.gov/compound/91723653) |
| IMPHY011590 | d-Borneol | O[C@@H]1C[C@H]2C([C@@]1(C)CC2)(C)C | [CID:61060](https://pubchem.ncbi.nlm.nih.gov/compound/61060) |
| IMPHY011599 | Terpinolene | CC1=CCC(=C(C)C)CC1 | [CID:11463](https://pubchem.ncbi.nlm.nih.gov/compound/11463) |
| IMPHY011643 | alpha-Terpinene | CC1=CC=C(CC1)C(C)C | [CID:7462](https://pubchem.ncbi.nlm.nih.gov/compound/7462) |
| IMPHY011761 | Humulene | C/C/1=CCC(C)(C)/C=C/C/C(=C/CC1)/C | [CID:5281520](https://pubchem.ncbi.nlm.nih.gov/compound/5281520) |
| IMPHY011789 | Citral | O=C/C=C(/CCC=C(C)C)C | [CID:638011](https://pubchem.ncbi.nlm.nih.gov/compound/638011) |
| IMPHY011790 | Neral | O=C/C=C(CCC=C(C)C)/C | [CID:643779](https://pubchem.ncbi.nlm.nih.gov/compound/643779) |
| IMPHY011890 | Elemol | C=C[C@]1(C)CC[C@H](C[C@H]1C(=C)C)C(O)(C)C | [CID:92138](https://pubchem.ncbi.nlm.nih.gov/compound/92138) |
| IMPHY011957 | (+)-delta-Cadinene | CC1=C[C@@H]2C(=C(C)CC[C@H]2C(C)C)CC1 | [CID:441005](cid:441005) |
| IMPHY011965 | (+)-beta-Phellandrene | CC([C@@H]1CCC(=C)C=C1)C | [CID:442484](https://pubchem.ncbi.nlm.nih.gov/compound/442484) |
| IMPHY011988 | (-)-trans-Carveol | CC(=C)[C@@H]1CC=C([C@H](C1)O)C | [CID:94221](https://pubchem.ncbi.nlm.nih.gov/compound/94221) |
| IMPHY012036 | Camphor | O=C1CC2C(C1(C)CC2)(C)C | [CID:2537](https://pubchem.ncbi.nlm.nih.gov/compound/2537) |
| IMPHY012058 | Linalool | C=CC(CCC=C(C)C)(O)C | [CID:6549](https://pubchem.ncbi.nlm.nih.gov/compound/6549) |
| IMPHY012061 | alpha-Pinene | CC1=CCC2CC1C2(C)C | [CID:6654](https://pubchem.ncbi.nlm.nih.gov/compound/6654) |
| IMPHY012086 | Citronellal | O=CCC(CCC=C(C)C)C | [CID:7794](https://pubchem.ncbi.nlm.nih.gov/compound/7794) |
| IMPHY012104 | Citronellol | OCCC(CCC=C(C)C)C | [CID:8842](https://pubchem.ncbi.nlm.nih.gov/compound/8842) |
| IMPHY012147 | beta-Pinene | C=C1CCC2CC1C2(C)C | [CID:14896](https://pubchem.ncbi.nlm.nih.gov/compound/14896) |
| IMPHY012160 | alpha-Terpineol | CC1=CCC(CC1)C(O)(C)C | [CID:17100](https://pubchem.ncbi.nlm.nih.gov/compound/17100) |
| IMPHY012165 | Sabinene | C=C1CCC2(C1C2)C(C)C | [CID:18818](https://pubchem.ncbi.nlm.nih.gov/compound/18818) |
| IMPHY012168 | (1S,2S,6S,7R,8R)-1,3-dimethyl-8-propan-2-yltricyclo[4.4.0.02,7]dec-3-ene | CC([C@H]1CC[C@]2([C@@H]3[C@@H]1[C@H]2C(=CC3)C)C)C | [CID:101607926](https://pubchem.ncbi.nlm.nih.gov/compound/101607926) |
| IMPHY012255 | (+)-trans-Piperitenol | CC1=C[C@@H]([C@H](CC1)C(C)C)O | [CID:85568](https://pubchem.ncbi.nlm.nih.gov/compound/85568) |
| IMPHY012910 | trans-Calamenene | CC([C@H]1CC[C@@H](c2c1cc(C)cc2)C)C | [CID:6429022](https://pubchem.ncbi.nlm.nih.gov/compound/6429022) |
| IMPHY013080 | alpha-Calacorene | CC([C@@H]1CC=C(c2c1cc(C)cc2)C)C | [CID:12302243](https://pubchem.ncbi.nlm.nih.gov/compound/12302243) |
| IMPHY013093 | delta-Elemene | C=C[C@@]1(C)CCC(=C[C@@H]1C(=C)C)C(C)C | [CID:12309449](https://pubchem.ncbi.nlm.nih.gov/compound/12309449) |
| IMPHY013836 | Fenchone | O=C1C2(C)CCC(C1(C)C)C2 | [CID:14525](cid:14525) |
| IMPHY014690 | (-)-Globulol | C[C@@H]1CC[C@@H]2[C@@H]1[C@H]1[C@H](C1(C)C)CC[C@@]2(C)O | [CID:12304985](https://pubchem.ncbi.nlm.nih.gov/compound/12304985) |
| IMPHY014806 | Caswell No. 264AB | CC([C@@H]1CC[C@H]([C@]23[C@H]1[C@H]2C(=CC3)C)C)C | [CID:442359](https://pubchem.ncbi.nlm.nih.gov/compound/442359) |
| IMPHY014811 | alpha-Phellandrene | CC1=CCC(C=C1)C(C)C | [CID:7460](https://pubchem.ncbi.nlm.nih.gov/compound/7460) |
| IMPHY014831 | beta-Caryophyllene | C/C/1=CCCC(=C)[C@@H]2[C@@H](CC1)C(C2)(C)C | [CID:5281515](https://pubchem.ncbi.nlm.nih.gov/compound/5281515) |
| IMPHY014835 | (E)-beta-ocimene | C=C/C(=C/CC=C(C)C)/C | [CID:5281553](https://pubchem.ncbi.nlm.nih.gov/compound/5281553) |
| IMPHY014836 | beta-Sitosterol | CC[C@@H](C(C)C)CC[C@H]([C@H]1CC[C@@H]2[C@]1(C)CC[C@H]1[C@H]2CC=C2[C@]1(C)CC[C@@H](C2)O)C | [CID:222284](https://pubchem.ncbi.nlm.nih.gov/compound/222284) |
| IMPHY014847 | Bornyl acetate | CC(=O)OC1CC2C(C1(C)CC2)(C)C | [CID:6448](https://pubchem.ncbi.nlm.nih.gov/compound/6448) |
| IMPHY014852 | Camphene | C=C1C2CCC(C1(C)C)C2 | [CID:6616](https://pubchem.ncbi.nlm.nih.gov/compound/6616) |
| IMPHY014874 | cis-Sabinene hydrate | C[C@@H]1CC[C@@]2(C1C2)C(C)C | [CID:101629835](https://pubchem.ncbi.nlm.nih.gov/compound/101629835) |
| IMPHY014988 | Limonene | CC1=CCC(CC1)C(=C)C | [CID:22311](cid:22311) |
| IMPHY014989 | trans-Linalool oxide | C=C[C@]1(C)CC[C@H](O1)C(O)(C)C | [CID:6432254](https://pubchem.ncbi.nlm.nih.gov/compound/6432254) |
| IMPHY015123 | alpha-Copaene | CC([C@@H]1CC[C@]2([C@@H]3[C@H]1C2C(=CC3)C)C)C | [CID:70678558](https://pubchem.ncbi.nlm.nih.gov/compound/70678558) |
| IMPHY002333 | N-isobutyl-2e,4e,8z-eicosatrienamide | CCCCCCCCCCC/C=CCC/C=C/C=C/C(=O)NCC(C)C | 0 |
| IMPHY003955 | (R)-4-Isopropylcyclohex-2-enone | CC([C@H]1CCC(=O)C=C1)C | [CID:642520](https://pubchem.ncbi.nlm.nih.gov/compound/642520) |
| IMPHY004192 | Piperine | O=C(N1CCCCC1)/C=C/C=C/c1ccc2c(c1)OCO2 | [CID:638024](https://pubchem.ncbi.nlm.nih.gov/compound/638024) |
| IMPHY004217 | Chavicine | O=C(N1CCCCC1)/C=CC=C/c1ccc2c(c1)OCO2 | [CID:1548912](https://pubchem.ncbi.nlm.nih.gov/compound/1548912) |
| IMPHY006362 | Ascorbic acid | OC[C@@H]([C@H]1OC(=O)C(=C1O)O)O | [CID:54670067](https://pubchem.ncbi.nlm.nih.gov/compound/54670067) |
| IMPHY007055 | Piperonal | O=Cc1ccc2c(c1)OCO2 | [CID:8438](https://pubchem.ncbi.nlm.nih.gov/compound/8438) |
| IMPHY007188 | Piperidine | C1CCCNC1 | [CID:8082](https://pubchem.ncbi.nlm.nih.gov/compound/8082) |
| IMPHY007269 | Trichostachine | O=C(N1CCCC1)/C=C/C=C/c1ccc2c(c1)OCO2 | [CID:636537](https://pubchem.ncbi.nlm.nih.gov/compound/636537) |
| IMPHY011593 | Pellitorine | CCCCC/C=C/C=C/C(=O)NCC(C)C | [CID:5318516](https://pubchem.ncbi.nlm.nih.gov/compound/5318516) |
| IMPHY011609 | alpha-Carotene | C/C(=CC=CC=C(C=CC=C(C=CC1=C(C)CCCC1(C)C)/C)/C)/C=C/C=C(/C=C/[C@H]1C(=CCCC1(C)C)C)C | [CID:6419725](https://pubchem.ncbi.nlm.nih.gov/compound/6419725) |
| IMPHY011879 | Moupinamide | COc1cc(/C=C/C(=O)NCCc2ccc(cc2)O)ccc1O | [CID:5280537](https://pubchem.ncbi.nlm.nih.gov/compound/5280537) |
| IMPHY012104 | Citronellol | OCCC(CCC=C(C)C)C | [CID:8842](https://pubchem.ncbi.nlm.nih.gov/compound/8842) |
| IMPHY014852 | Camphene | C=C1C2CCC(C1(C)C)C2 | [CID:6616](https://pubchem.ncbi.nlm.nih.gov/compound/6616) |
| IMPHY000027 | (E)-Piperolein A | O=C(N1CCCCC1)CCCC/C=C/c1ccc2c(c1)OCO2 | [CID:11141599](https://pubchem.ncbi.nlm.nih.gov/compound/11141599) |
| IMPHY000056 | 1-[(2E,4E)-2,4-dodecadienoyl]pyrrolidine | CCCCCCC/C=C/C=C/C(=O)N1CCCC1 | [CID:10999431](https://pubchem.ncbi.nlm.nih.gov/compound/10999431) |
| IMPHY000099 | Myrtenol | OCC1=CCC2CC1C2(C)C | [CID:10582](https://pubchem.ncbi.nlm.nih.gov/compound/10582) |
| IMPHY000289 | Retrofractamide A | CC(CNC(=O)/C=C/C=C/CC/C=C/c1ccc2c(c1)OCO2)C | [CID:11012859](https://pubchem.ncbi.nlm.nih.gov/compound/11012859) |
| IMPHY000399 | beta-Bisabolene | CC(=CCCC(=C)[C@H]1CCC(=CC1)C)C | [CID:10104370](https://pubchem.ncbi.nlm.nih.gov/compound/10104370) |
| IMPHY000545 | O-Cymene | CC(c1ccccc1C)C | [CID:10703](https://pubchem.ncbi.nlm.nih.gov/compound/10703) |
| IMPHY001316 | (2E,4E,6E)-7-(2H-1,3-benzodioxol-5-yl)-1-(piperidin-1-yl)hepta-2,4,6-trien-1-one | O=C(N1CCCCC1)/C=C/C=C/C=C/c1ccc2c(c1)OCO2 | [CID:10244953](https://pubchem.ncbi.nlm.nih.gov/compound/10244953) |
| IMPHY001565 | Capsaicin | COc1cc(CNC(=O)CCCC/C=C/C(C)C)ccc1O | [CID:1548943](https://pubchem.ncbi.nlm.nih.gov/compound/1548943) |
| IMPHY001912 | Cubebin | O[C@H]1OC[C@@H]([C@H]1Cc1ccc2c(c1)OCO2)Cc1ccc2c(c1)OCO2 | [CID:117443](https://pubchem.ncbi.nlm.nih.gov/compound/117443) |
| IMPHY002872 | Piperolactam D | COc1c(OC)c2C(=O)Nc3c2c(c1O)c1ccccc1c3 | [CID:14039008](https://pubchem.ncbi.nlm.nih.gov/compound/14039008) |
| IMPHY002913 | Sesquisabinene | CC(=CCCC(C12CCC(=C)C2C1)C)C | [CID:25202482](https://pubchem.ncbi.nlm.nih.gov/compound/25202482) |
| IMPHY002950 | 2-Ethylpyrazine | CCc1cnccn1 | [CID:26331](https://pubchem.ncbi.nlm.nih.gov/compound/26331) |
| IMPHY002958 | 2,3,5-Trimethylpyrazine | Cc1cnc(c(n1)C)C | [CID:26808](https://pubchem.ncbi.nlm.nih.gov/compound/26808) |
| IMPHY002969 | Retrofractamide C | CC(CNC(=O)/C=C/CCCC/C=C/c1ccc2c(c1)OCO2)C | [CID:25255091](https://pubchem.ncbi.nlm.nih.gov/compound/25255091) |
| IMPHY003392 | Butylated hydroxytoluene | Cc1cc(c(c(c1)C(C)(C)C)O)C(C)(C)C | [CID:31404](https://pubchem.ncbi.nlm.nih.gov/compound/31404) |
| IMPHY003484 | 2,5-Dimethylpyrazine | Cc1ncc(nc1)C | [CID:31252](https://pubchem.ncbi.nlm.nih.gov/compound/31252) |
| IMPHY003485 | Myrcene | C=CC(=C)CCC=C(C)C | [CID:31253](https://pubchem.ncbi.nlm.nih.gov/compound/31253) |
| IMPHY003536 | Eugenol | C=CCc1ccc(c(c1)OC)O | [CID:3314](https://pubchem.ncbi.nlm.nih.gov/compound/3314) |
| IMPHY003616 | Bicyclogermacrene | C/C/1=CCC/C(=C/[C@H]2[C@@H](CC1)C2(C)C)/C | [CID:13894537](https://pubchem.ncbi.nlm.nih.gov/compound/13894537) |
| IMPHY003982 | gamma-Terpinene | CC1=CCC(=CC1)C(C)C | [CID:7461](https://pubchem.ncbi.nlm.nih.gov/compound/7461) |
| IMPHY004022 | Tricyclo(6.3.1.02,5)dodecan-1-ol, 4,4,8-trimethyl-, (1R,2S,5R,8S)- | C[C@]12CCC[C@](C2)(O)[C@@H]2[C@@H](CC1)C(C2)(C)C | [CID:11746218](https://pubchem.ncbi.nlm.nih.gov/compound/11746218) |
| IMPHY004079 | Catechol | Oc1ccccc1O | [CID:289](https://pubchem.ncbi.nlm.nih.gov/compound/289) |
| IMPHY004192 | Piperine | O=C(N1CCCCC1)/C=C/C=C/c1ccc2c(c1)OCO2 | [CID:638024](https://pubchem.ncbi.nlm.nih.gov/compound/638024) |
| IMPHY004531 | p-Mentha-1,8-dien-4-ol | CC1=CCC(CC1)(O)C(=C)C | [CID:527428](https://pubchem.ncbi.nlm.nih.gov/compound/527428) |
| IMPHY004549 | Safrole | C=CCc1ccc2c(c1)OCO2 | [CID:5144](https://pubchem.ncbi.nlm.nih.gov/compound/5144) |
| IMPHY005670 | 2,3-Dimethylpyrazine | Cc1nccnc1C | [CID:22201](https://pubchem.ncbi.nlm.nih.gov/compound/22201) |
| IMPHY005720 | Piperolein B | O=C(N1CCCCC1)CCCCCC/C=C/c1ccc2c(c1)OCO2 | [CID:21580213](https://pubchem.ncbi.nlm.nih.gov/compound/21580213) |
| IMPHY005944 | Pipercide | CC(CNC(=O)/C=C/C=C/CCCC/C=C/c1ccc2c(c1)OCO2)C | [CID:5372162](https://pubchem.ncbi.nlm.nih.gov/compound/5372162) |
| IMPHY006145 | p-Cymene | Cc1ccc(cc1)C(C)C | [CID:7463](https://pubchem.ncbi.nlm.nih.gov/compound/7463) |
| IMPHY006184 | Dehydropipernonaline | O=C(N1CCCCC1)/C=C/C=C/CC/C=C/c1ccc2c(c1)OCO2 | [CID:6439947](https://pubchem.ncbi.nlm.nih.gov/compound/6439947) |
| IMPHY006207 | Wisanine | COc1cc2OCOc2cc1/C=C/C=C/C(=O)N1CCCCC1 | [CID:6441085](https://pubchem.ncbi.nlm.nih.gov/compound/6441085) |
| IMPHY006215 | (2E,4E)-1-(Pyrrolidin-1-yl)deca-2,4-dien-1-one | CCCCC/C=C/C=C/C(=O)N1CCCC1 | [CID:6440616](https://pubchem.ncbi.nlm.nih.gov/compound/6440616) |
| IMPHY006266 | Guineensine | CC(CNC(=O)/C=C/C=C/CCCCCC/C=C/c1ccc2c(c1)OCO2)C | [CID:6442405](https://pubchem.ncbi.nlm.nih.gov/compound/6442405) |
| IMPHY006292 | Kakuol | CCC(=O)c1cc2OCOc2cc1O | [CID:596894](https://pubchem.ncbi.nlm.nih.gov/compound/596894) |
| IMPHY006362 | Ascorbic acid | OC[C@@H]([C@H]1OC(=O)C(=C1O)O)O | [CID:54670067](https://pubchem.ncbi.nlm.nih.gov/compound/54670067) |
| IMPHY006515 | Hedycaryol | C/C/1=C/CC/C(=CC[C@H](CC1)C(O)(C)C)/C | [CID:6432240](https://pubchem.ncbi.nlm.nih.gov/compound/6432240) |
| IMPHY006932 | Cuparene | Cc1ccc(cc1)[C@]1(C)CCCC1(C)C | [CID:86895](https://pubchem.ncbi.nlm.nih.gov/compound/86895) |
| IMPHY006953 | 2,6-Dimethylpyrazine | Cc1cncc(n1)C | [CID:7938](https://pubchem.ncbi.nlm.nih.gov/compound/7938) |
| IMPHY006978 | 2-Methylpyrazine | Cc1cnccn1 | [CID:7976](https://pubchem.ncbi.nlm.nih.gov/compound/7976) |
| IMPHY007055 | Piperonal | O=Cc1ccc2c(c1)OCO2 | [CID:8438](https://pubchem.ncbi.nlm.nih.gov/compound/8438) |
| IMPHY007188 | Piperidine | C1CCCNC1 | [CID:8082](https://pubchem.ncbi.nlm.nih.gov/compound/8082) |
| IMPHY007202 | Nonane | CCCCCCCCC | [CID:8141](https://pubchem.ncbi.nlm.nih.gov/compound/8141) |
| IMPHY007269 | Trichostachine | O=C(N1CCCC1)/C=C/C=C/c1ccc2c(c1)OCO2 | [CID:636537](https://pubchem.ncbi.nlm.nih.gov/compound/636537) |
| IMPHY007371 | Cepharadione A | O=C1N(C)c2cc3ccccc3c3c2c(C1=O)cc1c3OCO1 | [CID:94577](https://pubchem.ncbi.nlm.nih.gov/compound/94577) |
| IMPHY007442 | 5-Methyl-2-propan-2-ylcyclohexa-2,4-dien-1-ol | CC1=CC=C(C(C1)O)C(C)C | [CID:91752234](https://pubchem.ncbi.nlm.nih.gov/compound/91752234) |
| IMPHY007445 | (Z)-beta-Ocimenol | C=C/C(=CC(C=C(C)C)O)/C | [CID:91753567](https://pubchem.ncbi.nlm.nih.gov/compound/91753567) |
| IMPHY007559 | Pipernonaline | O=C(N1CCCCC1)/C=C/CCCC/C=C/c1ccc2c(c1)OCO2 | [CID:9974595](https://pubchem.ncbi.nlm.nih.gov/compound/9974595) |
| IMPHY007574 | Curcumin | COc1cc(/C=C/C(=O)CC(=O)/C=C/c2ccc(c(c2)OC)O)ccc1O | [CID:969516](https://pubchem.ncbi.nlm.nih.gov/compound/969516) |
| IMPHY007840 | Spathulenol | C=C1CC[C@@H]2[C@H]([C@H]3[C@H]1CC[C@]3(C)O)C2(C)C | [CID:92231](https://pubchem.ncbi.nlm.nih.gov/compound/92231) |
| IMPHY008686 | 2-Ethyl-5-methylpyrazine | CCc1cnc(cn1)C | [CID:25915](https://pubchem.ncbi.nlm.nih.gov/compound/25915) |
| IMPHY008936 | alpha-Guaiene | CC(=C)[C@@H]1CC[C@@H](C2=C(C1)[C@@H](C)CC2)C | [CID:5317844](https://pubchem.ncbi.nlm.nih.gov/compound/5317844) |
| IMPHY008946 | delta-Guaiene | CC(=C)[C@@H]1CCC(=C2[C@@H](C1)[C@@H](C)CC2)C | [CID:94275](https://pubchem.ncbi.nlm.nih.gov/compound/94275) |
| IMPHY009604 | Dihydroferuperine | COC1=CC(=CCC1O)/C=CC=CC(=O)N1CCCCC1 | [CID:131752910](https://pubchem.ncbi.nlm.nih.gov/compound/131752910) |
| IMPHY009605 | Feruperine | COc1cc(/C=CC=CC(=O)N2CCCCC2)ccc1O | [CID:131752909](https://pubchem.ncbi.nlm.nih.gov/compound/131752909) |
| IMPHY010072 | Eucalyptol | CC12CCC(CC1)C(O2)(C)C | [CID:2758](https://pubchem.ncbi.nlm.nih.gov/compound/2758) |
| IMPHY010080 | beta-Elemene | C=C[C@]1(C)CC[C@H](C[C@H]1C(=C)C)C(=C)C | [CID:6918391](https://pubchem.ncbi.nlm.nih.gov/compound/6918391) |
| IMPHY010205 | Pipecolic acid | OC(=O)C1CCCCN1 | [CID:849](https://pubchem.ncbi.nlm.nih.gov/compound/849) |
| IMPHY010244 | (1S,5S,8S)-4,4,8-Trimethyltricyclo[6.3.1.01,5]dodec-2-ene | C[C@]12CCC[C@@]3(C2)[C@@H](CC1)C(C=C3)(C)C | [CID:10102](https://pubchem.ncbi.nlm.nih.gov/compound/10102) |
| IMPHY011392 | 3-Carene | CC1=CCC2C(C1)C2(C)C | [CID:26049](https://pubchem.ncbi.nlm.nih.gov/compound/26049) |
| IMPHY011396 | 4-Carvomenthenol | CC1=CCC(CC1)(O)C(C)C | [CID:11230](https://pubchem.ncbi.nlm.nih.gov/compound/11230) |
| IMPHY011464 | Isobutyramide | CC(C(=O)N)C | [CID:68424](https://pubchem.ncbi.nlm.nih.gov/compound/68424) |
| IMPHY011519 | alpha-Terpinyl acetate | CC(=O)OC(C1CCC(=CC1)C)(C)C | [CID:111037](https://pubchem.ncbi.nlm.nih.gov/compound/111037) |
| IMPHY011542 | beta-Eudesmol | C=C1CCC[C@]2([C@H]1C[C@@H](CC2)C(O)(C)C)C | [CID:91457](https://pubchem.ncbi.nlm.nih.gov/compound/91457) |
| IMPHY011552 | (1R)-2-methyl-5-propan-2-ylbicyclo[3.1.0]hex-2-ene | CC1=CCC2([C@@H]1C2)C(C)C | [CID:6451618](https://pubchem.ncbi.nlm.nih.gov/compound/6451618) |
| IMPHY011581 | alpha-Selinene | CC1=CCC[C@]2([C@H]1C[C@@H](CC2)C(=C)C)C | [CID:10856614](https://pubchem.ncbi.nlm.nih.gov/compound/10856614) |
| IMPHY011586 | (S,1Z,6Z)-8-Isopropyl-1-methyl-5-methylenecyclodeca-1,6-diene | C/C/1=C/CCC(=C)/C=C[C@@H](CC1)C(C)C | [CID:91723653](https://pubchem.ncbi.nlm.nih.gov/compound/91723653) |
| IMPHY011590 | d-Borneol | O[C@@H]1C[C@H]2C([C@@]1(C)CC2)(C)C | [CID:61060](https://pubchem.ncbi.nlm.nih.gov/compound/61060) |
| IMPHY011593 | Pellitorine | CCCCC/C=C/C=C/C(=O)NCC(C)C | [CID:5318516](https://pubchem.ncbi.nlm.nih.gov/compound/5318516) |
| IMPHY011599 | Terpinolene | CC1=CCC(=C(C)C)CC1 | [CID:11463](cid:11463) |
| IMPHY011609 | alpha-Carotene | C/C(=CC=CC=C(C=CC=C(C=CC1=C(C)CCCC1(C)C)/C)/C)/C=C/C=C(/C=C/[C@H]1C(=CCCC1(C)C)C)C | [CID:6419725](https://pubchem.ncbi.nlm.nih.gov/compound/6419725) |
| IMPHY011643 | alpha-Terpinene | CC1=CC=C(CC1)C(C)C | [CID:7462](https://pubchem.ncbi.nlm.nih.gov/compound/7462) |
| IMPHY011647 | Geranyl acetate | C/C(=CCOC(=O)C)/CCC=C(C)C | [CID:1549026](https://pubchem.ncbi.nlm.nih.gov/compound/1549026) |
| IMPHY011648 | Neryl acetate | C/C(=C/COC(=O)C)/CCC=C(C)C | [CID:1549025](https://pubchem.ncbi.nlm.nih.gov/compound/1549025) |
| IMPHY011658 | beta-Farnesene | C=CC(=C)CC/C=C(/CCC=C(C)C)C | [CID:5281517](https://pubchem.ncbi.nlm.nih.gov/compound/5281517) |
| IMPHY011741 | Tannic acid | O=C(c1cc(O)c(c(c1)OC(=O)c1cc(O)c(c(c1)O)O)O)O[C@@H]1[C@@H](COC(=O)c2cc(O)c(c(c2)OC(=O)c2cc(O)c(c(c2)O)O)O)O[C@H]([C@@H]([C@H]1OC(=O)c1cc(O)c(c(c1)OC(=O)c1cc(O)c(c(c1)O)O)O)OC(=O)c1cc(O)c(c(c1)OC(=O)c1cc(O)c(c(c1)O)O)O)OC(=O)c1cc(O)c(c(c1)OC(=O)c1cc(O)c(c(c1)O)O)O | [CID:16129778](https://pubchem.ncbi.nlm.nih.gov/compound/16129778) |
| IMPHY011761 | Humulene | C/C/1=CCC(C)(C)/C=C/C/C(=C/CC1)/C | [CID:5281520](https://pubchem.ncbi.nlm.nih.gov/compound/5281520) |
| IMPHY011792 | gamma-Muurolene | CC1=C[C@@H]2[C@H](CC1)C(=C)CC[C@H]2C(C)C | [CID:12313020](https://pubchem.ncbi.nlm.nih.gov/compound/12313020) |
| IMPHY011817 | alpha-Farnesene | C=C/C(=C/C/C=C(/CCC=C(C)C)C)/C | [CID:5281516](https://pubchem.ncbi.nlm.nih.gov/compound/5281516) |
| IMPHY011879 | Moupinamide | COc1cc(/C=C/C(=O)NCCc2ccc(cc2)O)ccc1O | [CID:5280537](https://pubchem.ncbi.nlm.nih.gov/compound/5280537) |
| IMPHY011890 | Elemol | C=C[C@]1(C)CC[C@H](C[C@H]1C(=C)C)C(O)(C)C | [CID:92138](https://pubchem.ncbi.nlm.nih.gov/compound/92138) |
| IMPHY011957 | (+)-delta-Cadinene | CC1=C[C@@H]2C(=C(C)CC[C@H]2C(C)C)CC1 | [CID:441005](https://pubchem.ncbi.nlm.nih.gov/compound/441005) |
| IMPHY011988 | (-)-trans-Carveol | CC(=C)[C@@H]1CC=C([C@H](C1)O)C | [CID:94221](https://pubchem.ncbi.nlm.nih.gov/compound/94221) |
| IMPHY012012 | (E,E)-2,4-Decadienoic isobutylamide | CCCCC/C=CC=CC(=O)NCC(C)C | [CID:11368078](https://pubchem.ncbi.nlm.nih.gov/compound/11368078) |
| IMPHY012058 | Linalool | C=CC(CCC=C(C)C)(O)C | [CID:6549](https://pubchem.ncbi.nlm.nih.gov/compound/6549) |
| IMPHY012061 | alpha-Pinene | CC1=CCC2CC1C2(C)C | [CID:6654](https://pubchem.ncbi.nlm.nih.gov/compound/6654) |
| IMPHY012086 | Citronellal | O=CCC(CCC=C(C)C)C | [CID:7794](https://pubchem.ncbi.nlm.nih.gov/compound/7794) |
| IMPHY012104 | Citronellol | OCCC(CCC=C(C)C)C | [CID:8842](https://pubchem.ncbi.nlm.nih.gov/compound/8842) |
| IMPHY012130 | Dihydrocarveol | CC(=C)C1CCC(C(C1)O)C | [CID:12072](https://pubchem.ncbi.nlm.nih.gov/compound/12072) |
| IMPHY012147 | beta-Pinene | C=C1CCC2CC1C2(C)C | [CID:14896](https://pubchem.ncbi.nlm.nih.gov/compound/14896) |
| IMPHY012160 | alpha-Terpineol | CC1=CCC(CC1)C(O)(C)C | [CID:17100](cid:17100) |
| IMPHY012165 | Sabinene | C=C1CCC2(C1C2)C(C)C | [CID:18818](https://pubchem.ncbi.nlm.nih.gov/compound/18818) |
| IMPHY012175 | D-Limonene | CC1=CC[C@@H](CC1)C(=C)C | [CID:440917](https://pubchem.ncbi.nlm.nih.gov/compound/440917) |
| IMPHY012279 | alpha-Curcumene | CC(=CCCC(c1ccc(cc1)C)C)C | [CID:92139](https://pubchem.ncbi.nlm.nih.gov/compound/92139) |
| IMPHY012667 | Caryophyllene oxide | C=C1CC[C@H]2O[C@@]2(CC[C@@H]2[C@@H]1CC2(C)C)C | [CID:1742210](https://pubchem.ncbi.nlm.nih.gov/compound/1742210) |
| IMPHY014708 | beta-Selinene | C=C1CCC[C@]2([C@H]1C[C@@H](CC2)C(=C)C)C | [CID:442393](https://pubchem.ncbi.nlm.nih.gov/compound/442393) |
| IMPHY014801 | Zizanene | CC1=C[C@@H]2[C@H](CC1)C(=CC[C@@H]2C(C)C)C | [CID:12306046](https://pubchem.ncbi.nlm.nih.gov/compound/12306046) |
| IMPHY014806 | Caswell No. 264AB | CC([C@@H]1CC[C@H]([C@]23[C@H]1[C@H]2C(=CC3)C)C)C | [CID:442359](https://pubchem.ncbi.nlm.nih.gov/compound/442359) |
| IMPHY014811 | alpha-Phellandrene | CC1=CCC(C=C1)C(C)C | [CID:7460](https://pubchem.ncbi.nlm.nih.gov/compound/7460) |
| IMPHY014831 | beta-Caryophyllene | C/C/1=CCCC(=C)[C@@H]2[C@@H](CC1)C(C2)(C)C | [CID:5281515](https://pubchem.ncbi.nlm.nih.gov/compound/5281515) |
| IMPHY014835 | (E)-beta-ocimene | C=C/C(=C/CC=C(C)C)/C | [CID:5281553](https://pubchem.ncbi.nlm.nih.gov/compound/5281553) |
| IMPHY014852 | Camphene | C=C1C2CCC(C1(C)C)C2 | [CID:6616](https://pubchem.ncbi.nlm.nih.gov/compound/6616) |
| IMPHY014857 | Cedrol | C[C@@H]1CC[C@@H]2[C@@]31CC[C@@]([C@H](C3)C2(C)C)(C)O | [CID:65575](https://pubchem.ncbi.nlm.nih.gov/compound/65575) |
| IMPHY014871 | cis-Nerolidol | C=CC(CC/C=C(CCC=C(C)C)/C)(O)C | [CID:5320128](cid:5320128) |
| IMPHY014907 | 6-Epi-beta-bisabolol | CC(=CCC[C@@H]([C@@]1(O)CCC(=CC1)C)C)C | [CID:12300148](https://pubchem.ncbi.nlm.nih.gov/compound/12300148) |
| IMPHY014988 | Limonene | CC1=CCC(CC1)C(=C)C | [CID:22311](https://pubchem.ncbi.nlm.nih.gov/compound/22311) |
| IMPHY014989 | trans-Linalool oxide | C=C[C@]1(C)CC[C@H](O1)C(O)(C)C | [CID:6432254](https://pubchem.ncbi.nlm.nih.gov/compound/6432254) |
| IMPHY015042 | Piperitone | CC1=CC(=O)C(CC1)C(C)C | [CID:6987](https://pubchem.ncbi.nlm.nih.gov/compound/6987) |
| IMPHY015095 | 2-Cyclohexen-1-ol, 1-methyl-4-(1-methylethyl)-, trans- | CC([C@@H]1CC[C@@](C=C1)(C)O)C | [CID:122484](https://pubchem.ncbi.nlm.nih.gov/compound/122484) |
| IMPHY015123 | alpha-Copaene | CC([C@@H]1CC[C@]2([C@@H]3[C@H]1C2C(=CC3)C)C)C | [CID:70678558](https://pubchem.ncbi.nlm.nih.gov/compound/70678558) |
| IMPHY015919 | 2-Ethyl-6-methylpyrazine | CCc1cncc(n1)C | [CID:26332](https://pubchem.ncbi.nlm.nih.gov/compound/26332) |
| IMPHY016027 | trans-Sabinene hydrate | CC([C@@]12CC[C@](C2C1)(C)O)C | [CID:12315151](https://pubchem.ncbi.nlm.nih.gov/compound/12315151) |
| IMPHY016054 | trans-alpha-Bergamotene | CC(=CCC[C@]1(C)[C@H]2CC=C([C@@H]1C2)C)C | [CID:6429302](https://pubchem.ncbi.nlm.nih.gov/compound/6429302) |
| IMPHY016453 | 5-Ethyl-2-methylpyridine | CCc1ccc(nc1)C | [CID:7728](https://pubchem.ncbi.nlm.nih.gov/compound/7728) |
| IMPHY017003 | 1-Acetylpiperidine | CC(=O)N1CCCCC1 | [CID:12058](https://pubchem.ncbi.nlm.nih.gov/compound/12058) |
| IMPHY017167 | N-Formylpiperidine | O=CN1CCCCC1 | [CID:17429](https://pubchem.ncbi.nlm.nih.gov/compound/17429) |
| IMPHY017242 | 3-Ethyl-2,5-dimethylpyrazine | CCc1nc(C)cnc1C | CID: 25916 |
| IMPHY017245 | 2-Ethyl-3,5-dimethylpyrazine | CCc1ncc(nc1C)C | CID: 26334 |
| IMPHY017248 | 2-Ethyl-3-methylpyrazine | CCc1nccnc1C | CID: 27457 |
| IMPHY017258 | 3,5-Diethyl-2-methylpyrazine | CCc1cnc(c(n1)CC)C | CID: 28906 |
| IMPHY017687 | 2-Isopropylpyridine | CC(c1ccccn1)C | CID: 69523 |
| IMPHY017708 | 2-Heptylpyridine | CCCCCCCc1ccccn1 | CID: 72919 |
| IMPHY017728 | 2-Ethylpyridine | CCc1ccccn1 | CID: 7523 |
| IMPHY017744 | 2-Butylpyridine | CCCCc1ccccn1 | CID: 78750 |
| IMPHY017751 | 2,6-Dimethylpyridine | Cc1cccc(n1)C | CID: 7937 |

| ***13.Plant name : Rosa centifolia*** | | | |
| --- | --- | --- | --- |
| **IMPPAT Phytochemical identifier:** | **Phytochemical name:** | **SMILES:** | **CID** |
| IMPHY000308 | Hexadecane | CCCCCCCCCCCCCCCC | 11006 |
| IMPHY001846 | Dimethyl disulfide | CSSC | 12232 |
| IMPHY001854 | 3-Methylbutanal | O=CCC(C)C | 11552 |
| IMPHY001896 | Heptacosane | CCCCCCCCCCCCCCCCCCCCCCCCCCC | 11636 |
| IMPHY001915 | Octadecane | CCCCCCCCCCCCCCCCCC | 11635 |
| IMPHY002434 | beta-Maaliene | CC1=C2[C@H]3[C@H](C3(C)C)CC[C@@]2(CCC1)C | 101596917 |
| IMPHY002875 | Undecane | CCCCCCCCCCC | 14257 |
| IMPHY002915 | Benzyl Alcohol | OCc1ccccc1 | 244 |
| IMPHY002949 | 1-Butanol | CCCCO | 263 |
| IMPHY003050 | Methyl salicylate | COC(=O)c1ccccc1O | 4133 |
| IMPHY003482 | 4-Methoxybenzaldehyde | COc1ccc(cc1)C=O | 31244 |
| IMPHY003485 | Myrcene | C=CC(=C)CCC=C(C)C | 31253 |
| IMPHY003519 | Isoamyl acetate | CC(CCOC(=O)C)C | 31276 |
| IMPHY003536 | Eugenol | C=CCc1ccc(c(c1)OC)O | 3314 |
| IMPHY004751 | 3-(Methylthio)propionaldehyde | CSCCC=O | 18635 |
| IMPHY006037 | 2-Methylbutyraldehyde | CC(C=O)CC | 7284 |
| IMPHY006138 | Anisole | COc1ccccc1 | 7519 |
| IMPHY006145 | p-Cymene | Cc1ccc(cc1)C(C)C | 7463 |
| IMPHY006177 | Methyl geranate | COC(=O)/C=C(/CCC=C(C)C)C | 5365910 |
| IMPHY006279 | 2-Phenylethanol | OCCc1ccccc1 | 6054 |
| IMPHY006347 | Hexanal | CCCCCC=O | 6184 |
| IMPHY006352 | Nerol oxide | CC(=CC1OCC=C(C1)C)C | 61275 |
| IMPHY006404 | Damascenone | C/C=C/C(=O)C1=C(C)C=CCC1(C)C | 5366074 |
| IMPHY006485 | beta-Ionone | CC(=O)/C=C/C1=C(C)CCCC1(C)C | 638014 |
| IMPHY006574 | Ethyl benzoate | CCOC(=O)c1ccccc1 | 7165 |
| IMPHY006696 | Methyleugenol | C=CCc1ccc(c(c1)OC)OC | 7127 |
| IMPHY006700 | Methyl benzoate | COC(=O)c1ccccc1 | 7150 |
| IMPHY006934 | 2-Methyl-1-butanol | CCC(CO)C | 8723 |
| IMPHY006951 | Eicosane | CCCCCCCCCCCCCCCCCCCC | 8222 |
| IMPHY006989 | 2-Pentanone | CCCC(=O)C | 7895 |
| IMPHY007041 | Furfural | O=Cc1ccco1 | 7362 |
| IMPHY007083 | Methionine sulfoxide | CS(=O)CCC(C(=O)O)N | 847 |
| IMPHY007131 | Perillene | CC(=CCCc1cocc1)C | 68316 |
| IMPHY007171 | 1-Hexanol | CCCCCCO | 8103 |
| IMPHY007186 | Heptanal | CCCCCCC=O | 8130 |
| IMPHY007214 | Octyl acetate | CCCCCCCCOC(=O)C | 8164 |
| IMPHY007302 | Hexyl acetate | CCCCCCOC(=O)C | 8908 |
| IMPHY007331 | 6-Methyl-5-hepten-2-one | CC(=O)CCC=C(C)C | 9862 |
| IMPHY007421 | Citronellyl acetate | CC(CCC=C(C)C)CCOC(=O)C | 9017 |
| IMPHY008146 | 1-Pentanol | CCCCCO | 6276 |
| IMPHY008946 | delta-Guaiene | CC(=C)[C@@H]1CCC(=C2[C@@H](C1)[C@@H](C)CC2)C | 94275 |
| IMPHY008972 | Butyl acetate | CCCCOC(=O)C | 31272 |
| IMPHY008991 | Benzyl acetate | CC(=O)OCc1ccccc1 | 8785 |
| IMPHY009355 | Tetracosane | CCCCCCCCCCCCCCCCCCCCCCCC | 12592 |
| IMPHY009359 | Hexacosane | CCCCCCCCCCCCCCCCCCCCCCCCCC | 12407 |
| IMPHY009368 | Heptadecane | CCCCCCCCCCCCCCCCC | 12398 |
| IMPHY009369 | Nonadecane | CCCCCCCCCCCCCCCCCCC | 12401 |
| IMPHY009375 | Docosane | CCCCCCCCCCCCCCCCCCCCCC | 12405 |
| IMPHY009377 | Pentacosane | CCCCCCCCCCCCCCCCCCCCCCCCC | 12406 |
| IMPHY009382 | Heneicosane | CCCCCCCCCCCCCCCCCCCCC | 12403 |
| IMPHY009389 | Pentadecane | CCCCCCCCCCCCCCC | 12391 |
| IMPHY009490 | Tricosane | CCCCCCCCCCCCCCCCCCCCCCC | 12534 |
| IMPHY009596 | Pentyl acetate | CCCCCOC(=O)C | 12348 |
| IMPHY009879 | Hexyl benzoate | CCCCCCOC(=O)c1ccccc1 | 23235 |
| IMPHY009946 | Benzaldehyde | O=Cc1ccccc1 | 240 |
| IMPHY009955 | Cyclohexane | C1CCCCC1 | 8078 |
| IMPHY010072 | Eucalyptol | CC12CCC(CC1)C(O2)(C)C | 2758 |
| IMPHY010086 | 6-Methylhept-5-en-2-ol | CC(CCC=C(C)C)O | 20745 |
| IMPHY010097 | Benzyl benzoate | O=C(c1ccccc1)OCc1ccccc1 | 2345 |
| IMPHY010995 | Toluene | Cc1ccccc1 | 1140 |
| IMPHY011010 | 1-Nonadecene | CCCCCCCCCCCCCCCCCC=C | 29075 |
| IMPHY011396 | 4-Carvomenthenol | CC1=CCC(CC1)(O)C(C)C | 11230 |
| IMPHY011586 | (S,1Z,6Z)-8-Isopropyl-1-methyl-5-methylenecyclodeca-1,6-diene | C/C/1=C/CCC(=C)/C=C[C@@H](CC1)C(C)C | 91723653 |
| IMPHY011599 | Terpinolene | CC1=CCC(=C(C)C)CC1 | 11463 |
| IMPHY011632 | Farnesol | OC/C=C(/CC/C=C(/CCC=C(C)C)C)C | 445070 |
| IMPHY011647 | Geranyl acetate | C/C(=CCOC(=O)C)/CCC=C(C)C | 1549026 |
| IMPHY011648 | Neryl acetate | C/C(=C/COC(=O)C)/CCC=C(C)C | 1549025 |
| IMPHY011659 | alpha-Muurolene | CC1=C[C@@H]2[C@H](CC1)C(=CC[C@H]2C(C)C)C | 12306047 |
| IMPHY011761 | Humulene | C/C/1=CCC(C)(C)/C=C/C/C(=C/CC1)/C | 5281520 |
| IMPHY011763 | Anethole | C/C=C/c1ccc(cc1)OC | 637563 |
| IMPHY011789 | Citral | O=C/C=C(/CCC=C(C)C)C | 638011 |
| IMPHY011790 | Neral | O=C/C=C(CCC=C(C)C)/C | 643779 |
| IMPHY011804 | cis-3-Hexenyl acetate | CC/C=CCCOC(=O)C | 5363388 |
| IMPHY011884 | Pulegone | C[C@@H]1CCC(=C(C)C)C(=O)C1 | 442495 |
| IMPHY011938 | gamma-Eudesmol | CC1=C2C[C@@H](CC[C@]2(CCC1)C)C(O)(C)C | 6432005 |
| IMPHY011957 | (+)-delta-Cadinene | CC1=C[C@@H]2C(=C(C)CC[C@H]2C(C)C)CC1 | 441005 |
| IMPHY011965 | (+)-beta-Phellandrene | CC([C@@H]1CCC(=C)C=C1)C | 442484 |
| IMPHY012036 | Camphor | O=C1CC2C(C1(C)CC2)(C)C | 2537 |
| IMPHY012058 | Linalool | C=CC(CCC=C(C)C)(O)C | 6549 |
| IMPHY012061 | alpha-Pinene | CC1=CCC2CC1C2(C)C | 6654 |
| IMPHY012070 | o-Xylene | Cc1ccccc1C | 7237 |
| IMPHY012104 | Citronellol | OCCC(CCC=C(C)C)C | 8842 |
| IMPHY012147 | beta-Pinene | C=C1CCC2CC1C2(C)C | 14896 |
| IMPHY012160 | alpha-Terpineol | CC1=CCC(CC1)C(O)(C)C | 17100 |
| IMPHY012165 | Sabinene | C=C1CCC2(C1C2)C(C)C | 18818 |
| IMPHY012179 | (2S,4R)-4-methyl-2-(2-methylprop-1-en-1-yl)tetrahydro-2H-pyran | C[C@@H]1CCO[C@@H](C1)C=C(C)C | 1712087 |
| IMPHY012654 | Nerol | OC/C=C(CCC=C(C)C)/C | 643820 |
| IMPHY013836 | Fenchone | O=C1C2(C)CCC(C1(C)C)C2 | 14525 |
| IMPHY014817 | Aromadendrene | CC1CCC2C1C1C(C1(C)C)CCC2=C | 91354 |
| IMPHY014831 | beta-Caryophyllene | C/C/1=CCCC(=C)[C@@H]2[C@@H](CC1)C(C2) | 5281515 |
| IMPHY014835 | (E)-beta-ocimene | C=C/C(=C/CC=C(C)C)/C | 5281553 |
| IMPHY014923 | Geraniol | OC/C=C(/CCC=C(C)C)C | 637566 |
| IMPHY014988 | Limonene | CC1=CCC(CC1)C(=C)C | 22311 |
| IMPHY015003 | Menthol | CC1CCC(C(C1)O)C(C)C | 1254 |
| IMPHY015004 | Menthone | C[C@@H]1CC[C@H](C(=O)C1)C(C)C | 26447 |
| IMPHY015022 | Nerolidol | C=CC(CC/C=C(/CCC=C(C)C)C)(O)C | 5284507 |
| IMPHY015123 | alpha-Copaene | CC([C@@H]1CC[C@]2([C@@H]3[C@H]1C2C(=CC3)C)C)C | 70678558 |
| IMPHY015376 | 4-(Prop-1-en-2-yl)benzaldehyde | O=Cc1ccc(cc1)C(=C)C | 14597914 |
| IMPHY015751 | 3-Methylbutyl pentanoate | CCCCC(=O)OCCC(C)C | 74901 |
| IMPHY017272 | Chlorodibromomethane | ClC(Br)Br | 31296 |
| IMPHY017472 | Bromoform | BrC(Br)Br | 5558 |
| IMPHY007083 | Methionine sulfoxide | CS(=O)CCC(C(=O)O)N | 847 |
| IMPHY002915 | Benzyl Alcohol | OCc1ccccc1 | 244 |
| IMPHY003485 | Myrcene | C=CC(=C)CCC=C(C)C | 31253 |
| IMPHY003536 | Eugenol | C=CCc1ccc(c(c1)OC)O | 3314 |
| IMPHY004536 | Geranic acid | CC(=CCC/C(=C/C(=O)O)/C)C | 5275520 |
| IMPHY006279 | 2-Phenylethanol | OCCc1ccccc1 | 6054 |
| IMPHY007421 | Citronellyl acetate | CC(CCC=C(C)C)CCOC(=O)C | 9017 |
| IMPHY011647 | Geranyl acetate | C/C(=CCOC(=O)C)/CCC=C(C)C | 1549026 |
| IMPHY011789 | Citral | O=C/C=C(/CCC=C(C)C)C | 638011 |
| IMPHY012058 | Linalool | C=CC(CCC=C(C)C)(O)C | 6549 |
| IMPHY012061 | alpha-Pinene | CC1=CCC2CC1C2(C)C | 6654 |
| IMPHY012104 | Citronellol | OCCC(CCC=C(C)C)C | 8842 |
| IMPHY012147 | beta-Pinene | C=C1CCC2CC1C2(C)C | 14896 |
| IMPHY012524 | Cyanin | OC[C@H]1O[C@@H](Oc2cc(O)cc3c2cc(O[C@@H]2O[C@H](CO)[C@H]([C@@H]([C@H]2O)O)O)c([o+]3)c2ccc(c(c2)O)O)[C@@H]([C@H]([C@@H]1O)O)O | 441688 |
| IMPHY012654 | Nerol | OC/C=C(CCC=C(C)C)/C | 643820 |
| IMPHY014923 | Geraniol | OC/C=C(/CCC=C(C)C)C | 637566 |
| IMPHY015054 | Quercitrin | Oc1cc(O)c2c(c1)oc(c(c2=O)O[C@@H]1O[C@@H](C)[C@@H]([C@H]([C@H]1O)O)O)c1ccc(c(c1)O)O | 5280459 |
|  |  |  |  |
|  |  |  |  |
|  |  |  |  |
| ***14. Plant name : Citrus limon*** | | | |
| **IMPPAT Phytochemical identifier:** | **Phytochemical name:** | **SMILES:** | **CID** |
| IMPHY003485 | Myrcene | C=CC(=C)CCC=C(C)C | 31253 |
| IMPHY003710 | (-)-Isopulegol | C[C@@H]1CC[C@H]([C@@H](C1)O)C(=C)C | 170833 |
| IMPHY003982 | gamma-Terpinene | CC1=CCC(=CC1)C(C)C | 7461 |
| IMPHY006550 | : Thymol | Cc1ccc(c(c1)O)C(C)C | 6989 |
| IMPHY006970 | Decanal | CCCCCCCCCC=O | 8175 |
| IMPHY007421 | Citronellyl acetate | CC(CCC=C(C)C)CCOC(=O)C | 9017 |
| IMPHY011647 | Geranyl acetate | C/C(=CCOC(=O)C)/CCC=C(C)C | 1549026 |
| IMPHY011789 | Citral | O=C/C=C(/CCC=C(C)C)C | 638011 |
| IMPHY012058 | Linalool | C=CC(CCC=C(C)C)(O)C | 6549 |
| IMPHY012061 | alpha-Pinene | CC1=CCC2CC1C2(C)C | 6654 |
| IMPHY012147 | beta-Pinene | C=C1CCC2CC1C2(C)C | 14896 |
| IMPHY012175 | D-Limonene | CC1=CC[C@@H](CC1)C(=C)C | 440917 |
| IMPHY012665 | Levomenol | CC(=CCC[C@@]([C@H]1CCC(=CC1)C)(O)C)C | 442343 |
| IMPHY012712 | Phytol | OC/C=C(/CCC[C@@H](CCC[C@@H](CCCC(C)C)C)C)C | 5280435 |
| IMPHY012739 | (Z)-beta-Ocimene | C=C/C(=CCC=C(C)C)/C | 5320250 |
| IMPHY014831 | beta-Caryophyllene | C/C/1=CCCC(=C)[C@@H]2[C@@H](CC1)C(C2)(C)C | 5281515 |
| IMPHY014835 | (E)-beta-ocimene | C=C/C(=C/CC=C(C)C)/C | 5281553 |
| IMPHY014852 | Camphene | C=C1C2CCC(C1(C)C)C2 | 6616 |
| IMPHY015098 | trans-Verbenol | CC1=C[C@H](O)[C@@H]2C[C@H]1C2(C)C | 89664 |
| IMPHY015997 | (+)-alpha-Terpineol | CC1=CC[C@@H](CC1)C(O)(C)C | 442501 |
| IMPHY016940 | 3,7-Dimethyloct-7-enal | O=CCC(CCCC(=C)C)C | 101628 |
| IMPHY011395 | Scoparone | COc1cc2oc(=O)ccc2cc1OC | 8417 |
| IMPHY008724 | Isorhamnetin | COc1cc(ccc1O)c1oc2cc(O)cc(c2c(=O)c1O)O | 5281654 |
| IMPHY000005 | Thiamine | OCCc1sc[n+](c1C)Cc1cnc(nc1N)C | 1130 |
| IMPHY000171 | Ascaridole | CC(C12CCC(OO1)(C=C2)C)C | 10545 |
| IMPHY000399 | beta-Bisabolene | CC(=CCCC(=C)[C@H]1CCC(=CC1)C)C | 10104370 |
| IMPHY000602 | M-Cymene | Cc1cccc(c1)C(C)C | 10812 |
| IMPHY000619 | Perillyl alcohol | OCC1=CCC(CC1)C(=C)C | 10819 |
| IMPHY000795 | Octanal | CCCCCCCC=O | 454 |
| IMPHY000911 | Naringin | OC[C@H]1O[C@@H](Oc2cc(O)c3c(c2)O[C@@H](CC3=O)c2ccc(cc2)O)[C@@H]([C@H]([C@@H]1O)O)O[C@@H]1O[C@@H](C)[C@@H]([C@H]([C@H]1O)O)O | 442428 |
| IMPHY001246 | Carvacrol | CC(c1ccc(c(c1)O)C)C | 10364 |
| IMPHY001816 | gamma-Terpineol | CC(=C1CCC(CC1)(C)O)C | 11467 |
| IMPHY002047 | (2S,3R,4S,5S,6R)-2-[4-[1,3-Dihydroxy-2-[4-[(E)-3-hydroxyprop-1-enyl]-2,6-dimethoxyphenoxy]propyl]-2-methoxyphenoxy]-6-(hydroxymethyl)oxane-3,4,5-triol | OC/C=C/c1cc(OC)c(c(c1)OC)OC(C(c1ccc(c(c1)OC)O[C@@H]1O[C@H](CO)[C@H]([C@@H]([C@H]1O)O)O)O)CO | 11972318 |
| IMPHY002129 | : 2,2-Dimethyl-3-(3-methylenepent-4-enyl)oxirane | C=CC(=C)CCC1OC1(C)C | 122371 |
| IMPHY002825 | 2-(4-Methylphenyl)propan-2-ol | Cc1ccc(cc1)C(O)(C)C | 14529 |
| IMPHY002875 | Undecane | CCCCCCCCCCC | 14257 |
| IMPHY003104 | Decanoic acid | CCCCCCCCCC(=O)O | 2969 |
| IMPHY003485 | Myrcene | C=CC(=C)CCC=C(C)C | 31253 |
| IMPHY003490 | Coumarin | O=c1ccc2c(o1)cccc2 | 323 |
| IMPHY003495 | 2-Methoxy-4-vinylphenol | COc1cc(C=C)ccc1O | 332 |
| IMPHY003500 | Citric acid | OC(=O)C(CC(=O)O)(CC(=O)O)O | 311 |
| IMPHY003525 | Nonanal | CCCCCCCCC=O | 31289 |
| IMPHY003536 | Eugenol | C=CCc1ccc(c(c1)OC)O | 3314 |
| IMPHY003537 | Tetradecanal | CCCCCCCCCCCCCC=O | 31291 |
| IMPHY003545 | 4-Isopropylbenzaldehyde | O=Cc1ccc(cc1)C(C)C | 326 |
| IMPHY003567 | alpha-Fenchene | C=C1CC2C(C1CC2)(C)C | 28930 |
| IMPHY003616 | Bicyclogermacrene | C/C/1=CCC/C(=C/[C@H]2[C@@H](CC1)C2(C)C)/C | 13894537 |
| IMPHY003710 | (-)-Isopulegol | C[C@@H]1CC[C@H]([C@@H](C1)O)C(=C)C | 170833 |
| IMPHY003719 | beta-Copaene | CC([C@@H]1CC[C@]2([C@@H]3[C@H]1C2C(=C)CC3)C)C | 57339298 |
| IMPHY003798 | (Z)-alpha-Bisabolene | CC(=CC/C=C(C1CCC(=CC1)C)/C)C | 5352653 |
| IMPHY003807 | (E)-alpha-bisabolene | CC(=CC/C=C(/C1CCC(=CC1)C)C)C | 5315468 |
| IMPHY003915 | 2-Decenal | CCCCCCC/C=C/C=O | 5283345 |
| IMPHY003982 | gamma-Terpinene | CC1=CCC(=CC1)C(C)C | 7461 |
| IMPHY003992 | Hesperidin | COc1ccc(cc1O)[C@@H]1CC(=O)c2c(O1)cc(cc2O)O[C@@H]1O[C@H](CO[C@@H]2O[C@@H](C)[C@@H]([C@H]([C@H]2O)O)O)[C@H]([C@@H]([C@H]1O)O)O | 10621 |
| IMPHY004077 | Verbenone | CC1=CC(=O)C2CC1C2(C)C | 29025 |
| IMPHY004130 | Sinapic acid | COc1cc(/C=C/C(=O)O)cc(c1O)OC | 637775 |
| IMPHY004151 | Geranyl formate | O=COC/C=C(/CCC=C(C)C)C | 5282109 |
| IMPHY004286 | Longifolene | C=C1C2CCC3C1(C)CCCC(C23)(C)C | 289151 |
| IMPHY004433 | Chrysoeriol | COc1cc(ccc1O)c1cc(=O)c2c(o1)cc(cc2O)O | 5280666 |
| IMPHY003987 | Eugenyl glucoside | C=CCc1ccc(c(c1)OC)O[C@@H]1O[C@H](CO)[C@H]([C@@H]([C@H]1O)O)O | 3084296 |
| IMPHY004555 | 1,3,3-Trimethyl-2-oxabicyclo[2.2.2]oct-5-ene | CC12CCC(C=C1)C(O2)(C)C | 523035 |
| IMPHY004603 | : Coniferin | OC/C=C/c1ccc(c(c1)OC)O[C@@H]1O[C@H](CO)[C@H]([C@@H]([C@H]1O)O)O | 5280372 |
| IMPHY004619 | Quercetin | Oc1cc(O)c2c(c1)oc(c(c2=O)O)c1ccc(c(c1)O)O | 5280343 |
| IMPHY004660 | Luteolin | Oc1cc(O)c2c(c1)oc(cc2=O)c1ccc(c(c1)O)O | 5280445 |
| IMPHY004661 | Apigenin | Oc1ccc(cc1)c1cc(=O)c2c(o1)cc(cc2O)O | 5280443 |
| IMPHY004889 | Cinnamyl alcohol | OC/C=C/c1ccccc1 | 5315892 |
| IMPHY004977 | Syringin | OC/C=C/c1cc(OC)c(c(c1)OC)O[C@@H]1O[C@H](CO)[C@H]([C@@H]([C@H]1O)O)O | 5316860 |
| IMPHY005428 | Bergapten | COc1c2ccc(=O)oc2cc2c1cco2 | 2355 |
| IMPHY005587 | Umbelliferone | Oc1ccc2c(c1)oc(=O)cc2 | 5281426 |
| IMPHY005609 | beta-Sinensal | C=CC(=C)CC/C=C(/CC/C=C(/C=O)C)C | 5281535 |
| IMPHY005618 | Germacrene B | C/C/1=CCC/C(=C/CC(=C(C)C)CC1)/C | 5281519 |
| IMPHY005636 | Cortisone | OCC(=O)[C@@]1(O)CC[C@@H]2[C@]1(C)CC(=O)[C@H]1[C@H]2CCC2=CC(=O)CC[C@]12C | 222786 |
| IMPHY006106 | Limocitrin | COc1cc(ccc1O)c1oc2c(OC)c(O)cc(c2c(=O)c1O)O | 5489485 |
| IMPHY006145 | p-Cymene | Cc1ccc(cc1)C(C)C | 7463 |
| IMPHY006177 | Methyl geranate | COC(=O)/C=C(/CCC=C(C)C)C | 5365910 |
| IMPHY006279 | 2-Phenylethanol | OCCc1ccccc1 | 6054 |
| IMPHY006324 | Linalyl propionate | CCC(=O)OC(CCC=C(C)C)(C=C)C | 61098 |
| IMPHY006347 | Hexanal | CCCCCC=O | 6184 |
| IMPHY006350 | Psoralen | O=c1ccc2c(o1)cc1c(c2)cco1 | 6199 |
| IMPHY006362 | Ascorbic acid | OC[C@@H]([C@H]1OC(=O)C(=C1O)O)O | 54670067 |
| IMPHY006550 | Thymol | Cc1ccc(c(c1)O)C(C)C | 6989 |
| IMPHY006947 | 1-Decanol | CCCCCCCCCCO | 8174 |
| IMPHY006948 | beta-Terpineol | CC(=C)C1CCC(CC1)(C)O | 8748 |
| IMPHY006950 | Tricyclene | CC12C3C1CC(C2(C)C)C3 | 79035 |
| IMPHY006970 | Decanal | CCCCCCCCCC=O | 8175 |
| IMPHY006981 | Indole | c1ccc2c(c1)[nH]cc2 | 798 |
| IMPHY007067 | Linalyl acetate | C=CC(OC(=O)C)(CCC=C(C)C)C | 8294 |
| IMPHY007068 | Undecanoic acid | CCCCCCCCCCC(=O)O | 8180 |
| IMPHY007076 | Undecanal | CCCCCCCCCCC=O | 8186 |
| IMPHY007099 | Decyl acetate | CCCCCCCCCCOC(=O)C | 8167 |
| IMPHY007100 | 1-Dodecanol | CCCCCCCCCCCCO | 8193 |
| IMPHY007137 | Methyl 2-(methylamino)benzoate | COC(=O)c1ccccc1NC | 6826 |
| IMPHY007204 | Dodecanal | CCCCCCCCCCCC=O | 8194 |
| IMPHY007206 | Dodecyl acetate | CCCCCCCCCCCCOC(=O)C | 8205 |
| IMPHY007207 | Tangeretin | COc1ccc(cc1)c1cc(=O)c2c(o1)c(OC)c(c(c2OC)OC)OC | 68077 |
| IMPHY007214 | Octyl acetate | CCCCCCCCOC(=O)C | 8164 |
| IMPHY007221 | Methyl decanoate | CCCCCCCCCC(=O)OC | 8050 |
| IMPHY007302 | Hexyl acetate | CCCCCCOC(=O)C | 8908 |
| IMPHY007331 | 6-Methyl-5-hepten-2-one | CC(=O)CCC=C(C)C | 9862 |
| IMPHY007376 | beta-Cubebene | CC([C@@H]1CC[C@H]([C@]23[C@H]1[C@H]2C(=C)CC3)C)C | 93081 |
| IMPHY007421 | Citronellyl acetate | CC(CCC=C(C)C)CCOC(=O)C | 9017 |
| IMPHY007450 | Oxalic acid | OC(=O)C(=O)O | 971 |
| IMPHY007528 | Cadinane | C[C@H]1CC[C@@H]2[C@@H](C1)[C@@H](CC[C@@H]2C)C(C)C | 9548708 |
| IMPHY007620 | 1-Octanol | CCCCCCCCO | 957 |
| IMPHY007840 | Spathulenol | C=C1CC[C@@H]2[C@H]([C@H]3[C@H]1CC[C@]3(C)O)C2(C)C | 92231 |
| IMPHY007853 | Camphane | CC1(C)C2CCC1(C)CC2 | 92108 |
| IMPHY008150 | 1-Methyl-4-(prop-1-en-2-yl)benzene | Cc1ccc(cc1)C(=C)C | 62385 |
| IMPHY008597 | Nonyl acetate | CCCCCCCCCOC(=O)C | 8918 |
| IMPHY008724 | Isorhamnetin | COc1cc(ccc1O)c1oc2cc(O)cc(c2c(=O)c1O)O | 5281654 |
| IMPHY008942 | Citrusin A | OC/C=C/c1ccc(c(c1)OC)OC(C(c1ccc(c(c1)OC)O[C@@H]1O[C@H](CO)[C@H]([C@@H]([C@H]1O)O)O)O)CO | 101601468 |
| IMPHY009317 | Limocitrol | COc1cc(ccc1O)c1oc2c(OC)c(O)c(c(c2c(=O)c1O)O)OC | 12311234 |
| IMPHY009360 | Nootkatone | O=C1C[C@@H](C)[C@]2(C(=C1)CC[C@H](C2)C(=C)C)C | 1268142 |
| IMPHY009389 | Pentadecane | CCCCCCCCCCCCCCC | 12391 |
| IMPHY009642 | 2-Nonanone | CCCCCCCC(=O)C | 13187 |
| IMPHY009739 | 5-Isopropylbicyclo[3.1.0]hexan-2-one | O=C1CCC2(C1C2)C(C)C | 92784 |
| IMPHY009743 | beta-Gurjunene | C[C@@H]1CC[C@@H]2[C@H]([C@H]3[C@@H]1CCC3=C)C2(C)C | 6450812 |
| IMPHY009762 | Heptyl acetate | CCCCCCCOC(=O)C | 8159 |
| IMPHY009852 | Methyl citronellate | COC(=O)CC(CCC=C(C)C)C | 61290 |
| IMPHY009853 | Naphthalene | c1ccc2c(c1)cccc2 | 931 |
| IMPHY009858 | 1-Undecanol | CCCCCCCCCCCO | 8184 |
| IMPHY009872 | p-Mentha-3,8-diene | CC1CCC(=CC1)C(=C)C | 521851 |
| IMPHY010000 | Dodecane | CCCCCCCCCCCC | 8182 |
| IMPHY010072 | Eucalyptol | CC12CCC(CC1)C(O2)(C)C | 2758 |
| IMPHY010080 | beta-Elemene | C=C[C@]1(C)CC[C@H](C[C@H]1C(=C)C)C(=C)C | 6918391 |
| IMPHY010318 | Geranyl propionate | CCC(=O)OC/C=C(/CCC=C(C)C)C | 5355853 |
| IMPHY010781 | Limonene oxide, cis-(-)- | CC(=C)[C@H]1CC[C@]2([C@@H](C1)O2)C | 6452061 |
| IMPHY011004 | p-Mentha-1,3,8-triene | CC1=CC=C(CC1)C(=C)C | 176983 |
| IMPHY011016 | 2,6-Dimethyl-5-heptenal | O=CC(CCC=C(C)C)C | 61016 |
| IMPHY011086 | alpha-Sinensal | C=C/C(=C/C/C=C(/CC/C=C(/C=O)C)C)/C | 5281534 |
| IMPHY011215 | Tetradecane | CCCCCCCCCCCCCC | 12389 |
| IMPHY011354 | trans-Sabinene hydrate acetate | CC(=O)O[C@@]1(C)CC[C@@]2(C1C2)C(C)C | 6427504 |
| IMPHY011392 | 3-Carene | CC1=CCC2C(C1)C2(C)C | 26049 |
| IMPHY011395 | Scoparone | COc1cc2oc(=O)ccc2cc1OC | 8417 |
| IMPHY011396 | 4-Carvomenthenol | CC1=CCC(CC1)(O)C(C)C | 11230 |
| IMPHY011586 | Bergamottin | C/C(=CCOc1c2ccoc2cc2c1ccc(=O)o2)/CCC=C(C)C | 5471349 |
| IMPHY011519 | alpha-Terpinyl acetate | CC(=O)OC(C1CCC(=CC1)C)(C)C | 111037 |
| IMPHY011541 | Scopoletin | COc1cc2ccc(=O)oc2cc1O | 5280460 |
| IMPHY011542 | beta-Eudesmol | C=C1CCC[C@]2([C@H]1C[C@@H](CC2)C(O)(C)C)C | 91457 |
| IMPHY011549 | Citropten | COc1cc(OC)c2c(c1)oc(=O)cc2 | 2775 |
| IMPHY011552 | 1R)-2-methyl-5-propan-2-ylbicyclo[3.1.0]hex-2-ene | CC1=CCC2([C@@H]1C2)C(C)C | 6451618 |
| IMPHY011562 | 2-Hexenal | CCC/C=C/C=O | 5281168 |
| IMPHY011581 | alpha-Selinene | CC1=CCC[C@]2([C@H]1C[C@@H](CC2)C(=C)C)C | 10856614 |
| IMPHY011586 | (S,1Z,6Z)-8-Isopropyl-1-methyl-5-methylenecyclodeca-1,6-diene | C/C/1=C/CCC(=C)/C=C[C@@H](CC1)C(C)C | 91723653 |
| IMPHY011590 | d-Borneol | O[C@@H]1C[C@H]2C([C@@]1(C)CC2)(C)C | 61060 |
| IMPHY011599 | Terpinolene | CC1=CCC(=C(C)C)CC1 | 11463 |
| IMPHY011609 | alpha-Carotene | C/C(=CC=CC=C(C=CC=C(C=CC1=C(C)CCCC1(C)C)/C)/C)/C=C/C=C(/C=C/[C@H]1C(=CCCC1(C)C)C)C | 6419725 |
| IMPHY011632 | Farnesol | OC/C=C(/CC/C=C(/CCC=C(C)C)C)C | 445070 |
| IMPHY011643 | alpha-Terpinene | CC1=CC=C(CC1)C(C)C | 7462 |
| IMPHY011647 | Geranyl acetate | C/C(=CCOC(=O)C)/CCC=C(C)C | 1549026 |
| IMPHY011648 | Neryl acetate | C/C(=C/COC(=O)C)/CCC=C(C)C | 1549025 |
| IMPHY011657 | cis-beta-Farnesene | C=CC(=C)CC/C=C(CCC=C(C)C)/C | 5317319 |
| IMPHY011658 | beta-Farnesene | C=CC(=C)CC/C=C(/CCC=C(C)C)C | 5281517 |
| IMPHY011659 | alpha-Muurolene | CC1=C[C@@H]2[C@H](CC1)C(=CC[C@H]2C(C)C)C | 12306047 |
| IMPHY011668 | beta-Santalene | CC(=CCC[C@]1(C)[C@H]2CC[C@@H](C1=C)C2)C | 10889018 |
| IMPHY011687 | trans-3-Hexenyl acetate | CC/C=C/CCOC(=O)C | 5352557 |
| IMPHY011761 | Humulene | C/C/1=CCC(C)(C)/C=C/C/C(=C/CC1)/C | 5281520 |
| IMPHY011789 | Citral | O=C/C=C(/CCC=C(C)C)C | 638011 |
| IMPHY011790 | Neral | O=C/C=C(CCC=C(C)C)/C | 643779 |
| IMPHY011792 | gamma-Muurolene | CC1=C[C@@H]2[C@H](CC1)C(=C)CC[C@H]2C(C)C | 12313020 |
| IMPHY011793 | (+)-gamma-Cadinene | CC1=C[C@@H]2[C@@H](CC1)C(=C)CC[C@H]2C(C)C | 6432404 |
| IMPHY011794 | (1E,4E,8E)-2,6,6,9-tetramethylcycloundeca-1,4,8-triene | C/C/1=C/CC(C)(C)/C=CC/C(=CCC1)/C | 6508206 |
| IMPHY011797 | Oleic acid | CCCCCCCC/C=CCCCCCCCC(=O)O | 445639 |
| IMPHY011817 | alpha-Farnesene | C=C/C(=C/C/C=C(/CCC=C(C)C)C)/C | 5281516 |
| IMPHY011839 | (Z)-gamma-bisabolene | CC(=CCC/C(=C1/CCC(=CC1)C)/C)C | 3033866 |
| IMPHY011890 | Elemol | C=C[C@]1(C)CC[C@H](C[C@H]1C(=C)C)C(O)(C)C | 92138 |
| IMPHY011896 | Valencene | CC(=C)[C@@H]1CCC2=CCC[C@H]([C@@]2(C1)C)C | 9855795 |
| IMPHY011938 | gamma-Eudesmol | CC1=C2C[C@@H](CC[C@]2(CCC1)C)C(O)(C)C | 6432005 |
| IMPHY011957 | (+)-delta-Cadinene | CC1=C[C@@H]2C(=C(C)CC[C@H]2C(C)C)CC1 | 441005 |
| IMPHY011965 | (+)-beta-Phellandrene | CC([C@@H]1CCC(=C)C=C1)C | 442484 |
| IMPHY011973 | (-)-cis-Carveol | CC(=C)[C@@H]1CC=C([C@@H](C1)O)C | 330573 |
| IMPHY011974 | 4-Hydroxycinnamic acid | OC(=O)/C=C/c1ccc(cc1)O | 637542 |
| IMPHY011988 | (-)-trans-Carveol | CC(=C)[C@@H]1CC=C([C@H](C1)O)C | 94221 |
| IMPHY012036 | Camphor | O=C1CC2C(C1(C)CC2)(C)C | 2537 |
| IMPHY012058 | Linalool | C=CC(CCC=C(C)C)(O)C | 6549 |
| IMPHY012061 | alpha-Pinene | CC1=CCC2CC1C2(C)C | 6654 |
| IMPHY012075 | Carvone | CC(=C)C1CC=C(C(=O)C1)C | 7439 |
| IMPHY012086 | Citronellal | O=CCC(CCC=C(C)C)C | 7794 |
| IMPHY012097 | Methyl octanoate | CCCCCCCC(=O)OC | 8091 |
| IMPHY012104 | Citronellol | OCCC(CCC=C(C)C)C | **8842** |
| IMPHY012147 | beta-Pinene | C=C1CCC2CC1C2(C)C | 14896 |
| IMPHY012152 | alpha-Fenchol | O[C@H]1[C@@]2(C)CC[C@@H](C1(C)C)C2 | 439711 |
| IMPHY012157 | Perillaldehyde | O=CC1=CCC(CC1)C(=C)C | 16441 |
| IMPHY012160 | alpha-Terpineol | CC1=CCC(CC1)C(O)(C)C | 17100 |
| IMPHY012165 | Sabinene | C=C1CCC2(C1C2)C(C)C | 18818 |
| IMPHY012175 | D-Limonene | CC1=CC[C@@H](CC1)C(=C)C | 440917 |
| IMPHY012252 | : Eriocitrin | O[C@@H]1[C@@H](O)[C@H](Oc2cc(O)c3c(c2)O[C@@H](CC3=O)c2ccc(c(c2)O)O)O[C@@H]([C@H]1O)CO[C@@H]1O[C@@H](C)[C@@H]([C@H]([C@H]1O)O)O | 83489 |
| IMPHY012255 | (+)-trans-Piperitenol | CC1=C[C@@H]([C@H](CC1)C(C)C)O | 85568 |
| IMPHY012261 | alpha-Bergamotene | CC(=CCCC1(C)C2CC=C(C1C2)C)C | 86608 |
| IMPHY012265 | (1r,3s,5r)-6,6-Dimethyl-2-methylidenebicyclo[3.1.1]heptan-3-ol | C=C1[C@@H](O)C[C@H]2C[C@@H]1C2(C)C | 88302 |
| IMPHY012464 | Clionasterol | CC[C@H](C(C)C)CC[C@H]([C@H]1CC[C@@H]2[C@]1(C)CC[C@H]1[C@H]2CC=C2[C@]1(C)CC[C@@H](C2)O)C | 457801 |
| IMPHY012586 | (-)-alpha-Cadinol | CC1=CC2C(CC1)[C@@](C)(O)CC[C@@H]2C(C)C | 6431302 |
| IMPHY012589 | 3-(1,5-Dimethyl-4-hexenyl)-6-methylene-1-cyclohexene | CC(C1CCC(=C)C=C1)CCC=C(C)C | 519764 |
| IMPHY012623 | trans-3-Caren-2-ol | CC1=CCC2C(C1O)C2(C)C | 576906 |
| IMPHY012654 | Nerol | OC/C=C(CCC=C(C)C)/C | 643820 |
| IMPHY012665 | Levomenol | CC(=CCC[C@@]([C@H]1CCC(=CC1)C)(O)C)C | 442343 |
| IMPHY012667 | Caryophyllene oxide | C=C1CC[C@H]2O[C@@]2(CC[C@@H]2[C@@H]1CC2(C)C)C | 1742210 |
| IMPHY012739 | (Z)-beta-Ocimene | C=C/C(=CCC=C(C)C)/C | 5320250 |
| IMPHY012907 | trans-Sesquisabinene hydrate | CC(=CCCC([C@]12CC[C@](C2C1)(C)O)C)C | 6428444 |
| IMPHY012921 | gamma-Elemene | C=C[C@]1(C)CCC(=C(C)C)C[C@H]1C(=C)C | 6432312 |
| IMPHY013093 | delta-Elemene | C=C[C@@]1(C)CCC(=C[C@@H]1C(=C)C)C(C)C | 12309449 |
| IMPHY013133 | (Z)-p-Menth-2-en-1-ol | CC([C@@H]1CC[C@](C=C1)(C)O)C | 13918681 |
| IMPHY013575 | Campherenol | CC(=CCCC1(C)C2CCC1(C)[C@@H](C2)O)C | 91747494 |
| IMPHY013709 | Citrusin D | OCC1OC(OC/C=Cc2ccc(c(c2)OC)O)C(C(C1O)O)O | 131752609 |
| IMPHY013836 | Fenchone | O=C1C2(C)CCC(C1(C)C)C2 | 14525 |
| IMPHY014112 | 3,6-Octadienal, 3,7-dimethyl- | O=CC/C(=C/CC=C(C)C)/C | 6428928 |
| IMPHY014690 | -)-Globulol | C[C@@H]1CC[C@@H]2[C@@H]1[C@H]1[C@H](C1(C)C)CC[C@@]2(C)O | 12304985 |
| IMPHY014699 | D-Camphor | O=C1C[C@@H]2C([C@@]1(C)CC2)(C)C | 159055 |
| IMPHY014708 | beta-Selinene | C=C1CCC[C@]2([C@H]1C[C@@H](CC2)C(=C)C)C | 442393 |
| IMPHY014806 | Caswell No. 264AB | CC([C@@H]1CC[C@H]([C@]23[C@H]1[C@H]2C(=CC3)C)C)C | 442359 |
| IMPHY014811 | alpha-Phellandrene | CC1=CCC(C=C1)C(C)C | 7460 |
| IMPHY014817 | Aromadendrene | CC1CCC2C1C1C(C1(C)C)CCC2=C | 91354 |
| IMPHY014831 | beta-Caryophyllene | C/C/1=CCCC(=C)[C@@H]2[C@@H](CC1)C(C2)(C)C | 5281515 |
| IMPHY014835 | (E)-beta-ocimene | C=C/C(=C/CC=C(C)C)/C | 5281553 |
| IMPHY014836 | beta-Sitosterol | CC[C@@H](C(C)C)CC[C@H]([C@H]1CC[C@@H]2[C@]1(C)CC[C@H]1[C@H]2CC=C2[C@]1(C)CC[C@@H](C2)O)C | 222284 |
| IMPHY014847 | Bornyl acetate | CC(=O)OC1CC2C(C1(C)CC2)(C)C | 6448 |
| IMPHY014852 | Camphene | C=C1C2CCC(C1(C)C)C2 | 6616 |
| IMPHY014863 | cis-alpha-Bergamotene | CC(=CCCC1(C)[C@@H]2CC=C([C@H]1C2)C)C | 91753502 |
| IMPHY014873 | 2-Cyclohexen-1-ol, 3-methyl-6-(1-methylethyl)-, (1R,6S)-rel- | CC1=C[C@@H]([C@@H](CC1)C(C)C)O | 85567 |
| IMPHY014874 | cis-Sabinene hydrate | C[C@@H]1CC[C@@]2(C1C2)C(C)C | 101629835 |
| IMPHY014907 | 6-Epi-beta-bisabolol | CC(=CCC[C@@H]([C@@]1(O)CCC(=CC1)C)C)C | 12300148 |
| IMPHY014914 | Fenchol | OC1C2(C)CCC(C1(C)C)C2 | 15406 |
| IMPHY014923 | Geraniol | OC/C=C(/CCC=C(C)C)C | 637566 |
| IMPHY014979 | (-)-Limonene | CC1=CC[C@H](CC1)C(=C)C | 439250 |
| IMPHY014986 | Ledol | C[C@@H]1CC[C@H]2[C@@H]1[C@H]1[C@H](C1(C)C)CC[C@@]2(C)O | 92812 |
| IMPHY014988 | Limonene | CC1=CCC(CC1)C(=C)C | 22311 |
| IMPHY014989 | trans-Linalool oxide | C=C[C@]1(C)CC[C@H](O1)C(O)(C)C | 6432254 |
| IMPHY015004 | Menthone | C[C@@H]1CC[C@H](C(=O)C1)C(C)C | 26447 |
| IMPHY015022 | Nerolidol | C=CC(CC/C=C(/CCC=C(C)C)C)(O)C | 5284507 |
| IMPHY015042 | Piperitone | CC1=CC(=O)C(CC1)C(C)C | 6987 |
| IMPHY015047 | Rutin | Oc1cc(O)c2c(c1)oc(c(c2=O)O[C@@H]1O[C@H](CO[C@@H]2O[C@@H](C)[C@@H]([C@H]([C@H]2O)O)O)[C@H]([C@@H]([C@H]1O)O)O)c1ccc(c(c1)O)O | 5280805 |
| IMPHY015059 | Rose oxide | CC1CCOC(C1)C=C(C)C | 27866 |
| IMPHY015085 | Tetradec-2-enal | CCCCCCCCCCCC=CC=O | 116625 |
| IMPHY015094 | (+)-trans-Limonene oxide | CC(=C)[C@@H]1CC[C@]2([C@@H](C1)O2)C | 449290 |
| IMPHY015118 | Ylangene | CC(C1CC[C@]2(C3C1C2C(=CC3)C)C)C | 6432119 |
| IMPHY015123 | alpha-Copaene | CC([C@@H]1CC[C@]2([C@@H]3[C@H]1C2C(=CC3)C)C)C | 70678558 |
| IMPHY015128 | T-Muurolol | CC1=C[C@@H]2[C@H](CC1)[C@@](C)(O)CC[C@H]2C(C)C | 3084331 |
| IMPHY015497 | Tert-butylbenzene | CC(c1ccccc1)(C)C | 7366 |
| IMPHY015552 | Cedranyl acetate | CC(=O)O[C@]1(C)CC[C@@]23C[C@@H]1C(C)(C)[C@@H]2CC[C@H]3C | 13918856 |
| IMPHY015554 | 5alpha-Cholestan-3beta-ol, 2-methylene- | CC(CCC[C@H]([C@H]1CC[C@@H]2[C@]1(C)CC[C@H]1[C@H]2CC[C@@H]2[C@]1(C)CC(=C)[C@@H](C2)O)C)C | 22213932 |
| IMPHY015660 | Ethyl decanoate | CCCCCCCCCC(=O)OCC | 8048 |
| IMPHY015698 | Geranyl vinyl ether | C=COC/C=C(/CCC=C(C)C)C | 5365842 |
| IMPHY015713 | Hept-1-enyl acetate | CCCCC/C=C/OC(=O)C | 5463146 |
| IMPHY015876 | 2,3-Epoxygerianial | O=CC1OC1(C)CCC=C(C)C | 534994 |
| IMPHY015948 | Terpinyl propionate | CCC(=O)OC(C1CCC(=CC1)C)(C)C | 62328 |
| IMPHY016012 | Allo-Aromadendrene | C[C@@H]1CC[C@H]2[C@@H]1C1C(C1(C)C)CCC2=C | 42608158 |
| IMPHY016027 | trans-Sabinene hydrate | CC([C@@]12CC[C@](C2C1)(C)O)C | 12315151 |
| IMPHY016053 | Viridiflorol | C[C@@H]1CC[C@H]2[C@@H]1[C@H]1[C@H](C1(C)C)CC[C@]2(C)O | 11996452 |
| IMPHY016054 | trans-alpha-Bergamotene | CC(=CCC[C@]1(C)[C@H]2CC=C([C@@H]1C2)C)C | 6429302 |
| IMPHY016781 | Bicyclo[2.2.1]heptan-1-ol | OC12CCC(C2)CC1 | 524266 |
| IMPHY016801 | Mentha-1,4,8-triene | CC1=CCC(=CC1)C(=C)C | 527141 |
| IMPHY017676 | 2'-Methoxyacetophenone | COc1ccccc1C(=O)C | 68481 |
| IMPHY017713 | Octadienyl formate | CCCCC=CC=COC=O | 73472835 |
| IMPHY000005 | Thiamine | OCCc1sc[n+](c1C)Cc1cnc(nc1N)C | 1130 |
| IMPHY000399 | beta-Bisabolene | CC(=CCCC(=C)[C@H]1CCC(=CC1)C)C | 10104370 |
| IMPHY000545 | O-Cymene | CC(c1ccccc1C)C | 10703 |
| IMPHY000795 | Octanal | CCCCCCCC=O | 454 |
| IMPHY003485 | Myrcene | C=CC(=C)CCC=C(C)C | 31253 |
| IMPHY003525 | Nonanal | CCCCCCCCC=O | 31289 |
| IMPHY003567 | alpha-Fenchene | C=C1CC2C(C1CC2)(C)C | 28930 |
| IMPHY003616 | Bicyclogermacrene | C/C/1=CCC/C(=C/[C@H]2[C@@H](CC1)C2(C)C)/C | 13894537 |
| IMPHY003710 | (-)-Isopulegol | C[C@@H]1CC[C@H]([C@@H](C1)O)C(=C)C | 170833 |
| IMPHY003798 | (Z)-alpha-Bisabolene | CC(=CC/C=C(C1CCC(=CC1)C)/C)C | 5352653 |
| IMPHY003982 | gamma-Terpinene | CC1=CCC(=CC1)C(C)C | 7461 |
| IMPHY004150 | Neryl formate | O=COC/C=C(CCC=C(C)C)/C | 5354882 |
| IMPHY004151 | Geranyl formate | O=COC/C=C(/CCC=C(C)C)C | 5282109 |
| IMPHY005609 | beta-Sinensal | C=CC(=C)CC/C=C(/CC/C=C(/C=O)C)C | 5281535 |
| IMPHY006145 | p-Cymene | Cc1ccc(cc1)C(C)C | 7463 |
| IMPHY006177 | Methyl geranate | COC(=O)/C=C(/CCC=C(C)C)C | 5365910 |
| IMPHY006324 | Linalyl propionate | CCC(=O)OC(CCC=C(C)C)(C=C)C | 61098 |
| IMPHY006347 | Hexanal | CCCCCC=O | 6184 |
| IMPHY006550 | Thymol | Cc1ccc(c(c1)O)C(C)C | 6989 |
| IMPHY006948 | beta-Terpineol | CC(=C)C1CCC(CC1)(C)O | 8748 |
| IMPHY006950 | Tricyclene | CC12C3C1CC(C2(C)C)C3 | 79035 |
| IMPHY006970 | Decanal | CCCCCCCCCC=O | 8175 |
| IMPHY007067 | Linalyl acetate | C=CC(OC(=O)C)(CCC=C(C)C)C | 8294 |
| IMPHY007076 | Undecanal | CCCCCCCCCCC=O | 8186 |
| IMPHY007137 | Methyl 2-(methylamino)benzoate | COC(=O)c1ccccc1NC | 6826 |
| IMPHY007171 | 1-Hexanol | CCCCCCO | 8103 |
| IMPHY007204 | : Dodecanal | CCCCCCCCCCCC=O | 8194 |
| IMPHY007214 | Octyl acetate | CCCCCCCCOC(=O)C | 8164 |
| IMPHY007276 | Nonan-1-ol | CCCCCCCCCO | 8914 |
| IMPHY007302 | Hexyl acetate | CCCCCCOC(=O)C | 8908 |
| IMPHY007331 | 6-Methyl-5-hepten-2-one | CC(=O)CCC=C(C)C | 9862 |
| IMPHY007421 | Citronellyl acetate | CC(CCC=C(C)C)CCOC(=O)C | 9017 |
| IMPHY007620 | : 1-Octanol | CCCCCCCCO | 957 |
| IMPHY007840 | Spathulenol | C=C1CC[C@@H]2[C@H]([C@H]3[C@H]1CC[C@]3(C)O)C2(C)C | 92231 |
| IMPHY008150 | 1-Methyl-4-(prop-1-en-2-yl)benzene | Cc1ccc(cc1)C(=C)C | 62385 |
| IMPHY008597 | Nonyl acetate | CCCCCCCCCOC(=O)C | 8918 |
| IMPHY009762 | Heptyl acetate | CCCCCCCOC(=O)C | 8159 |
| IMPHY009764 | 2,10-Epoxypinane | CC1(C)C2CCC3(C1C2)CO3 | 93046 |
| IMPHY009871 | Isoterpinolene | CC1CCC(=C(C)C)C=C1 | 102443 |
| IMPHY010072 | Eucalyptol | CC12CCC(CC1)C(O2)(C)C | 2758 |
| IMPHY010080 | beta-Elemene | C=C[C@]1(C)CC[C@H](C[C@H]1C(=C)C)C(=C)C | 6918391 |
| IMPHY010318 | Geranyl propionate | CCC(=O)OC/C=C(/CCC=C(C)C)C | 5355853 |
| IMPHY010781 | Limonene oxide, cis-(-)- | CC(=C)[C@H]1CC[C@]2([C@@H](C1)O2)C | 6452061 |
| IMPHY011016 | 2,6-Dimethyl-5-heptenal | O=CC(CCC=C(C)C)C | 61016 |
| IMPHY011086 | alpha-Sinensal | C=C/C(=C/C/C=C(/CC/C=C(/C=O)C)C)/C | 5281534 |
| IMPHY011392 | 3-Carene | CC1=CCC2C(C1)C2(C)C | 26049 |
| IMPHY011396 | 4-Carvomenthenol | CC1=CCC(CC1)(O)C(C)C | 11230 |
| IMPHY011519 | alpha-Terpinyl acetate | CC(=O)OC(C1CCC(=CC1)C)(C)C | 111037 |
| IMPHY011552 | (1R)-2-methyl-5-propan-2-ylbicyclo[3.1.0]hex-2-ene | CC1=CCC2([C@@H]1C2)C(C)C | 6451618 |
| IMPHY011562 | 2-Hexenal | CCC/C=C/C=O | 5281168 |
| IMPHY011570 | 2Z,6E)-Farnesyl acetate | C/C(=CCC/C(=CCOC(=O)C)/C)/CCC=C(C)C | 1551480 |
| IMPHY011586 | (S,1Z,6Z)-8-Isopropyl-1-methyl-5-methylenecyclodeca-1,6-diene | C/C/1=C/CCC(=C)/C=C[C@@H](CC1)C(C)C | 91723653 |
| IMPHY011588 | cis-3-Hexen-1-ol | OCC/C=CCC | 5281167 |
| IMPHY011590 | d-Borneol | O[C@@H]1C[C@H]2C([C@@]1(C)CC2)(C)C | 61060 |
| IMPHY011599 | Terpinolene | CC1=CCC(=C(C)C)CC1 | 11463 |
| IMPHY011643 | alpha-Terpinene | CC1=CC=C(CC1)C(C)C | 7462 |
| IMPHY011647 | Geranyl acetate | C/C(=CCOC(=O)C)/CCC=C(C)C | 1549026 |
| IMPHY011648 | Neryl acetate | C/C(=C/COC(=O)C)/CCC=C(C)C | 1549025 |
| IMPHY011657 | cis-beta-Farnesene | C=CC(=C)CC/C=C(CCC=C(C)C)/C | 5317319 |
| IMPHY011658 | beta-Farnesene | C=CC(=C)CC/C=C(/CCC=C(C)C)C | 5281517 |
| IMPHY011668 | beta-Santalene | CC(=CCC[C@]1(C)[C@H]2CC[C@@H](C1=C)C2)C | 10889018 |
| IMPHY011695 | cis-2-Hexen-1-ol | CCC/C=CCO | 5324489 |
| IMPHY011761 | Humulene | C/C/1=CCC(C)(C)/C=C/C/C(=C/CC1)/C | 5281520 |
| IMPHY011789 | Citral | O=C/C=C(/CCC=C(C)C)C | 638011 |
| IMPHY011790 | Neral | O=C/C=C(CCC=C(C)C)/C | 643779 |
| IMPHY011817 | alpha-Farnesene | C=C/C(=C/C/C=C(/CCC=C(C)C)C)/C | 5281516 |
| IMPHY011957 | (+)-delta-Cadinene | CC1=C[C@@H]2C(=C(C)CC[C@H]2C(C)C)CC1 | 441005 |
| IMPHY011965 | (+)-beta-Phellandrene | CC([C@@H]1CCC(=C)C=C1)C | 442484 |
| IMPHY012058 | Linalool | C=CC(CCC=C(C)C)(O)C | 6549 |
| IMPHY012061 | alpha-Pinene | CC1=CCC2CC1C2(C)C | 6654 |
| IMPHY012075 | Carvone | CC(=C)C1CC=C(C(=O)C1)C | 7439 |
| IMPHY012086 | Citronellal | O=CCC(CCC=C(C)C)C | 7794 |
| IMPHY012104 | Citronellol | OCCC(CCC=C(C)C)C | 8842 |
| IMPHY012147 | beta-Pinene | C=C1CCC2CC1C2(C)C | 14896 |
| IMPHY012152 | alpha-Fenchol | O[C@H]1[C@@]2(C)CC[C@@H](C1(C)C)C2 | 439711 |
| IMPHY012160 | alpha-Terpineol | CC1=CCC(CC1)C(O)(C)C | 17100 |
| IMPHY012165 | Sabinene | C=C1CCC2(C1C2)C(C)C | 18818 |
| IMPHY012205 | Sabinene hydrate | CC(C12CCC(C2C1)(C)O)C | 62367 |
| IMPHY012261 | alpha-Bergamotene | CC(=CCCC1(C)C2CC=C(C1C2)C)C | 86608 |
| IMPHY012586 | (-)-alpha-Cadinol | CC1=CC2C(CC1)[C@@](C)(O)CC[C@@H]2C(C)C | 6431302 |
| IMPHY012654 | Nerol | OC/C=C(CCC=C(C)C)/C | 643820 |
| IMPHY012665 | Levomenol | CC(=CCC[C@@]([C@H]1CCC(=CC1)C)(O)C)C | 442343 |
| IMPHY012667 | Caryophyllene oxide | C=C1CC[C@H]2O[C@@]2(CC[C@@H]2[C@@H]1CC2(C)C)C | 1742210 |
| IMPHY012739 | (Z)-beta-Ocimene | C=C/C(=CCC=C(C)C)/C | 5320250 |
| IMPHY012907 | trans-Sesquisabinene hydrate | CC(=CCCC([C@]12CC[C@](C2C1)(C)O)C)C | 6428444 |
| IMPHY012920 | 2-Furanmethanol, 5-ethenyltetrahydro-alpha,alpha,5-trimethyl-, cis | C=C[C@@]1(C)CC[C@H](O1)C(O)(C)C | 11116492 |
| IMPHY013093 | delta-Elemene | C=C[C@@]1(C)CCC(=C[C@@H]1C(=C)C)C(C)C | 12309449 |
| IMPHY013133 | (Z)-p-Menth-2-en-1-ol | CC([C@@H]1CC[C@](C=C1)(C)O)C | 13918681 |
| IMPHY013575 | Campherenol | CC(=CCCC1(C)C2CCC1(C)[C@@H](C2)O)C | 91747494 |
| IMPHY014112 | 3,6-Octadienal, 3,7-dimethyl- | O=CC/C(=C/CC=C(C)C)/C | 6428928 |
| IMPHY014811 | alpha-Phellandrene | CC1=CCC(C=C1)C(C)C | 7460 |
| IMPHY014831 | beta-Caryophyllene | C/C/1=CCCC(=C)[C@@H]2[C@@H](CC1)C(C2)(C)C | 5281515 |
| IMPHY014835 | (E)-beta-ocimene | C=C/C(=C/CC=C(C)C)/C | 5281553 |
| IMPHY014847 | Bornyl acetate | CC(=O)OC1CC2C(C1(C)CC2)(C)C | 6448 |
| IMPHY014852 | Camphene | C=C1C2CCC(C1(C)C)C2 | 6616 |
| IMPHY014863 | cis-alpha-Bergamotene | CC(=CCCC1(C)[C@@H]2CC=C([C@H]1C2)C)C | 91753502 |
| IMPHY014874 | cis-Sabinene hydrate | C[C@@H]1CC[C@@]2(C1C2)C(C)C | 101629835 |
| IMPHY014923 | Geraniol | OC/C=C(/CCC=C(C)C)C | 637566 |
| IMPHY014988 | Limonene | CC1=CCC(CC1)C(=C)C | 22311 |
| IMPHY015022 | Nerolidol | C=CC(CC/C=C(/CCC=C(C)C)C)(O)C | 5284507 |
| IMPHY015094 | (+)-trans-Limonene oxide | CC(=C)[C@@H]1CC[C@]2([C@@H](C1)O2)C | 449290 |
| IMPHY015095 | 2-Cyclohexen-1-ol, 1-methyl-4-(1-methylethyl)-, trans- | CC([C@@H]1CC[C@@](C=C1)(C)O)C | 122484 |
| IMPHY016054 | trans-alpha-Bergamotene | CC(=CCC[C@]1(C)[C@H]2CC=C([C@@H]1C2)C)C | 6429302 |
| IMPHY016781 | Bicyclo[2.2.1]heptan-1-ol | OC12CCC(C2)CC1 | 524266 |
| IMPHY006654 | Xanthyletin | CC1(C)C=Cc2c(O1)cc1c(c2)ccc(=O)o1 | 65188 |
| IMPHY014836 | beta-Sitosterol | CC[C@@H](C(C)C)CC[C@H]([C@H]1CC[C@@H]2[C@]1(C)CC[C@H]1[C@H]2CC=C2[C@]1(C)CC[C@@H](C2)O)C | 222284 |
| IMPHY014842 | Stigmasterol | CC[C@@H](C(C)C)/C=C/[C@H]([C@H]1CC[C@@H]2[C@]1(C)CC[C@H]1[C@H]2CC=C2[C@]1(C)CC[C@@H](C2)O)C | 5280794 |
| IMPHY015042 | Piperitone | CC1=CC(=O)C(CC1)C(C)C | 6987 |
| IMPHY001934 | Obacunone | O=C1C=C[C@]2([C@H](C(O1)(C)C)CC(=O)[C@@]1([C@@H]2CC[C@@]2([C@]31O[C@@H]3C(=O)O[C@H]2c1ccoc1)C)C)C | 119041 |
| IMPHY003289 | Kinetin | c1coc(c1)CNc1ncnc2c1[nH]cn2 | 3830 |
| IMPHY003373 | Limonin | O=C1OC[C@]23[C@H](C1)OC([C@@H]2CC(=O)[C@@]1([C@@H]3CC[C@@]2([C@]31O[C@@H]3C(=O)O[C@H]2c1cocc1)C)C)(C)C | 179651 |
| IMPHY006687 | Nomilin | CC(=O)O[C@H]1CC(=O)OC([C@H]2[C@@]1(C)[C@H]1CC[C@@]3([C@]4([C@@]1(C(=O)C2)C)O[C@@H]4C(=O)O[C@H]3c1ccoc1)C)(C)C | 72320 |
| IMPHY010588 | 3-[(1R,2R,5R,6R,7R,10S,11S,14S)-11-(furan-3-yl)-5-(2-hydroxypropan-2-yl)-2,6,10-trimethyl-3,13-dioxo-12,15-dioxatetracyclo[8.5.0.01,14.02,7]pentadecan-6-yl]-3-hydroxypropanoic acid | OC(=O)CC([C@@]1(C)[C@@H](CC(=O)[C@@]2([C@@H]1CC[C@@]1([C@]32O[C@@H]3C(=O)O[C@H]1c1ccoc1)C)C)C(O)(C)C)O | 101601415 |
| IMPHY013121 | Deacetylnomilin | O=C1CC(O)C2(C(C(O1)(C)C)CC(=O)C1(C2CCC2(C31OC3C(=O)OC2c1ccoc1)C)C)C | 13857953 |
| IMPHY013349 | Limonin 17-beta-D-glucopyranoside | OC[C@H]1O[C@@H](O[C@H]([C@]2(C)CC[C@H]3[C@@]([C@@]42O[C@@H]4C(=O)O)(C)C(=O)C[C@@H]2[C@]43COC(=O)C[C@@H]4OC2(C)C)c2cocc2)[C@@H]([C@H]([C@@H]1O)O)O | 24820753 |
| IMPHY011395 | Scoparone | COc1cc2oc(=O)ccc2cc1OC | 8417 |
| IMPHY011789 | Citral | O=C/C=C(/CCC=C(C)C)C | 638011 |
| IMPHY000005 | Thiamine | OCCc1sc[n+](c1C)Cc1cnc(nc1N)C | 1130 |
| IMPHY000022 | Myrcenol | C=CC(=C)CCCC(O)(C)C | 10975 |
| IMPHY000137 | Auranetin | COc1ccc(cc1)c1oc2c(OC)c(OC)c(cc2c(=O)c1OC)OC | 10643 |
| IMPHY000185 | Acetic acid 3-(3,4,5-trimethoxyphenyl)propyl ester | COc1cc(CCCOC(=O)C)cc(c1OC)OC | 11196443 |
| IMPHY000360 | Byakangelicin | COc1c2ccoc2c(c2c1ccc(=O)o2)OC[C@H](C(O)(C)C)O | 10211 |
| IMPHY000399 | beta-Bisabolene | CC(=CCCC(=C)[C@H]1CCC(=CC1)C)C | 10104370 |
| IMPHY000688 | 7-Methoxycoumarin | COc1ccc2c(c1)oc(=O)cc2 | 10748 |
| IMPHY000795 | Octanal | CCCCCCCC=O | 454 |
| IMPHY000846 | Riboflavin | OC[C@H]([C@H]([C@H](Cn1c2-c(nc3c1cc(C)c(c3)C)c(=O)[nH]c(=O)n2)O)O)O | 493570 |
| IMPHY000879 | Isomeranzin | COc1ccc2c(c1CC(=O)C(C)C)oc(=O)cc2 | 473252 |
| IMPHY000890 | Ichangin | O=C1OC[C@]2([C@H](C1)O)[C@@H](CC(=O)[C@@]1([C@@H]2CC[C@@]2([C@]31O[C@@H]3C(=O)O[C@H]2c1cocc1)C)C)C(O)(C)C | 441801 |
| IMPHY000913 | (S)-7-(((2-O-6-Deoxy-alpha-L-mannopyranosyl)-beta-D-glucopyranosyl)oxy)-2,3-dihydro-5-hydroxy-2-(3-hydroxy-4-methoxyphenyl)-4H-1-benzopyran-4-one | OC[C@H]1O[C@@H](Oc2cc3O[C@@H](CC(=O)c3c(c2)O)c2ccc(c(c2)O)OC)[C@@H]([C@H]([C@@H]1O)O)O[C@@H]1O[C@@H](C)[C@@H]([C@H]([C@H]1O)O)O | 442439 |
| IMPHY000914 | Poncirin | OC[C@H]1O[C@@H](Oc2cc3O[C@@H](CC(=O)c3c(c2)O)c2ccc(cc2)OC)[C@@H]([C@H]([C@@H]1O)O)O[C@@H]1O[C@@H](C)[C@@H]([C@H]([C@H]1O)O)O | 442456 |
| IMPHY001134 | Osthole | COc1ccc2c(c1CC=C(C)C)oc(=O)cc2 | 10228 |
| IMPHY001516 | Decane | CCCCCCCCCC | 15600 |
| IMPHY001552 | Auraptene | C/C(=CCOc1ccc2c(c1)oc(=O)cc2)/CCC=C(C)C | 1550607 |
| IMPHY002286 | 1'-OH-gamma-carotene glucoside/(Carotenoids B-G) | OC[C@H]1O[C@@H](OC(CCC/C(=C/C=C/C(=C/C=C/C(=C/C=C/C=C(/C=C/C=C(/C=C/C2=C(C)CCCC2(C)C)C)C)/C)/C)/C)(C)C)C(C([C@@H]1O)O)O | 16061280 |
| IMPHY002533 | (-)-beta-Curcumene | CC(=CCC[C@H](C1=CCC(=CC1)C)C)C | 14014430 |
| IMPHY002591 | Sinensetin | COc1ccc(cc1OC)c1cc(=O)c2c(o1)cc(c(c2OC)OC)OC | 145659 |
| IMPHY002744 | 2''-O-Xylosylvitexin | OC[C@H]1O[C@H]([C@@H]([C@@H]([C@@H]1O)O)O[C@@H]1OC[C@@H]([C@H]([C@@H]1O)O)O)c1c(O)cc(c2c1oc(cc2=O)c1ccc(cc1)O)O | 101406315 |
| IMPHY003161 | Byakangelicol | COc1c2ccoc2c(c2c1ccc(=O)o2)OC[C@H]1OC1(C)C | 3055167 |
| IMPHY003373 | Limonin | O=C1OC[C@]23[C@H](C1)OC([C@@H]2CC(=O)[C@@]1([C@@H]3CC[C@@]2([C@]31O[C@@H]3C(=O)O[C@H]2c1cocc1)C)C)(C)C | 179651 |
| IMPHY003378 | Meranzin | COc1ccc2c(c1C[C@@H]1OC1(C)C)oc(=O)cc2 | 1803558 |
| IMPHY003441 | 2-Carboxy-D-arabinitol | OC[C@H]([C@H]([C@@](C(=O)O)(CO)O)O)O | 439944 |
| IMPHY003485 | Myrcene | C=CC(=C)CCC=C(C)C | 31253 |
| IMPHY003500 | Citric acid | OC(=O)C(CC(=O)O)(CC(=O)O)O | 311 |
| IMPHY003525 | Nonanal | CCCCCCCCC=O | 31289 |
| IMPHY003536 | Eugenol | C=CCc1ccc(c(c1)OC)O | 3314 |
| IMPHY003537 | Tetradecanal | CCCCCCCCCCCCCC=O | 31291 |
| IMPHY003545 | 4-Isopropylbenzaldehyde | O=Cc1ccc(cc1)C(C)C | 326 |
| IMPHY003552 | 2,3-Dimethylstyrene | C=Cc1cccc(c1C)C | 33936 |
| IMPHY003616 | Bicyclogermacrene | C/C/1=CCC/C(=C/[C@H]2[C@@H](CC1)C2(C)C)/C | 13894537 |
| IMPHY003710 | (-)-Isopulegol | C[C@@H]1CC[C@H]([C@@H](C1)O)C(=C)C | 170833 |
| IMPHY003760 | 2-Nonenal | CCCCCC/C=C/C=O | 5283335 |
| IMPHY003798 | (Z)-alpha-Bisabolene | CC(=CC/C=C(C1CCC(=CC1)C)/C)C | 5352653 |
| IMPHY003977 | (-)-beta-Bourbonene | CC([C@@H]1CC[C@@]2([C@H]1[C@H]1C(=C)CC[C@@H]21)C)C | 62566 |
| IMPHY003982 | gamma-Terpinene | CC1=CCC(=CC1)C(C)C | 7461 |
| IMPHY003992 | Hesperidin | COc1ccc(cc1O)[C@@H]1CC(=O)c2c(O1)cc(cc2O)O[C@@H]1O[C@H](CO[C@@H]2O[C@@H](C)[C@@H]([C@H]([C@H]2O)O)O)[C@H]([C@@H]([C@H]1O)O)O | 10621 |
| IMPHY004077 | Verbenone | CC1=CC(=O)C2CC1C2(C)C | 29025 |
| IMPHY004194 | (1S,2R,5S)-2-isopropyl-5-methylcyclohexyl acetate | C[C@H]1CC[C@@H]([C@H](C1)OC(=O)C)C(C)C | 62335 |
| IMPHY004426 | 2-Dodecenal | CCCCCCCCC/C=C/C=O | 5283361 |
| IMPHY004433 | Chrysoeriol | COc1cc(ccc1O)c1cc(=O)c2c(o1)cc(cc2O)O | 5280666 |
| IMPHY004438 | Geranyl butyrate | CCCC(=O)OC/C=C(/CCC=C(C)C)C | 5355856 |
| IMPHY004619 | Quercetin | Oc1cc(O)c2c(c1)oc(c(c2=O)O)c1ccc(c(c1)O)O | 5280343 |
| IMPHY004660 | Luteolin | Oc1cc(O)c2c(c1)oc(cc2=O)c1ccc(c(c1)O)O | 5280445 |
| IMPHY004661 | Apigenin | Oc1ccc(cc1)c1cc(=O)c2c(o1)cc(cc2O)O | 5280443 |
| IMPHY004681 | Aureusidin | Oc1cc2O/C(=Cc3ccc(c(c3)O)O)/C(=O)c2c(c1)O | 5281220 |
| IMPHY004889 | Cinnamyl alcohol | OC/C=C/c1ccccc1 | 5315892 |
| IMPHY004977 | Syringin | OC/C=C/c1cc(OC)c(c(c1)OC)O[C@@H]1O[C@H](CO)[C@H]([C@@H]([C@H]1O)O)O | 5316860 |
| IMPHY005128 | Oxypeucedanin hydrate | O=c1ccc2c(o1)cc1c(c2OC[C@H](C(O)(C)C)O)cco1 | 17536 |
| IMPHY005428 | Bergapten | COc1c2ccc(=O)oc2cc2c1cco2 | 2355 |
| IMPHY005521 | 1-Hexen-3-OL | CCCC(C=C)O | 20928 |
| IMPHY005587 | Umbelliferone | Oc1ccc2c(c1)oc(=O)cc2 | 5281426 |
| IMPHY006106 | Limocitrin | COc1cc(ccc1O)c1oc2c(OC)c(O)cc(c2c(=O)c1O)O | 5489485 |
| IMPHY006145 | p-Cymene | Cc1ccc(cc1)C(C)C | 7463 |
| IMPHY006177 | Methyl geranate | COC(=O)/C=C(/CCC=C(C)C)C | 5365910 |
| IMPHY006324 | Linalyl propionate | CCC(=O)OC(CCC=C(C)C)(C=C)C | 61098 |
| IMPHY006362 | Ascorbic acid | OC[C@@H]([C@H]1OC(=O)C(=C1O)O)O | 54670067 |
| IMPHY006417 | 2,6-Dimethyl-2,4,6-octatriene | C/C=C(/C=C/C=C(C)C)C | 5368821 |
| IMPHY006550 | Thymol | Cc1ccc(c(c1)O)C(C)C | 6989 |
| IMPHY006551 | Isoscopoletin | COc1cc2oc(=O)ccc2cc1O | 69894 |
| IMPHY006618 | Nobiletin | COc1cc(ccc1OC)c1cc(=O)c2c(o1)c(OC)c(c(c2OC)OC)OC | 72344 |
| IMPHY006696 | Methyleugenol | C=CCc1ccc(c(c1)OC)OC | 7127 |
| IMPHY006700 | Methyl benzoate | COC(=O)c1ccccc1 | 7150 |
| IMPHY006709 | Acetyleugenol | C=CCc1ccc(c(c1)OC)OC(=O)C | 7136 |
| IMPHY006944 | Estragole | COc1ccc(cc1)CC=C | 8815 |
| IMPHY006950 | Tricyclene | CC12C3C1CC(C2(C)C)C3 | 79035 |
| IMPHY006965 | alpha,alpha-Dimethyl-4-methylenecyclohexanemethanolCC(C1CCC(=C)CC1)(O)C | CC(C1CCC(=C)CC1)(O)C | 81722 |
| IMPHY006970 | Decanal | CCCCCCCCCC=O | 8175 |
| IMPHY007065 | Indole-3-acetate | [O-]C(=O)Cc1c[nH]c2c1cccc2 | 801 |
| IMPHY007067 | Linalyl acetate | C=CC(OC(=O)C)(CCC=C(C)C)C | 8294 |
| IMPHY007076 | Undecanal | CCCCCCCCCCC=O | 8186 |
| IMPHY007099 | Decyl acetate | CCCCCCCCCCOC(=O)C | 8167 |
| IMPHY007120 | Seselin | CC1(C)C=Cc2c(O1)ccc1c2oc(=O)cc1 | 68229 |
| IMPHY007128 | Suberosin | COc1cc2oc(=O)ccc2cc1CC=C(C)C | 68486 |
| IMPHY007151 | Pantothenic acid | OCC([C@H](C(=O)NCCC(=O)O)O)(C)C | 6613 |
| IMPHY007197 | Isopimpinellin | COc1c2ccc(=O)oc2c(c2c1cco2)OC | 68079 |
| IMPHY007198 | Isoimperatorin | CC(=CCOc1c2ccoc2cc2c1ccc(=O)o2)C | 68081 |
| IMPHY007204 | Dodecanal | CCCCCCCCCCCC=O | 8194 |
| IMPHY007207 | Tangeretin | COc1ccc(cc1)c1cc(=O)c2c(o1)c(OC)c(c(c2OC)OC)OC | 68077 |
| IMPHY007214 | Octyl acetate | CCCCCCCCOC(=O)C | 8164 |
| IMPHY007231 | 8-[(2S)-2,3-dihydroxy-3-methylbutyl]-7-methoxychromen-2-one | COc1ccc2c(c1C[C@@H](C(O)(C)C)O)oc(=O)cc2 | 821434 |
| IMPHY007302 | Hexyl acetate | CCCCCCOC(=O)C | 8908 |
| IMPHY007329 | Phellopterin | COc1c2ccoc2c(c2c1ccc(=O)o2)OCC=C(C)C | 98608 |
| IMPHY007331 | 6-Methyl-5-hepten-2-one | CC(=O)CCC=C(C)C | 9862 |
| IMPHY007376 | beta-Cubebene | CC([C@@H]1CC[C@H]([C@]23[C@H]1[C@H]2C(=C)CC3)C)C | 93081 |
| IMPHY007421 | Citronellyl acetate | CC(CCC=C(C)C)CCOC(=O)C | 9017 |
| IMPHY007528 | Cadinane | C[C@H]1CC[C@@H]2[C@@H](C1)[C@@H](CC[C@@H]2C)C(C)C | 9548708 |
| IMPHY007620 | 1-Octanol | CCCCCCCCO | 957 |
| IMPHY007840 | Spathulenol | C=C1CC[C@@H]2[C@H]([C@H]3[C@H]1CC[C@]3(C)O)C2(C)C | 92231 |
| IMPHY008252 | Inositol | OC1C(O)C(O)C(C(C1O)O)O | 892 |
| IMPHY008597 | Nonyl acetate | CCCCCCCCCOC(=O)C | 8918 |
| IMPHY008724 | Isorhamnetin | COc1cc(ccc1O)c1oc2cc(O)cc(c2c(=O)c1O)O | 5281654 |
| IMPHY008873 | Luteolin 7-rutinoside | OC1[C@@H](OC([C@H]([C@@H]1O)O)CO[C@@H]1OC(C)[C@@H]([C@@H](C1O)O)O)Oc1cc(O)c2c(c1)oc(cc2=O)c1ccc(c(c1)O)O | 44258082 |
| IMPHY008877 | Quercetin-3,5-diglucoside | OC[C@H]1O[C@@H](Oc2c(oc3c(c2=O)c(cc(c3)O)O[C@@H]2O[C@H](CO)[C@H]([C@H]([C@H]2O)O)O)c2ccc(c(c2)O)O)[C@@H]([C@@H]([C@@H]1O)O)O | 22524457 |
| IMPHY008913 | Imperatorin | CC(=CCOc1c2occc2cc2c1oc(=O)cc2)C | 10212 |
| IMPHY008991 | Benzyl acetate | CC(=O)OCc1ccccc1 | 8785 |
| IMPHY009317 | Limocitrol | COc1cc(ccc1O)c1oc2c(OC)c(O)c(c(c2c(=O)c1O)O)OC | 12311234 |
| IMPHY009360 | Nootkatone | O=C1C[C@@H](C)[C@]2(C(=C1)CC[C@H](C2)C(=C)C)C | 1268142 |
| IMPHY009762 | Heptyl acetate | CCCCCCCOC(=O)C | 8159 |
| IMPHY009815 | gamma-Curcumene | CC(=CCC[C@H](C1=CC=C(CC1)C)C)C | 12304273 |
| IMPHY009826 | 1-(11Z-icosenoyl)-2-(9Z,12Z-octadecadienoyl)-sn-glycero-3-phosphoethanolamine | CCCCCCCC/C=CCCCCCCCCCC(=O)OC[C@@H](OC(=O)CCCCCCC/C=CC/C=CCCCCC)COP(=O)(OCC[NH3+])[O-] | 102515444 |
| IMPHY010049 | Artesin[sesquiterpene] | O=C1O[C@H]2[C@H]([C@@H]1C)CC[C@@]1(C2=C(C)CC[C@H]1O)C | 10422228 |
| IMPHY010072 | Eucalyptol | CC12CCC(CC1)C(O2)(C)C | 2758 |
| IMPHY010080 | beta-Elemene | C=C[C@]1(C)CC[C@H](C[C@H]1C(=C)C)C(=C)C | 6918391 |
| IMPHY010097 | Benzyl benzoate | O=C(c1ccccc1)OCc1ccccc1 | 2345 |
| IMPHY010557 | 5,7-dihydroxy-2-(4-hydroxy-3-methoxyphenyl)-8-[(2S,3R,4R,5S,6R)-3,4,5-trihydroxy-6-(hydroxymethyl)oxan-2-yl]chromen-4-one | OC[C@H]1O[C@H]([C@@H]([C@H]([C@@H]1O)O)O)c1c(O)cc(c2c1oc(cc2=O)c1ccc(c(c1)OC)O)O | 20055255 |
| IMPHY010781 | Limonene oxide, cis-(-)- | CC(=C)[C@H]1CC[C@]2([C@@H](C1)O2)C | 6452061 |
| IMPHY011354 | trans-Sabinene hydrate acetate | CC(=O)O[C@@]1(C)CC[C@@]2(C1C2)C(C)C | 6427504 |
| IMPHY011392 | 3-Carene | CC1=CCC2C(C1)C2(C)C | 26049 |
| IMPHY011395 | Scoparone | COc1cc2oc(=O)ccc2cc1OC | 8417 |
| IMPHY011396 | 4-Carvomenthenol | CC1=CCC(CC1)(O)C(C)C | 11230 |
| IMPHY011408 | Demethylnobiletin | COc1cc(ccc1OC)c1cc(=O)c2c(o1)c(OC)c(c(c2O)OC)OC | 358832 |
| IMPHY011474 | Bergamottin | C/C(=CCOc1c2ccoc2cc2c1ccc(=O)o2)/CCC=C(C)C | 5471349 |
| IMPHY011519 | alpha-Terpinyl acetate | CC(=O)OC(C1CCC(=CC1)C)(C)C | 111037 |
| IMPHY011541 | Scopoletin | COc1cc2ccc(=O)oc2cc1O | 5280460 |
| IMPHY011542 | beta-Eudesmol | C=C1CCC[C@]2([C@H]1C[C@@H](CC2)C(O)(C)C)C | 91457 |
| IMPHY011549 | Citropten | COc1cc(OC)c2c(c1)oc(=O)cc2 | 2775 |
| IMPHY011552 | (1R)-2-methyl-5-propan-2-ylbicyclo[3.1.0]hex-2-ene | CC1=CCC2([C@@H]1C2)C(C)C | 6451618 |
| IMPHY011559 | Gibberellic acid | OC(=O)[C@H]1[C@H]2[C@]3([C@H]4[C@]51CC(=C)[C@](C5)(O)CC4)C=C[C@@H]([C@@]2(C)C(=O)O3)O | 6466 |
| IMPHY011581 | alpha-Selinene | CC1=CCC[C@]2([C@H]1C[C@@H](CC2)C(=C)C)C | 10856614 |
| IMPHY011586 | (S,1Z,6Z)-8-Isopropyl-1-methyl-5-methylenecyclodeca-1,6-diene | C/C/1=C/CCC(=C)/C=C[C@@H](CC1)C(C)C | 91723653 |
| IMPHY011588 | cis-3-Hexen-1-ol | OCC/C=CCC | 5281167 |
| IMPHY011590 | d-Borneol | O[C@@H]1C[C@H]2C([C@@]1(C)CC2)(C)C | 61060 |
| IMPHY011594 | Isorhoifolin | O[C@H]1[C@@H](O[C@@H]([C@H]([C@@H]1O)O)CO[C@@H]1O[C@@H](C)[C@@H]([C@H]([C@H]1O)O)O)Oc1cc(O)c2c(c1)oc(cc2=O)c1ccc(cc1)O | 9851181 |
| IMPHY011599 | Terpinolene | CC1=CCC(=C(C)C)CC1 | 11463 |
| IMPHY011632 | Farnesol | OC/C=C(/CC/C=C(/CCC=C(C)C)C)C | 445070 |
| IMPHY011643 | alpha-Terpinene | CC1=CC=C(CC1)C(C)C | 7462 |
| IMPHY011647 | Geranyl acetate | C/C(=CCOC(=O)C)/CCC=C(C)C | 1549026 |
| IMPHY011648 | Neryl acetate | C/C(=C/COC(=O)C)/CCC=C(C)C | 1549025 |
| IMPHY011652 | : (2Z,4E)-5-[(1S)-1-hydroxy-2,6,6-trimethyl-4-oxocyclohex-2-en-1-yl]-3-methylpenta-2,4-dienoic acid | OC(=O)/C=C(C=C[C@@]1(O)C(=CC(=O)CC1(C)C)C)/C | 5280896 |
| IMPHY011657 | cis-beta-Farnesene | C=CC(=C)CC/C=C(CCC=C(C)C)/C | 5317319 |
| IMPHY011658 | beta-Farnesene | C=CC(=C)CC/C=C(/CCC=C(C)C)C | 5281517 |
| IMPHY011659 | alpha-Muurolene | CC1=C[C@@H]2[C@H](CC1)C(=CC[C@H]2C(C)C)C | 12306047 |
| IMPHY011660 | (+)-alpha-Cadinene | CC1=C[C@@H]2[C@@H](CC1)C(=CC[C@H]2C(C)C)C | 12306048 |
| IMPHY011667 | alpha-Gurjunene | C[C@@H]1CC[C@@H]2[C@H](C3=C(CC[C@H]13)C)C2(C)C | 15560276 |
| IMPHY011668 | beta-Santalene | CC(=CCC[C@]1(C)[C@H]2CC[C@@H](C1=C)C2)C | 10889018 |
| IMPHY011761 | Humulene | C/C/1=CCC(C)(C)/C=C/C/C(=C/CC1)/C | 5281520 |
| IMPHY011763 | Anethole | C/C=C/c1ccc(cc1)OC | 637563 |
| IMPHY011789 | Citral | O=C/C=C(/CCC=C(C)C)C | 638011 |
| IMPHY011790 | Neral | O=C/C=C(CCC=C(C)C)/C | 643779 |
| IMPHY011792 | gamma-Muurolene | CC1=C[C@@H]2[C@H](CC1)C(=C)CC[C@H]2C(C)C | 12313020 |
| IMPHY011793 | (+)-gamma-Cadinene | CC1=C[C@@H]2[C@@H](CC1)C(=C)CC[C@H]2C(C)C | 6432404 |
| IMPHY011817 | alpha-Farnesene | C=C/C(=C/C/C=C(/CCC=C(C)C)C)/C | 5281516 |
| IMPHY011839 | (Z)-gamma-bisabolene | CC(=CCC/C(=C1/CCC(=CC1)C)/C)C | 3033866 |
| IMPHY011882 | Cinnamaldehyde | O=C/C=C/c1ccccc1 | 637511 |
| IMPHY011890 | Elemol | C=C[C@]1(C)CC[C@H](C[C@H]1C(=C)C)C(O)(C)C | 92138 |
| IMPHY011896 | Valencene | CC(=C)[C@@H]1CCC2=CCC[C@H]([C@@]2(C1)C)C | 9855795 |
| IMPHY011901 | Thujone | O=C1C[C@]2([C@@H]([C@H]1C)C2)C(C)C | 261491 |
| IMPHY011902 | beta-Thujone | O=C1C[C@]2([C@@H]([C@@H]1C)C2)C(C)C | 91456 |
| IMPHY011933 | Caffeic acid | OC(=O)/C=C/c1ccc(c(c1)O)O | 689043 |
| IMPHY011957 | (+)-delta-Cadinene | CC1=C[C@@H]2C(=C(C)CC[C@H]2C(C)C)CC1 | 441005 |
| IMPHY011965 | (+)-beta-Phellandrene | CC([C@@H]1CCC(=C)C=C1)C | 442484 |
| IMPHY011973 | (-)-cis-Carveol | CC(=C)[C@@H]1CC=C([C@@H](C1)O)C | 330573 |
| IMPHY011974 | 4-Hydroxycinnamic acid | OC(=O)/C=C/c1ccc(cc1)O | 637542 |
| IMPHY011983 | cis-Cinnamaldehyde | O=C/C=Cc1ccccc1 | 6428995 |
| IMPHY011988 | (-)-trans-Carveol | CC(=C)[C@@H]1CC=C([C@H](C1)O)C | 94221 |
| IMPHY012036 | Camphor | O=C1CC2C(C1(C)CC2)(C)C | 2537 |
| IMPHY012058 | Linalool | C=CC(CCC=C(C)C)(O)C | 6549 |
| IMPHY012061 | alpha-Pinene | CC1=CCC2CC1C2(C)C | 6654 |
| IMPHY012075 | Carvone | CC(=C)C1CC=C(C(=O)C1)C | 7439 |
| IMPHY012086 | Citronellal | O=CCC(CCC=C(C)C)C | 7794 |
| IMPHY012104 | Citronellol | OCCC(CCC=C(C)C)C | 8842 |
| IMPHY012147 | beta-Pinene | C=C1CCC2CC1C2(C)C | 14896 |
| IMPHY012152 | alpha-Fenchol | O[C@H]1[C@@]2(C)CC[C@@H](C1(C)C)C2 | 439711 |
| IMPHY012157 | Perillaldehyde | O=CC1=CCC(CC1)C(=C)C | 16441 |
| IMPHY012160 | alpha-Terpineol | CC1=CCC(CC1)C(O)(C)C | 17100 |
| IMPHY012165 | Sabinene | C=C1CCC2(C1C2)C(C)C | 18818 |
| IMPHY012168 | (1S,2S,6S,7R,8R)-1,3-dimethyl-8-propan-2-yltricyclo[4.4.0.02,7]dec-3-ene | CC([C@H]1CC[C@]2([C@@H]3[C@@H]1[C@H]2C(=CC3)C)C)C | 101607926 |
| IMPHY012175 | D-Limonene | CC1=CC[C@@H](CC1)C(=C)C | 440917 |
| IMPHY012178 | p-Menthan-3-one | CC1CCC(C(=O)C1)C(C)C | 6986 |
| IMPHY012198 | Verbenol | CC1=CC(O)C2CC1C2(C)C | 61126 |
| IMPHY012252 | Eriocitrin | O[C@@H]1[C@@H](O)[C@H](Oc2cc(O)c3c(c2)O[C@@H](CC3=O)c2ccc(c(c2)O)O)O[C@@H]([C@H]1O)CO[C@@H]1O[C@@H](C)[C@@H]([C@H]([C@H]1O)O)O | 83489 |
| IMPHY012261 | alpha-Bergamotene | CC(=CCCC1(C)C2CC=C(C1C2)C)C | 86608 |
| IMPHY012265 | (1r,3s,5r)-6,6-Dimethyl-2-methylidenebicyclo[3.1.1]heptan-3-ol | C=C1[C@@H](O)C[C@H]2C[C@@H]1C2(C)C | 88302 |
| IMPHY012384 | 4-[(3,3-Dimethyloxiran-2-yl)methoxy]furo[3,2-g]chromen-7-one | O=c1ccc2c(o1)cc1c(c2OCC2OC2(C)C)cco1 | 160544 |
| IMPHY012585 | delta-Cadinol | CC1=C[C@@H]2[C@H](CC1)[C@](C)(O)CC[C@H]2C(C)C | 3084311 |
| IMPHY012586 | (-)-alpha-Cadinol | CC1=CC2C(CC1)[C@@](C)(O)CC[C@@H]2C(C)C | 6431302 |
| IMPHY012589 | 3-(1,5-Dimethyl-4-hexenyl)-6-methylene-1-cyclohexene | CC(C1CCC(=C)C=C1)CCC=C(C)C | 519764 |
| IMPHY012654 | Nerol | OC/C=C(CCC=C(C)C)/C | 643820 |
| IMPHY012665 | Levomenol | CC(=CCC[C@@]([C@H]1CCC(=CC1)C)(O)C)C | 442343 |
| IMPHY012667 | Caryophyllene oxide | C=C1CC[C@H]2O[C@@]2(CC[C@@H]2[C@@H]1CC2(C)C)C | 1742210 |
| IMPHY012739 | (Z)-beta-Ocimene | C=C/C(=CCC=C(C)C)/C | 5320250 |
| IMPHY013093 | delta-Elemene | C=C[C@@]1(C)CCC(=C[C@@H]1C(=C)C)C(C)C | 12309449 |
| IMPHY013121 | Deacetylnomilin | O=C1CC(O)C2(C(C(O1)(C)C)CC(=O)C1(C2CCC2(C31OC3C(=O)OC2c1ccoc1)C)C)C | 13857953 |
| IMPHY013575 | Campherenol | CC(=CCCC1(C)C2CCC1(C)[C@@H](C2)O)C | 91747494 |
| IMPHY013836 | Fenchone | O=C1C2(C)CCC(C1(C)C)C2 | 14525 |
| IMPHY013838 | 3,7-Dimethyloct-6-en-3-ol | CCC(CCC=C(C)C)(O)C | 86749 |
| IMPHY014060 | Xanthotoxol geranyl ether | C/C(=CCOc1c2occc2cc2c1oc(=O)cc2)/CCC=C(C)C | 5317564 |
| IMPHY014708 | beta-Selinene | C=C1CCC[C@]2([C@H]1C[C@@H](CC2)C(=C)C)C | 442393 |
| IMPHY014806 | Caswell No. 264AB | CC([C@@H]1CC[C@H]([C@]23[C@H]1[C@H]2C(=CC3)C)C)C | 442359 |
| IMPHY014811 | alpha-Phellandrene | CC1=CCC(C=C1)C(C)C | 7460 |
| IMPHY014830 | Bergamotene | CC(=CCCC1(C)C2CCC(=C)C1C2)C | 521569 |
| IMPHY014831 | beta-Caryophyllene | C/C/1=CCCC(=C)[C@@H]2[C@@H](CC1)C(C2)(C)C | 5281515 |
| IMPHY014835 | (E)-beta-ocimene | C=C/C(=C/CC=C(C)C)/C | 5281553 |
| IMPHY014836 | beta-Sitosterol | CC[C@@H](C(C)C)CC[C@H]([C@H]1CC[C@@H]2[C@]1(C)CC[C@H]1[C@H]2CC=C2[C@]1(C)CC[C@@H](C2)O)C | 222284 |
| IMPHY014838 | Daucosterol | CC[C@@H](C(C)C)CC[C@H]([C@H]1CC[C@@H]2[C@]1(C)CC[C@H]1[C@H]2CC=C2[C@]1(C)CC[C@@H](C2)O[C@@H]1O[C@H](CO)[C@H]([C@@H]([C@H]1O)O)O)C | 5742590 |
| IMPHY014842 | Stigmasterol | CC[C@@H](C(C)C)/C=C/[C@H]([C@H]1CC[C@@H]2[C@]1(C)CC[C@H]1[C@H]2CC=C2[C@]1(C)CC[C@@H](C2)O)C | 5280794 |
| IMPHY014847 | Bornyl acetate | CC(=O)OC1CC2C(C1(C)CC2)(C)C | 6448 |
| IMPHY014852 | Camphene | C=C1C2CCC(C1(C)C)C2 | 6616 |
| IMPHY014863 | cis-alpha-Bergamotene | CC(=CCCC1(C)[C@@H]2CC=C([C@H]1C2)C)C | 91753502 |
| IMPHY014874 | cis-Sabinene hydrate | C[C@@H]1CC[C@@]2(C1C2)C(C)C | 101629835 |
| IMPHY014876 | 7-epi-cis-Sesquisabinene hydrate | CC(=CCCC([C@]12CC[C@@](C2C1)(C)O)C)C | 6428435 |
| IMPHY014877 | (S)-cis-Verbenol | CC1=C[C@H](O)[C@H]2C[C@@H]1C2(C)C | 87839 |
| IMPHY014885 | 1-Isopropyl-4,7-dimethyl-1,3,4,5,6,8a-hexahydro-4a(2H)-naphthalenol | CC1=CC2C(CC1)(O)C(C)CCC2C(C)C | 519857 |
| IMPHY014906 | Cedrelanol | CC1=C[C@@H]2[C@@H](CC1)[C@@](C)(O)CC[C@H]2C(C)C | 160799 |
| IMPHY014923 | Geraniol | OC/C=C(/CCC=C(C)C)C | 637566 |
| IMPHY014988 | Limonene | CC1=CCC(CC1)C(=C)C | 22311 |
| IMPHY014990 | Linoleic acid | CCCCC/C=CC/C=CCCCCCCCC(=O)O | 5280450 |
| IMPHY015003 | Menthol | CC1CCC(C(C1)O)C(C)C | 1254 |
| IMPHY015004 | Menthone | C[C@@H]1CC[C@H](C(=O)C1)C(C)C | 26447 |
| IMPHY015022 | Nerolidol | C=CC(CC/C=C(/CCC=C(C)C)C)(O)C | 5284507 |
| IMPHY015042 | Piperitone | CC1=CC(=O)C(CC1)C(C)C | 6987 |
| IMPHY015047 | : Rutin | Oc1cc(O)c2c(c1)oc(c(c2=O)O[C@@H]1O[C@H](CO[C@@H]2O[C@@H](C)[C@@H]([C@H]([C@H]2O)O)O)[C@H]([C@@H]([C@H]1O)O)O)c1ccc(c(c1)O)O | 5280805 |
| IMPHY015094 | (+)-trans-Limonene oxide | CC(=C)[C@@H]1CC[C@]2([C@@H](C1)O2)C | 449290 |
| IMPHY015095 | 2-Cyclohexen-1-ol, 1-methyl-4-(1-methylethyl)-, trans | CC([C@@H]1CC[C@@](C=C1)(C)O)C | 122484 |
| IMPHY015098 | trans-Verbenol | CC1=C[C@H](O)[C@@H]2C[C@H]1C2(C)C | 89664 |
| IMPHY015123 | alpha-Copaene | CC([C@@H]1CC[C@]2([C@@H]3[C@H]1C2C(=CC3)C)C)C | 70678558 |
| IMPHY015128 | T-Muurolol | CC1=C[C@@H]2[C@H](CC1)[C@@](C)(O)CC[C@H]2C(C)C | 3084331 |
| IMPHY015749 | Isoamyl isovalerate | CC(CCOC(=O)CC(C)C)C | 12613 |
| IMPHY016012 | Allo-Aromadendrene | C[C@@H]1CC[C@H]2[C@@H]1C1C(C1(C)C)CCC2=C | 42608158 |
| IMPHY016014 | Isobornyl acetate | CC(=O)O[C@H]1C[C@@H]2C([C@]1(C)CC2)(C)C | 247573 |
| IMPHY016027 | trans-Sabinene hydrate | CC([C@@]12CC[C@](C2C1)(C)O)C | 12315151 |
| IMPHY016054 | trans-alpha-Bergamotene | CC(=CCC[C@]1(C)[C@H]2CC=C([C@@H]1C2)C)C | 6429302 |
| IMPHY016635 | Epoxyaurapten | C/C(=CCOc1ccc2c(c1)oc(=O)cc2)/CCC1OC1(C)C | 9796891 |
| IMPHY016636 | Epoxybergamottin | C/C(=CCOc1c2ccoc2cc2c1ccc(=O)o2)/CCC1OC1(C)C | 9946625 |
| IMPHY016930 | Cnidicin | CC(=CCOc1c2occc2c(c2c1oc(=O)cc2)OCC=C(C)C)C | 10043694 |
| IMPHY017658 | 9-Hydroxy-4-(3,7-dimethyl-2,6-octadienyloxy)-psoralen | COc1c2occc2c(c2c1oc(=O)cc2)OC/C=C(/CCC=C(C)C)C | 6440422 |
|  |  |  |  |
|  |  |  |  |
|  |  |  |  |
|  |  |  |  |
|  |  |  |  |
| ***15. Plant name: Citrus sinensis*** | | | |
| **IMPPAT Phytochemical identifier:** | **Phytochemical name:** | **SMILES:** | **CID** |
| IMPHY000399 | beta-Bisabolene | CC(=CCCC(=C)[C@H]1CCC(=CC1)C)C | 10104370 |
| IMPHY001144 | Dillapiol | C=CCc1cc2OCOc2c(c1OC)OC | 10231 |
| IMPHY001351 | Elemicin | C=CCc1cc(OC)c(c(c1)OC)OC | 10248 |
| IMPHY003485 | Myrcene | C=CC(=C)CCC=C(C)C | 31253 |
| IMPHY003982 | gamma-Terpinene | CC1=CCC(=CC1)C(C)C | 7461 |
| IMPHY004077 | Verbenone | CC1=CC(=O)C2CC1C2(C)C | 29025 |
| IMPHY006145 | p-Cymene | Cc1ccc(cc1)C(C)C | 7463 |
| IMPHY010072 | Eucalyptol | CC12CCC(CC1)C(O2)(C)C | 2758 |
| IMPHY011392 | 3-Carene | CC1=CCC2C(C1)C2(C)C | 26049 |
| IMPHY011396 | 4-Carvomenthenol | CC1=CCC(CC1)(O)C(C)C | 11230 |
| IMPHY011519 | alpha-Terpinyl acetate | CC(=O)OC(C1CCC(=CC1)C)(C)C | 111037 |
| IMPHY011552 | (1R)-2-methyl-5-propan-2-ylbicyclo[3.1.0]hex-2-ene | CC1=CCC2([C@@H]1C2)C(C)C | 6451618 |
| IMPHY011590 | d-Borneol | O[C@@H]1C[C@H]2C([C@@]1(C)CC2)(C)C | 61060 |
| IMPHY011647 | Geranyl acetate | C/C(=CCOC(=O)C)/CCC=C(C)C | 1549026 |
| IMPHY011648 | Neryl acetate | C/C(=C/COC(=O)C)/CCC=C(C)C | 1549025 |
| IMPHY011789 | Citral | O=C/C=C(/CCC=C(C)C)C | 638011 |
| IMPHY011790 | Neral | O=C/C=C(CCC=C(C)C)/C | 643779 |
| IMPHY011965 | (+)-beta-Phellandrene | CC([C@@H]1CCC(=C)C=C1)C | 442484 |
| IMPHY012036 | Camphor | O=C1CC2C(C1(C)CC2)(C)C | 2537 |
| IMPHY012058 | Linalool | C=CC(CCC=C(C)C)(O)C | 6549 |
| IMPHY012061 | alpha-Pinene | CC1=CCC2CC1C2(C)C | 6654 |
| IMPHY012086 | Citronellal | O=CCC(CCC=C(C)C)C | 7794 |
| IMPHY012147 | beta-Pinene | C=C1CCC2CC1C2(C)C | 14896 |
| IMPHY012160 | alpha-Terpineol | CC1=CCC(CC1)C(O)(C)C | 17100 |
| IMPHY012165 | Sabinene | C=C1CCC2(C1C2)C(C)C | 18818 |
| IMPHY012265 | (1r,3s,5r)-6,6-Dimethyl-2-methylidenebicyclo[3.1.1]heptan-3-ol | C=C1[C@@H](O)C[C@H]2C[C@@H]1C2(C)C | 88302 |
| IMPHY014690 | (-)-Globulol | C[C@@H]1CC[C@@H]2[C@@H]1[C@H]1[C@H](C1(C)C)CC[C@@]2(C)O | 12304985 |
| IMPHY014811 | alpha-Phellandrene | CC1=CCC(C=C1)C(C)C | 7460 |
| IMPHY014831 | beta-Caryophyllene | C/C/1=CCCC(=C)[C@@H]2[C@@H](CC1)C(C2)(C)C | 5281515 |
| IMPHY014835 | (E)-beta-ocimene | C=C/C(=C/CC=C(C)C)/C | 5281553 |
| IMPHY014847 | Bornyl acetate | CC(=O)OC1CC2C(C1(C)CC2)(C)C | 6448 |
| IMPHY014852 | Camphene | C=C1C2CCC(C1(C)C)C2 | 6616 |
| IMPHY014988 | Limonene | CC1=CCC(CC1)C(=C)C | 22311 |
| IMPHY016012 | Allo-Aromadendrene | C[C@@H]1CC[C@H]2[C@@H]1C1C(C1(C)C)CCC2=C | 42608158 |
| IMPHY001555 | Jasmone | CC/C=CCC1=C(C)CCC1=O | 1549018 |
| IMPHY002983 | 1-Hexadecanol | CCCCCCCCCCCCCCCCO | 2682 |
| IMPHY003485 | Myrcene | C=CC(=C)CCC=C(C)C | 31253 |
| IMPHY003495 | 2-Methoxy-4-vinylphenol | COc1cc(C=C)ccc1O | 332 |
| IMPHY003537 | Tetradecanal | CCCCCCCCCCCCCC=O | 31291 |
| IMPHY003616 | Bicyclogermacrene | C/C/1=CCC/C(=C/[C@H]2[C@@H](CC1)C2(C)C)/C | 13894537 |
| IMPHY003661 | 24-Methylenecholesterol | O[C@H]1CC[C@]2(C(=CC[C@@H]3[C@@H]2CC[C@]2([C@H]3CC[C@@H]2[C@@H](CCC(=C)C(C)C)C)C)C1)C | 92113 |
| IMPHY003982 | gamma-Terpinene | CC1=CCC(=CC1)C(C)C | 7461 |
| IMPHY005609 | beta-Sinensal | C=CC(=C)CC/C=C(/CC/C=C(/C=O)C)C | 5281535 |
| IMPHY005833 | 24-Methyldesmosterol | O[C@H]1CC[C@]2(C(=CC[C@@H]3[C@@H]2CC[C@]2([C@H]3CC[C@@H]2[C@@H](CCC(=C(C)C)C)C)C)C1)C | 193567 |
| IMPHY006012 | 3-Cyclohexene-1-carboxaldehyde | O=CC1CCC=CC1 | 7508 |
| IMPHY006279 | 2-Phenylethanol | OCCc1ccccc1 | 6054 |
| IMPHY006941 | Phenylacetonitrile | N#CCc1ccccc1 | 8794 |
| IMPHY006968 | Methyl anthranilate | COC(=O)c1ccccc1N | 8635 |
| IMPHY007067 | Linalyl acetate | C=CC(OC(=O)C)(CCC=C(C)C)C | 8294 |
| IMPHY007539 | Phenylacetaldehyde | O=CCc1ccccc1 | 998 |
| IMPHY009355 | Tetracosane | CCCCCCCCCCCCCCCCCCCCCCCC | 12592 |
| IMPHY009368 | Heptadecane | CCCCCCCCCCCCCCCCC | 12398 |
| IMPHY009369 | Nonadecane | CCCCCCCCCCCCCCCCCCC | 12401 |
| IMPHY009382 | Heneicosane | CCCCCCCCCCCCCCCCCCCCC | 12403 |
| IMPHY009490 | Tricosane | CCCCCCCCCCCCCCCCCCCCCCC | 12534 |
| IMPHY009853 | Naphthalene | c1ccc2c(c1)cccc2 | 931 |
| IMPHY009946 | Benzaldehyde | O=Cc1ccccc1 | 240 |
| IMPHY010080 | beta-Elemene | C=C[C@]1(C)CC[C@H](C[C@H]1C(=C)C)C(=C)C | 6918391 |
| IMPHY011086 | alpha-Sinensal | C=C/C(=C/C/C=C(/CC/C=C(/C=O)C)C)/C | 5281534 |
| IMPHY011392 | 3-Carene | CC1=CCC2C(C1)C2(C)C | 26049 |
| IMPHY011396 | 4-Carvomenthenol | CC1=CCC(CC1)(O)C(C)C | 11230 |
| IMPHY011552 | (1R)-2-methyl-5-propan-2-ylbicyclo[3.1.0]hex-2-ene | CC1=CCC2([C@@H]1C2)C(C)C | 6451618 |
| IMPHY011562 | 2-Hexenal | CCC/C=C/C=O | 5281168 |
| IMPHY011599 | Terpinolene | CC1=CCC(=C(C)C)CC1 | 11463 |
| IMPHY011613 | Fucosterol | C/C=C(C(C)C)/CC[C@H]([C@H]1CC[C@@H]2[C@]1(C)CC[C@H]1[C@H]2CC=C2[C@]1(C)CC[C@@H](C2)O)C | 5281326 |
| IMPHY011632 | Farnesol | OC/C=C(/CC/C=C(/CCC=C(C)C)C)C | 445070 |
| IMPHY011643 | alpha-Terpinene | CC1=CC=C(CC1)C(C)C | 7462 |
| IMPHY011647 | Geranyl acetate | C/C(=CCOC(=O)C)/CCC=C(C)C | 1549026 |
| IMPHY011648 | Neryl acetate | C/C(=C/COC(=O)C)/CCC=C(C)C | 1549025 |
| IMPHY011658 | beta-Farnesene | C=CC(=C)CC/C=C(/CCC=C(C)C)C | 5281517 |
| IMPHY011761 | Humulene | C/C/1=CCC(C)(C)/C=C/C/C(=C/CC1)/C | 5281520 |
| IMPHY011817 | alpha-Farnesene | C=C/C(=C/C/C=C(/CCC=C(C)C)C)/C | 5281516 |
| IMPHY012058 | Linalool | C=CC(CCC=C(C)C)(O)C | 6549 |
| IMPHY012061 | alpha-Pinene | CC1=CCC2CC1C2(C)C | 6654 |
| IMPHY012147 | beta-Pinene | C=C1CCC2CC1C2(C)C | 14896 |
| IMPHY012160 | alpha-Terpineol | CC1=CCC(CC1)C(O)(C)C | 17100 |
| IMPHY012165 | Sabinene | C=C1CCC2(C1C2)C(C)C | 18818 |
| IMPHY012402 | Campesterol | O[C@H]1CC[C@]2(C(=CC[C@@H]3[C@@H]2CC[C@]2([C@H]3CC[C@@H]2[C@@H](CC[C@H](C(C)C)C)C)C)C1)C | 173183 |
| IMPHY012654 | Nerol | OC/C=C(CCC=C(C)C)/C | 643820 |
| IMPHY012667 | Caryophyllene oxide | C=C1CC[C@H]2O[C@@]2(CC[C@@H]2[C@@H]1CC2(C)C)C | 1742210 |
| IMPHY012920 | 2-Furanmethanol, 5-ethenyltetrahydro-alpha,alpha,5-trimethyl-, cis- | C=C[C@@]1(C)CC[C@H](O1)C(O)(C)C | 11116492 |
| IMPHY014438 | 1,6,10-Dodecatrien-3-ol | CC=CCCC=CCCC(C=C)O | 54135459 |
| IMPHY014811 | alpha-Phellandrene | CC1=CCC(C=C1)C(C)C | 7460 |
| IMPHY014831 | beta-Caryophyllene | C/C/1=CCCC(=C)[C@@H]2[C@@H](CC1)C(C2)(C)C | 5281515 |
| IMPHY014835 | (E)-beta-ocimene | C=C/C(=C/CC=C(C)C)/C | 5281553 |
| IMPHY014836 | beta-Sitosterol | CC[C@@H](C(C)C)CC[C@H]([C@H]1CC[C@@H]2[C@]1(C)CC[C@H]1[C@H]2CC=C2[C@]1(C)CC[C@@H](C2)O)C | 222284 |
| IMPHY014852 | Camphene | C=C1C2CCC(C1(C)C)C2 | 6616 |
| IMPHY014923 | Geraniol | OC/C=C(/CCC=C(C)C)C | 637566 |
| IMPHY014988 | Limonene | CC1=CCC(CC1)C(=C)C | 22311 |
| IMPHY014989 | trans-Linalool oxide | C=C[C@]1(C)CC[C@H](O1)C(O)(C)C | 6432254 |
| IMPHY015022 | Nerolidol | C=CC(CC/C=C(/CCC=C(C)C)C)(O)C | 5284507 |
| IMPHY015128 | T-Muurolol | CC1=C[C@@H]2[C@H](CC1)[C@@](C)(O)CC[C@H]2C(C)C | 3084331 |
| IMPHY015160 | 1,3,6-Octatriene | C/C=C/C/C=C/C=C | 5367382 |
| IMPHY015238 | 2,6,10-Dodecatrienal | CC=CCCC=CCCC=CC=O | 76538710 |
| IMPHY015328 | 3-Cyclohexene-1-methanol | OCC1CCC=CC1 | 15512 |
| IMPHY015446 | 7-Tetradecenal | CCCCCC/C=C/CCCCCC=O | 5283367 |
| IMPHY015882 | Pentadecanenitrile | CCCCCCCCCCCCCCC#N | 87568 |
| IMPHY016027 | trans-Sabinene hydrate | CC([C@@]12CC[C@](C2C1)(C)O)C | 12315151 |
| IMPHY016223 | 2-Cyclohexen-1-OL | OC1CCCC=C1 | 13198 |
| IMPHY017377 | 4-Methyl-3-pentenyl | [CH2-]C[C-]=C([CH2+])[CH2+] | 53628256 |
| IMPHY000022 | Myrcenol | C=CC(=C)CCCC(O)(C)C | 10975 |
| IMPHY000042 | 3-Methyl-2-butanone | CC(=O)C(C)C | 11251 |
| IMPHY000171 | Ascaridole | CC(C12CCC(OO1)(C=C2)C)C | 10545 |
| IMPHY000173 | Thujyl alcohol | OC1CC2(C(C1C)C2)C(C)C | 10550 |
| IMPHY000399 | beta-Bisabolene | CC(=CCCC(=C)[C@H]1CCC(=CC1)C)C | 10104370 |
| IMPHY000619 | Perillyl alcohol | OCC1=CCC(CC1)C(=C)C | 10819 |
| IMPHY000795 | Octanal | CCCCCCCC=O | 454 |
| IMPHY000911 | Naringin | OC[C@H]1O[C@@H](Oc2cc(O)c3c(c2)O[C@@H](CC3=O)c2ccc(cc2)O)[C@@H]([C@H]([C@@H]1O)O)O[C@@H]1O[C@@H](C)[C@@H]([C@H]([C@H]1O)O)O | 442428 |
| IMPHY001246 | Carvacrol | CC(c1ccc(c(c1)O)C)C | 10364 |
| IMPHY001680 | 2-(E)-O-feruloyl-D-galactaric acid | COc1cc(/C=C/C(=O)O[C@H]([C@H]([C@H]([C@@H](C(=O)O)O)O)O)C(=O)O)ccc1O | 14104340 |
| IMPHY001817 | p-Menth-3-en-1-ol | CC(C1=CCC(CC1)(C)O)C | 11468 |
| IMPHY001915 | Octadecane | CCCCCCCCCCCCCCCCCC | 11635 |
| IMPHY002029 | Luteoxanthin | C/C(=CC=CC=C(C=CC=C(C1C=C2[C@@](O1)(C)C[C@H](CC2(C)C)O)/C)/C)/C=C/C=C(/C=C/[C@]12O[C@]2(C)C[C@H](CC1(C)C)O)C | 12112747 |
| IMPHY002129 | 2,2-Dimethyl-3-(3-methylenepent-4-enyl)oxirane | C=CC(=C)CCC1OC1(C)C | 122371 |
| IMPHY002281 | (3E,5E,7E,9E,11E,13E,15E,17E,19E)-20-[(4R)-4-hydroxy-2,6,6-trimethylcyclohexen-1-yl]-5,9,14,18-tetramethylicosa-3,5,7,9,11,13,15,17,19-nonaen-2-one | O[C@@H]1CC(=C(C(C1)(C)C)/C=C/C(=C/C=C/C(=C/C=C/C=C(/C=C/C=C(/C=C/C(=O)C)C)C)/C)/C)C | 101306769 |
| IMPHY002591 | Sinensetin | COc1ccc(cc1OC)c1cc(=O)c2c(o1)cc(c(c2OC)OC)OC | 145659 |
| IMPHY002825 | 2-(4-Methylphenyl)propan-2-ol | Cc1ccc(cc1)C(O)(C)C | 14529 |
| IMPHY002915 | Benzyl Alcohol | OCc1ccccc1 | 244 |
| IMPHY002926 | 2,3-Butanediol | CC(C(O)C)O | 262 |
| IMPHY003016 | Lauric acid | CCCCCCCCCCCC(=O)O | 3893 |
| IMPHY003040 | Tridecanal | CCCCCCCCCCCCC=O | 25311 |
| IMPHY003104 | Decanoic acid | CCCCCCCCCC(=O)O | 2969 |
| IMPHY003179 | 3-Octanone | CCCCCC(=O)CC | 246728 |
| IMPHY003460 | Gibberellin A53 | OC(=O)[C@H]1[C@H]2[C@@]([C@H]3[C@]41CC(=C)[C@](C4)(O)CC3)(C)CCC[C@@]2(C)C(=O)O | 440914 |
| IMPHY003485 | Myrcene | C=CC(=C)CCC=C(C)C | 31253 |
| IMPHY003495 | 2-Methoxy-4-vinylphenol | COc1cc(C=C)ccc1O | 332 |
| IMPHY003512 | Ethyl hexanoate | CCCCCC(=O)OCC | 31265 |
| IMPHY003519 | Isoamyl acetate | CC(CCOC(=O)C)C | 31276 |
| IMPHY003525 | Nonanal | CCCCCCCCC=O | 31289 |
| IMPHY003536 | Eugenol | C=CCc1ccc(c(c1)OC)O | 3314 |
| IMPHY003537 | Tetradecanal | CCCCCCCCCCCCCC=O | 31291 |
| IMPHY003545 | 4-Isopropylbenzaldehyde | O=Cc1ccc(cc1)C(C)C | 326 |
| IMPHY003616 | Bicyclogermacrene | C/C/1=CCC/C(=C/[C@H]2[C@@H](CC1)C2(C)C)/C | 13894537 |
| IMPHY003710 | (-)-Isopulegol | C[C@@H]1CC[C@H]([C@@H](C1)O)C(=C)C | 170833 |
| IMPHY003719 | beta-Copaene | CC([C@@H]1CC[C@]2([C@@H]3[C@H]1C2C(=C)CC3)C)C | 57339298 |
| IMPHY003760 | 2-Nonenal | CCCCCC/C=C/C=O | 5283335 |
| IMPHY003915 | 2-Decenal | CCCCCCC/C=C/C=O | 5283345 |
| IMPHY003982 | gamma-Terpinene | CC1=CCC(=CC1)C(C)C | 7461 |
| IMPHY003992 | Hesperidin | COc1ccc(cc1O)[C@@H]1CC(=O)c2c(O1)cc(cc2O)O[C@@H]1O[C@H](CO[C@@H]2O[C@@H](C)[C@@H]([C@H]([C@H]2O)O)O)[C@H]([C@@H]([C@H]1O)O)O | 10621 |
| IMPHY004116 | (1R,2R,5S,8S,9S,10S,11S)-5-hydroxy-11-methyl-6-methylidene-12-oxo-13-oxapentacyclo[9.3.3.15,8.01,10.02,8]octadecane-9-carboxylic acid | OC(=O)[C@H]1[C@H]2[C@]3([C@H]4[C@]51CC(=C)[C@](C5)(O)CC4)CCC[C@]2(C)C(=O)OC3 | 443756 |
| IMPHY004194 | (1S,2R,5S)-2-isopropyl-5-methylcyclohexyl acetate | C[C@H]1CC[C@@H]([C@H](C1)OC(=O)C)C(C)C | 62335 |
| IMPHY004372 | Selina-4(15),7(11)-diene | C=C1CCC[C@]2([C@H]1CC(=C(C)C)CC2)C | 10655819 |
| IMPHY004380 | Phytoene | C/C(=CC=CC=C(CC/C=C(/CC/C=C(/CCC=C(C)C)C)C)/C)/CC/C=C(/CC/C=C(/CCC=C(C)C)C)C | 5280784 |
| IMPHY004532 | 4-Methyl-beta-methylenecyclohex-3-ene-1-ethanol | OCC(=C)C1CCC(=CC1)C | 527143 |
| IMPHY004555 | 1,3,3-Trimethyl-2-oxabicyclo[2.2.2]oct-5-ene | CC12CCC(C=C1)C(O2)(C)C | 523035 |
| IMPHY004603 | Coniferin | OC/C=C/c1ccc(c(c1)OC)O[C@@H]1O[C@H](CO)[C@H]([C@@H]([C@H]1O)O)O | 5280372 |
| IMPHY004607 | Citroxanthin | C/C(=CC=CC=C(C=CC=C(C1C=C2C(O1)(C)CCCC2(C)C)/C)/C)/C=C/C=C(/C=C/C1=C(C)CCCC1(C)C)C | 5281246 |
| IMPHY004629 | Gibberellin A1 | OC(=O)[C@H]1[C@H]2[C@]3([C@H]4[C@]51CC(=C)[C@](C5)(O)CC4)CC[C@@H]([C@@]2(C)C(=O)O3)O | 5280379 |
| IMPHY004645 | beta-Cryptoxanthin | O[C@@H]1CC(=C(C(C1)(C)C)/C=C/C(=C/C=C/C(=C/C=C/C=C(/C=C/C=C(/C=C/C1=C(C)CCCC1(C)C)C)C)/C)/C)C | 5281235 |
| IMPHY004679 | P-Menth-1-en-9-al | O=CC(C1CCC(=CC1)C)C | 520440 |
| IMPHY005526 | 2-Undecenal | CCCCCCCC/C=C/C=O | 5283356 |
| IMPHY005609 | beta-Sinensal | C=CC(=C)CC/C=C(/CC/C=C(/C=O)C)C | 5281535 |
| IMPHY005653 | 2-Octanol | CCCCCCC(O)C | 20083 |
| IMPHY006033 | Ethyl butyrate | CCCC(=O)OCC | 7762 |
| IMPHY006145 | p-Cymene | Cc1ccc(cc1)C(C)C | 7463 |
| IMPHY006177 | Methyl geranate | COC(=O)/C=C(/CCC=C(C)C)C | 5365910 |
| IMPHY006279 | 2-Phenylethanol | OCCc1ccccc1 | 6054 |
| IMPHY006332 | Isosafrole | C/C=C/c1ccc2c(c1)OCO2 | 637796 |
| IMPHY006347 | Hexanal | CCCCCC=O | 6184 |
| IMPHY006362 | Ascorbic acid | OC[C@@H]([C@H]1OC(=O)C(=C1O)O)O | 54670067 |
| IMPHY006404 | Damascenone | C/C=C/C(=O)C1=C(C)C=CCC1(C)C | 5366074 |
| IMPHY006465 | Phytofluene | C/C(=CC=CC=C(C=CC=C(CC/C=C(/CCC=C(C)C)C)/C)/C)/CC/C=C(/CC/C=C(/CCC=C(C)C)C)C | 6436722 |
| IMPHY006485 | beta-Ionone | CC(=O)/C=C/C1=C(C)CCCC1(C)C | 638014 |
| IMPHY006519 | Linalool oxide, pyrane, (Z)- | C=C[C@]1(C)OC(C)(C)CC[C@@H]1O | 6431477 |
| IMPHY006550 | Thymol | Cc1ccc(c(c1)O)C(C)C | 6989 |
| IMPHY006612 | Corosolic acid | C[C@@H]1CC[C@]2([C@@H]([C@H]1C)C1=CC[C@H]3[C@@]([C@@]1(CC2)C)(C)CC[C@@H]1[C@]3(C)C[C@@H](O)[C@@H](C1(C)C)O)C(=O)O | 6918774 |
| IMPHY006618 | Nobiletin | COc1cc(ccc1OC)c1cc(=O)c2c(o1)c(OC)c(c(c2OC)OC)OC | 72344 |
| IMPHY006947 | 1-Decanol | CCCCCCCCCCO | 8174 |
| IMPHY006950 | Tricyclene | CC12C3C1CC(C2(C)C)C3 | 79035 |
| IMPHY006970 | Decanal | CCCCCCCCCC=O | 8175 |
| IMPHY007007 | Ethyl isobutyrate | CCOC(=O)C(C)C | 7342 |
| IMPHY007067 | Linalyl acetate | C=CC(OC(=O)C)(CCC=C(C)C)C | 8294 |
| IMPHY007068 | Undecanoic acid | CCCCCCCCCCC(=O)O | 8180 |
| IMPHY007076 | Undecanal | CCCCCCCCCCC=O | 8186 |
| IMPHY007099 | Decyl acetate | CCCCCCCCCCOC(=O)C | 8167 |
| IMPHY007137 | Methyl 2-(methylamino)benzoate | COC(=O)c1ccccc1NC | 6826 |
| IMPHY007146 | Methyl acetate | COC(=O)C | 6584 |
| IMPHY007171 | 1-Hexanol | CCCCCCO | 8103 |
| IMPHY007186 | Heptanal | CCCCCCC=O | 8130 |
| IMPHY007200 | 2-Octanone | CCCCCCC(=O)C | 8093 |
| IMPHY007204 | Dodecanal | CCCCCCCCCCCC=O | 8194 |
| IMPHY007207 | Tangeretin | COc1ccc(cc1)c1cc(=O)c2c(o1)c(OC)c(c(c2OC)OC)OC | 68077 |
| IMPHY007214 | Octyl acetate | CCCCCCCCOC(=O)C | 8164 |
| IMPHY007219 | Isobutyl acetate | CC(COC(=O)C)C | 8038 |
| IMPHY007221 | Methyl decanoate | CCCCCCCCCC(=O)OC | 8050 |
| IMPHY007276 | Nonan-1-ol | CCCCCCCCCO | 8914 |
| IMPHY007302 | Hexyl acetate | CCCCCCOC(=O)C | 8908 |
| IMPHY007331 | 6-Methyl-5-hepten-2-one | CC(=O)CCC=C(C)C | 9862 |
| IMPHY007376 | beta-Cubebene | CC([C@@H]1CC[C@H]([C@]23[C@H]1[C@H]2C(=C)CC3)C)C | 93081 |
| IMPHY007417 | Ethyl acetate | CCOC(=O)C | 8857 |
| IMPHY007421 | Citronellyl acetate | CC(CCC=C(C)C)CCOC(=O)C | 9017 |
| IMPHY007467 | Hexadecanal | CCCCCCCCCCCCCCCC=O | 984 |
| IMPHY007620 | 1-Octanol | CCCCCCCCO | 957 |
| IMPHY007713 | Butyl butyrate | CCCCOC(=O)CCC | 7983 |
| IMPHY007714 | Butyraldehyde | CCCC=O | 261 |
| IMPHY007840 | Spathulenol | C=C1CC[C@@H]2[C@H]([C@H]3[C@H]1CC[C@]3(C)O)C2(C)C | 92231 |
| IMPHY007843 | Gibberellin A4 | C=C1C[C@@]23C[C@H]1CC[C@H]3[C@@]13[C@H]([C@@H]2C(=O)O)[C@@](C)([C@H](CC1)O)C(=O)O3 | 92109 |
| IMPHY007908 | Dendrolasin | C/C(=CCCc1cocc1)/CCC=C(C)C | 5316534 |
| IMPHY007949 | Limonene dioxide | CC1(OC1)C1CCC2(C(C1)O2)C | 232703 |
| IMPHY008065 | Ethyl dodecanoate | CCCCCCCCCCCC(=O)OCC | 7800 |
| IMPHY008080 | Gibberellin A19 | O=C[C@]12CCC[C@@]([C@H]2[C@@H]([C@@]23[C@H]1CC[C@](C3)(C(=C)C2)O)C(=O)O)(C)C(=O)O | 5460209 |
| IMPHY008084 | Gibberellin A20 | OC(=O)[C@H]1[C@H]2[C@]3([C@H]4[C@]51CC(=C)[C@](C5)(O)CC4)CCC[C@@]2(C)C(=O)O3 | 5280481 |
| IMPHY008150 | 1-Methyl-4-(prop-1-en-2-yl)benzene | Cc1ccc(cc1)C(=C)C | 62385 |
| IMPHY008369 | Methyl isobutyl ketone | CC(CC(=O)C)C | 7909 |
| IMPHY008440 | Isopiperitenone | CC1=CC(=O)C(CC1)C(=C)C | 79036 |
| IMPHY008597 | Nonyl acetate | CCCCCCCCCOC(=O)C | 8918 |
| IMPHY008727 | Gibberellin A24 | O=C[C@]12CCC[C@@]([C@H]2[C@@H]([C@@]23[C@H]1CC[C@H](C3)C(=C)C2)C(=O)O)(C)C(=O)O | 443454 |
| IMPHY008972 | Butyl acetate | CCCCOC(=O)C | 31272 |
| IMPHY009360 | Nootkatone | O=C1C[C@@H](C)[C@]2(C(=C1)CC[C@H](C2)C(=C)C)C | 1268142 |
| IMPHY009368 | Heptadecane | CCCCCCCCCCCCCCCCC | 12398 |
| IMPHY009382 | Heneicosane | CCCCCCCCCCCCCCCCCCCCC | 12403 |
| IMPHY009460 | 2-Decanone | CCCCCCCCC(=O)C | 12741 |
| IMPHY009739 | 5-Isopropylbicyclo[3.1.0]hexan-2-one | O=C1CCC2(C1C2)C(C)C | 92784 |
| IMPHY009743 | beta-Gurjunene | C[C@@H]1CC[C@@H]2[C@H]([C@H]3[C@@H]1CCC3=C)C2(C)C | 6450812 |
| IMPHY009751 | 1-Heptanol | CCCCCCCO | 8129 |
| IMPHY009762 | Heptyl acetate | CCCCCCCOC(=O)C | 8159 |
| IMPHY009852 | Methyl citronellate | COC(=O)CC(CCC=C(C)C)C | 61290 |
| IMPHY009853 | Naphthalene | c1ccc2c(c1)cccc2 | 931 |
| IMPHY009859 | Octadecanal | CCCCCCCCCCCCCCCCCC=O | 12533 |
| IMPHY009872 | p-Mentha-3,8-diene | CC1CCC(=CC1)C(=C)C | 521851 |
| IMPHY009946 | Benzaldehyde | O=Cc1ccccc1 | 240 |
| IMPHY010016 | beta-Zeacarotene | C/C(=CC=CC=C(C=CC=C(C=CC1=C(C)CCCC1(C)C)/C)/C)/C=C/C=C(/CC/C=C(/CCC=C(C)C)C)C | 5280790 |
| IMPHY010072 | Eucalyptol | CC12CCC(CC1)C(O2)(C)C | 2758 |
| IMPHY010080 | beta-Elemene | C=C[C@]1(C)CC[C@H](C[C@H]1C(=C)C)C(=C)C | 6918391 |
| IMPHY010161 | (6S,7aR)-2-[(2E,4E,6E,8E,10E,12E,14E)-15-[(6S,7aR)-6-hydroxy-4,4,7a-trimethyl-2,5,6,7-tetrahydro-1-benzofuran-2-yl]-6,11-dimethylhexadeca-2,4,6,8,10,12,14-heptaen-2-yl]-4,4,7a-trimethyl-2,5,6,7-tetrah | O[C@@H]1C[C@@]2(C)OC(C=C2C(C1)(C)C)/C(=C/C=C/C(=C/C=C/C=C(/C=C/C=C(/C1C=C2[C@@](O1)(C)C[C@H](CC2(C)C)O)C)C)/C)/C | 134781708 |
| IMPHY010179 | (-)-beta-Chamigrene | CC1=CC[C@@]2(CC1)C(=C)CCCC2(C)C | 442353 |
| IMPHY010318 | Geranyl propionate | CCC(=O)OC/C=C(/CCC=C(C)C)C | 5355853 |
| IMPHY010620 | 3,3',4',5,6,7,8-Heptamethoxyflavone | COc1cc(ccc1OC)c1oc2c(OC)c(OC)c(c(c2c(=O)c1OC)OC)OC | 150893 |
| IMPHY010781 | Limonene oxide, cis-(-)- | CC(=C)[C@H]1CC[C@]2([C@@H](C1)O2)C | 6452061 |
| IMPHY010959 | Apocarotenal | O=C/C(=C/C=C/C(=C/C=C/C=C(/C=C/C=C(/C=C/C1=C(C)CCCC1(C)C)C)C)/C)/C | 5478003 |
| IMPHY011004 | p-Mentha-1,3,8-triene | CC1=CC=C(CC1)C(=C)C | 176983 |
| IMPHY011086 | alpha-Sinensal | C=C/C(=C/C/C=C(/CC/C=C(/C=O)C)C)/C | 5281534 |
| IMPHY011202 | Narirutin | Oc1ccc(cc1)[C@@H]1CC(=O)c2c(O1)cc(cc2O)O[C@@H]1O[C@H](CO[C@@H]2O[C@@H](C)[C@@H]([C@H]([C@H]2O)O)O)[C@H]([C@@H]([C@H]1O)O)O | 442431 |
| IMPHY011215 | Tetradecane | CCCCCCCCCCCCCC | 12389 |
| IMPHY011357 | Nerolidyl acetate | C=CC(OC(=O)C)(CC/C=C(/CCC=C(C)C)C)C | 5363426 |
| IMPHY011392 | 3-Carene | CC1=CCC2C(C1)C2(C)C | 26049 |
| IMPHY011396 | 4-Carvomenthenol | CC1=CCC(CC1)(O)C(C)C | 11230 |
| IMPHY011408 | Demethylnobiletin | COc1cc(ccc1OC)c1cc(=O)c2c(o1)c(OC)c(c(c2O)OC)OC | 358832 |
| IMPHY011513 | Isosinensetin | COc1cc(ccc1OC)c1cc(=O)c2c(o1)c(OC)c(cc2OC)OC | 632135 |
| IMPHY011519 | alpha-Terpinyl acetate | CC(=O)OC(C1CCC(=CC1)C)(C)C | 111037 |
| IMPHY011552 | (1R)-2-methyl-5-propan-2-ylbicyclo[3.1.0]hex-2-ene | CC1=CCC2([C@@H]1C2)C(C)C | 6451618 |
| IMPHY011562 | 2-Hexenal | CCC/C=C/C=O | 5281168 |
| IMPHY011579 | Eremophilene | CC(=C)[C@@H]1CCC2=CCC[C@@H]([C@]2(C1)C)C | 12309744 |
| IMPHY011586 | (S,1Z,6Z)-8-Isopropyl-1-methyl-5-methylenecyclodeca-1,6-diene | C/C/1=C/CCC(=C)/C=C[C@@H](CC1)C(C)C | 91723653 |
| IMPHY011588 | cis-3-Hexen-1-ol | OCC/C=CCC | 5281167 |
| IMPHY011590 | d-Borneol | O[C@@H]1C[C@H]2C([C@@]1(C)CC2)(C)C | 61060 |
| IMPHY011599 | Terpinolene | CC1=CCC(=C(C)C)CC1 | 11463 |
| IMPHY011633 | (2Z,6E)-Farnesol | OC/C=C(CC/C=C(/CCC=C(C)C)C)/C | 1549108 |
| IMPHY011643 | alpha-Terpinene | CC1=CC=C(CC1)C(C)C | 7462 |
| IMPHY011647 | Geranyl acetate | C/C(=CCOC(=O)C)/CCC=C(C)C | 1549026 |
| IMPHY011648 | Neryl acetate | C/C(=C/COC(=O)C)/CCC=C(C)C | 1549025 |
| IMPHY011657 | cis-beta-Farnesene | C=CC(=C)CC/C=C(CCC=C(C)C)/C | 5317319 |
| IMPHY011658 | beta-Farnesene | C=CC(=C)CC/C=C(/CCC=C(C)C)C | 5281517 |
| IMPHY011659 | alpha-Muurolene | CC1=C[C@@H]2[C@H](CC1)C(=CC[C@H]2C(C)C)C | 12306047 |
| IMPHY011668 | beta-Santalene | CC(=CCC[C@]1(C)[C@H]2CC[C@@H](C1=C)C2)C | 10889018 |
| IMPHY011761 | Humulene | C/C/1=CCC(C)(C)/C=C/C/C(=C/CC1)/C | 5281520 |
| IMPHY011789 | Citral | O=C/C=C(/CCC=C(C)C)C | 638011 |
| IMPHY011790 | Neral | O=C/C=C(CCC=C(C)C)/C | 643779 |
| IMPHY011794 | (1E,4E,8E)-2,6,6,9-tetramethylcycloundeca-1,4,8-triene | C/C/1=C/CC(C)(C)/C=CC/C(=CCC1)/C | 6508206 |
| IMPHY011817 | alpha-Farnesene | C=C/C(=C/C/C=C(/CCC=C(C)C)C)/C | 5281516 |
| IMPHY011882 | Cinnamaldehyde | O=C/C=C/c1ccccc1 | 637511 |
| IMPHY011890 | Elemol | C=C[C@]1(C)CC[C@H](C[C@H]1C(=C)C)C(O)(C)C | 92138 |
| IMPHY011896 | Valencene | CC(=C)[C@@H]1CCC2=CCC[C@H]([C@@]2(C1)C)C | 9855795 |
| IMPHY011938 | gamma-Eudesmol | CC1=C2C[C@@H](CC[C@]2(CCC1)C)C(O)(C)C | 6432005 |
| IMPHY011957 | (+)-delta-Cadinene | CC1=C[C@@H]2C(=C(C)CC[C@H]2C(C)C)CC1 | 441005 |
| IMPHY011965 | (+)-beta-Phellandrene | CC([C@@H]1CCC(=C)C=C1)C | 442484 |
| IMPHY011973 | (-)-cis-Carveol | CC(=C)[C@@H]1CC=C([C@@H](C1)O)C | 330573 |
| IMPHY011988 | (-)-trans-Carveol | CC(=C)[C@@H]1CC=C([C@H](C1)O)C | 94221 |
| IMPHY012002 | (+)-Dihydrocarvone | CC(=C)[C@@H]1CC[C@H](C(=O)C1)C | 22227 |
| IMPHY012036 | Camphor | O=C1CC2C(C1(C)CC2)(C)C | 2537 |
| IMPHY012058 | Linalool | C=CC(CCC=C(C)C)(O)C | 6549 |
| IMPHY012061 | alpha-Pinene | CC1=CCC2CC1C2(C)C | 6654 |
| IMPHY012075 | Carvone | CC(=C)C1CC=C(C(=O)C1)C | 7439 |
| IMPHY012086 | Citronellal | O=CCC(CCC=C(C)C)C | 7794 |
| IMPHY012097 | Methyl octanoate | CCCCCCCC(=O)OC | 8091 |
| IMPHY012100 | Nonanoic acid | CCCCCCCCC(=O)O | 8158 |
| IMPHY012104 | Citronellol | OCCC(CCC=C(C)C)C | 8842 |
| IMPHY012130 | Dihydrocarveol | CC(=C)C1CCC(C(C1)O)C | 12072 |
| IMPHY012147 | beta-Pinene | C=C1CCC2CC1C2(C)C | 14896 |
| IMPHY012157 | Perillaldehyde | O=CC1=CCC(CC1)C(=C)C | 16441 |
| IMPHY012160 | alpha-Terpineol | CC1=CCC(CC1)C(O)(C)C | 17100 |
| IMPHY012165 | Sabinene | C=C1CCC2(C1C2)C(C)C | 18818 |
| IMPHY012175 | D-Limonene | CC1=CC[C@@H](CC1)C(=C)C | 440917 |
| IMPHY012255 | (+)-trans-Piperitenol | CC1=C[C@@H]([C@H](CC1)C(C)C)O | 85568 |
| IMPHY012516 | Limonoic acid | OC[C@]12[C@H](CC(=O)O)OC([C@@H]1CC(=O)[C@@]1([C@@H]2CC[C@@]([C@]21O[C@@H]2C(=O)O)(C)[C@H](c1cocc1)O)C)(C)C | 439529 |
| IMPHY012586 | (-)-alpha-Cadinol | CC1=CC2C(CC1)[C@@](C)(O)CC[C@@H]2C(C)C | 6431302 |
| IMPHY012589 | 3-(1,5-Dimethyl-4-hexenyl)-6-methylene-1-cyclohexene | CC(C1CCC(=C)C=C1)CCC=C(C)C | 519764 |
| IMPHY012654 | Nerol | OC/C=C(CCC=C(C)C)/C | 643820 |
| IMPHY012665 | Levomenol | CC(=CCC[C@@]([C@H]1CCC(=CC1)C)(O)C)C | 442343 |
| IMPHY012712 | Phytol | OC/C=C(/CCC[C@@H](CCC[C@@H](CCCC(C)C)C)C)C | 5280435 |
| IMPHY012739 | (Z)-beta-Ocimene | C=C/C(=CCC=C(C)C)/C | 5320250 |
| IMPHY012907 | trans-Sesquisabinene hydrate | CC(=CCCC([C@]12CC[C@](C2C1)(C)O)C)C | 6428444 |
| IMPHY012920 | 2-Furanmethanol, 5-ethenyltetrahydro-alpha,alpha,5-trimethyl-, cis- | C=C[C@@]1(C)CC[C@H](O1)C(O)(C)C | 11116492 |
| IMPHY013080 | alpha-Calacorene | CC([C@@H]1CC=C(c2c1cc(C)cc2)C)C | 12302243 |
| IMPHY013093 | delta-Elemene | C=C[C@@]1(C)CCC(=C[C@@H]1C(=C)C)C(C)C | 12309449 |
| IMPHY013133 | (Z)-p-Menth-2-en-1-ol | CC([C@@H]1CC[C@](C=C1)(C)O)C | 13918681 |
| IMPHY013575 | Campherenol | CC(=CCCC1(C)C2CCC1(C)[C@@H](C2)O)C | 91747494 |
| IMPHY013579 | trans-p-Mentha-2,8-dienol | C[C@H]1CC[C@@H](C(=C1)O)C(=C)C | 91753981 |
| IMPHY013694 | 2'-Apo-beta-carotenal | O=C/C=CC(=C/C=CC(=CC=CC(=CC=C/C=C(/C=C/C=C(C=CC1=C(C)CCCC1(C)C)/C)C)C)C)C | 131751675 |
| IMPHY013764 | (-)-Carvomenthone | CC([C@H]1CC[C@@H](C(=O)C1)C)C | 6432474 |
| IMPHY013835 | (+)-Fenchone | O=C1[C@@]2(C)CC[C@@H](C1(C)C)C2 | 1201521 |
| IMPHY014112 | 3,6-Octadienal, 3,7-dimethyl- | O=CC/C(=C/CC=C(C)C)/C | 6428928 |
| IMPHY014690 | (-)-Globulol | C[C@@H]1CC[C@@H]2[C@@H]1[C@H]1[C@H](C1(C)C)CC[C@@]2(C)O | 12304985 |
| IMPHY014708 | beta-Selinene | C=C1CCC[C@]2([C@H]1C[C@@H](CC2)C(=C)C)C | 442393 |
| IMPHY014718 | cis-4-Decenal | CCCCC/C=CCCC=O | 5362620 |
| IMPHY014805 | Cedr-8-ene | CC1=CC[C@@]23C[C@@H]1C(C)(C)[C@@H]2CC[C@H]3C | 6431015 |
| IMPHY014806 | Caswell No. 264AB | CC([C@@H]1CC[C@H]([C@]23[C@H]1[C@H]2C(=CC3)C)C)C | 442359 |
| IMPHY014811 | alpha-Phellandrene | CC1=CCC(C=C1)C(C)C | 7460 |
| IMPHY014817 | Aromadendrene | CC1CCC2C1C1C(C1(C)C)CCC2=C | 91354 |
| IMPHY014831 | beta-Caryophyllene | C/C/1=CCCC(=C)[C@@H]2[C@@H](CC1)C(C2)(C)C | 5281515 |
| IMPHY014835 | (E)-beta-ocimene | C=C/C(=C/CC=C(C)C)/C | 5281553 |
| IMPHY014847 | Bornyl acetate | CC(=O)OC1CC2C(C1(C)CC2)(C)C | 6448 |
| IMPHY014852 | Camphene | C=C1C2CCC(C1(C)C)C2 | 6616 |
| IMPHY014857 | Cedrol | C[C@@H]1CC[C@@H]2[C@@]31CC[C@@]([C@H](C3)C2(C)C)(C)O | 65575 |
| IMPHY014863 | cis-alpha-Bergamotene | CC(=CCCC1(C)[C@@H]2CC=C([C@H]1C2)C)C | 91753502 |
| IMPHY014871 | cis-Nerolidol | C=CC(CC/C=C(CCC=C(C)C)/C)(O)C | 5320128 |
| IMPHY014873 | 2-Cyclohexen-1-ol, 3-methyl-6-(1-methylethyl)-, (1R,6S)-rel- | CC1=C[C@@H]([C@@H](CC1)C(C)C)O | 85567 |
| IMPHY014874 | cis-Sabinene hydrate | C[C@@H]1CC[C@@]2(C1C2)C(C)C | 101629835 |
| IMPHY014907 | 6-Epi-beta-bisabolol | CC(=CCC[C@@H]([C@@]1(O)CCC(=CC1)C)C)C | 12300148 |
| IMPHY014914 | Fenchol | OC1C2(C)CCC(C1(C)C)C2 | 15406 |
| IMPHY014923 | Geraniol | OC/C=C(/CCC=C(C)C)C | 637566 |
| IMPHY014988 | Limonene | CC1=CCC(CC1)C(=C)C | 22311 |
| IMPHY014989 | trans-Linalool oxide | C=C[C@]1(C)CC[C@H](O1)C(O)(C)C | 6432254 |
| IMPHY015004 | Menthone | C[C@@H]1CC[C@H](C(=O)C1)C(C)C | 26447 |
| IMPHY015016 | alpha-Muurolol | CC1=C[C@@H]2[C@H](CC1)[C@](C)(O)CC[C@@H]2C(C)C | 91753440 |
| IMPHY015022 | Nerolidol | C=CC(CC/C=C(/CCC=C(C)C)C)(O)C | 5284507 |
| IMPHY015094 | (+)-trans-Limonene oxide | CC(=C)[C@@H]1CC[C@]2([C@@H](C1)O2)C | 449290 |
| IMPHY015123 | alpha-Copaene | CC([C@@H]1CC[C@]2([C@@H]3[C@H]1C2C(=CC3)C)C)C | 70678558 |
| IMPHY015128 | T-Muurolol | CC1=C[C@@H]2[C@H](CC1)[C@@](C)(O)CC[C@H]2C(C)C | 3084331 |
| IMPHY015162 | 1,4,7,10,13,16-Hexaoxacyclooctadecane | O1CCOCCOCCOCCOCCOCC1 | 28557 |
| IMPHY015163 | 1,4,7,10-Tetraoxacyclodecane | C1COCCOOCCO1 | 19087899 |
| IMPHY015389 | 4-Methylpyrrolidine-2-carboxylic acid | CC1CC(NC1)C(=O)O | 352051 |
| IMPHY015403 | 5-Acetyl-2-methylpyridine | Cc1ccc(cn1)C(=O)C | 95292 |
| IMPHY015660 | Ethyl decanoate | CCCCCCCCCC(=O)OCC | 8048 |
| IMPHY015670 | Ethyl propionate | CCOC(=O)CC | 7749 |
| IMPHY015749 | Isoamyl isovalerate | CC(CCOC(=O)CC(C)C)C | 12613 |
| IMPHY015889 | Perillyl acetate | CC(=O)OCC1=CCC(CC1)C(=C)C | 61780 |
| IMPHY016027 | trans-Sabinene hydrate | CC([C@@]12CC[C@](C2C1)(C)O)C | 12315151 |
| IMPHY016053 | Viridiflorol | C[C@@H]1CC[C@H]2[C@@H]1[C@H]1[C@H](C1(C)C)CC[C@]2(C)O | 11996452 |
| IMPHY016054 | trans-alpha-Bergamotene | CC(=CCC[C@]1(C)[C@H]2CC=C([C@@H]1C2)C)C | 6429302 |
| IMPHY016244 | 2-Isononenal | O=C/C=C/CCCC(C)C | 87291600 |
| IMPHY016748 | Methyl isobutyrate | COC(=O)C(C)C | 11039 |
| IMPHY016781 | Bicyclo[2.2.1]heptan-1-ol | OC12CCC(C2)CC1 | 524266 |
| IMPHY017283 | 1,4,7,10,13-Pentaoxacyclopentadecane | O1CCOCCOCCOCCOCC1 | 36336 |
| IMPHY017676 | 2'-Methoxyacetophenone | COc1ccccc1C(=O)C | 68481 |
| IMPHY017929 | Dehydrocarveol | CC(=C)C1CC=C(C(=C1)O)C | 45082895 |
| IMPHY017948 | 3,9-Epoxy-p-menta-1,8(10)-diene | C=C1CCC2C(C1)OCC2=C | 584863 |
| IMPHY000399 | beta-Bisabolene | CC(=CCCC(=C)[C@H]1CCC(=CC1)C)C | 10104370 |
| IMPHY000402 | 1,4-Cineole | CC(C12CCC(O2)(CC1)C)C | 10106 |
| IMPHY000545 | O-Cymene | CC(c1ccccc1C)C | 10703 |
| IMPHY000795 | Octanal | CCCCCCCC=O | 454 |
| IMPHY001246 | Carvacrol | CC(c1ccc(c(c1)O)C)C | 10364 |
| IMPHY001555 | Jasmone | CC/C=CCC1=C(C)CCC1=O | 1549018 |
| IMPHY001856 | Stachydrine | [O-]C(=O)[C@@H]1CCC[N+]1(C)C | 115244 |
| IMPHY003050 | Methyl salicylate | COC(=O)c1ccccc1O | 4133 |
| IMPHY003485 | Myrcene | C=CC(=C)CCC=C(C)C | 31253 |
| IMPHY003495 | 2-Methoxy-4-vinylphenol | COc1cc(C=C)ccc1O | 332 |
| IMPHY003525 | Nonanal | CCCCCCCCC=O | 31289 |
| IMPHY003537 | Tetradecanal | CCCCCCCCCCCCCC=O | 31291 |
| IMPHY003616 | Bicyclogermacrene | C/C/1=CCC/C(=C/[C@H]2[C@@H](CC1)C2(C)C)/C | 13894537 |
| IMPHY003695 | (-)-Germacrene A | C/C/1=CCC/C(=C/C[C@H](CC1)C(=C)C)/C | 9548706 |
| IMPHY003982 | gamma-Terpinene | CC1=CCC(=CC1)C(C)C | 7461 |
| IMPHY003992 | Hesperidin | COc1ccc(cc1O)[C@@H]1CC(=O)c2c(O1)cc(cc2O)O[C@@H]1O[C@H](CO[C@@H]2O[C@@H](C)[C@@H]([C@H]([C@H]2O)O)O)[C@H]([C@@H]([C@H]1O)O)O | 10621 |
| IMPHY004077 | Verbenone | CC1=CC(=O)C2CC1C2(C)C | 29025 |
| IMPHY004151 | Geranyl formate | O=COC/C=C(/CCC=C(C)C)C | 5282109 |
| IMPHY004889 | Cinnamyl alcohol | OC/C=C/c1ccccc1 | 5315892 |
| IMPHY005345 | 1-Octen-3-OL | CCCCCC(C=C)O | 18827 |
| IMPHY005541 | 1,4,4-Trimethyl-8-methylene-1,5-cycloundecadiene | C=C1CCC/C(=CCC(/C=CC1)(C)C)/C | 21159064 |
| IMPHY005569 | alpha-Ionone | CC(=O)/C=C/C1C(=CCCC1(C)C)C | 5282108 |
| IMPHY005609 | beta-Sinensal | C=CC(=C)CC/C=C(/CC/C=C(/C=O)C)C | 5281535 |
| IMPHY006007 | Citronellyl formate | O=COCCC(CCC=C(C)C)C | 7778 |
| IMPHY006145 | p-Cymene | Cc1ccc(cc1)C(C)C | 7463 |
| IMPHY006177 | Methyl geranate | COC(=O)/C=C(/CCC=C(C)C)C | 5365910 |
| IMPHY006279 | 2-Phenylethanol | OCCc1ccccc1 | 6054 |
| IMPHY006347 | Hexanal | CCCCCC=O | 6184 |
| IMPHY006414 | Geranyl tiglate | C/C=C(/C(=O)OC/C=C(/CCC=C(C)C)C)C | 5367785 |
| IMPHY006550 | Thymol | Cc1ccc(c(c1)O)C(C)C | 6989 |
| IMPHY006618 | Nobiletin | COc1cc(ccc1OC)c1cc(=O)c2c(o1)c(OC)c(c(c2OC)OC)OC | 72344 |
| IMPHY006941 | Phenylacetonitrile | N#CCc1ccccc1 | 8794 |
| IMPHY006947 | 1-Decanol | CCCCCCCCCCO | 8174 |
| IMPHY006948 | beta-Terpineol | CC(=C)C1CCC(CC1)(C)O | 8748 |
| IMPHY006968 | Methyl anthranilate | COC(=O)c1ccccc1N | 8635 |
| IMPHY006970 | Decanal | CCCCCCCCCC=O | 8175 |
| IMPHY007067 | Linalyl acetate | C=CC(OC(=O)C)(CCC=C(C)C)C | 8294 |
| IMPHY007076 | Undecanal | CCCCCCCCCCC=O | 8186 |
| IMPHY007171 | 1-Hexanol | CCCCCCO | 8103 |
| IMPHY007204 | Dodecanal | CCCCCCCCCCCC=O | 8194 |
| IMPHY007276 | Nonan-1-ol | CCCCCCCCCO | 8914 |
| IMPHY007331 | 6-Methyl-5-hepten-2-one | CC(=O)CCC=C(C)C | 9862 |
| IMPHY007421 | Citronellyl acetate | CC(CCC=C(C)C)CCOC(=O)C | 9017 |
| IMPHY007539 | Phenylacetaldehyde | O=CCc1ccccc1 | 998 |
| IMPHY007620 | 1-Octanol | CCCCCCCCO | 957 |
| IMPHY007840 | Spathulenol | C=C1CC[C@@H]2[C@H]([C@H]3[C@H]1CC[C@]3(C)O)C2(C)C | 92231 |
| IMPHY007908 | Dendrolasin | C/C(=CCCc1cocc1)/CCC=C(C)C | 5316534 |
| IMPHY008150 | 1-Methyl-4-(prop-1-en-2-yl)benzene | Cc1ccc(cc1)C(=C)C | 62385 |
| IMPHY008991 | Benzyl acetate | CC(=O)OCc1ccccc1 | 8785 |
| IMPHY009355 | Tetracosane | CCCCCCCCCCCCCCCCCCCCCCCC | 12592 |
| IMPHY009368 | Heptadecane | CCCCCCCCCCCCCCCCC | 12398 |
| IMPHY009369 | Nonadecane | CCCCCCCCCCCCCCCCCCC | 12401 |
| IMPHY009382 | Heneicosane | CCCCCCCCCCCCCCCCCCCCC | 12403 |
| IMPHY009835 | Linalyl formate | O=COC(CCC=C(C)C)(C=C)C | 61040 |
| IMPHY009946 | Benzaldehyde | O=Cc1ccccc1 | 240 |
| IMPHY010072 | Eucalyptol | CC12CCC(CC1)C(O2)(C)C | 2758 |
| IMPHY010080 | beta-Elemene | C=C[C@]1(C)CC[C@H](C[C@H]1C(=C)C)C(=C)C | 6918391 |
| IMPHY011086 | alpha-Sinensal | C=C/C(=C/C/C=C(/CC/C=C(/C=O)C)C)/C | 5281534 |
| IMPHY011392 | 3-Carene | CC1=CCC2C(C1)C2(C)C | 26049 |
| IMPHY011396 | 4-Carvomenthenol | CC1=CCC(CC1)(O)C(C)C | 11230 |
| IMPHY009490 | Tricosane | CCCCCCCCCCCCCCCCCCCCCCC | 12534 |
| IMPHY009751 | 1-Heptanol | CCCCCCCO | 8129 |
| IMPHY009761 | Citronellyl tiglate | C/C=C(/C(=O)OCCC(CCC=C(C)C)C)C | 6386037 |
| IMPHY011519 | alpha-Terpinyl acetate | CC(=O)OC(C1CCC(=CC1)C)(C)C | 111037 |
| IMPHY011552 | (1R)-2-methyl-5-propan-2-ylbicyclo[3.1.0]hex-2-ene | CC1=CCC2([C@@H]1C2)C(C)C | 6451618 |
| IMPHY011562 | 2-Hexenal | CCC/C=C/C=O | 5281168 |
| IMPHY011568 | alpha-Fenchyl acetate | CC(=O)O[C@@H]1[C@]2(C)CC[C@H](C1(C)C)C2 | 7530950 |
| IMPHY011586 | (S,1Z,6Z)-8-Isopropyl-1-methyl-5-methylenecyclodeca-1,6-diene | C/C/1=C/CCC(=C)/C=C[C@@H](CC1)C(C)C | 91723653 |
| IMPHY011588 | cis-3-Hexen-1-ol | OCC/C=CCC | 5281167 |
| IMPHY011590 | d-Borneol | O[C@@H]1C[C@H]2C([C@@]1(C)CC2)(C)C | 61060 |
| IMPHY011599 | Terpinolene | CC1=CCC(=C(C)C)CC1 | 11463 |
| IMPHY011632 | Farnesol | OC/C=C(/CC/C=C(/CCC=C(C)C)C)C | 445070 |
| IMPHY011643 | alpha-Terpinene | CC1=CC=C(CC1)C(C)C | 7462 |
| IMPHY011647 | Geranyl acetate | C/C(=CCOC(=O)C)/CCC=C(C)C | 1549026 |
| IMPHY011648 | Neryl acetate | C/C(=C/COC(=O)C)/CCC=C(C)C | 1549025 |
| IMPHY011658 | beta-Farnesene | C=CC(=C)CC/C=C(/CCC=C(C)C)C | 5281517 |
| IMPHY011761 | Humulene | C/C/1=CCC(C)(C)/C=C/C/C(=C/CC1)/C | 5281520 |
| IMPHY011777 | Farnesyl acetate | C/C(=CCC/C(=C/COC(=O)C)/C)/CCC=C(C)C | 638500 |
| IMPHY011789 | Citral | O=C/C=C(/CCC=C(C)C)C | 638011 |
| IMPHY011790 | Neral | O=C/C=C(CCC=C(C)C)/C | 643779 |
| IMPHY011817 | alpha-Farnesene | C=C/C(=C/C/C=C(/CCC=C(C)C)C)/C | 5281516 |
| IMPHY011890 | Elemol | C=C[C@]1(C)CC[C@H](C[C@H]1C(=C)C)C(O)(C)C | 92138 |
| IMPHY011896 | Valencene | CC(=C)[C@@H]1CCC2=CCC[C@H]([C@@]2(C1)C)C | 9855795 |
| IMPHY011939 | 10-epi-gamma-Eudesmol | CC1=C2C[C@@H](CC[C@@]2(CCC1)C)C(O)(C)C | 6430754 |
| IMPHY011957 | (+)-delta-Cadinene | CC1=C[C@@H]2C(=C(C)CC[C@H]2C(C)C)CC1 | 441005 |
| IMPHY011965 | (+)-beta-Phellandrene | CC([C@@H]1CCC(=C)C=C1)C | 442484 |
| IMPHY012036 | Camphor | O=C1CC2C(C1(C)CC2)(C)C | 2537 |
| IMPHY012058 | Linalool | C=CC(CCC=C(C)C)(O)C | 6549 |
| IMPHY012061 | alpha-Pinene | CC1=CCC2CC1C2(C)C | 6654 |
| IMPHY012075 | Carvone | CC(=C)C1CC=C(C(=O)C1)C | 7439 |
| IMPHY012086 | Citronellal | O=CCC(CCC=C(C)C)C | 7794 |
| IMPHY012104 | Citronellol | OCCC(CCC=C(C)C)C | 8842 |
| IMPHY012130 | Dihydrocarveol | CC(=C)C1CCC(C(C1)O)C | 12072 |
| IMPHY012147 | beta-Pinene | C=C1CCC2CC1C2(C)C | 14896 |
| IMPHY012157 | Perillaldehyde | O=CC1=CCC(CC1)C(=C)C | 16441 |
| IMPHY012160 | alpha-Terpineol | CC1=CCC(CC1)C(O)(C)C | 17100 |
| IMPHY012165 | Sabinene | C=C1CCC2(C1C2)C(C)C | 18818 |
| IMPHY012179 | (2S,4R)-4-methyl-2-(2-methylprop-1-en-1-yl)tetrahydro-2H-pyran | C[C@@H]1CCO[C@@H](C1)C=C(C)C | 1712087 |
| IMPHY012261 | alpha-Bergamotene | CC(=CCCC1(C)C2CC=C(C1C2)C)C | 86608 |
| IMPHY012654 | Nerol | OC/C=C(CCC=C(C)C)/C | 643820 |
| IMPHY012665 | Levomenol | CC(=CCC[C@@]([C@H]1CCC(=CC1)C)(O)C)C | 442343 |
| IMPHY012667 | Caryophyllene oxide | C=C1CC[C@H]2O[C@@]2(CC[C@@H]2[C@@H]1CC2(C)C)C | 1742210 |
| IMPHY012712 | Phytol | OC/C=C(/CCC[C@@H](CCC[C@@H](CCCC(C)C)C)C)C | 5280435 |
| IMPHY012739 | (Z)-beta-Ocimene | C=C/C(=CCC=C(C)C)/C | [CID:5320250](https://pubchem.ncbi.nlm.nih.gov/compound/5320250) |
| IMPHY012920 | 2-Furanmethanol, 5-ethenyltetrahydro-alpha,alpha,5-trimethyl-, cis- | C=C[C@@]1(C)CC[C@H](O1)C(O)(C)C | [CID:11116492](https://pubchem.ncbi.nlm.nih.gov/compound/11116492) |
| IMPHY013133 | (Z)-p-Menth-2-en-1-ol | CC([C@@H]1CC[C@](C=C1)(C)O)C | [CID:13918681](cid:13918681) |
| IMPHY014718 | cis-4-Decenal | CCCCC/C=CCCC=O | [CID:5362620](https://pubchem.ncbi.nlm.nih.gov/compound/5362620) |
| IMPHY014806 | Caswell No. 264AB | CC([C@@H]1CC[C@H]([C@]23[C@H]1[C@H]2C(=CC3)C)C)C | [CID:442359](https://pubchem.ncbi.nlm.nih.gov/compound/442359) |
| IMPHY014811 | alpha-Phellandrene | CC1=CCC(C=C1)C(C)C | [CID:7460](https://pubchem.ncbi.nlm.nih.gov/compound/7460) |
| IMPHY014831 | beta-Caryophyllene | C/C/1=CCCC(=C)[C@@H]2[C@@H](CC1)C(C2)(C)C | [CID:5281515](https://pubchem.ncbi.nlm.nih.gov/compound/5281515) |
| IMPHY014835 | (E)-beta-ocimene | C=C/C(=C/CC=C(C)C)/C | [CID:5281553](https://pubchem.ncbi.nlm.nih.gov/compound/5281553) |
| IMPHY014847 | Bornyl acetate | CC(=O)OC1CC2C(C1(C)CC2)(C)C | [CID:6448](https://pubchem.ncbi.nlm.nih.gov/compound/6448) |
| IMPHY014852 | Camphene | C=C1C2CCC(C1(C)C)C2 | [CID:6616](https://pubchem.ncbi.nlm.nih.gov/compound/6616) |
| IMPHY014871 | cis-Nerolidol | C=CC(CC/C=C(CCC=C(C)C)/C)(O)C | [CID:5320128](https://pubchem.ncbi.nlm.nih.gov/compound/5320128) |
| IMPHY014873 | 2-Cyclohexen-1-ol, 3-methyl-6-(1-methylethyl)-, (1R,6S)-rel- | CC1=C[C@@H]([C@@H](CC1)C(C)C)O | [CID:85567](https://pubchem.ncbi.nlm.nih.gov/compound/85567) |
| IMPHY014874 | cis-Sabinene hydrate | C[C@@H]1CC[C@@]2(C1C2)C(C)C | [CID:101629835](cid:101629835) |
| IMPHY014923 | Geraniol | OC/C=C(/CCC=C(C)C)C | [CID:637566](https://pubchem.ncbi.nlm.nih.gov/compound/637566) |
| IMPHY014988 | Limonene | CC1=CCC(CC1)C(=C)C | [CID:22311](https://pubchem.ncbi.nlm.nih.gov/compound/22311) |
| IMPHY014989 | trans-Linalool oxide | C=C[C@]1(C)CC[C@H](O1)C(O)(C)C | [CID:6432254](https://pubchem.ncbi.nlm.nih.gov/compound/6432254) |
| IMPHY015003 | Menthol | CC1CCC(C(C1)O)C(C)C | [CID:1254](https://pubchem.ncbi.nlm.nih.gov/compound/1254) |
| IMPHY015016 | alpha-Muurolol | CC1=C[C@@H]2[C@H](CC1)[C@](C)(O)CC[C@@H]2C(C)C | [CID:91753440](https://pubchem.ncbi.nlm.nih.gov/compound/91753440) |
| IMPHY015022 | Nerolidol | C=CC(CC/C=C(/CCC=C(C)C)C)(O)C | [CID:5284507](https://pubchem.ncbi.nlm.nih.gov/compound/5284507) |
| IMPHY015095 | 2-Cyclohexen-1-ol, 1-methyl-4-(1-methylethyl)-, trans- | CC([C@@H]1CC[C@@](C=C1)(C)O)C | [CID:122484](https://pubchem.ncbi.nlm.nih.gov/compound/122484) |
| IMPHY015123 | alpha-Copaene | CC([C@@H]1CC[C@]2([C@@H]3[C@H]1C2C(=CC3)C)C)C | [CID:70678558](https://pubchem.ncbi.nlm.nih.gov/compound/70678558) |
| IMPHY015128 | T-Muurolol | CC1=C[C@@H]2[C@H](CC1)[C@@](C)(O)CC[C@H]2C(C)C | [CID:3084331](https://pubchem.ncbi.nlm.nih.gov/compound/3084331) |
| IMPHY015531 | Butyl propionate | CCCCOC(=O)CC | [CID:11529](https://pubchem.ncbi.nlm.nih.gov/compound/11529) |
| IMPHY016012 | Allo-Aromadendrene | C[C@@H]1CC[C@H]2[C@@H]1C1C(C1(C)C)CCC2=C | [CID:42608158](https://pubchem.ncbi.nlm.nih.gov/compound/42608158) |
| IMPHY016027 | trans-Sabinene hydrate | CC([C@@]12CC[C@](C2C1)(C)O)C | [CID:12315151](https://pubchem.ncbi.nlm.nih.gov/compound/12315151) |
| IMPHY016064 | (3E,7E)-4,8,12-trimethyltrideca-1,3,7,11-tetraene | C=C/C=C(/CC/C=C(/CCC=C(C)C)C)C | [CID:6443227](https://pubchem.ncbi.nlm.nih.gov/compound/6443227) |
| IMPHY016374 | 3-Methylcoumarin | O=c1oc2ccccc2cc1C | [CID:17130](https://pubchem.ncbi.nlm.nih.gov/compound/17130) |
| IMPHY016562 | Cinnamyl formate | O=COC/C=C/c1ccccc1 | [CID:5354883](https://pubchem.ncbi.nlm.nih.gov/compound/5354883) |
| IMPHY006654 | Xanthyletin | CC1(C)C=Cc2c(O1)cc1c(c2)ccc(=O)o1 | [CID:65188](https://pubchem.ncbi.nlm.nih.gov/compound/65188) |
| IMPHY014836 | beta-Sitosterol | CC[C@@H](C(C)C)CC[C@H]([C@H]1CC[C@@H]2[C@]1(C)CC[C@H]1[C@H]2CC=C2[C@]1(C)CC[C@@H](C2)O)C | [CID:222284](https://pubchem.ncbi.nlm.nih.gov/compound/222284) |
| IMPHY014842 | Stigmasterol | CC[C@@H](C(C)C)/C=C/[C@H]([C@H]1CC[C@@H]2[C@]1(C)CC[C@H]1[C@H]2CC=C2[C@]1(C)CC[C@@H](C2)O)C | [CID:5280794](https://pubchem.ncbi.nlm.nih.gov/compound/5280794) |
| IMPHY000060 | Myristic acid | CCCCCCCCCCCCCC(=O)O | [CID:11005](https://pubchem.ncbi.nlm.nih.gov/compound/11005) |
| IMPHY000890 | Ichangin | O=C1OC[C@]2([C@H](C1)O)[C@@H](CC(=O)[C@@]1([C@@H]2CC[C@@]2([C@]31O[C@@H]3C(=O)O[C@H]2c1cocc1)C)C)C(O)(C)C | [CID:441801](https://pubchem.ncbi.nlm.nih.gov/compound/441801) |
| IMPHY001934 | Obacunone | O=C1C=C[C@]2([C@H](C(O1)(C)C)CC(=O)[C@@]1([C@@H]2CC[C@@]2([C@]31O[C@@H]3C(=O)O[C@H]2c1ccoc1)C)C)C | [CID:119041](https://pubchem.ncbi.nlm.nih.gov/compound/119041) |
| IMPHY003373 | Limonin | O=C1OC[C@]23[C@H](C1)OC([C@@H]2CC(=O)[C@@]1([C@@H]3CC[C@@]2([C@]31O[C@@H]3C(=O)O[C@H]2c1cocc1)C)C)(C)C | [CID:179651](https://pubchem.ncbi.nlm.nih.gov/compound/179651) |
| IMPHY003982 | gamma-Terpinene | CC1=CCC(=CC1)C(C)C | [CID:7461](https://pubchem.ncbi.nlm.nih.gov/compound/7461) |
| IMPHY004631 | Stearic acid | CCCCCCCCCCCCCCCCCC(=O)O | [CID:5281](https://pubchem.ncbi.nlm.nih.gov/compound/5281) |
| IMPHY005562 | Hexadecenoic acid | CCCCCCCCCCCCC/C=C/C(=O)O | [CID:5282743](https://pubchem.ncbi.nlm.nih.gov/compound/5282743) |
| IMPHY006145 | p-Cymene | Cc1ccc(cc1)C(C)C | [CID:7463](https://pubchem.ncbi.nlm.nih.gov/compound/7463) |
| IMPHY007327 | Palmitic acid | CCCCCCCCCCCCCCCC(=O)O | [CID:985](https://pubchem.ncbi.nlm.nih.gov/compound/985) |
| IMPHY011394 | Arachidic acid | CCCCCCCCCCCCCCCCCCCC(=O)O | [CID:10467](https://pubchem.ncbi.nlm.nih.gov/compound/10467) |
| IMPHY011797 | Oleic acid | CCCCCCCC/C=CCCCCCCCC(=O)O | [CID:445639](https://pubchem.ncbi.nlm.nih.gov/compound/445639) |
| IMPHY012723 | Linolenic acid | CC/C=CC/C=CC/C=CCCCCCCCC(=O)O | [CID:5280934](https://pubchem.ncbi.nlm.nih.gov/compound/5280934) |
| IMPHY014990 | Linoleic acid | CCCCC/C=CC/C=CCCCCCCCC(=O)O | [CID:5280450](https://pubchem.ncbi.nlm.nih.gov/compound/5280450) |
| IMPHY000022 | Myrcenol | C=CC(=C)CCCC(O)(C)C | [CID:10975](https://pubchem.ncbi.nlm.nih.gov/compound/10975) |
| IMPHY000037 | 3-Methyl-2-buten-1-OL | OCC=C(C)C | [CID:11173](https://pubchem.ncbi.nlm.nih.gov/compound/11173) |
| IMPHY000060 | Myristic acid | CCCCCCCCCCCCCC(=O)O | [CID:11005](https://pubchem.ncbi.nlm.nih.gov/compound/11005) |
| IMPHY000270 | Citrusin II | O=C1CNC(=O)[C@@H](NC(=O)[C@H](Cc2ccccc2)NC(=O)[C@H]2N(C(=O)[C@@H](NC(=O)[C@H]3N(C(=O)CN1)CCC3)C)CCC2)Cc1c[nH]c2c1cccc2 | [CID:102082877](https://pubchem.ncbi.nlm.nih.gov/compound/102082877) |
| IMPHY000360 | Byakangelicin | COc1c2ccoc2c(c2c1ccc(=O)o2)OC[C@H](C(O)(C)C)O | [CID:10211](https://pubchem.ncbi.nlm.nih.gov/compound/10211) |
| IMPHY000399 | beta-Bisabolene | CC(=CCCC(=C)[C@H]1CCC(=CC1)C)C | [CID:10104370](https://pubchem.ncbi.nlm.nih.gov/compound/10104370) |
| IMPHY000563 | 3-Butylpyridine | CCCCc1cccnc1 | [CID:10874](https://pubchem.ncbi.nlm.nih.gov/compound/10874) |
| IMPHY000619 | Perillyl alcohol | OCC1=CCC(CC1)C(=C)C | [CID:10819](https://pubchem.ncbi.nlm.nih.gov/compound/10819) |
| IMPHY000653 | Citrusinine II | COc1c(O)cc(c2c1n(C)c1c(c2=O)cccc1O)O | [CID:10016895](https://pubchem.ncbi.nlm.nih.gov/compound/10016895) |
| IMPHY000688 | 7-Methoxycoumarin | COc1ccc2c(c1)oc(=O)cc2 | [CID:10748](https://pubchem.ncbi.nlm.nih.gov/compound/10748) |
| IMPHY000795 | Octanal | CCCCCCCC=O | [CID:454](https://pubchem.ncbi.nlm.nih.gov/compound/454) |
| IMPHY000879 | Isomeranzin | COc1ccc2c(c1CC(=O)C(C)C)oc(=O)cc2 | [CID:473252](https://pubchem.ncbi.nlm.nih.gov/compound/473252) |
| IMPHY001134 | Osthole | COc1ccc2c(c1CC=C(C)C)oc(=O)cc2 | [CID:10228](https://pubchem.ncbi.nlm.nih.gov/compound/10228) |
| IMPHY001215 | Propanol | CCCO | [CID:1031](https://pubchem.ncbi.nlm.nih.gov/compound/1031) |
| IMPHY001315 | Flavanone | O=C1CC(Oc2c1cccc2)c1ccccc1 | [CID:10251](https://pubchem.ncbi.nlm.nih.gov/compound/10251) |
| IMPHY001435 | Citrusin I | CC[C@@H]([C@@H]1NC(=O)[C@H](CC(C)C)NC(=O)[C@H](Cc2ccccc2)NC(=O)[C@@H](NC(=O)CNC(=O)[C@@H](NC(=O)[C@@H](NC1=O)C)[C@H](O)C)[C@H](O)C)C | [CID:15232519](https://pubchem.ncbi.nlm.nih.gov/compound/15232519) |
| IMPHY001548 | Geranylacetone | C/C(=CCCC(=O)C)/CCC=C(C)C | [CID:1549778](https://pubchem.ncbi.nlm.nih.gov/compound/1549778) |
| IMPHY001552 | Auraptene | C/C(=CCOc1ccc2c(c1)oc(=O)cc2)/CCC=C(C)C | [CID:1550607](https://pubchem.ncbi.nlm.nih.gov/compound/1550607) |
| IMPHY001577 | Ethyl vinyl ketone | CCC(=O)C=C | [CID:15394](https://pubchem.ncbi.nlm.nih.gov/compound/15394) |
| IMPHY001680 | 2-(E)-O-feruloyl-D-galactaric acid | COc1cc(/C=C/C(=O)O[C@H]([C@H]([C@H]([C@@H](C(=O)O)O)O)O)C(=O)O)ccc1O | [CID:14104340](https://pubchem.ncbi.nlm.nih.gov/compound/14104340) |
| IMPHY001849 | 3-Pentanol | CCC(CC)O | [CID:11428](https://pubchem.ncbi.nlm.nih.gov/compound/11428) |
| IMPHY001931 | Vanillin | COc1cc(C=O)ccc1O | [CID:1183](https://pubchem.ncbi.nlm.nih.gov/compound/1183) |
| IMPHY001941 | Sativene | CC([C@H]1CC[C@@]2([C@@H]3[C@H]1[C@@H](CC3)C2=C)C)C | [CID:11830550](https://pubchem.ncbi.nlm.nih.gov/compound/11830550) |
| IMPHY001971 | 1-Penten-3-OL | CCC(C=C)O | [CID:12020](https://pubchem.ncbi.nlm.nih.gov/compound/12020) |
| IMPHY002571 | Citrunobin | COc1cc(O)c(c2c1C=CC(O2)(C)C)C(=O)/C=C/c1ccc(cc1)O | [CID:14542258](https://pubchem.ncbi.nlm.nih.gov/compound/14542258) |
| IMPHY002586 | Gossypetin hexamethyl ether | COc1cc(ccc1OC)c1oc2c(OC)c(OC)cc(c2c(=O)c1OC)OC | [CID:146093](https://pubchem.ncbi.nlm.nih.gov/compound/146093) |
| IMPHY002591 | Sinensetin | COc1ccc(cc1OC)c1cc(=O)c2c(o1)cc(c(c2OC)OC)OC | [CID:145659](https://pubchem.ncbi.nlm.nih.gov/compound/145659) |
| IMPHY002667 | Pentadecanoic acid | CCCCCCCCCCCCCCC(=O)O | [CID:13849](https://pubchem.ncbi.nlm.nih.gov/compound/13849) |
| IMPHY002691 | 2-Phenylpyridine | c1ccc(cc1)c1ccccn1 | [CID:13887](https://pubchem.ncbi.nlm.nih.gov/compound/13887) |
| IMPHY002875 | Undecane | CCCCCCCCCCC | [CID:14257](https://pubchem.ncbi.nlm.nih.gov/compound/14257) |
| IMPHY002886 | 3-Hydroxy-N-methyl-3-phenyl-propylamine | CNCCC(c1ccccc1)O | [CID:2733989](https://pubchem.ncbi.nlm.nih.gov/compound/2733989) |
| IMPHY002904 | Nootkatene | CC(=C)[C@@H]1CC=C2[C@](C1)(C)[C@H](C)CC=C2 | [CID:25200342](https://pubchem.ncbi.nlm.nih.gov/compound/25200342) |
| IMPHY002915 | Benzyl Alcohol | OCc1ccccc1 | [CID:244](https://pubchem.ncbi.nlm.nih.gov/compound/244) |
| IMPHY002939 | Ethyl 2-methylbutyrate | CCOC(=O)C(CC)C | [CID:24020](https://pubchem.ncbi.nlm.nih.gov/compound/24020) |
| IMPHY002949 | 1-Butanol | CCCCO | [CID:263](https://pubchem.ncbi.nlm.nih.gov/compound/263) |
| IMPHY003016 | Lauric acid | CCCCCCCCCCCC(=O)O | [CID:3893](https://pubchem.ncbi.nlm.nih.gov/compound/3893) |
| IMPHY003040 | Tridecanal | CCCCCCCCCCCCC=O | [CID:25311](https://pubchem.ncbi.nlm.nih.gov/compound/25311) |
| IMPHY003050 | Methyl salicylate | COC(=O)c1ccccc1O | [CID:4133](https://pubchem.ncbi.nlm.nih.gov/compound/4133) |
| IMPHY003161 | Byakangelicol | COc1c2ccoc2c(c2c1ccc(=O)o2)OC[C@H]1OC1(C)C | [CID:3055167](https://pubchem.ncbi.nlm.nih.gov/compound/3055167) |
| IMPHY003301 | Octanoic acid | CCCCCCCC(=O)O | [CID:379](https://pubchem.ncbi.nlm.nih.gov/compound/379) |
| IMPHY003316 | Pentadecanal | CCCCCCCCCCCCCCC=O | [CID:17697](https://pubchem.ncbi.nlm.nih.gov/compound/17697) |
| IMPHY003340 | Acetoin | CC(=O)C(O)C | [CID:179](https://pubchem.ncbi.nlm.nih.gov/compound/179) |
| IMPHY003378 | Meranzin | COc1ccc2c(c1C[C@@H]1OC1(C)C)oc(=O)cc2 | [CID:1803558](https://pubchem.ncbi.nlm.nih.gov/compound/1803558) |
| IMPHY003387 | Nootkatol | O[C@H]1C[C@@H](C)[C@]2(C(=C1)CC[C@H](C2)C(=C)C)C | [CID:182645](https://pubchem.ncbi.nlm.nih.gov/compound/182645) |
| IMPHY003485 | Myrcene | C=CC(=C)CCC=C(C)C | [CID:31253](https://pubchem.ncbi.nlm.nih.gov/compound/31253) |
| IMPHY003495 | 2-Methoxy-4-vinylphenol | COc1cc(C=C)ccc1O | [CID:332](https://pubchem.ncbi.nlm.nih.gov/compound/332) |
| IMPHY003512 | Ethyl hexanoate | CCCCCC(=O)OCC | [CID:31265](https://pubchem.ncbi.nlm.nih.gov/compound/31265) |
| IMPHY003513 | Isoamyl alcohol | OCCC(C)C | [CID:31260](https://pubchem.ncbi.nlm.nih.gov/compound/31260) |
| IMPHY003519 | Isoamyl acetate | CC(CCOC(=O)C)C | [CID:31276](https://pubchem.ncbi.nlm.nih.gov/compound/31276) |
| IMPHY003525 | Nonanal | CCCCCCCCC=O | [CID:31289](https://pubchem.ncbi.nlm.nih.gov/compound/31289) |
| IMPHY003536 | Eugenol | C=CCc1ccc(c(c1)OC)O | [CID:3314](https://pubchem.ncbi.nlm.nih.gov/compound/3314) |
| IMPHY003537 | Tetradecanal | CCCCCCCCCCCCCC=O | [CID:31291](https://pubchem.ncbi.nlm.nih.gov/compound/31291) |
| IMPHY003616 | Bicyclogermacrene | C/C/1=CCC/C(=C/[C@H]2[C@@H](CC1)C2(C)C)/C | [CID:13894537](https://pubchem.ncbi.nlm.nih.gov/compound/13894537) |
| IMPHY003649 | 2-Heptenal | CCCC/C=C/C=O | [CID:5283316](https://pubchem.ncbi.nlm.nih.gov/compound/5283316) |
| IMPHY003719 | beta-Copaene | CC([C@@H]1CC[C@]2([C@@H]3[C@H]1C2C(=C)CC3)C)C | [CID:57339298](cid:57339298) |
| IMPHY003760 | 2-Nonenal | CCCCCC/C=C/C=O | [CID:5283335](https://pubchem.ncbi.nlm.nih.gov/compound/5283335) |
| IMPHY003915 | 2-Decenal | CCCCCCC/C=C/C=O | [CID:5283345](https://pubchem.ncbi.nlm.nih.gov/compound/5283345) |
| IMPHY003982 | gamma-Terpinene | CC1=CCC(=CC1)C(C)C | [CID:7461](https://pubchem.ncbi.nlm.nih.gov/compound/7461) |
| IMPHY003992 | Hesperidin | COc1ccc(cc1O)[C@@H]1CC(=O)c2c(O1)cc(cc2O)O[C@@H]1O[C@H](CO[C@@H]2O[C@@H](C)[C@@H]([C@H]([C@H]2O)O)O)[C@H]([C@@H]([C@H]1O)O)O | [CID:10621](https://pubchem.ncbi.nlm.nih.gov/compound/10621) |
| IMPHY004077 | Verbenone | CC1=CC(=O)C2CC1C2(C)C | [CID:29025](https://pubchem.ncbi.nlm.nih.gov/compound/29025) |
| IMPHY004164 | Loliolide | O[C@@H]1C[C@@]2(C)OC(=O)C=C2C(C1)(C)C | [CID:100332](https://pubchem.ncbi.nlm.nih.gov/compound/100332) |
| IMPHY004216 | (1S,2S,7S,8S)-2,6,6,9-tetramethyltricyclo[5.4.0.02,8]undec-9-ene | CC1=CC[C@H]2[C@H]3[C@@H]1[C@@]2(C)CCCC3(C)C | [CID:91753627](https://pubchem.ncbi.nlm.nih.gov/compound/91753627) |
| IMPHY004286 | Longifolene | C=C1C2CCC3C1(C)CCCC(C23)(C)C | [CID:289151](https://pubchem.ncbi.nlm.nih.gov/compound/289151) |
| IMPHY004333 | 3-Methylpentacosane | CCCCCCCCCCCCCCCCCCCCCCC(CC)C | [CID:189001](https://pubchem.ncbi.nlm.nih.gov/compound/189001) |
| IMPHY004399 | Methyl linolenate | CC/C=CC/C=CC/C=CCCCCCCCC(=O)OC | [CID:5319706](https://pubchem.ncbi.nlm.nih.gov/compound/5319706) |
| IMPHY004528 | 1,3-p-Menthadien-7-al | O=CC1=CC=C(CC1)C(C)C | [CID:526762](https://pubchem.ncbi.nlm.nih.gov/compound/526762) |
| IMPHY004533 | 2-Methyltetracosane | CCCCCCCCCCCCCCCCCCCCCCC(C)C | [CID:527459](https://pubchem.ncbi.nlm.nih.gov/compound/527459) |
| IMPHY004631 | Stearic acid | CCCCCCCCCCCCCCCCCC(=O)O | [CID:5281](https://pubchem.ncbi.nlm.nih.gov/compound/5281) |
| IMPHY004645 | beta-Cryptoxanthin | O[C@@H]1CC(=C(C(C1)(C)C)/C=C/C(=C/C=C/C(=C/C=C/C=C(/C=C/C=C(/C=C/C1=C(C)CCCC1(C)C)C)C)/C)/C)C | [CID:5281235](https://pubchem.ncbi.nlm.nih.gov/compound/5281235) |
| IMPHY004693 | Antheraxanthin | C/C(=CC=CC=C(C=CC=C(C=C[C@]12O[C@]2(C)C[C@H](CC1(C)C)O)/C)/C)/C=C/C=C(/C=C/C1=C(C)C[C@H](CC1(C)C)O)C | [CID:5281223](https://pubchem.ncbi.nlm.nih.gov/compound/5281223) |
| IMPHY004880 | 3-Methyltricosane | CCCCCCCCCCCCCCCCCCCCC(CC)C | [CID:528561](https://pubchem.ncbi.nlm.nih.gov/compound/528561) |
| IMPHY004889 | Cinnamyl alcohol | OC/C=C/c1ccccc1 | [CID:5315892](https://pubchem.ncbi.nlm.nih.gov/compound/5315892) |
| IMPHY005076 | 2-Pentylpyridine | CCCCCc1ccccn1 | [CID:16800](https://pubchem.ncbi.nlm.nih.gov/compound/16800) |
| IMPHY005127 | Hexyl butyrate | CCCCCCOC(=O)CCC | [CID:17525](https://pubchem.ncbi.nlm.nih.gov/compound/17525) |
| IMPHY005128 | Oxypeucedanin hydrate | O=c1ccc2c(o1)cc1c(c2OC[C@H](C(O)(C)C)O)cco1 | [CID:17536](https://pubchem.ncbi.nlm.nih.gov/compound/17536) |
| IMPHY005336 | Citbrasine | COc1c(OC)c(OC)c2c(c1O)c(=O)c1c(n2C)c(O)ccc1 | [CID:19093029](https://pubchem.ncbi.nlm.nih.gov/compound/19093029) |
| IMPHY005345 | 1-Octen-3-OL | CCCCCC(C=C)O | [CID:18827](https://pubchem.ncbi.nlm.nih.gov/compound/18827) |
| IMPHY005428 | Bergapten | COc1c2ccc(=O)oc2cc2c1cco2 | [CID:2355](https://pubchem.ncbi.nlm.nih.gov/compound/2355) |
| IMPHY005526 | 2-Undecenal | CCCCCCCC/C=C/C=O | [CID:5283356](https://pubchem.ncbi.nlm.nih.gov/compound/5283356) |
| IMPHY005549 | 2-Octenal | CCCCC/C=C/C=O | [CID:5283324](https://pubchem.ncbi.nlm.nih.gov/compound/5283324) |
| IMPHY005551 | 2,4-Heptadienal | CC/C=C/C=C/C=O | [CID:5283321](https://pubchem.ncbi.nlm.nih.gov/compound/5283321) |
| IMPHY005575 | Triprolidine | Cc1ccc(cc1)/C(=CCN1CCCC1)/c1ccccn1 | [CID:5282443](https://pubchem.ncbi.nlm.nih.gov/compound/5282443) |
| IMPHY005609 | beta-Sinensal | C=CC(=C)CC/C=C(/CC/C=C(/C=O)C)C | [CID:5281535](https://pubchem.ncbi.nlm.nih.gov/compound/5281535) |
| IMPHY005803 | Furaneol | CC1OC(=C(C1=O)O)C | [CID:19309](https://pubchem.ncbi.nlm.nih.gov/compound/19309) |
| IMPHY005981 | Cryptoflavin | OC1CC(=C(C(C1)(C)C)/C=C/C(=C/C=C/C(=C/C=C/C=C(/C=C/C=C(/C1C=C2C(O1)(C)CCCC2(C)C)C)C)/C)/C)C | [CID:5376350](https://pubchem.ncbi.nlm.nih.gov/compound/5376350) |
| IMPHY005993 | 2-Pentanol | CCCC(O)C | [CID:22386](https://pubchem.ncbi.nlm.nih.gov/compound/22386) |
| IMPHY006009 | Acetal | CCOC(OCC)C | [CID:7765](https://pubchem.ncbi.nlm.nih.gov/compound/7765) |
| IMPHY006032 | 2-Ethylhexanol | CCCCC(CO)CC | [CID:7720](https://pubchem.ncbi.nlm.nih.gov/compound/7720) |
| IMPHY006033 | Ethyl butyrate | CCCC(=O)OCC | [CID:7762](https://pubchem.ncbi.nlm.nih.gov/compound/7762) |
| IMPHY006059 | 2-Methyl-5-phenylpyridine | Cc1ccc(cn1)c1ccccc1 | [CID:76744](https://pubchem.ncbi.nlm.nih.gov/compound/76744) |
| IMPHY006097 | citracridone I | COc1c(O)ccc2c1n(C)c1c(c2=O)c(O)cc2c1C=CC(O2)(C)C | [CID:5487591](https://pubchem.ncbi.nlm.nih.gov/compound/5487591) |
| IMPHY006101 | Citrusinine I | COc1c(OC)cc(c2c1n(C)c1c(c2=O)cccc1O)O | [CID:5487772](https://pubchem.ncbi.nlm.nih.gov/compound/5487772) |
| IMPHY006145 | p-Cymene | Cc1ccc(cc1)C(C)C | [CID:7463](https://pubchem.ncbi.nlm.nih.gov/compound/7463) |
| IMPHY006279 | 2-Phenylethanol | OCCc1ccccc1 | CID:6054, |
| IMPHY006314 | Ethyl 3-hydroxyhexanoate | CCCC(CC(=O)OCC)O | [CID:61293](https://pubchem.ncbi.nlm.nih.gov/compound/61293) |
| IMPHY006322 | Safranal | O=CC1=C(C)C=CCC1(C)C | [CID:61041](https://pubchem.ncbi.nlm.nih.gov/compound/61041) |
| IMPHY006330 | Octyl butyrate | CCCCCCCCOC(=O)CCC | [CID:61030](https://pubchem.ncbi.nlm.nih.gov/compound/61030) |
| IMPHY006347 | Hexanal | CCCCCC=O | [CID:6184](https://pubchem.ncbi.nlm.nih.gov/compound/6184) |
| IMPHY006362 | Ascorbic acid | OC[C@@H]([C@H]1OC(=O)C(=C1O)O)O | [CID:54670067](https://pubchem.ncbi.nlm.nih.gov/compound/54670067) |
| IMPHY006419 | 6-Methyl-3,5-heptadien-2-one | CC(=C/C=C/C(=O)C)C | [CID:5370101](https://pubchem.ncbi.nlm.nih.gov/compound/5370101) |
| IMPHY006486 | Squalene | C/C(=CCC/C=C(/CC/C=C(/CCC=C(C)C)C)C)/CC/C=C(/CCC=C(C)C)C | [CID:638072](https://pubchem.ncbi.nlm.nih.gov/compound/638072) |
| IMPHY006550 | Thymol | Cc1ccc(c(c1)O)C(C)C | [CID:6989](https://pubchem.ncbi.nlm.nih.gov/compound/6989) |
| IMPHY006574 | Ethyl benzoate | CCOC(=O)c1ccccc1 | [CID:7165](https://pubchem.ncbi.nlm.nih.gov/compound/7165) |
| IMPHY006618 | Nobiletin | COc1cc(ccc1OC)c1cc(=O)c2c(o1)c(OC)c(c(c2OC)OC)OC | [CID:72344](https://pubchem.ncbi.nlm.nih.gov/compound/72344) |
| IMPHY006934 | 2-Methyl-1-butanol | CCC(CO)C | [CID:8723](https://pubchem.ncbi.nlm.nih.gov/compound/8723) |
| IMPHY006948 | beta-Terpineol | CC(=C)C1CCC(CC1)(C)O | [CID:8748](https://pubchem.ncbi.nlm.nih.gov/compound/8748) |
| IMPHY006970 | Decanal | CCCCCCCCCC=O | CID:8175 |
| IMPHY006996 | 1-Dodecene | CCCCCCCCCCC=C | CID:8183 |
| IMPHY007007 | Ethyl isobutyrate | CCOC(=O)C(C)C | CID:7342 |
| IMPHY007039 | Acetophenone | CC(=O)c1ccccc1 | CID:7410 |
| IMPHY007067 | Linalyl acetate | C=CC(OC(=O)C)(CCC=C(C)C)C | CID:8294 |
| IMPHY007076 | Undecanal | CCCCCCCCCCC=O | CID:8186 |
| IMPHY007099 | Decyl acetate | CCCCCCCCCCOC(=O)C | CID:8167 |
| IMPHY007137 | Methyl 2-(methylamino)benzoate | COC(=O)c1ccccc1NC | CID:6826 |
| IMPHY007153 | 3-Methyl-2-phenylpyridine | Cc1cccnc1c1ccccc1 | CID:66302 |
| IMPHY007162 | 2-Methyl-3-buten-2-OL | C=CC(O)(C)C | CID:8257 |
| IMPHY007171 | 1-Hexanol | CCCCCCO | CID:8103 |
| IMPHY007186 | Heptanal | CCCCCCC=O | CID:8130 |
| IMPHY007197 | Isopimpinellin | COc1c2ccc(=O)oc2c(c2c1cco2)OC | CID:68079 |
| IMPHY007198 | Isoimperatorin | CC(=CCOc1c2ccoc2cc2c1ccc(=O)o2)C | CID:68081 |
| IMPHY007202 | Nonane | CCCCCCCCC | CID:8141 |
| IMPHY007204 | Dodecanal | CCCCCCCCCCCC=O | CID:8194 |
| IMPHY007206 | Dodecyl acetate | CCCCCCCCCCCCOC(=O)C | CID:8205 |
| IMPHY007207 | Tangeretin | COc1ccc(cc1)c1cc(=O)c2c(o1)c(OC)c(c(c2OC)OC)OC | CID:68077 |
| IMPHY007214 | Octyl acetate | CCCCCCCCOC(=O)C | CID:8164 |
| IMPHY007231 | 8-[(2S)-2,3-dihydroxy-3-methylbutyl]-7-methoxychromen-2-one | COc1ccc2c(c1C[C@@H](C(O)(C)C)O)oc(=O)cc2 | CID:821434 |
| IMPHY007276 | Nonan-1-ol | CCCCCCCCCO | CID:8914 |
| IMPHY007299 | Phellandral | O=CC1=CCC(CC1)C(C)C | CID:89488 |
| IMPHY007302 | Hexyl acetate | CCCCCCOC(=O)C | CID:8908 |
| IMPHY007327 | Palmitic acid | CCCCCCCCCCCCCCCC(=O)O | CID:985 |
| IMPHY007329 | Phellopterin | COc1c2ccoc2c(c2c1ccc(=O)o2)OCC=C(C)C | CID:98608 |
| IMPHY007354 | Hexanoic acid | CCCCCC(=O)O | CID:8892 |
| IMPHY007376 | beta-Cubebene | CC([C@@H]1CC[C@H]([C@]23[C@H]1[C@H]2C(=C)CC3)C)C | CID:93081 |
| IMPHY007417 | Ethyl acetate | CCOC(=O)C | CID:8857 |
| IMPHY007421 | Citronellyl acetate | CC(CCC=C(C)C)CCOC(=O)C | CID:9017 |
| IMPHY007467 | Hexadecanal | CCCCCCCCCCCCCCCC=O | CID:984 |
| IMPHY007481 | Dehydrololiolide | O=C1C=C2C(O1)(C)CC(=O)CC2(C)C | CID:9815531 |
| IMPHY007528 | Cadinane | C[C@H]1CC[C@@H]2[C@@H](C1)[C@@H](CC[C@@H]2C)C(C)C | CID:9548708 |
| IMPHY007539 | Phenylacetaldehyde | O=CCc1ccccc1 | CID:998 |
| IMPHY007620 | 1-Octanol | CCCCCCCCO | CID:957 |
| IMPHY007840 | Spathulenol | C=C1CC[C@@H]2[C@H]([C@H]3[C@H]1CC[C@]3(C)O)C2(C)C | CID:92231 |
| IMPHY007967 | Ethyl 3-hydroxybutyrate | CCOC(=O)CC(O)C | CID:62572 |
| IMPHY008065 | Ethyl dodecanoate | CCCCCCCCCCCC(=O)OCC | CID:7800 |
| IMPHY008066 | Ethyl octanoate | CCCCCCCC(=O)OCC | CID:7799 |
| IMPHY008163 | 2-Methyl-5-vinylpyridine | C=Cc1ccc(nc1)C | CID:8817 |
| IMPHY008597 | Nonyl acetate | CCCCCCCCCOC(=O)C | CID:8918 |
| IMPHY008722 | 3-Phenylpropionic acid | OC(=O)CCc1ccccc1 | CID:107 |
| IMPHY008772 | Propyl butyrate | CCCOC(=O)CCC | CID:7770 |
| IMPHY008913 | Imperatorin | CC(=CCOc1c2occc2cc2c1oc(=O)cc2)C | CID:10212 |
| IMPHY008991 | Benzyl acetate | CC(=O)OCc1ccccc1 | CID:8785 |
| IMPHY009349 | Perillic acid | CC(=C)C1CCC(=CC1)C(=O)O | CID:1256 |
| IMPHY009360 | Nootkatone | O=C1C[C@@H](C)[C@]2(C(=C1)CC[C@H](C2)C(=C)C)C | CID:1268142 |
| IMPHY009626 | 2-Nonanol | CCCCCCCC(O)C | CID:12367 |
| IMPHY009749 | Heptadecanal | CCCCCCCCCCCCCCCCC=O | CID:71552 |
| IMPHY009752 | beta-Cyclocitral | O=CC1=C(C)CCCC1(C)C | CID:9895 |
| IMPHY009859 | Octadecanal | CCCCCCCCCCCCCCCCCC=O | CID:12533 |
| IMPHY009874 | Myrtenyl acetate | CC(=O)OCC1=CCC2CC1C2(C)C | CID:61262 |
| IMPHY009946 | Benzaldehyde | O=Cc1ccccc1 | CID:240 |
| IMPHY010080 | beta-Elemene | C=C[C@]1(C)CC[C@H](C[C@H]1C(=C)C)C(=C)C | CID:6918391 |
| IMPHY010620 | 3,3',4',5,6,7,8-Heptamethoxyflavone | COc1cc(ccc1OC)c1oc2c(OC)c(OC)c(c(c2c(=O)c1OC)OC)OC | CID:150893 |
| IMPHY010781 | Limonene oxide, cis-(-)- | CC(=C)[C@H]1CC[C@]2([C@@H](C1)O2)C | CID:6452061 |
| IMPHY010841 | Ethyl linoleate | CCCCC/C=CC/C=CCCCCCCCC(=O)OCC | CID:5282184 |
| IMPHY011004 | p-Mentha-1,3,8-triene | CC1=CC=C(CC1)C(=C)C | CID:176983 |
| IMPHY011086 | alpha-Sinensal | C=C/C(=C/C/C=C(/CC/C=C(/C=O)C)C)/C | CID:5281534 |
| IMPHY011202 | Narirutin | Oc1ccc(cc1)[C@@H]1CC(=O)c2c(O1)cc(cc2O)O[C@@H]1O[C@H](CO[C@@H]2O[C@@H](C)[C@@H]([C@H]([C@H]2O)O)O)[C@H]([C@@H]([C@H]1O)O)O | CID:442431 |
| IMPHY011381 | (4S)-1-Methyl-4-(prop-1-EN-2-YL)-7-oxabicyclo[4.1.0]heptane | CC(=C)[C@H]1CCC2(C(C1)O2)C | CID:10953718 |
| IMPHY011392 | 3-Carene | CC1=CCC2C(C1)C2(C)C | CID:26049 |
| IMPHY011395 | Scoparone | COc1cc2oc(=O)ccc2cc1OC | CID:8417 |
| IMPHY011396 | 4-Carvomenthenol | CC1=CCC(CC1)(O)C(C)C | CID:11230 |
| IMPHY011408 | Demethylnobiletin | COc1cc(ccc1OC)c1cc(=O)c2c(o1)c(OC)c(c(c2O)OC)OC | CID:358832 |
| IMPHY011474 | Bergamottin | C/C(=CCOc1c2ccoc2cc2c1ccc(=O)o2)/CCC=C(C)C | CID:5471349 |
| IMPHY011519 | alpha-Terpinyl acetate | CC(=O)OC(C1CCC(=CC1)C)(C)C | CID:111037 |
| IMPHY011549 | Citropten | COc1cc(OC)c2c(c1)oc(=O)cc2 | CID:2775 |
| IMPHY011552 | (1R)-2-methyl-5-propan-2-ylbicyclo[3.1.0]hex-2-ene | CC1=CCC2([C@@H]1C2)C(C)C | CID:6451618 |
| IMPHY011562 | 2-Hexenal | CCC/C=C/C=O | CID:5281168 |
| IMPHY011581 | alpha-Selinene | CC1=CCC[C@]2([C@H]1C[C@@H](CC2)C(=C)C)C | CID:10856614 |
| IMPHY011586 | (S,1Z,6Z)-8-Isopropyl-1-methyl-5-methylenecyclodeca-1,6-diene | C/C/1=C/CCC(=C)/C=C[C@@H](CC1)C(C)C | CID:91723653 |
| IMPHY011588 | cis-3-Hexen-1-ol | OCC/C=CCC | CID:5281167 |
| IMPHY011590 | d-Borneol | O[C@@H]1C[C@H]2C([C@@]1(C)CC2)(C)C | CID:61060 |
| IMPHY011599 | Terpinolene | CC1=CCC(=C(C)C)CC1 | CID:11463 |
| IMPHY011620 | Lutein | C/C(=CC=CC=C(C=CC=C(C=CC1=C(C)C[C@H](CC1(C)C)O)/C)/C)/C=C/C=C(/C=C/[C@H]1C(=C[C@@H](CC1(C)C)O)C)C | CID:5281243 |
| IMPHY011632 | Farnesol | OC/C=C(/CC/C=C(/CCC=C(C)C)C)C | CID:445070 |
| IMPHY011643 | alpha-Terpinene | CC1=CC=C(CC1)C(C)C | CID:7462 |
| IMPHY011647 | Geranyl acetate | C/C(=CCOC(=O)C)/CCC=C(C)C | CID:1549026 |
| IMPHY011648 | Neryl acetate | C/C(=C/COC(=O)C)/CCC=C(C)C | CID:1549025 |
| IMPHY011657 | cis-beta-Farnesene | C=CC(=C)CC/C=C(CCC=C(C)C)/C | CID:5317319 |
| IMPHY011658 | beta-Farnesene | C=CC(=C)CC/C=C(/CCC=C(C)C)C | CID:5281517 |
| IMPHY011659 | alpha-Muurolene | CC1=C[C@@H]2[C@H](CC1)C(=CC[C@H]2C(C)C)C | CID:12306047 |
| IMPHY011660 | (+)-alpha-Cadinene | CC1=C[C@@H]2[C@@H](CC1)C(=CC[C@H]2C(C)C)C | CID:12306048 |
| IMPHY011761 | Humulene | C/C/1=CCC(C)(C)/C=C/C/C(=C/CC1)/C | CID:5281520 |
| IMPHY011789 | Citral | O=C/C=C(/CCC=C(C)C)C | CID:638011 |
| IMPHY011790 | Neral | O=C/C=C(CCC=C(C)C)/C | CID:643779 |
| IMPHY011792 | gamma-Muurolene | CC1=C[C@@H]2[C@H](CC1)C(=C)CC[C@H]2C(C)C | CID:12313020 |
| IMPHY011793 | (+)-gamma-Cadinene | CC1=C[C@@H]2[C@@H](CC1)C(=C)CC[C@H]2C(C)C | CID:6432404 |
| IMPHY011817 | alpha-Farnesene | C=C/C(=C/C/C=C(/CCC=C(C)C)C)/C | CID:5281516 |
| IMPHY011890 | Elemol | C=C[C@]1(C)CC[C@H](C[C@H]1C(=C)C)C(O)(C)C | CID:92138 |
| IMPHY011896 | Valencene | CC(=C)[C@@H]1CCC2=CCC[C@H]([C@@]2(C1)C)C | CID:9855795 |
| IMPHY011957 | (+)-delta-Cadinene | CC1=C[C@@H]2C(=C(C)CC[C@H]2C(C)C)CC1 | CID:441005 |
| IMPHY011960 | Cinnamic acid | OC(=O)/C=C/c1ccccc1 | CID:444539 |
| IMPHY011965 | (+)-beta-Phellandrene | CC([C@@H]1CCC(=C)C=C1)C | CID:442484 |
| IMPHY011973 | (-)-cis-Carveol | CC(=C)[C@@H]1CC=C([C@@H](C1)O)C | CID:330573 |
| IMPHY011974 | 4-Hydroxycinnamic acid | OC(=O)/C=C/c1ccc(cc1)O | CID:637542 |
| IMPHY011984 | Ethyl cinnamate | CCOC(=O)/C=C/c1ccccc1 | CID:637758 |
| IMPHY011988 | (-)-trans-Carveol | CC(=C)[C@@H]1CC=C([C@H](C1)O)C | CID:94221 |
| IMPHY011999 | Carveol | CC(=C)C1CC=C(C(C1)O)C | CID:7438 |
| IMPHY012002 | (+)-Dihydrocarvone | CC(=C)[C@@H]1CC[C@H](C(=O)C1)C | CID:22227 |
| IMPHY012036 | Camphor | O=C1CC2C(C1(C)CC2)(C)C | CID:2537 |
| IMPHY012058 | Linalool | C=CC(CCC=C(C)C)(O)C | CID:6549 |
| IMPHY012059 | Isobutanol | OCC(C)C | CID:6560 |
| IMPHY012061 | alpha-Pinene | CC1=CCC2CC1C2(C)C | CID:6654 |
| IMPHY012075 | Carvone | CC(=C)C1CC=C(C(=O)C1)C | CID:7439 |
| IMPHY012086 | Citronellal | O=CCC(CCC=C(C)C)C | CID:7794 |
| IMPHY012100 | Nonanoic acid | CCCCCCCCC(=O)O | CID:8158 |
| IMPHY012104 | Citronellol | OCCC(CCC=C(C)C)C | CID:8842 |
| IMPHY012132 | Propyl hexanoate | CCCCCC(=O)OCCC | CID:12293 |
| IMPHY012145 | 1-Tetradecene | CCCCCCCCCCCCC=C | CID:14260 |
| IMPHY012147 | beta-Pinene | C=C1CCC2CC1C2(C)C | CID:14896 |
| IMPHY012153 | Undecyl acetate | CCCCCCCCCCCOC(=O)C | CID:15605 |
| IMPHY012157 | Perillaldehyde | O=CC1=CCC(CC1)C(=C)C | CID:16441 |
| IMPHY012160 | alpha-Terpineol | CC1=CCC(CC1)C(O)(C)C | CID:17100 |
| IMPHY012165 | Sabinene | C=C1CCC2(C1C2)C(C)C | CID:18818 |
| IMPHY012168 | (1S,2S,6S,7R,8R)-1,3-dimethyl-8-propan-2-yltricyclo[4.4.0.02,7]dec-3-ene | CC([C@H]1CC[C@]2([C@@H]3[C@@H]1[C@H]2C(=CC3)C)C)C | CID:101607926 |
| IMPHY012175 | D-Limonene | CC1=CC[C@@H](CC1)C(=C)C | CID:440917 |
| IMPHY012198 | Verbenol | CC1=CC(O)C2CC1C2(C)C | CID:61126 |
| IMPHY012254 | Isopinocamphone | O=C1C[C@H]2C[C@@H]([C@@H]1C)C2(C)C | CID:84532 |
| IMPHY012261 | alpha-Bergamotene | CC(=CCCC1(C)C2CC=C(C1C2)C)C | CID:86608 |
| IMPHY012362 | P-Mentha-2,8-dien-1-ol | CC(=C)C1CCC(C=C1)(C)O | CID:155626 |
| IMPHY012384 | 4-[(3,3-Dimethyloxiran-2-yl)methoxy]furo[3,2-g]chromen-7-one | O=c1ccc2c(o1)cc1c(c2OCC2OC2(C)C)cco1 | CID:160544 |
| IMPHY012589 | 3-(1,5-Dimethyl-4-hexenyl)-6-methylene-1-cyclohexene | CC(C1CCC(=C)C=C1)CCC=C(C)C | CID:519764 |
| IMPHY012654 | Nerol | OC/C=C(CCC=C(C)C)/C | CID:643820 |
| IMPHY012667 | Caryophyllene oxide | C=C1CC[C@H]2O[C@@]2(CC[C@@H]2[C@@H]1CC2(C)C)C | CID:1742210 |
| IMPHY012739 | (Z)-beta-Ocimene | C=C/C(=CCC=C(C)C)/C | CID:5320250 |
| IMPHY013080 | alpha-Calacorene | CC([C@@H]1CC=C(c2c1cc(C)cc2)C)C | CID:12302243 |
| IMPHY013093 | delta-Elemene | C=C[C@@]1(C)CCC(=C[C@@H]1C(=C)C)C(C)C | CID:12309449 |
| IMPHY013349 | Limonin 17-beta-D-glucopyranoside | OC[C@H]1O[C@@H](O[C@H]([C@]2(C)CC[C@H]3[C@@]([C@@]42O[C@@H]4C(=O)O)(C)C(=O)C[C@@H]2[C@]43COC(=O)C[C@@H]4OC2(C)C)c2cocc2)[C@@H]([C@H]([C@@H]1O)O)O | CID:24820753 |
| IMPHY013764 | (-)-Carvomenthone | CC([C@H]1CC[C@@H](C(=O)C1)C)C | CID:6432474 |
| IMPHY013775 | Methyl butyrate | CCCC(=O)OC | CID:12180 |
| IMPHY014060 | Xanthotoxol geranyl ether | C/C(=CCOc1c2occc2cc2c1oc(=O)cc2)/CCC=C(C)C | CID:5317564 |
| IMPHY014708 | beta-Selinene | C=C1CCC[C@]2([C@H]1C[C@@H](CC2)C(=C)C)C | CID:442393 |
| IMPHY014806 | Caswell No. 264AB | CC([C@@H]1CC[C@H]([C@]23[C@H]1[C@H]2C(=CC3)C)C)C | CID:442359 |
| IMPHY014811 | alpha-Phellandrene | CC1=CCC(C=C1)C(C)C | CID:7460 |
| IMPHY014831 | beta-Caryophyllene | C/C/1=CCCC(=C)[C@@H]2[C@@H](CC1)C(C2)(C)C | CID:5281515 |
| IMPHY014835 | (E)-beta-ocimene | C=C/C(=C/CC=C(C)C)/C | CID:5281553 |
| IMPHY014847 | Bornyl acetate | CC(=O)OC1CC2C(C1(C)CC2)(C)C | CID:6448 |
| IMPHY014852 | Camphene | C=C1C2CCC(C1(C)C)C2 | CID:6616 |
| IMPHY014871 | cis-Nerolidol | C=CC(CC/C=C(CCC=C(C)C)/C)(O)C | CID:5320128 |
| IMPHY014874 | cis-Sabinene hydrate | C[C@@H]1CC[C@@]2(C1C2)C(C)C | CID:101629835 |
| IMPHY014881 | Copaene | CC(C1CCC2(C3C1C2C(=CC3)C)C)C | CID:19725 |
| IMPHY014923 | Geraniol | OC/C=C(/CCC=C(C)C)C | CID:637566 |
| IMPHY014988 | Limonene | CC1=CCC(CC1)C(=C)C | CID:22311 |
| IMPHY014989 | trans-Linalool oxide | C=C[C@]1(C)CC[C@H](O1)C(O)(C)C | CID:6432254 |
| IMPHY014990 | Linoleic acid | CCCCC/C=CC/C=CCCCCCCCC(=O)O | CID:5280450 |
| IMPHY015003 | Menthol | CC1CCC(C(C1)O)C(C)C | CID:1254 |
| IMPHY015022 | Nerolidol | C=CC(CC/C=C(/CCC=C(C)C)C)(O)C | CID:5284507 |
| IMPHY015094 | (+)-trans-Limonene oxide | CC(=C)[C@@H]1CC[C@]2([C@@H](C1)O2)C | CID:449290 |
| IMPHY015123 | alpha-Copaene | CC([C@@H]1CC[C@]2([C@@H]3[C@H]1C2C(=CC3)C)C)C | CID:70678558 |
| IMPHY015613 | Diethyl succinate | CCOC(=O)CCC(=O)OCC | CID:31249 |
| IMPHY015660 | Ethyl decanoate | CCCCCCCCCC(=O)OCC | CID:8048 |
| IMPHY015670 | Ethyl propionate | CCOC(=O)CC | CID:7749 |
| IMPHY015889 | Perillyl acetate | CC(=O)OCC1=CCC(CC1)C(=C)C | CID:61780 |
| IMPHY016012 | Allo-Aromadendrene | C[C@@H]1CC[C@H]2[C@@H]1C1C(C1(C)C)CCC2=C | CID:42608158 |
| IMPHY016054 | trans-alpha-Bergamotene | CC(=CCC[C@]1(C)[C@H]2CC=C([C@@H]1C2)C)C | CID:6429302 |
| IMPHY016635 | Epoxyaurapten | C/C(=CCOc1ccc2c(c1)oc(=O)cc2)/CCC1OC1(C)C | CID:9796891 |
| IMPHY016636 | Epoxybergamottin | C/C(=CCOc1c2ccoc2cc2c1ccc(=O)o2)/CCC1OC1(C)C | CID:9946625 |
| IMPHY016923 | 4-Hydroxy-2,6,6-trimethyl-1-cyclohexanone | OC1CC(C)C(=O)C(C1)(C)C | CID: 14322959 |
| IMPHY016930 | Cnidicin | CC(=CCOc1c2occc2c(c2c1oc(=O)cc2)OCC=C(C)C)C | CID:10043694 |
| IMPHY016959 | 5-Hexyl-4-methyldihydrofuran-2(3H)-one | CCCCCCC1OC(=O)CC1C | CID:106756 |
| IMPHY017028 | s,s'-Ethylidene dithioacetate | CC(SC(=O)C)SC(=O)C | CID:12453928 |
| IMPHY017120 | Propyl 3-hydroxyhexanoate | CCCOC(=O)CC(CCC)O | CID:14915819 |
| IMPHY017141 | (2e,4e,6e)-2,4,6-Nonatrienal | CC/C=C/C=C/C=C/C=O | CID:15755823 |
| IMPHY017183 | 3-(4-hydroxyphenyl)propyl Acetate | CC(=O)OCCCc1ccc(cc1)O | CID:187242 |
| IMPHY017269 | Dioctyl adipate | CCCCCCCCOC(=O)CCCCC(=O)OCCCCCCCC | CID:31271 |
| IMPHY017390 | 3-Hydroxy-beta-ionone | OC1CC(=C(C(C1)(C)C)/C=C/C(=O)C)C | CID:5363700 |
| IMPHY017404 | Ethyl trans-2-hexenoate | CCC/C=C/C(=O)OCC | CID:5364778 |
| IMPHY017417 | Butyl crotonate | CCCCOC(=O)/C=C/C | CID:5366039 |
| IMPHY017426 | Butyl (2E)-2-hexenoate | CCCCOC(=O)/C=C/CCC | CID:5370502 |
| IMPHY017565 | 4-Phenyl-2-butanol | CC(CCc1ccccc1)O | CID:61302 |
| IMPHY017588 | 1,10-Dihydronootkatone | O=C1CC(C)C2(C(C1)CCC(C2)C(=C)C)C | CID:6427078 |
| IMPHY017650 | 1,2-Epoxyhumulene | CC1CCCC2(C)OC2CC(/C=C/C1)(C)C | CID:6432846 |
| IMPHY017658 | 9-Hydroxy-4-(3,7-dimethyl-2,6-octadienyloxy)-psoralen | COc1c2occc2c(c2c1oc(=O)cc2)OC/C=C(/CCC=C(C)C)C | CID:6440422 |
| IMPHY017758 | Diisopropyl trisulfide | CC(SSSC(C)C)C | CID:80046 |
| IMPHY017805 | Ethyl 3-acetoxyhexanoate | CCCC(OC(=O)C)CC(=O)OCC | CID:89464 |
| IMPHY017928 | 1-Ethoxy-1 methoxy-ethane | CCOC(OC)C | CID: 25298 |

| ***16. Plant name: Curcuma longa*** | | | |
| --- | --- | --- | --- |
| **IMPPAT Phytochemical identifier:** | **Phytochemical name:** | **SMILES:** | **CID** |
| IMPHY000399 | beta-Bisabolene | CC(=CCCC(=C)[C@H]1CCC(=CC1)C)C | 10104370 |
| IMPHY002825 | 2-(4-Methylphenyl)propan-2-ol | Cc1ccc(cc1)C(O)(C)C | 14529 |
| IMPHY003485 | Myrcene | C=CC(=C)CCC=C(C)C | 31253 |
| IMPHY003567 | alpha-Fenchene | C=C1CC2C(C1CC2)(C)C | 28930 |
| IMPHY003982 | gamma-Terpinene | CC1=CCC(=CC1)C(C)C | 7461 |
| IMPHY005042 | Bisacumol | CC(=CC(CC(c1ccc(cc1)C)C)O)C | 5315469 |
| IMPHY005798 | Curlone | CC(C1CCC(=C)C=C1)CC(=O)C=C(C)C | 196216 |
| IMPHY006145 | p-Cymene | Cc1ccc(cc1)C(C)C | 7463 |
| IMPHY006463 | Germacrone | C/C/1=CCC(=C(C)C)C(=O)C/C(=C/CC1)/C | 6436348 |
| IMPHY006950 | Tricyclene | CC12C3C1CC(C2(C)C)C3 | 79035 |
| IMPHY008150 | 1-Methyl-4-(prop-1-en-2-yl)benzene | Cc1ccc(cc1)C(=C)C | 62385 |
| IMPHY010072 | Eucalyptol | CC12CCC(CC1)C(O2)(C)C | 2758 |
| IMPHY010677 | Curcuphenol | CC(=CCC[C@H](c1ccc(cc1O)C)C)C | 360253 |
| IMPHY011004 | p-Mentha-1,3,8-triene | CC1=CC=C(CC1)C(=C)C | 176983 |
| IMPHY011087 | alpha-Turmerone | CC(=CC(=O)CC(C1CC=C(C=C1)C)C)C | 14632996 |
| IMPHY011392 | 3-Carene | CC1=CCC2C(C1)C2(C)C | 26049 |
| IMPHY011396 | 4-Carvomenthenol | CC1=CCC(CC1)(O)C(C)C | 11230 |
| IMPHY011521 | 2-Undecanone | CCCCCCCCCC(=O)C | 8163 |
| IMPHY011557 | 4-Isopropylbenzyl alcohol | OCc1ccc(cc1)C(C)C | 325 |
| IMPHY011599 | Terpinolene | CC1=CCC(=C(C)C)CC1 | 11463 |
| IMPHY011643 | alpha-Terpinene | CC1=CC=C(CC1)C(C)C | 7462 |
| IMPHY011647 | Geranyl acetate | C/C(=CCOC(=O)C)/CCC=C(C)C | 1549026 |
| IMPHY011658 | beta-Farnesene | C=CC(=C)CC/C=C(/CCC=C(C)C)C | 5281517 |
| IMPHY011745 | Zingiberene | CC(=CCC[C@@H]([C@H]1CC=C(C=C1)C)C)C | 92776 |
| IMPHY011761 | Humulene | C/C/1=CCC(C)(C)/C=C/C/C(=C/CC1)/C | 5281520 |
| IMPHY012058 | Linalool | C=CC(CCC=C(C)C)(O)C | 6549 |
| IMPHY012061 | alpha-Pinene | CC1=CCC2CC1C2(C)C | 6654 |
| IMPHY012147 | beta-Pinene | C=C1CCC2CC1C2(C)C | 14896 |
| IMPHY012160 | alpha-Terpineol | CC1=CCC(CC1)C(O)(C)C | 17100 |
| IMPHY012165 | Sabinene | C=C1CCC2(C1C2)C(C)C | 18818 |
| IMPHY012279 | alpha-Curcumene | CC(=CCCC(c1ccc(cc1)C)C)C | 92139 |
| IMPHY012381 | ar-Turmerone | CC(=CC(=O)C[C@@H](c1ccc(cc1)C)C)C | 160512 |
| IMPHY012589 | 3-(1,5-Dimethyl-4-hexenyl)-6-methylene-1-cyclohexene | CC(C1CCC(=C)C=C1)CCC=C(C)C | 519764 |
| IMPHY012739 | (Z)-beta-Ocimene | C=C/C(=CCC=C(C)C)/C | 5320250 |
| IMPHY014811 | alpha-Phellandrene | CC1=CCC(C=C1)C(C)C | 7460 |
| IMPHY014831 | beta-Caryophyllene | C/C/1=CCCC(=C)[C@@H]2[C@@H](CC1)C(C2)(C)C | 5281515 |
| IMPHY014835 | (E)-beta-ocimene | C=C/C(=C/CC=C(C)C)/C | 5281553 |
| IMPHY014863 | cis-alpha-Bergamotene | CC(=CCCC1(C)[C@@H]2CC=C([C@H]1C2)C)C | 91753502 |
| IMPHY014988 | Limonene | CC1=CCC(CC1)C(=C)C | 22311 |
| IMPHY015022 | Nerolidol | C=CC(CC/C=C(/CCC=C(C)C)C)(O)C | 5284507 |
| IMPHY017919 | Ar-dihydro-turmerone | CC(CC(=O)C[C@@H](c1ccc(cc1)C)C)C | 13970960 |
| IMPHY000084 | 2-Heptanol | CCCCCC(O)C | 10976 |
| IMPHY000099 | Myrtenol | OCC1=CCC2CC1C2(C)C | 10582 |
| IMPHY000399 | beta-Bisabolene | CC(=CCCC(=C)[C@H]1CCC(=CC1)C)C | 10104370 |
| IMPHY001246 | Carvacrol | CC(c1ccc(c(c1)O)C)C | 10364 |
| IMPHY001351 | Elemicin | C=CCc1cc(OC)c(c(c1)OC)OC | 10248 |
| IMPHY001553 | Cinnamyl cinnamate | O=C(/C=C/c1ccccc1)OC/C=C/c1ccccc1 | 1550890 |
| IMPHY002533 | (-)-beta-Curcumene | CC(=CCC[C@H](C1=CCC(=CC1)C)C)C | 14014430 |
| IMPHY002825 | 2-(4-Methylphenyl)propan-2-ol | Cc1ccc(cc1)C(O)(C)C | 14529 |
| IMPHY002851 | 2-Decanol | CCCCCCCCC(O)C | 14254 |
| IMPHY003104 | Decanoic acid | CCCCCCCCCC(=O)O | 2969 |
| IMPHY003485 | Myrcene | C=CC(=C)CCC=C(C)C | 31253 |
| IMPHY003536 | Eugenol | C=CCc1ccc(c(c1)OC)O | 3314 |
| IMPHY003567 | alpha-Fenchene | C=C1CC2C(C1CC2)(C)C | 28930 |
| IMPHY003789 | Furanodienon | C/C/1=C/C(=O)c2c(C/C(=CCC1)/C)occ2C | 6442374 |
| IMPHY003840 | (E)-Tagetenone | C=C/C(=C/C(=O)C=C(C)C)/C | 6428431 |
| IMPHY003982 | gamma-Terpinene | CC1=CCC(=CC1)C(C)C | 7461 |
| IMPHY004121 | 2-Heptanone | CCCCCC(=O)C | 8051 |
| IMPHY004151 | Geranyl formate | O=COC/C=C(/CCC=C(C)C)C | 5282109 |
| IMPHY004438 | Geranyl butyrate | CCCC(=O)OC/C=C(/CCC=C(C)C)C | 5355856 |
| IMPHY004549 | Safrole | C=CCc1ccc2c(c1)OCO2 | 5144 |
| IMPHY005042 | Bisacumol | CC(=CC(CC(c1ccc(cc1)C)C)O)C | 5315469 |
| IMPHY005521 | 1-Hexen-3-OL | CCCC(C=C)O | 20928 |
| IMPHY005618 | Germacrene B | C/C/1=CCC/C(=C/CC(=C(C)C)CC1)/C | 5281519 |
| IMPHY005653 | 2-Octanol | CCCCCCC(O)C | 20083 |
| IMPHY005798 | Curlone | CC(C1CCC(=C)C=C1)CC(=O)C=C(C)C | 196216 |
| IMPHY006145 | p-Cymene | Cc1ccc(cc1)C(C)C | 7463 |
| IMPHY006214 | Germacr-1(10)-ene-5,8-dione | C/C/1=CCC[C@H](C)C(=O)C[C@H](C(=O)C1)C(C)C | 6441391 |
| IMPHY006324 | Linalyl propionate | CCC(=O)OC(CCC=C(C)C)(C=C)C | 61098 |
| IMPHY006325 | Myrtenal | O=CC1=CCC2CC1C2(C)C | 61130 |
| IMPHY006463 | Germacrone | C/C/1=CCC(=C(C)C)C(=O)C/C(=C/CC1)/C | 6436348 |
| IMPHY006550 | Thymol | Cc1ccc(c(c1)O)C(C)C | 6989 |
| IMPHY006696 | Methyleugenol | C=CCc1ccc(c(c1)OC)OC | 7127 |
| IMPHY006950 | Tricyclene | CC12C3C1CC(C2(C)C)C3 | 79035 |
| IMPHY007067 | Linalyl acetate | C=CC(OC(=O)C)(CCC=C(C)C)C | 8294 |
| IMPHY007069 | 4'-Methylacetophenone | Cc1ccc(cc1)C(=O)C | 8500 |
| IMPHY007110 | Perilla ketone | CC(CCC(=O)c1cocc1)C | 68381 |
| IMPHY008150 | 1-Methyl-4-(prop-1-en-2-yl)benzene | Cc1ccc(cc1)C(=C)C | 62385 |
| IMPHY008936 | alpha-Guaiene | CC(=C)[C@@H]1CC[C@@H](C2=C(C1)[C@@H](C)CC2)C | 5317844 |
| IMPHY009626 | 2-Nonanol | CCCCCCCC(O)C | 12367 |
| IMPHY009642 | 2-Nonanone | CCCCCCCC(=O)C | 13187 |
| IMPHY009713 | (Z)-Methyl jasmonate | CC/C=CC[C@H]1C(=O)CC[C@@H]1C(=O)OC | 6430765 |
| IMPHY009732 | cis-Carvotanacetol | CC([C@H]1CC=C([C@H](C1)O)C)C | 12233170 |
| IMPHY009740 | Camphene hydrate | CC1(C)C2CCC(C1(C)O)C2 | 101680 |
| IMPHY009780 | Heptyl salicylate | CCCCCCCOC(=O)c1ccccc1O | 80420 |
| IMPHY009858 | 1-Undecanol | CCCCCCCCCCCO | 8184 |
| IMPHY009866 | 2-(4-Methylphenyl)propan-1-ol | OCC(c1ccc(cc1)C)C | 95376 |
| IMPHY009998 | Geranyl hexanoate | CCCCCC(=O)OC/C=C(/CCC=C(C)C)C | 5365992 |
| IMPHY010072 | Eucalyptol | CC12CCC(CC1)C(O2)(C)C | 2758 |
| IMPHY010080 | beta-Elemene | C=C[C@]1(C)CC[C@H](C[C@H]1C(=C)C)C(=C)C | 6918391 |
| IMPHY010541 | 1-Bisabolone | CC(=CCC[C@@H]([C@H]1CCC(=CC1=O)C)C)C | 10421034 |
| IMPHY010611 | Curzerene | C=CC1(C)Cc2occ(c2CC1C(=C)C)C | 572766 |
| IMPHY010677 | Curcuphenol | CC(=CCC[C@H](c1ccc(cc1O)C)C)C | 360253 |
| IMPHY010887 | Thymol acetate | CC(=O)Oc1cc(C)ccc1C(C)C | 68252 |
| IMPHY010995 | Toluene | Cc1ccccc1 | 1140 |
| IMPHY011004 | p-Mentha-1,3,8-triene | CC1=CC=C(CC1)C(=C)C | 176983 |
| IMPHY011050 | 3-Buten-2-OL | CC(C=C)O | 11716 |
| IMPHY011061 | 5-Hydroxy-p-menth-6-en-2-one | CC(C1CC(=O)C(=CC1O)C)C | 14106048 |
| IMPHY011087 | alpha-Turmerone | CC(=CC(=O)CC(C1CC=C(C=C1)C)C)C | 14632996 |
| IMPHY011215 | Tetradecane | CCCCCCCCCCCCCC | 12389 |
| IMPHY011392 | 3-Carene | CC1=CCC2C(C1)C2(C)C | 26049 |
| IMPHY011396 | 4-Carvomenthenol | CC1=CCC(CC1)(O)C(C)C | 11230 |
| IMPHY011521 | 2-Undecanone | CCCCCCCCCC(=O)C | 8163 |
| IMPHY011542 | beta-Eudesmol | C=C1CCC[C@]2([C@H]1C[C@@H](CC2)C(O)(C)C)C | 91457 |
| IMPHY011552 | (1R)-2-methyl-5-propan-2-ylbicyclo[3.1.0]hex-2-ene | CC1=CCC2([C@@H]1C2)C(C)C | 6451618 |
| IMPHY011557 | 4-Isopropylbenzyl alcohol | OCc1ccc(cc1)C(C)C | 325 |
| IMPHY011563 | Curzerenone | C=C[C@@]1(C)Cc2occ(c2C(=O)[C@@H]1C(=C)C)C | 3081930 |
| IMPHY011581 | alpha-Selinene | CC1=CCC[C@]2([C@H]1C[C@@H](CC2)C(=C)C)C | 10856614 |
| IMPHY011586 | (S,1Z,6Z)-8-Isopropyl-1-methyl-5-methylenecyclodeca-1,6-diene | C/C/1=C/CCC(=C)/C=C[C@@H](CC1)C(C)C | 91723653 |
| IMPHY011588 | cis-3-Hexen-1-ol | OCC/C=CCC | 5281167 |
| IMPHY011590 | d-Borneol | O[C@@H]1C[C@H]2C([C@@]1(C)CC2)(C)C | 61060 |
| IMPHY011599 | Terpinolene | CC1=CCC(=C(C)C)CC1 | 11463 |
| IMPHY011632 | Farnesol | OC/C=C(/CC/C=C(/CCC=C(C)C)C)C | 445070 |
| IMPHY011643 | alpha-Terpinene | CC1=CC=C(CC1)C(C)C | 7462 |
| IMPHY011647 | Geranyl acetate | C/C(=CCOC(=O)C)/CCC=C(C)C | 1549026 |
| IMPHY011657 | cis-beta-Farnesene | C=CC(=C)CC/C=C(CCC=C(C)C)/C | 5317319 |
| IMPHY011658 | beta-Farnesene | C=CC(=C)CC/C=C(/CCC=C(C)C)C | 5281517 |
| IMPHY011659 | alpha-Muurolene | CC1=C[C@@H]2[C@H](CC1)C(=CC[C@H]2C(C)C)C | 12306047 |
| IMPHY011660 | (+)-alpha-Cadinene | CC1=C[C@@H]2[C@@H](CC1)C(=CC[C@H]2C(C)C)C | 12306048 |
| IMPHY011745 | Zingiberene | CC(=CCC[C@@H]([C@H]1CC=C(C=C1)C)C)C | 92776 |
| IMPHY011749 | Humulene epoxide II | C/C/1=CCC(C)(C)/C=C/C[C@@]2([C@@H](CC1)O2)C | 10704181 |
| IMPHY011761 | Humulene | C/C/1=CCC(C)(C)/C=C/C/C(=C/CC1)/C | 5281520 |
| IMPHY011789 | Citral | O=C/C=C(/CCC=C(C)C)C | 638011 |
| IMPHY011790 | Neral | O=C/C=C(CCC=C(C)C)/C | 643779 |
| IMPHY011804 | cis-3-Hexenyl acetate | CC/C=CCCOC(=O)C | 5363388 |
| IMPHY011817 | alpha-Farnesene | C=C/C(=C/C/C=C(/CCC=C(C)C)C)/C | 5281516 |
| IMPHY011838 | (E)-gamma-Bisabolene | CC(=CCC/C(=C/1CCC(=CC1)C)/C)C | 5352437 |
| IMPHY011882 | Cinnamaldehyde | O=C/C=C/c1ccccc1 | 637511 |
| IMPHY011939 | 10-epi-gamma-Eudesmol | CC1=C2C[C@@H](CC[C@@]2(CCC1)C)C(O)(C)C | 6430754 |
| IMPHY011965 | (+)-beta-Phellandrene | CC([C@@H]1CCC(=C)C=C1)C | 442484 |
| IMPHY011973 | (-)-cis-Carveol | CC(=C)[C@@H]1CC=C([C@@H](C1)O)C | 330573 |
| IMPHY012036 | Camphor | O=C1CC2C(C1(C)CC2)(C)C | 2537 |
| IMPHY012058 | Linalool | C=CC(CCC=C(C)C)(O)C | 6549 |
| IMPHY012061 | alpha-Pinene | CC1=CCC2CC1C2(C)C | 6654 |
| IMPHY012075 | Carvone | CC(=C)C1CC=C(C(=O)C1)C | 7439 |
| IMPHY012147 | beta-Pinene | C=C1CCC2CC1C2(C)C | 14896 |
| IMPHY012152 | alpha-Fenchol | O[C@H]1[C@@]2(C)CC[C@@H](C1(C)C)C2 | 439711 |
| IMPHY012160 | alpha-Terpineol | CC1=CCC(CC1)C(O)(C)C | 17100 |
| IMPHY012165 | Sabinene | C=C1CCC2(C1C2)C(C)C | 18818 |
| IMPHY012261 | alpha-Bergamotene | CC(=CCCC1(C)C2CC=C(C1C2)C)C | 86608 |
| IMPHY012279 | alpha-Curcumene | CC(=CCCC(c1ccc(cc1)C)C)C | 92139 |
| IMPHY012305 | beta-Patchoulene | CC1CCC2=C1CC1CCC2(C1(C)C)C | 101731 |
| IMPHY012381 | ar-Turmerone | CC(=CC(=O)C[C@@H](c1ccc(cc1)C)C)C | 160512 |
| IMPHY012587 | tau-Cadinol | CC1=C[C@H]2[C@H](CC1)[C@](C)(O)CC[C@H]2C(C)C | 12302222 |
| IMPHY012589 | 3-(1,5-Dimethyl-4-hexenyl)-6-methylene-1-cyclohexene | CC(C1CCC(=C)C=C1)CCC=C(C)C | 519764 |
| IMPHY012665 | Levomenol | CC(=CCC[C@@]([C@H]1CCC(=CC1)C)(O)C)C | 442343 |
| IMPHY012667 | Caryophyllene oxide | C=C1CC[C@H]2O[C@@]2(CC[C@@H]2[C@@H]1CC2(C)C)C | 1742210 |
| IMPHY012739 | (Z)-beta-Ocimene | C=C/C(=CCC=C(C)C)/C | 5320250 |
| IMPHY012907 | trans-Sesquisabinene hydrate | CC(=CCCC([C@]12CC[C@](C2C1)(C)O)C)C | 6428444 |
| IMPHY012920 | 2-Furanmethanol, 5-ethenyltetrahydro-alpha,alpha,5-trimethyl-, cis- | C=C[C@@]1(C)CC[C@H](O1)C(O)(C)C | 11116492 |
| IMPHY012921 | gamma-Elemene | C=C[C@]1(C)CCC(=C(C)C)C[C@H]1C(=C)C | 6432312 |
| IMPHY013019 | 3-Hydroxy-4,5,6-trimethoxyspiro[1H-indole-2(3H),1'(3'H)-[2H]isoindole]-3'-one | COc1c(OC)cc2c(c1OC)C(O)C1(N2)NC(=O)c2c1cccc2 | 10545230 |
| IMPHY013093 | delta-Elemene | C=C[C@@]1(C)CCC(=C[C@@H]1C(=C)C)C(C)C | 12309449 |
| IMPHY014811 | alpha-Phellandrene | CC1=CCC(C=C1)C(C)C | 7460 |
| IMPHY014831 | beta-Caryophyllene | C/C/1=CCCC(=C)[C@@H]2[C@@H](CC1)C(C2)(C)C | 5281515 |
| IMPHY014835 | (E)-beta-ocimene | C=C/C(=C/CC=C(C)C)/C | 5281553 |
| IMPHY014852 | Camphene | C=C1C2CCC(C1(C)C)C2 | 6616 |
| IMPHY014863 | cis-alpha-Bergamotene | CC(=CCCC1(C)[C@@H]2CC=C([C@H]1C2)C)C | 91753502 |
| IMPHY014866 | 2-Cyclohexen-1-ol, 2-methyl-5-(1-methylethenyl)-, acetate, cis- | CC(=O)O[C@@H]1C[C@@H](CC=C1C)C(=C)C | 102024 |
| IMPHY014871 | cis-Nerolidol | C=CC(CC/C=C(CCC=C(C)C)/C)(O)C | 5320128 |
| IMPHY014876 | 7-epi-cis-Sesquisabinene hydrate | CC(=CCCC([C@]12CC[C@@](C2C1)(C)O)C)C | 6428435 |
| IMPHY014906 | Cedrelanol | CC1=C[C@@H]2[C@@H](CC1)[C@@](C)(O)CC[C@H]2C(C)C | 160799 |
| IMPHY014907 | 6-Epi-beta-bisabolol | CC(=CCC[C@@H]([C@@]1(O)CCC(=CC1)C)C)C | 12300148 |
| IMPHY014923 | Geraniol | OC/C=C(/CCC=C(C)C)C | 637566 |
| IMPHY014988 | Limonene | CC1=CCC(CC1)C(=C)C | 22311 |
| IMPHY014989 | trans-Linalool oxide | C=C[C@]1(C)CC[C@H](O1)C(O)(C)C | 6432254 |
| IMPHY015022 | Nerolidol | C=CC(CC/C=C(/CCC=C(C)C)C)(O)C | 5284507 |
| IMPHY015042 | Piperitone | CC1=CC(=O)C(CC1)C(C)C | 6987 |
| IMPHY015062 | (+)-cis-Sabinol | CC([C@]12C[C@@H]2C(=C)[C@@H](C1)O)C | 94147 |
| IMPHY015063 | Sabinyl acetate | CC(=O)OC1CC2(C(C1=C)C2)C(C)C | 94266 |
| IMPHY015095 | 2-Cyclohexen-1-ol, 1-methyl-4-(1-methylethyl)-, trans- | CC([C@@H]1CC[C@@](C=C1)(C)O)C | 122484 |
| IMPHY016014 | Isobornyl acetate | CC(=O)O[C@H]1C[C@@H]2C([C@]1(C)CC2)(C)C | 247573 |
| IMPHY016053 | Viridiflorol | C[C@@H]1CC[C@H]2[C@@H]1[C@H]1[C@H](C1(C)C)CC[C@]2(C)O | 11996452 |
| IMPHY017919 | Ar-dihydro-turmerone | CC(CC(=O)C[C@@H](c1ccc(cc1)C)C)C | 13970960 |
| IMPHY000073 | 2-Hepten-4-one, 6-(2-hydroxy-4-methylphenyl)-2-methyl- | CC(=CC(=O)CC(c1ccc(cc1O)C)C)C | 10955433 |
| IMPHY000084 | 2-Heptanol | CCCCCC(O)C | 10976 |
| IMPHY000099 | Myrtenol | OCC1=CCC2CC1C2(C)C | 10582 |
| IMPHY000399 | beta-Bisabolene | CC(=CCCC(=C)[C@H]1CCC(=CC1)C)C | 10104370 |
| IMPHY000545 | O-Cymene | CC(c1ccccc1C)C | 10703 |
| IMPHY001107 | (1E)-1,7-bis(4-hydroxy-3-methoxyphenyl)hept-1-ene-3,5-dione | COc1cc(CCC(=O)CC(=O)/C=C/c2ccc(c(c2)OC)O)ccc1O | 10429233 |
| IMPHY001246 | Carvacrol | CC(c1ccc(c(c1)O)C)C | 10364 |
| IMPHY001351 | Elemicin | C=CCc1cc(OC)c(c(c1)OC)OC | 10248 |
| IMPHY001430 | Bicyclo(4.1.0)heptan-3-one, 1-methyl-4-(1-methylethylidene)-7-(3-oxobutyl)-, (1S,6R,7R)- | CC(=O)CC[C@@H]1[C@@H]2[C@@]1(C)CC(=O)C(=C(C)C)C2 | 153845 |
| IMPHY001538 | 2-Undecanol | CCCCCCCCCC(O)C | 15448 |
| IMPHY001553 | Cinnamyl cinnamate | O=C(/C=C/c1ccccc1)OC/C=C/c1ccccc1 | 1550890 |
| IMPHY001611 | Turmeronol A | CC(=CC(=O)CC(c1ccc(c(c1)O)C)C)C | 15858385 |
| IMPHY001816 | gamma-Terpineol | CC(=C1CCC(CC1)(C)O)C | 11467 |
| IMPHY002179 | Humuladienone | C/C/1=C/CC(C)(C)/C=CCC(C(=O)CC1)C | 101297706 |
| IMPHY002533 | (-)-beta-Curcumene | CC(=CCC[C@H](C1=CCC(=CC1)C)C)C | 14014430 |
| IMPHY002581 | Procurcumadiol | CC1=CC(=O)C(=C(C)C)CC2(C1CCC2(C)O)O | 14633011 |
| IMPHY002626 | (6Z)-6,10-dimethyl-3-propan-2-ylidenecyclodec-6-ene-1,4-dione | C/C/1=C/CCC(C)C(=O)CC(=C(C)C)C(=O)C1 | 14191392 |
| IMPHY002825 | 2-(4-Methylphenyl)propan-2-ol | Cc1ccc(cc1)C(O)(C)C | 14529 |
| IMPHY002850 | Curcumol | CC([C@@H]1C[C@]23O[C@]1(O)CC(=C)[C@@H]3CC[C@@H]2C)C | 14240392 |
| IMPHY002851 | 2-Decanol | CCCCCCCCC(O)C | 14254 |
| IMPHY002893 | Bisacurone | CC(=CC(=O)C[C@@H]([C@@H]1C=C[C@]([C@H](C1)O)(C)O)C)C | 14287397 |
| IMPHY002913 | Sesquisabinene | CC(=CCCC(C12CCC(=C)C2C1)C)C | 25202482 |
| IMPHY003104 | Decanoic acid | CCCCCCCCCC(=O)O | 2969 |
| IMPHY003485 | Myrcene | C=CC(=C)CCC=C(C)C | 31253 |
| IMPHY003536 | Eugenol | C=CCc1ccc(c(c1)OC)O | 3314 |
| IMPHY003537 | Tetradecanal | CCCCCCCCCCCCCC=O | 31291 |
| IMPHY003567 | alpha-Fenchene | C=C1CC2C(C1CC2)(C)C | 28930 |
| IMPHY003768 | 4,10-Epizedoarondiol | CC(=C1C[C@H]2[C@H]([C@@](CC1=O)(C)O)CC[C@@]2(C)O)C | 24834047 |
| IMPHY003789 | Furanodienon | C/C/1=C/C(=O)c2c(C/C(=CCC1)/C)occ2C | 6442374 |
| IMPHY003982 | gamma-Terpinene | CC1=CCC(=CC1)C(C)C | 7461 |
| IMPHY004121 | 2-Heptanone | CCCCCC(=O)C | 8051 |
| IMPHY004129 | Demethoxycurcumin | COc1cc(/C=C/C(=O)CC(=O)/C=C/c2ccc(cc2)O)ccc1O | 5469424 |
| IMPHY004151 | Geranyl formate | O=COC/C=C(/CCC=C(C)C)C | 5282109 |
| IMPHY004192 | Piperine | O=C(N1CCCCC1)/C=C/C=C/c1ccc2c(c1)OCO2 | 638024 |
| IMPHY004288 | Epiprocurcumenol | CC1=CC(=O)C(=C(C)C)C[C@H]2[C@@H]1CC[C@]2(C)O | 10263440 |
| IMPHY004315 | Procurcumenol | CC1=CC(=O)C(=C(C)C)C[C@H]2[C@H]1CC[C@]2(C)O | 189061 |
| IMPHY004438 | Geranyl butyrate | CCCC(=O)OC/C=C(/CCC=C(C)C)C | 5355856 |
| IMPHY004549 | Safrole | C=CCc1ccc2c(c1)OCO2 | 5144 |
| IMPHY004666 | Bisabolane | CC(CCCC(C1CCC(CC1)C)C)C | 520453 |
| IMPHY004928 | Bisdemethoxycurcumin | O=C(CC(=O)/C=C/c1ccc(cc1)O)/C=C/c1ccc(cc1)O | 5315472 |
| IMPHY005042 | Bisacumol | CC(=CC(CC(c1ccc(cc1)C)C)O)C | 5315469 |
| IMPHY005521 | 1-Hexen-3-OL | CCCC(C=C)O | 20928 |
| IMPHY005618 | Germacrene B | C/C/1=CCC/C(=C/CC(=C(C)C)CC1)/C | 5281519 |
| IMPHY005653 | 2-Octanol | CCCCCCC(O)C | 20083 |
| IMPHY005798 | Curlone | CC(C1CCC(=C)C=C1)CC(=O)C=C(C)C | 196216 |
| IMPHY006145 | p-Cymene | Cc1ccc(cc1)C(C)C | 7463 |
| IMPHY006214 | Germacr-1(10)-ene-5,8-dione | C/C/1=CCC[C@H](C)C(=O)C[C@H](C(=O)C1)C(C)C | 6441391 |
| IMPHY006300 | Cholesterol | CC(CCC[C@H]([C@H]1CC[C@@H]2[C@]1(C)CC[C@H]1[C@H]2CC=C2[C@]1(C)CC[C@@H](C2)O)C)C | 5997 |
| IMPHY006324 | Linalyl propionate | CCC(=O)OC(CCC=C(C)C)(C=C)C | 61098 |
| IMPHY006325 | Myrtenal | O=CC1=CCC2CC1C2(C)C | 61130 |
| IMPHY006463 | Germacrone | C/C/1=CCC(=C(C)C)C(=O)C/C(=C/CC1)/C | 6436348 |
| IMPHY006549 | Cyclocurcumin | COc1cc(/C=C/C2=CC(=O)CC(O2)c2ccc(c(c2)OC)O)ccc1O | 69879809 |
| IMPHY006550 | Thymol | Cc1ccc(c(c1)O)C(C)C | 6989 |
| IMPHY006696 | Methyleugenol | C=CCc1ccc(c(c1)OC)OC | 7127 |
| IMPHY006758 | (1S,2S,5S,8S)-2-methyl-6-methylidene-9-propan-2-ylidene-11-oxatricyclo[6.2.1.01,5]undecan-8-ol | CC(=C1C[C@]23O[C@@]1(O)CC(=C)[C@@H]3CC[C@@H]2C)C | 12310886 |
| IMPHY006933 | 2-(Hydroxymethyl)anthraquinone | OCc1ccc2c(c1)C(=O)c1c(C2=O)cccc1 | 87014 |
| IMPHY006950 | Tricyclene | CC12C3C1CC(C2(C)C)C3 | 79035 |
| IMPHY007067 | Linalyl acetate | C=CC(OC(=O)C)(CCC=C(C)C)C | 8294 |
| IMPHY007069 | 4'-Methylacetophenone | Cc1ccc(cc1)C(=O)C | 8500 |
| IMPHY007110 | Perilla ketone | CC(CCC(=O)c1cocc1)C | 68381 |
| IMPHY007427 | Xanthorrhizol | CC(=CCC[C@H](c1ccc(c(c1)O)C)C)C | 93135 |
| IMPHY007574 | Curcumin | COc1cc(/C=C/C(=O)CC(=O)/C=C/c2ccc(c(c2)OC)O)ccc1O | 969516 |
| IMPHY008150 | 1-Methyl-4-(prop-1-en-2-yl)benzene | Cc1ccc(cc1)C(=C)C | 62385 |
| IMPHY008383 | Linalyl isobutyrate | C=CC(OC(=O)C(C)C)(CCC=C(C)C)C | 6532 |
| IMPHY008936 | alpha-Guaiene | CC(=C)[C@@H]1CC[C@@H](C2=C(C1)[C@@H](C)CC2)C | 5317844 |
| IMPHY009419 | Tridecane | CCCCCCCCCCCCC | 12388 |
| IMPHY009626 | 2-Nonanol | CCCCCCCC(O)C | 12367 |
| IMPHY009688 | 3,7(11)-Eudesmadiene | CC1=CCCC2(C1CC(=C(C)C)CC2)C | 522296 |
| IMPHY009706 | Isoledene | CC1CCC2C(C3=C1CCC3C)C2(C)C | 530426 |
| IMPHY009732 | cis-Carvotanacetol | CC([C@H]1CC=C([C@H](C1)O)C)C | 12233170 |
| IMPHY009740 | Camphene hydrate | CC1(C)C2CCC(C1(C)O)C2 | 101680 |
| IMPHY009780 | Heptyl salicylate | CCCCCCCOC(=O)c1ccccc1O | 80420 |
| IMPHY009858 | 1-Undecanol | CCCCCCCCCCCO | 8184 |
| IMPHY009909 | Ethene;2-methoxyphenol;phenol;propanedial | Oc1ccccc1.COc1ccccc1O.O=CCC=O.C=C.C=C | 122130015 |
| IMPHY009998 | Geranyl hexanoate | CCCCCC(=O)OC/C=C(/CCC=C(C)C)C | 5365992 |
| IMPHY010072 | Eucalyptol | CC12CCC(CC1)C(O2)(C)C | 2758 |
| IMPHY010080 | beta-Elemene | C=C[C@]1(C)CC[C@H](C[C@H]1C(=C)C)C(=C)C | 6918391 |
| IMPHY010541 | 1-Bisabolone | CC(=CCC[C@@H]([C@H]1CCC(=CC1=O)C)C)C | 10421034 |
| IMPHY010611 | Curzerene | C=CC1(C)Cc2occ(c2CC1C(=C)C)C | 572766 |
| IMPHY010676 | (+)-Curcumenol | CC(=C1C[C@]23O[C@]1(O)C=C([C@@H]3CC[C@@H]2C)C)C | 167812 |
| IMPHY010677 | Curcuphenol | CC(=CCC[C@H](c1ccc(cc1O)C)C)C | 360253 |
| IMPHY010887 | Thymol acetate | CC(=O)Oc1cc(C)ccc1C(C)C | 68252 |
| IMPHY010995 | Toluene | Cc1ccccc1 | 1140 |
| IMPHY011004 | p-Mentha-1,3,8-triene | CC1=CC=C(CC1)C(=C)C | 176983 |
| IMPHY011027 | Turmerol | CC(=CC(CC(C1=CCC(=CC1)C)C)O)C | 20055538 |
| IMPHY011050 | 3-Buten-2-OL | CC(C=C)O | 11716 |
| IMPHY011087 | alpha-Turmerone | CC(=CC(=O)CC(C1CC=C(C=C1)C)C)C | 14632996 |
| IMPHY011215 | Tetradecane | CCCCCCCCCCCCCC | 12389 |
| IMPHY011392 | 3-Carene | CC1=CCC2C(C1)C2(C)C | 26049 |
| IMPHY011396 | 4-Carvomenthenol | CC1=CCC(CC1)(O)C(C)C | 11230 |
| IMPHY011521 | 2-Undecanone | CCCCCCCCCC(=O)C | 8163 |
| IMPHY011542 | beta-Eudesmol | C=C1CCC[C@]2([C@H]1C[C@@H](CC2)C(O)(C)C)C | 91457 |
| IMPHY011552 | (1R)-2-methyl-5-propan-2-ylbicyclo[3.1.0]hex-2-ene | CC1=CCC2([C@@H]1C2)C(C)C | 6451618 |
| IMPHY011557 | 4-Isopropylbenzyl alcohol | OCc1ccc(cc1)C(C)C | 325 |
| IMPHY011563 | Curzerenone | C=C[C@@]1(C)Cc2occ(c2C(=O)[C@@H]1C(=C)C)C | 3081930 |
| IMPHY011581 | alpha-Selinene | CC1=CCC[C@]2([C@H]1C[C@@H](CC2)C(=C)C)C | 10856614 |
| IMPHY011586 | (S,1Z,6Z)-8-Isopropyl-1-methyl-5-methylenecyclodeca-1,6-diene | C/C/1=C/CCC(=C)/C=C[C@@H](CC1)C(C)C | 91723653 |
| IMPHY011588 | cis-3-Hexen-1-ol | OCC/C=CCC | 5281167 |
| IMPHY011590 | d-Borneol | O[C@@H]1C[C@H]2C([C@@]1(C)CC2)(C)C | 61060 |
| IMPHY011599 | Terpinolene | CC1=CCC(=C(C)C)CC1 | 11463 |
| IMPHY011632 | Farnesol | OC/C=C(/CC/C=C(/CCC=C(C)C)C)C | 445070 |
| IMPHY011643 | alpha-Terpinene | CC1=CC=C(CC1)C(C)C | 7462 |
| IMPHY011647 | Geranyl acetate | C/C(=CCOC(=O)C)/CCC=C(C)C | 1549026 |
| IMPHY011657 | cis-beta-Farnesene | C=CC(=C)CC/C=C(CCC=C(C)C)/C | 5317319 |
| IMPHY011658 | beta-Farnesene | C=CC(=C)CC/C=C(/CCC=C(C)C)C | 5281517 |
| IMPHY011659 | alpha-Muurolene | CC1=C[C@@H]2[C@H](CC1)C(=CC[C@H]2C(C)C)C | 12306047 |
| IMPHY011660 | (+)-alpha-Cadinene | CC1=C[C@@H]2[C@@H](CC1)C(=CC[C@H]2C(C)C)C | 12306048 |
| IMPHY011744 | delta-Curcumene | CC(=CCC[C@@H](C1CC=C(C=C1)C)C)C | 57386731 |
| IMPHY011745 | Zingiberene | CC(=CCC[C@@H]([C@H]1CC=C(C=C1)C)C)C | 92776 |
| IMPHY011749 | Humulene epoxide II | C/C/1=CCC(C)(C)/C=C/C[C@@]2([C@@H](CC1)O2)C | 10704181 |
| IMPHY011761 | Humulene | C/C/1=CCC(C)(C)/C=C/C/C(=C/CC1)/C | 5281520 |
| IMPHY011763 | Anethole | C/C=C/c1ccc(cc1)OC | 637563 |
| IMPHY011789 | Citral | O=C/C=C(/CCC=C(C)C)C | 638011 |
| IMPHY011790 | Neral | O=C/C=C(CCC=C(C)C)/C | 643779 |
| IMPHY011884 | Pulegone | C[C@@H]1CCC(=C(C)C)C(=O)C1 | 442495 |
| IMPHY011939 | 10-epi-gamma-Eudesmol | CC1=C2C[C@@H](CC[C@@]2(CCC1)C)C(O)(C)C | 6430754 |
| IMPHY011957 | (+)-delta-Cadinene | CC1=C[C@@H]2C(=C(C)CC[C@H]2C(C)C)CC1 | 441005 |
| IMPHY011965 | (+)-beta-Phellandrene | CC([C@@H]1CCC(=C)C=C1)C | 442484 |
| IMPHY011973 | (-)-cis-Carveol | CC(=C)[C@@H]1CC=C([C@@H](C1)O)C | 330573 |
| IMPHY012036 | Camphor | O=C1CC2C(C1(C)CC2)(C)C | 2537 |
| IMPHY012058 | Linalool | C=CC(CCC=C(C)C)(O)C | 6549 |
| IMPHY012061 | alpha-Pinene | CC1=CCC2CC1C2(C)C | 6654 |
| IMPHY012075 | Carvone | CC(=C)C1CC=C(C(=O)C1)C | 7439 |
| IMPHY012147 | beta-Pinene | C=C1CCC2CC1C2(C)C | 14896 |
| IMPHY012152 | alpha-Fenchol | O[C@H]1[C@@]2(C)CC[C@@H](C1(C)C)C2 | 439711 |
| IMPHY012160 | alpha-Terpineol | CC1=CCC(CC1)C(O)(C)C | 17100 |
| IMPHY012165 | Sabinene | C=C1CCC2(C1C2)C(C)C | 18818 |
| IMPHY012279 | alpha-Curcumene | CC(=CCCC(c1ccc(cc1)C)C)C | 92139 |
| IMPHY012381 | ar-Turmerone | CC(=CC(=O)C[C@@H](c1ccc(cc1)C)C)C | 160512 |
| IMPHY012402 | Campesterol | O[C@H]1CC[C@]2(C(=CC[C@@H]3[C@@H]2CC[C@]2([C@H]3CC[C@@H]2[C@@H](CC[C@H](C(C)C)C)C)C)C1)C | 173183 |
| IMPHY012587 | tau-Cadinol | CC1=C[C@H]2[C@H](CC1)[C@](C)(O)CC[C@H]2C(C)C | 12302222 |
| IMPHY012589 | 3-(1,5-Dimethyl-4-hexenyl)-6-methylene-1-cyclohexene | CC(C1CCC(=C)C=C1)CCC=C(C)C | 519764 |
| IMPHY012665 | Levomenol | CC(=CCC[C@@]([C@H]1CCC(=CC1)C)(O)C)C | 442343 |
| IMPHY012667 | Caryophyllene oxide | C=C1CC[C@H]2O[C@@]2(CC[C@@H]2[C@@H]1CC2(C)C)C | 1742210 |
| IMPHY012739 | (Z)-beta-Ocimene | C=C/C(=CCC=C(C)C)/C | 5320250 |
| IMPHY012907 | trans-Sesquisabinene hydrate | CC(=CCCC([C@]12CC[C@](C2C1)(C)O)C)C | 6428444 |
| IMPHY012920 | 2-Furanmethanol, 5-ethenyltetrahydro-alpha,alpha,5-trimethyl-, cis- | C=C[C@@]1(C)CC[C@H](O1)C(O)(C)C | 11116492 |
| IMPHY012921 | gamma-Elemene | C=C[C@]1(C)CCC(=C(C)C)C[C@H]1C(=C)C | 6432312 |
| IMPHY013093 | delta-Elemene | C=C[C@@]1(C)CCC(=C[C@@H]1C(=C)C)C(C)C | 12309449 |
| IMPHY013210 | Isoprocurcumenol | C=C1CC(=O)C(=C(C)C)C[C@H]2[C@H]1CC[C@]2(C)O | 14543198 |
| IMPHY014179 | (1E,4E)-1-(4-Hydroxy-3-methoxyphenyl)-5-(4-hydroxyphenyl)-1,4-pentadiene-3-one | COc1cc(/C=C/C(=O)/C=C/c2ccc(cc2)O)ccc1O | 10469828 |
| IMPHY014466 | 1,7-Bis(4-hydroxyphenyl)-1,4,6-heptatrien-3-one | O=C(C=Cc1ccc(cc1)O)C=CC=Cc1ccc(cc1)O | 71346280 |
| IMPHY014602 | Bisabola-3,10-diene-2-one | CC(=CCCC(C1CC=C(C(=O)C1)C)C)C | 101926857 |
| IMPHY014802 | alpha-Atlantone | CC(=CC(=O)/C=C([C@@H]1CCC(=CC1)C)/C)C | 12299867 |
| IMPHY014811 | alpha-Phellandrene | CC1=CCC(C=C1)C(C)C | 7460 |
| IMPHY014831 | beta-Caryophyllene | C/C/1=CCCC(=C)[C@@H]2[C@@H](CC1)C(C2)(C)C | 5281515 |
| IMPHY014833 | beta-Himachalene | CC1=C[C@H]2C(=C(C)CCCC2(C)C)CC1 | 11586487 |
| IMPHY014835 | (E)-beta-ocimene | C=C/C(=C/CC=C(C)C)/C | 5281553 |
| IMPHY014836 | beta-Sitosterol | CC[C@@H](C(C)C)CC[C@H]([C@H]1CC[C@@H]2[C@]1(C)CC[C@H]1[C@H]2CC=C2[C@]1(C)CC[C@@H](C2)O)C | 222284 |
| IMPHY014842 | Stigmasterol | CC[C@@H](C(C)C)/C=C/[C@H]([C@H]1CC[C@@H]2[C@]1(C)CC[C@H]1[C@H]2CC=C2[C@]1(C)CC[C@@H](C2)O)C | 5280794 |
| IMPHY014852 | Camphene | C=C1C2CCC(C1(C)C)C2 | 6616 |
| IMPHY014863 | cis-alpha-Bergamotene | CC(=CCCC1(C)[C@@H]2CC=C([C@H]1C2)C)C | 91753502 |
| IMPHY014866 | 2-Cyclohexen-1-ol, 2-methyl-5-(1-methylethenyl)-, acetate, cis- | CC(=O)O[C@@H]1C[C@@H](CC=C1C)C(=C)C | 102024 |
| IMPHY014876 | 7-epi-cis-Sesquisabinene hydrate | CC(=CCCC([C@]12CC[C@@](C2C1)(C)O)C)C | 6428435 |
| IMPHY014906 | Cedrelanol | CC1=C[C@@H]2[C@@H](CC1)[C@@](C)(O)CC[C@H]2C(C)C | 160799 |
| IMPHY014907 | 6-Epi-beta-bisabolol | CC(=CCC[C@@H]([C@@]1(O)CCC(=CC1)C)C)C | 12300148 |
| IMPHY014923 | Geraniol | OC/C=C(/CCC=C(C)C)C | 637566 |
| IMPHY014988 | Limonene | CC1=CCC(CC1)C(=C)C | 22311 |
| IMPHY014989 | trans-Linalool oxide | C=C[C@]1(C)CC[C@H](O1)C(O)(C)C | 6432254 |
| IMPHY015022 | Nerolidol | C=CC(CC/C=C(/CCC=C(C)C)C)(O)C | 5284507 |
| IMPHY015062 | (+)-cis-Sabinol | CC([C@]12C[C@@H]2C(=C)[C@@H](C1)O)C | 94147 |
| IMPHY015063 | Sabinyl acetate | CC(=O)OC1CC2(C(C1=C)C2)C(C)C | 94266 |
| IMPHY015095 | 2-Cyclohexen-1-ol, 1-methyl-4-(1-methylethyl)-, trans- | CC([C@@H]1CC[C@@](C=C1)(C)O)C | 122484 |
| IMPHY016012 | Allo-Aromadendrene | C[C@@H]1CC[C@H]2[C@@H]1C1C(C1(C)C)CCC2=C | 42608158 |
| IMPHY016014 | Isobornyl acetate | CC(=O)O[C@H]1C[C@@H]2C([C@]1(C)CC2)(C)C | 247573 |
| IMPHY016053 | Viridiflorol | C[C@@H]1CC[C@H]2[C@@H]1[C@H]1[C@H](C1(C)C)CC[C@]2(C)O | 11996452 |
| IMPHY016054 | trans-alpha-Bergamotene | CC(=CCC[C@]1(C)[C@H]2CC=C([C@@H]1C2)C)C | 6429302 |
| IMPHY017883 | (4S,5S)-Germacrone-4,5-epoxide | CC1=CCC[C@@]2(C)O[C@H]2CC(=C(C)C)C(=O)C1 | 91753231 |
| IMPHY017919 | Ar-dihydro-turmerone | CC(CC(=O)C[C@@H](c1ccc(cc1)C)C)C | 13970960 |
| IMPHY012589 | 3-(1,5-Dimethyl-4-hexenyl)-6-methylene-1-cyclohexene | CC(C1CCC(=C)C=C1)CCC=C(C)C | 519764 |
| IMPHY007574 | Curcumin | COc1cc(/C=C/C(=O)CC(=O)/C=C/c2ccc(c(c2)OC)O)ccc1O | 969516 |
| IMPHY000073 | 2-Hepten-4-one, 6-(2-hydroxy-4-methylphenyl)-2-methyl- | CC(=CC(=O)CC(c1ccc(cc1O)C)C)C | 10955433 |
| IMPHY001107 | (1E)-1,7-bis(4-hydroxy-3-methoxyphenyl)hept-1-ene-3,5-dione | COc1cc(CCC(=O)CC(=O)/C=C/c2ccc(c(c2)OC)O)ccc1O | 10429233 |
| IMPHY001430 | Bicyclo(4.1.0)heptan-3-one, 1-methyl-4-(1-methylethylidene)-7-(3-oxobutyl)-, (1S,6R,7R)- | CC(=O)CC[C@@H]1[C@@H]2[C@@]1(C)CC(=O)C(=C(C)C)C2 | 153845 |
| IMPHY001611 | Turmeronol A | CC(=CC(=O)CC(c1ccc(c(c1)O)C)C)C | 15858385 |
| IMPHY002581 | Procurcumadiol | CC1=CC(=O)C(=C(C)C)CC2(C1CCC2(C)O)O | 14633011 |
| IMPHY002626 | (6Z)-6,10-dimethyl-3-propan-2-ylidenecyclodec-6-ene-1,4-dione | C/C/1=C/CCC(C)C(=O)CC(=C(C)C)C(=O)C1 | 14191392 |
| IMPHY002893 | Bisacurone | CC(=CC(=O)C[C@@H]([C@@H]1C=C[C@]([C@H](C1)O)(C)O)C)C | 14287397 |
| IMPHY003392 | Butylated hydroxytoluene | Cc1cc(c(c(c1)C(C)(C)C)O)C(C)(C)C | 31404 |
| IMPHY003536 | Eugenol | C=CCc1ccc(c(c1)OC)O | 3314 |
| IMPHY003768 | 4,10-Epizedoarondiol | CC(=C1C[C@H]2[C@H]([C@@](CC1=O)(C)O)CC[C@@]2(C)O)C | 24834047 |
| IMPHY004129 | Demethoxycurcumin | COc1cc(/C=C/C(=O)CC(=O)/C=C/c2ccc(cc2)O)ccc1O | 5469424 |
| IMPHY004192 | Piperine | O=C(N1CCCCC1)/C=C/C=C/c1ccc2c(c1)OCO2 | 638024 |
| IMPHY004288 | Epiprocurcumenol | CC1=CC(=O)C(=C(C)C)C[C@H]2[C@@H]1CC[C@]2(C)O | 10263440 |
| IMPHY004315 | Procurcumenol | CC1=CC(=O)C(=C(C)C)C[C@H]2[C@H]1CC[C@]2(C)O | 189061 |
| IMPHY004928 | Bisdemethoxycurcumin | O=C(CC(=O)/C=C/c1ccc(cc1)O)/C=C/c1ccc(cc1)O | 5315472 |
| IMPHY005042 | Bisacumol | CC(=CC(CC(c1ccc(cc1)C)C)O)C | 5315469 |
| IMPHY005088 | 2-Methylisoborneol | CC12CCC(C2(C)C)CC1(C)O | 16913 |
| IMPHY005798 | Curlone | CC(C1CCC(=C)C=C1)CC(=O)C=C(C)C | 196216 |
| IMPHY006214 | Germacr-1(10)-ene-5,8-dione | C/C/1=CCC[C@H](C)C(=O)C[C@H](C(=O)C1)C(C)C | 6441391 |
| IMPHY006362 | Ascorbic acid | OC[C@@H]([C@H]1OC(=O)C(=C1O)O)O | 54670067 |
| IMPHY006549 | Cyclocurcumin | COc1cc(/C=C/C2=CC(=O)CC(O2)c2ccc(c(c2)OC)O)ccc1O | 69879809 |
| IMPHY006933 | 2-(Hydroxymethyl)anthraquinone | OCc1ccc2c(c1)C(=O)c1c(C2=O)cccc1 | 87014 |
| IMPHY007097 | (1E,4E)-1,5-bis(4-hydroxy-3-methoxyphenyl)penta-1,4-dien-3-one | COc1cc(/C=C/C(=O)/C=C/c2ccc(c(c2)OC)O)ccc1O | 6474893 |
| IMPHY007488 | Tetrahydrobisdemethoxydiferuloylmethane | O=C(CC(=O)CCc1ccc(cc1)O)CCc1ccc(cc1)O | 9796792 |
| IMPHY007574 | Curcumin | COc1cc(/C=C/C(=O)CC(=O)/C=C/c2ccc(c(c2)OC)O)ccc1O | 969516 |
| IMPHY008937 | Vitamin E | C[C@@H](CCC[C@]1(C)CCc2c(O1)c(C)c(c(c2C)O)C)CCC[C@@H](CCCC(C)C)C | 14985 |
| IMPHY010072 | Eucalyptol | CC12CCC(CC1)C(O2)(C)C | 2758 |
| IMPHY010541 | 1-Bisabolone | CC(=CCC[C@@H]([C@H]1CCC(=CC1=O)C)C)C | 10421034 |
| IMPHY010676 | (+)-Curcumenol | CC(=C1C[C@]23O[C@]1(O)C=C([C@@H]3CC[C@@H]2C)C)C | 167812 |
| IMPHY011087 | alpha-Turmerone | CC(=CC(=O)CC(C1CC=C(C=C1)C)C)C | 14632996 |
| IMPHY011563 | Curzerenone | C=C[C@@]1(C)Cc2occ(c2C(=O)[C@@H]1C(=C)C)C | 3081930 |
| IMPHY011590 | d-Borneol | O[C@@H]1C[C@H]2C([C@@]1(C)CC2)(C)C | 61060 |
| IMPHY011745 | Zingiberene | CC(=CCC[C@@H]([C@H]1CC=C(C=C1)C)C)C | 92776 |
| IMPHY012036 | Camphor | O=C1CC2C(C1(C)CC2)(C)C | 2537 |
| IMPHY012058 | Linalool | C=CC(CCC=C(C)C)(O)C | 6549 |
| IMPHY012061 | alpha-Pinene | CC1=CCC2CC1C2(C)C | 6654 |
| IMPHY012147 | beta-Pinene | C=C1CCC2CC1C2(C)C | 14896 |
| IMPHY012279 | alpha-Curcumene | CC(=CCCC(c1ccc(cc1)C)C)C | 92139 |
| IMPHY012381 | ar-Turmerone | CC(=CC(=O)C[C@@H](c1ccc(cc1)C)C)C | 160512 |
| IMPHY013073 | Oleoresin tumeric | O=C(CC(=O)/C=C/c1ccc(cc1)O)/C=C/c1ccc(cc1)O.COc1cc(/C=C/C(=O)CC(=O)/C=C/c2ccc(c(c2)OC)O)ccc1O.COc1cc(/C=C/C(=O)CC(=O)/C=C/c2ccc(cc2)O)ccc1O | 11979920 |
| IMPHY013210 | Isoprocurcumenol | C=C1CC(=O)C(=C(C)C)C[C@H]2[C@H]1CC[C@]2(C)O | 14543198 |
| IMPHY014831 | beta-Caryophyllene | C/C/1=CCCC(=C)[C@@H]2[C@@H](CC1)C(C2)(C)C | 5281515 |
| IMPHY014852 | Camphene | C=C1C2CCC(C1(C)C)C2 | 6616 |
| IMPHY014988 | Limonene | CC1=CCC(CC1)C(=C)C | 22311 |
|  |  |  |  |
|  |  |  |  |
|  |  |  |  |
|  |  |  |  |
|  |  |  |  |
| ***17. Plant name : Panax ginseng*** | | | |
| **IMPPAT Phytochemical identifier:** | **Phytochemical name:** | **SMILES:** | **CID** |
| IMPHY000147 | (20S)-Protopanaxadiol | CC(=CCC[C@@]([C@H]1CC[C@@]2([C@@H]1[C@H](O)C[C@H]1[C@@]2(C)CC[C@@H]2[C@]1(C)CC[C@@H](C2(C)C)O)C)(O)C)C | 11213350 |
| IMPHY005440 | Protopanaxatriol | CC(=CCC[C@@]([C@H]1CC[C@@]2([C@@H]1[C@H](O)C[C@H]1[C@@]2(C)C[C@@H]([C@@H]2[C@]1(C)CC[C@@H](C2(C)C)O)O)C)(O)C)C | 11468733 |
| IMPHY014754 | 20(R)-Protopanaxatriol | CC(=CCC[C@]([C@H]1CC[C@@]2([C@@H]1[C@H](O)C[C@H]1[C@@]2(C)C[C@@H]([C@@H]2[C@]1(C)CC[C@@H](C2(C)C)O)O)C)(O)C)C | 9847853 |
| IMPHY003983 | Ginsenoside rb1 | OC[C@H]1O[C@@H](O[C@H]2CC[C@]3([C@H](C2(C)C)CC[C@@]2([C@@H]3C[C@@H](O)[C@H]3[C@@]2(C)CC[C@@H]3[C@@](O[C@@H]2O[C@H](CO[C@@H]3O[C@H](CO)[C@H]([C@@H]([C@H]3O)O)O)[C@H]([C@@H]([C@H]2O)O)O)(CCC=C(C)C)C)C)C)[C@@H]([C@H]([C@@H]1O)O)O[C@@H]1O[C@H](CO)[C@H]([C@@H]([C@H]1O)O)O | 9898279 |
| IMPHY004045 | Ginsenoside RG2 | OC[C@H]1O[C@@H](O[C@H]2C[C@]3(C)[C@@H]([C@@]4([C@@H]2C(C)(C)[C@@H](O)CC4)C)C[C@H]([C@H]2[C@@]3(C)CC[C@@H]2[C@](CCC=C(C)C)(O)C)O)[C@@H]([C@H]([C@@H]1O)O)O[C@@H]1O[C@@H](C)[C@@H]([C@H]([C@H]1O)O)O | 21599924 |
| IMPHY004273 | Ginsenoside RG1 | OC[C@H]1O[C@@H](O[C@H]2C[C@]3(C)[C@@H]([C@@]4([C@@H]2C(C)(C)[C@@H](O)CC4)C)C[C@H]([C@H]2[C@@]3(C)CC[C@@H]2[C@@](O[C@@H]2O[C@H](CO)[C@H]([C@@H]([C@H]2O)O)O)(CCC=C(C)C)C)O)[C@@H]([C@H]([C@@H]1O)O)O | 441923 |
| IMPHY008835 | Ginsenoside rb3 | CC/C(=C/CC[C@](C1CC[C@]2(C1[C@@H](O)CC1C2CCC2[C@]1(C)CCC(C2(C)C)O[C@@H]1O[C@H](CO)[C@H]([C@@H]([C@H]1O[C@@H]1O[C@H](CO)[C@H]([C@@H]([C@H]1O)O)O)O)O)C)(O[C@@H]1O[C@H](CO[C@@H]2OC[C@H]([C@@H]([C@H]2O)O)O)[C@H]([C@H]([C@H]1O)O)O)C)/C | 6440398 |
| IMPHY009370 | Ginsenoside Rc | OC[C@H]1O[C@@H](O[C@H]2CC[C@]3([C@H](C2(C)C)CC[C@@]2([C@@H]3C[C@@H](O)[C@H]3[C@@]2(C)CC[C@@H]3[C@@](O[C@@H]2O[C@H](CO[C@@H]3O[C@H]([C@@H]([C@H]3O)O)CO)[C@H]([C@@H]([C@H]2O)O)O)(CCC=C(C)C)C)C)C)[C@@H]([C@H]([C@@H]1O)O)O[C@@H]1O[C@H](CO)[C@H]([C@@H]([C@H]1O)O)O | 12855889 |
| IMPHY010920 | Ginsenoside Rh1 | OC[C@H]1O[C@@H](O[C@H]2C[C@]3(C)[C@@H]([C@@]4([C@@H]2C(C)(C)[C@@H](O)CC4)C)C[C@H]([C@H]2[C@@]3(C)CC[C@@H]2[C@](CCC=C(C)C)(O)C)O)[C@@H]([C@H]([C@@H]1O)O)O | 12855920 |
| IMPHY010932 | (3S,4S,5S,6R)-2-[(3R,4S,5S,6R)-2-[[(3S,6S,8R,9R,10R,12R,17S)-3,12-dihydroxy-4,4,8,10,14-pentamethyl-17-[(2S)-6-methyl-2-[(3R,4S,5S,6R)-3,4,5-trihydroxy-6-(hydroxymethyl)oxan-2-yl]oxyhept-5-en-2-yl]-2, | OC[C@H]1OC(O[C@H]2C[C@]3(C)[C@@H]([C@@]4(C2C(C)(C)[C@@H](O)CC4)C)C[C@H](C2C3(C)CCC2[C@@](OC2O[C@H](CO)[C@H]([C@@H]([C@H]2O)O)O)(CCC=C(C)C)C)O)[C@@H]([C@H]([C@@H]1O)O)OC1O[C@H](C)[C@H]([C@@H]([C@@H]1O)O)O | 122130479 |
| IMPHY011454 | Gypenoside VIII | OC[C@H]1O[C@@H](O[C@H]2CC[C@]3([C@H](C2(C)C)CC[C@@]2([C@@H]3C[C@@H](O)[C@H]3[C@@]2(C)CC[C@@H]3[C@@](O[C@@H]2O[C@H](CO)[C@H]([C@@H]([C@H]2O)O)O)(CCC=C(C)C)C)C)C)[C@@H]([C@H]([C@@H]1O)O)O[C@@H]1O[C@H](CO)[C@H]([C@@H]([C@H]1O)O)O | 11679800 |
| IMPHY013064 | Ginsenoside Ro | OC[C@H]1O[C@@H](O[C@H]2[C@@H](O[C@@H]([C@H]([C@@H]2O)O)C(=O)O)O[C@H]2CC[C@]3([C@H](C2(C)C)CC[C@@]2([C@@H]3CC=C3[C@@]2(C)CC[C@@]2([C@H]3CC(C)(C)CC2)C(=O)O[C@@H]2O[C@H](CO)[C@H]([C@@H]([C@H]2O)O)O)C)C)[C@@H]([C@H]([C@@H]1O)O)O | 11815492 |
| IMPHY013710 | Ginsenoside M7cd | OCC1OC(OC(C2CCC3(C2C(O)CC2C3(C)CC(C3C2(C)CCC(C3(C)C)O)O)C)(CCC(C(=C)C)O)C)C(C(C1O)O)O | 131752701 |
| IMPHY016919 | Ginsenoside Rb2 | OC[C@H]1O[C@@H](O[C@H]2CC[C@]3([C@H](C2(C)C)CC[C@@]2([C@@H]3C[C@@H](O)[C@H]3[C@@]2(C)CC[C@@H]3[C@@](O[C@@H]2O[C@H](CO[C@@H]3OC[C@@H]([C@@H]([C@H]3O)O)O)[C@H]([C@@H]([C@H]2O)O)O)(CCC=C(C)C)C)C)C)[C@@H]([C@H]([C@@H]1O)O)O[C@@H]1O[C@H](CO)[C@H]([C@@H]([C@H]1O)O)O | 6917976 |
| IMPHY003639 | (20R)-Ginsenoside Rg3 | OC[C@H]1O[C@@H](O[C@H]2CC[C@]3([C@H](C2(C)C)CC[C@@]2([C@@H]3C[C@@H](O)[C@H]3[C@@]2(C)CC[C@@H]3[C@@](CCC=C(C)C)(O)C)C)C)[C@@H]([C@H]([C@@H]1O)O)O[C@@H]1O[C@H](CO)[C@H]([C@@H]([C@H]1O)O)O | 46887680 |
| IMPHY003983 | Ginsenoside rb1 | OC[C@H]1O[C@@H](O[C@H]2CC[C@]3([C@H](C2(C)C)CC[C@@]2([C@@H]3C[C@@H](O)[C@H]3[C@@]2(C)CC[C@@H]3[C@@](O[C@@H]2O[C@H](CO[C@@H]3O[C@H](CO)[C@H]([C@@H]([C@H]3O)O)O)[C@H]([C@@H]([C@H]2O)O)O)(CCC=C(C)C)C)C)C)[C@@H]([C@H]([C@@H]1O)O)O[C@@H]1O[C@H](CO)[C@H]([C@@H]([C@H]1O)O)O | 9898279 |
| IMPHY014754 | 20(R)-Protopanaxatriol | CC(=CCC[C@]([C@H]1CC[C@@]2([C@@H]1[C@H](O)C[C@H]1[C@@]2(C)C[C@@H]([C@@H]2[C@]1(C)CC[C@@H](C2(C)C)O)O)C)(O)C)C | 9847853 |
| IMPHY014836 | beta-Sitosterol | CC[C@@H](C(C)C)CC[C@H]([C@H]1CC[C@@H]2[C@]1(C)CC[C@H]1[C@H]2CC=C2[C@]1(C)CC[C@@H](C2)O)C | 222284 |
| IMPHY014838 | Daucosterol | CC[C@@H](C(C)C)CC[C@H]([C@H]1CC[C@@H]2[C@]1(C)CC[C@H]1[C@H]2CC=C2[C@]1(C)CC[C@@H](C2)O[C@@H]1O[C@H](CO)[C@H]([C@@H]([C@H]1O)O)O)C | 5742590 |
| IMPHY016919 | Ginsenoside Rb2 | OC[C@H]1O[C@@H](O[C@H]2CC[C@]3([C@H](C2(C)C)CC[C@@]2([C@@H]3C[C@@H](O)[C@H]3[C@@]2(C)CC[C@@H]3[C@@](O[C@@H]2O[C@H](CO[C@@H]3OC[C@@H]([C@@H]([C@H]3O)O)O)[C@H]([C@@H]([C@H]2O)O)O)(CCC=C(C)C)C)C)C)[C@@H]([C@H]([C@@H]1O)O)O[C@@H]1O[C@H](CO)[C@H]([C@@H]([C@H]1O)O)O | 6917976 |
| IMPHY001560 | 1-Heptadecanol | CCCCCCCCCCCCCCCCCO | 15076 |
| IMPHY003618 | Ginsenoside Rg3 | OC[C@H]1O[C@@H](O[C@H]2CC[C@]3([C@H](C2(C)C)CC[C@@]2([C@@H]3C[C@@H](O)[C@H]3[C@@]2(C)CC[C@@H]3[C@](CCC=C(C)C)(O)C)C)C)[C@@H]([C@H]([C@@H]1O)O)O[C@@H]1O[C@H](CO)[C@H]([C@@H]([C@H]1O)O)O | 9918693 |
| IMPHY003882 | 20(S)-Ginsenoside Rh2 | OC[C@H]1O[C@H](O[C@H]2CC[C@]3([C@H](C2(C)C)CC[C@@]2([C@@H]3C[C@@H](O)[C@H]3[C@@]2(C)CC[C@@H]3C(CCC=C(C)C)(O)C)C)C)[C@@H]([C@H]([C@@H]1O)O)O | 91758817 |
| IMPHY003983 | Ginsenoside rb1 | OC[C@H]1O[C@@H](O[C@H]2CC[C@]3([C@H](C2(C)C)CC[C@@]2([C@@H]3C[C@@H](O)[C@H]3[C@@]2(C)CC[C@@H]3[C@@](O[C@@H]2O[C@H](CO[C@@H]3O[C@H](CO)[C@H]([C@@H]([C@H]3O)O)O)[C@H]([C@@H]([C@H]2O)O)O)(CCC=C(C)C)C)C)C)[C@@H]([C@H]([C@@H]1O)O)O[C@@H]1O[C@H](CO)[C@H]([C@@H]([C@H]1O)O)O | 9898279 |
| IMPHY004040 | Ginsenoside Rh2 | OC[C@H]1O[C@@H](O[C@H]2CC[C@]3([C@H](C2(C)C)CC[C@@]2([C@@H]3C[C@@H](O)[C@H]3[C@@]2(C)CC[C@@H]3[C@](CCC=C(C)C)(O)C)C)C)[C@@H]([C@H]([C@@H]1O)O)O | 119307 |
| IMPHY004045 | Ginsenoside RG2 | OC[C@H]1O[C@@H](O[C@H]2C[C@]3(C)[C@@H]([C@@]4([C@@H]2C(C)(C)[C@@H](O)CC4)C)C[C@H]([C@H]2[C@@]3(C)CC[C@@H]2[C@](CCC=C(C)C)(O)C)O)[C@@H]([C@H]([C@@H]1O)O)O[C@@H]1O[C@@H](C)[C@@H]([C@H]([C@H]1O)O)O | 21599924 |
| IMPHY004046 | 20R-Ginsenoside Rg2 | OC[C@H]1O[C@@H](O[C@H]2C[C@]3(C)[C@@H]([C@@]4([C@@H]2C(C)(C)[C@@H](O)CC4)C)C[C@H]([C@H]2[C@@]3(C)CC[C@@H]2[C@@](CCC=C(C)C)(O)C)O)[C@@H]([C@H]([C@@H]1O)O)O[C@@H]1O[C@@H](C)[C@@H]([C@H]([C@H]1O)O)O | 75412551 |
| IMPHY004273 | Ginsenoside RG1 | OC[C@H]1O[C@@H](O[C@H]2C[C@]3(C)[C@@H]([C@@]4([C@@H]2C(C)(C)[C@@H](O)CC4)C)C[C@H]([C@H]2[C@@]3(C)CC[C@@H]2[C@@](O[C@@H]2O[C@H](CO)[C@H]([C@@H]([C@H]2O)O)O)(CCC=C(C)C)C)O)[C@@H]([C@H]([C@@H]1O)O)O | 441923 |
| IMPHY004275 | Ginsenoside-rgl | OCC1OC(O[C@H]2C[C@]3(C)[C@@H]([C@@]4([C@@H]2C(C)(C)[C@@H](O)CC4)C)C[C@H]([C@H]2[C@@]3(C)CCC2[C@@](OC2OC(CO)C(C(C2O)O)O)(CCC=C(C)C)C)O)C(C(C1O)O)O | 5280863 |
| IMPHY004388 | Kaempferol | Oc1ccc(cc1)c1oc2cc(O)cc(c2c(=O)c1O)O | 5280863 |
| IMPHY006734 | Ginsenoside Rh3 | OC[C@H]1O[C@@H](O[C@H]2CC[C@]3([C@H](C2(C)C)CC[C@@]2([C@@H]3C[C@@H](O)[C@H]3[C@@]2(C)CC[C@@H]3/C(=CCC=C(C)C)/C)C)C)[C@@H]([C@H]([C@@H]1O)O)O | 20839223 |
| IMPHY006971 | Methyl palmitate | CCCCCCCCCCCCCCCC(=O)OC | 8181 |
| IMPHY007322 | ginsenoside F2 | OC[C@H]1O[C@@H](O[C@H]2CC[C@]3([C@H](C2(C)C)CC[C@@]2([C@@H]3C[C@@H](O)[C@H]3[C@@]2(C)CC[C@@H]3[C@@](O[C@@H]2O[C@H](CO)[C@H]([C@@H]([C@H]2O)O)O)(CCC=C(C)C)C)C)C)[C@@H]([C@H]([C@@H]1O)O)O | 9918692 |
| IMPHY007327 | Palmitic acid | CCCCCCCCCCCCCCCC(=O)O | 985 |
| IMPHY007366 | alpha-Santalene | CC(=CCCC1(C)C2CC3C1(C)C3C2)C | 94164 |
| IMPHY008095 | Ginsenoside F3 | CC(=CCC[C@@]([C@H]1CC[C@@]2([C@@H]1[C@H](O)C[C@H]1[C@@]2(C)C[C@@H]([C@@H]2[C@]1(C)CC[C@@H](C2(C)C)O)O)C)(O[C@@H]1O[C@H](CO[C@@H]2OC[C@@H]([C@@H]([C@H]2O)O)O)[C@H]([C@@H]([C@H]1O)O)O)C)C | 46887678 |
| IMPHY008305 | 2-Heptadecanone | CCCCCCCCCCCCCCCC(=O)C | 18027 |
| IMPHY008835 | Ginsenoside rb3 | CC/C(=C/CC[C@](C1CC[C@]2(C1[C@@H](O)CC1C2CCC2[C@]1(C)CCC(C2(C)C)O[C@@H]1O[C@H](CO)[C@H]([C@@H]([C@H]1O[C@@H]1O[C@H](CO)[C@H]([C@@H]([C@H]1O)O)O)O)O)C)(O[C@@H]1O[C@H](CO[C@@H]2OC[C@H]([C@@H]([C@H]2O)O)O)[C@H]([C@H]([C@H]1O)O)O)C)/C | 6440398 |
| IMPHY008906 | Ginsenoside F1 | OC[C@H]1O[C@@H](O[C@]([C@H]2CC[C@@]3([C@@H]2[C@H](O)C[C@H]2[C@@]3(C)C[C@@H]([C@@H]3[C@]2(C)CC[C@@H](C3(C)C)O)O)C)(CCC=C(C)C)C)[C@@H]([C@H]([C@@H]1O)O)O | 9809542 |
| IMPHY008936 | alpha-Guaiene | CC(=C)[C@@H]1CC[C@@H](C2=C(C1)[C@@H](C)CC2)C | 5317844 |
| IMPHY009370 | Ginsenoside Rc | OC[C@H]1O[C@@H](O[C@H]2CC[C@]3([C@H](C2(C)C)CC[C@@]2([C@@H]3C[C@@H](O)[C@H]3[C@@]2(C)CC[C@@H]3[C@@](O[C@@H]2O[C@H](CO[C@@H]3O[C@H]([C@@H]([C@H]3O)O)CO)[C@H]([C@@H]([C@H]2O)O)O)(CCC=C(C)C)C)C)C)[C@@H]([C@H]([C@@H]1O)O)O[C@@H]1O[C@H](CO)[C@H]([C@@H]([C@H]1O)O)O | 12855889 |
| IMPHY009389 | Pentadecane | CCCCCCCCCCCCCCC | 12391 |
| IMPHY009550 | Ginsenoside-La | OC[C@H]1O[C@@H](O[C@H]2CC[C@]3(C(C2(C)C)CC[C@@H]2[C@@H]3C[C@H]3O[C@@H](C=C(C)C)C[C@]([C@@H]4C3[C@]2(C)CC4)(C)O[C@@H]2O[C@H](CO)[C@H]([C@@H]([C@H]2O)O)O)C)[C@@H]([C@H]([C@@H]1O)O)O | 130009 |
| IMPHY009743 | beta-Gurjunene | C[C@@H]1CC[C@@H]2[C@H]([C@H]3[C@@H]1CCC3=C)C2(C)C | 6450812 |
| IMPHY010080 | beta-Elemene | C=C[C@]1(C)CC[C@H](C[C@H]1C(=C)C)C(=C)C | 6918391 |
| IMPHY010085 | Ginsenoside RG4 | OC[C@H]1O[C@@H](O[C@H]2C[C@]3(C)[C@@H]([C@@]4([C@@H]2C(C)(C)[C@@H](O)CC4)C)C[C@H]([C@H]2[C@@]3(C)CC[C@@H]2/C(=CCC=C(C)C)/C)O)[C@@H]([C@H]([C@@H]1O)O)O[C@@H]1O[C@@H](C)[C@@H]([C@H]([C@H]1O)O)O | 102004835 |
| IMPHY010906 | Pseudoginsenoside FII | OC[C@H]1O[C@@H](O[C@H]2C[C@]3(C)[C@@H]([C@@]4([C@@H]2C(C)(C)[C@@H](O)CC4)C)C[C@H]([C@H]2[C@@]3(C)CC[C@@H]2[C@]2(C)CC[C@@H](O2)C(O)(C)C)O)[C@@H]([C@H]([C@@H]1O)O)O[C@@H]1O[C@@H](C)[C@@H]([C@H]([C@H]1O)O)O | 21633072 |
| IMPHY010920 | Ginsenoside Rh1 | OC[C@H]1O[C@@H](O[C@H]2C[C@]3(C)[C@@H]([C@@]4([C@@H]2C(C)(C)[C@@H](O)CC4)C)C[C@H]([C@H]2[C@@]3(C)CC[C@@H]2[C@](CCC=C(C)C)(O)C)O)[C@@H]([C@H]([C@@H]1O)O)O | 12855920 |
| IMPHY010932 | (3S,4S,5S,6R)-2-[(3R,4S,5S,6R)-2-[[(3S,6S,8R,9R,10R,12R,17S)-3,12-dihydroxy-4,4,8,10,14-pentamethyl-17-[(2S)-6-methyl-2-[(3R,4S,5S,6R)-3,4,5-trihydroxy-6-(hydroxymethyl)oxan-2-yl]oxyhept-5-en-2-yl]-2, | OC[C@H]1OC(O[C@H]2C[C@]3(C)[C@@H]([C@@]4(C2C(C)(C)[C@@H](O)CC4)C)C[C@H](C2C3(C)CCC2[C@@](OC2O[C@H](CO)[C@H]([C@@H]([C@H]2O)O)O)(CCC=C(C)C)C)O)[C@@H]([C@H]([C@@H]1O)O)OC1O[C@H](C)[C@H]([C@@H]([C@@H]1O)O)O | 122130479 |
| IMPHY011026 | (20R)-Ginsenoside Rh1 | OC[C@H]1O[C@@H](O[C@H]2C[C@]3(C)[C@@H]([C@@]4([C@@H]2C(C)(C)[C@@H](O)CC4)C)C[C@H]([C@H]2[C@@]3(C)CC[C@@H]2[C@@](CCC=C(C)C)(O)C)O)[C@@H]([C@H]([C@@H]1O)O)O | 21599923 |
| IMPHY011454 | Gypenoside VIII | OC[C@H]1O[C@@H](O[C@H]2CC[C@]3([C@H](C2(C)C)CC[C@@]2([C@@H]3C[C@@H](O)[C@H]3[C@@]2(C)CC[C@@H]3[C@@](O[C@@H]2O[C@H](CO)[C@H]([C@@H]([C@H]2O)O)O)(CCC=C(C)C)C)C)C)[C@@H]([C@H]([C@@H]1O)O)O[C@@H]1O[C@H](CO)[C@H]([C@@H]([C@H]1O)O)O | 11679800 |
| IMPHY011579 | Eremophilene | CC(=C)[C@@H]1CCC2=CCC[C@@H]([C@]2(C1)C)C | 12309744 |
| IMPHY011754 | Kaempferol 3-O-beta-D-glucosylgalactoside | OC[C@H]1O[C@@H](Oc2c(oc3c(c2=O)c(O)cc(c3)O)c2ccc(cc2)O)[C@@H]([C@H]([C@H]1O)O)O[C@@H]1O[C@H](CO)[C@H]([C@@H]([C@H]1O)O)O | 9986191 |
| IMPHY011761 | Humulene | C/C/1=CCC(C)(C)/C=C/C/C(=C/CC1)/C | 5281520 |
| IMPHY012151 | 2-Methyltetradecane | CCCCCCCCCCCCC(C)C | 15268 |
| IMPHY014624 | Dammaran-3,6,12,20,25-pentol-6-O-rhamnopyranosyl-(1-2)-O-glucopyranoside | OC[C@H]1O[C@@H](O[C@H]2C[C@]3(C)[C@@H]([C@@]4([C@@H]2C(C)(C)[C@@H](O)CC4)C)C[C@H]([C@H]2[C@@]3(C)CC[C@@H]2[C@@](CCCC(O)(C)C)(O)C)O)[C@@H]([C@H]([C@@H]1O)O)O[C@@H]1O[C@@H](C)[C@@H]([C@H]([C@H]1O)O)O | 129010973 |
| IMPHY014754 | 20(R)-Protopanaxatriol | CC(=CCC[C@]([C@H]1CC[C@@]2([C@@H]1[C@H](O)C[C@H]1[C@@]2(C)C[C@@H]([C@@H]2[C@]1(C)CC[C@@H](C2(C)C)O)O)C)(O)C)C | 9847853 |
| IMPHY014831 | beta-Caryophyllene | C/C/1=CCCC(=C)[C@@H]2[C@@H](CC1)C(C2)(C)C | 5281515 |
| IMPHY014966 | Trifolin | OC[C@H]1O[C@@H](Oc2c(oc3c(c2=O)c(O)cc(c3)O)c2ccc(cc2)O)[C@@H]([C@H]([C@H]1O)O)O | 5282149 |
| IMPHY016919 | Ginsenoside Rb2 | OC[C@H]1O[C@@H](O[C@H]2CC[C@]3([C@H](C2(C)C)CC[C@@]2([C@@H]3C[C@@H](O)[C@H]3[C@@]2(C)CC[C@@H]3[C@@](O[C@@H]2O[C@H](CO[C@@H]3OC[C@@H]([C@@H]([C@H]3O)O)O)[C@H]([C@@H]([C@H]2O)O)O)(CCC=C(C)C)C)C)C)[C@@H]([C@H]([C@@H]1O)O)O[C@@H]1O[C@H](CO)[C@H]([C@@H]([C@H]1O)O)O | 6917976 |
| IMPHY007778 | 1-Chloro-8-(3-heptyloxiran-2-yl)octa-4,6-diyne-2,3-diol | CCCCCCCC1OC1CC#CC#CC(C(CCl)O)O | 130756 |
| IMPHY009511 | Panaxacol | CCCCCCC[C@H]([C@@H](CC#CC#CC(=O)CC)O)O | 129429 |
| IMPHY009577 | (3S,9R,10R)-heptadeca-4,6-diyne-3,9,10-triol | CCCCCCC[C@H]([C@@H](CC#CC#C[C@H](CC)O)O)O | 130309 |
| IMPHY003618 | Ginsenoside Rg3 | OC[C@H]1O[C@@H](O[C@H]2CC[C@]3([C@H](C2(C)C)CC[C@@]2([C@@H]3C[C@@H](O)[C@H]3[C@@]2(C)CC[C@@H]3[C@](CCC=C(C)C)(O)C)C)C)[C@@H]([C@H]([C@@H]1O)O)O[C@@H]1O[C@H](CO)[C@H]([C@@H]([C@H]1O)O)O | 9918693 |
| IMPHY004045 | Ginsenoside RG2 | OC[C@H]1O[C@@H](O[C@H]2C[C@]3(C)[C@@H]([C@@]4([C@@H]2C(C)(C)[C@@H](O)CC4)C)C[C@H]([C@H]2[C@@]3(C)CC[C@@H]2[C@](CCC=C(C)C)(O)C)O)[C@@H]([C@H]([C@@H]1O)O)O[C@@H]1O[C@@H](C)[C@@H]([C@H]([C@H]1O)O)O | 21599924 |
| IMPHY009370 | Ginsenoside Rc | OC[C@H]1O[C@@H](O[C@H]2CC[C@]3([C@H](C2(C)C)CC[C@@]2([C@@H]3C[C@@H](O)[C@H]3[C@@]2(C)CC[C@@H]3[C@@](O[C@@H]2O[C@H](CO[C@@H]3O[C@H]([C@@H]([C@H]3O)O)CO)[C@H]([C@@H]([C@H]2O)O)O)(CCC=C(C)C)C)C)C)[C@@H]([C@H]([C@@H]1O)O)O[C@@H]1O[C@H](CO)[C@H]([C@@H]([C@H]1O)O)O | 12855889 |
| IMPHY010297 | Ginsenoside Rd1 | OC[C@H]1O[C@@H](OC2CC[C@]3([C@H](C2(C)C)CC[C@@]2([C@@H]3C[C@H](O)[C@H]3[C@]2(C)CC[C@@H]3[C@@](O[C@@H]2O[C@H](CO)[C@H]([C@@H]([C@H]2O)O)O)(CCC=C(C)C)C)C)C)[C@@H]([C@H]([C@@H]1O)O)O[C@@H]1O[C@H](CO)[C@H]([C@@H]([C@H]1O)O)O | 102221467 |
| IMPHY010920 | Ginsenoside Rh1 | OC[C@H]1O[C@@H](O[C@H]2C[C@]3(C)[C@@H]([C@@]4([C@@H]2C(C)(C)[C@@H](O)CC4)C)C[C@H]([C@H]2[C@@]3(C)CC[C@@H]2[C@](CCC=C(C)C)(O)C)O)[C@@H]([C@H]([C@@H]1O)O)O | 12855920 |
| IMPHY010932 | (3S,4S,5S,6R)-2-[(3R,4S,5S,6R)-2-[[(3S,6S,8R,9R,10R,12R,17S)-3,12-dihydroxy-4,4,8,10,14-pentamethyl-17-[(2S)-6-methyl-2-[(3R,4S,5S,6R)-3,4,5-trihydroxy-6-(hydroxymethyl)oxan-2-yl]oxyhept-5-en-2-yl]-2, | OC[C@H]1OC(O[C@H]2C[C@]3(C)[C@@H]([C@@]4(C2C(C)(C)[C@@H](O)CC4)C)C[C@H](C2C3(C)CCC2[C@@](OC2O[C@H](CO)[C@H]([C@@H]([C@H]2O)O)O)(CCC=C(C)C)C)O)[C@@H]([C@H]([C@@H]1O)O)OC1O[C@H](C)[C@H]([C@@H]([C@@H]1O)O)O | 122130479 |
| IMPHY011454 | Gypenoside VIII | OC[C@H]1O[C@@H](O[C@H]2CC[C@]3([C@H](C2(C)C)CC[C@@]2([C@@H]3C[C@@H](O)[C@H]3[C@@]2(C)CC[C@@H]3[C@@](O[C@@H]2O[C@H](CO)[C@H]([C@@H]([C@H]2O)O)O)(CCC=C(C)C)C)C)C)[C@@H]([C@H]([C@@H]1O)O)O[C@@H]1O[C@H](CO)[C@H]([C@@H]([C@H]1O)O)O | 11679800 |
| IMPHY012053 | Sucrose | OC[C@H]1O[C@@]([C@H]([C@@H]1O)O)(CO)O[C@H]1O[C@H](CO)[C@H]([C@@H]([C@H]1O)O)O | 5988 |
| IMPHY012197 | Adenosine | OC[C@H]1O[C@H]([C@@H]([C@@H]1O)O)n1cnc2c1ncnc2N | 60961 |
| IMPHY013064 | Ginsenoside Ro | OC[C@H]1O[C@@H](O[C@H]2[C@@H](O[C@@H]([C@H]([C@@H]2O)O)C(=O)O)O[C@H]2CC[C@]3([C@H](C2(C)C)CC[C@@]2([C@@H]3CC=C3[C@@]2(C)CC[C@@]2([C@H]3CC(C)(C)CC2)C(=O)O[C@@H]2O[C@H](CO)[C@H]([C@@H]([C@H]2O)O)O)C)C)[C@@H]([C@H]([C@@H]1O)O)O | 11815492 |
| IMPHY014836 | beta-Sitosterol | CC[C@@H](C(C)C)CC[C@H]([C@H]1CC[C@@H]2[C@]1(C)CC[C@H]1[C@H]2CC=C2[C@]1(C)CC[C@@H](C2)O)C | 222284 |
| IMPHY016919 | Ginsenoside Rb2 | OC[C@H]1O[C@@H](O[C@H]2CC[C@]3([C@H](C2(C)C)CC[C@@]2([C@@H]3C[C@@H](O)[C@H]3[C@@]2(C)CC[C@@H]3[C@@](O[C@@H]2O[C@H](CO[C@@H]3OC[C@@H]([C@@H]([C@H]3O)O)O)[C@H]([C@@H]([C@H]2O)O)O)(CCC=C(C)C)C)C)C)[C@@H]([C@H]([C@@H]1O)O)O[C@@H]1O[C@H](CO)[C@H]([C@@H]([C@H]1O)O)O | 6917976 |
| IMPHY000699 | Ginsenoside Rs3 | OC[C@H]1O[C@@H](O[C@H]2CC[C@]3([C@H](C2(C)C)CC[C@@]2([C@@H]3C[C@@H](O)[C@H]3[C@@]2(C)CC[C@@H]3[C@](CCC=C(C)C)(O)C)C)C)[C@@H]([C@H]([C@@H]1O)O)O[C@@H]1O[C@H](COC(=O)C)[C@H]([C@@H]([C@H]1O)O)O | 100937823 |
| IMPHY001199 | Senecrassidiol | O[C@@H]1CC[C@]2(C[C@]1(C)CC[C@@H]1[C@H]2CC1(C)C)O | 102059900 |
| IMPHY001560 | 1-Heptadecanol | CCCCCCCCCCCCCCCCCO | 15076 |
| IMPHY001717 | Perlolyrine | OCc1ccc(o1)c1nccc2c1[nH]c1c2cccc1 | 160179 |
| IMPHY001915 | Octadecane | CCCCCCCCCCCCCCCCCC | 11635 |
| IMPHY003208 | Ginsenol | CC1(C)C[C@@]2([C@@]3([C@H]1CC[C@@]2(C)CCC3)C)O | 3082861 |
| IMPHY003616 | Bicyclogermacrene | C/C/1=CCC/C(=C/[C@H]2[C@@H](CC1)C2(C)C)/C | 13894537 |
| IMPHY003695 | (-)-Germacrene A | C/C/1=CCC/C(=C/C[C@H](CC1)C(=C)C)/C | 9548706 |
| IMPHY003983 | Ginsenoside rb1 | OC[C@H]1O[C@@H](O[C@H]2CC[C@]3([C@H](C2(C)C)CC[C@@]2([C@@H]3C[C@@H](O)[C@H]3[C@@]2(C)CC[C@@H]3[C@@](O[C@@H]2O[C@H](CO[C@@H]3O[C@H](CO)[C@H]([C@@H]([C@H]3O)O)O)[C@H]([C@@H]([C@H]2O)O)O)(CCC=C(C)C)C)C)C)[C@@H]([C@H]([C@@H]1O)O)O[C@@H]1O[C@H](CO)[C@H]([C@@H]([C@H]1O)O)O | 9898279 |
| IMPHY004045 | Ginsenoside RG2 | OC[C@H]1O[C@@H](O[C@H]2C[C@]3(C)[C@@H]([C@@]4([C@@H]2C(C)(C)[C@@H](O)CC4)C)C[C@H]([C@H]2[C@@]3(C)CC[C@@H]2[C@](CCC=C(C)C)(O)C)O)[C@@H]([C@H]([C@@H]1O)O)O[C@@H]1O[C@@H](C)[C@@H]([C@H]([C@H]1O)O)O | 21599924 |
| IMPHY004120 | (-)-Cyperene | CC1=C2C[C@@H]3C([C@@]2(CC1)[C@H](C)CC3)(C)C | 12308843 |
| IMPHY004134 | gomisin N | COc1cc2C[C@@H](C)[C@@H](C)Cc3c(-c2c(c1OC)OC)c(OC)c1c(c3)OCO1 | 158103 |
| IMPHY004187 | L-(+)-Arabinose | OC[C@@H]([C@@H]([C@H](C=O)O)O)O | 5460291 |
| IMPHY004273 | Ginsenoside RG1 | OC[C@H]1O[C@@H](O[C@H]2C[C@]3(C)[C@@H]([C@@]4([C@@H]2C(C)(C)[C@@H](O)CC4)C)C[C@H]([C@H]2[C@@]3(C)CC[C@@H]2[C@@](O[C@@H]2O[C@H](CO)[C@H]([C@@H]([C@H]2O)O)O)(CCC=C(C)C)C)O)[C@@H]([C@H]([C@@H]1O)O)O | 441923 |
| IMPHY004631 | Stearic acid | CCCCCCCCCCCCCCCCCC(=O)O | 5281 |
| IMPHY004650 | 2-sec-Butyl-3-methoxypyrazine | CCC(c1nccnc1OC)C | 520098 |
| IMPHY004812 | Ginsenoside Rf | OC[C@H]1O[C@@H](O[C@H]2C[C@]3(C)[C@@H]([C@@]4([C@@H]2C(C)(C)[C@@H](O)CC4)C)C[C@H]([C@H]2[C@@]3(C)CC[C@@H]2[C@](CCC=C(C)C)(O)C)O)[C@@H]([C@H]([C@@H]1O)O)O[C@@H]1O[C@H](CO)[C@H]([C@@H]([C@H]1O)O)O | 441922 |
| IMPHY004965 | Trilinolein | CCCCC/C=CC/C=CCCCCCCCC(=O)OC(COC(=O)CCCCCCC/C=CC/C=CCCCCC)COC(=O)CCCCCCC/C=CC/C=CCCCCC | 5322095 |
| IMPHY005811 | 2-Pentylfuran | CCCCCc1ccco1 | 19602 |
| IMPHY006288 | beta-Panasinsene | C=C1CCCC2(C31CC(C3CC2)(C)C)C | 595133 |
| IMPHY006735 | 8-[(2R,3S)-3-hept-6-enyloxiran-2-yl]oct-1-en-4,6-diyn-3-yl acetate | CC(=O)OC(C#CC#CC[C@H]1O[C@H]1CCCCCC=C)C=C | 101618812 |
| IMPHY006736 | 8-[(2R,3S)-3-heptyloxiran-2-yl]octa-4,6-diyn-3-yl acetate | CCCCCCC[C@@H]1O[C@@H]1CC#CC#CC(OC(=O)C)CC | 101618813 |
| IMPHY006746 | 8-(3-heptyloxiran-2-yl)oct-1-en-4,6-diyn-3-yl (9E,12E)-octadeca-9,12-dienoate | CCCCCCCC1OC1CC#CC#CC(OC(=O)CCCCCCC/C=C/C/C=C/CCCCC)C=C | 131753067 |
| IMPHY006877 | (3r)-8-[(2r,3s)-3-Heptyloxiran-2-yl]oct-1-ene-4,6-diyn-3-ol | CCCCCCC[C@@H]1O[C@@H]1CC#CC#C[C@@H](C=C)O | 126312 |
| IMPHY006878 | Panaxynol linoleate | CCCCCCC/C=C/CC#CC#CC(OC(=O)CCCCCCC/C=C/C/C=CCCCCC)C=C | 131753057 |
| IMPHY006901 | (3S,5R,6S,8R,9R,10R,12R,13R,14R,17S)-4,4,8,10,14-pentamethyl-17-[(2R)-2,6,6-trimethyloxan-2-yl]-2,3,5,6,7,9,11,12,13,15,16,17-dodecahydro-1H-cyclopenta[a]phenanthrene-3,6,12-triol | O[C@H]1C[C@]2(C)[C@@H]([C@@]3([C@@H]1C(C)(C)[C@@H](O)CC3)C)C[C@H]([C@H]1[C@@]2(C)CC[C@@H]1[C@@]1(C)CCCC(O1)(C)C)O | 12313935 |
| IMPHY006971 | Methyl palmitate | CCCCCCCCCCCCCCCC(=O)OC | 8181 |
| IMPHY007012 | Panaxadiol | O[C@@H]1C[C@@H]2[C@@]3(C)CC[C@@H](C([C@@H]3CC[C@]2([C@]2([C@H]1[C@H](CC2)[C@@]1(C)CCCC(O1)(C)C)C)C)(C)C)O | 73498 |
| IMPHY007058 | Maltol | O=c1ccoc(c1O)C | 8369 |
| IMPHY007322 | ginsenoside F2 | OC[C@H]1O[C@@H](O[C@H]2CC[C@]3([C@H](C2(C)C)CC[C@@]2([C@@H]3C[C@@H](O)[C@H]3[C@@]2(C)CC[C@@H]3[C@@](O[C@@H]2O[C@H](CO)[C@H]([C@@H]([C@H]2O)O)O)(CCC=C(C)C)C)C)C)[C@@H]([C@H]([C@@H]1O)O)O | 9918692 |
| IMPHY007327 | Palmitic acid | CCCCCCCCCCCCCCCC(=O)O | 985 |
| IMPHY007366 | alpha-Santalene | CC(=CCCC1(C)C2CC3C1(C)C3C2)C | 94164 |
| IMPHY007378 | (2S,3R,4R,5S,6S)-2-(hydroxymethyl)-6-(((2S,3R,4R,5S,6R)-3,4,5-trihydroxy-6-(((4aR,8aS)-4-isopropyl-1,7-dimethyl-1,2,3,4,4a,5,8,8a-octahydronaphthalen-1-yl)oxy)tetrahydro-2H-pyran-2-yl)methoxy)tetrahyd | OC[C@@H]1O[C@H](OC[C@@H]2O[C@H](OC3(C)CCC([C@@H]4[C@@H]3CC(=CC4)C)C(C)C)[C@H]([C@@H]([C@H]2O)O)O)[C@H]([C@@H]([C@H]1O)O)O | 91754221 |
| IMPHY007386 | Panaxytriol | CCCCCCCC(C(CC#CC#CC(C=C)O)O)O | 93484 |
| IMPHY007840 | Spathulenol | C=C1CC[C@@H]2[C@H]([C@H]3[C@H]1CC[C@]3(C)O)C2(C)C | 92231 |
| IMPHY008085 | Ginsenoyne A | C=CCCCCCC1OC1CC#CC#CC(C=C)O | 5317632 |
| IMPHY008086 | Ginsenoyne A linoleate | CCCCC/C=C/C/C=C/CCCCCCCC(=O)OC(C#CC#CCC1OC1CCCCCC=C)C=C | 131753066 |
| IMPHY008087 | Ginsenoyne C | C=CCCCCCC(C(CC#CC#CC(C=C)O)O)O | 5317634 |
| IMPHY008088 | Ginsenoyne E | CCCCCCCC1OC1CC#CC#CC(=O)C=C | 5320336 |
| IMPHY008089 | (4E)-8-[(2R,3S)-3-heptyloxiran-2-yl]octa-1,4-dien-6-yn-3-ol | CCCCCCC[C@@H]1O[C@@H]1CC#C/C=C/C(C=C)O | 101625116 |
| IMPHY008094 | Ginsenoside Ra1 | OC[C@H]1O[C@@H](O[C@H]2CC[C@]3([C@H](C2(C)C)CC[C@@]2([C@@H]3C[C@@H](O)[C@H]3[C@@]2(C)CC[C@@H]3[C@@](O[C@@H]2O[C@H](CO[C@@H]3OC[C@@H]([C@@H]([C@H]3O)O)O[C@@H]3OC[C@H]([C@@H]([C@H]3O)O)O)[C@H]([C@@H]([C@H]2O)O)O)(CCC=C(C)C)C)C)C)[C@@H]([C@H]([C@@H]1O)O)O[C@@H]1O[C@H](CO)[C@H]([C@@H]([C@H]1O)O)O |  |
| IMPHY008131 | Ginsenoyne K | CCCCCCCC(/C=C/C#CC#CC(C=C)O)OO | 15736266 |
| IMPHY008148 | Ginsenoside Ra2 | OC[C@@H]1O[C@H]([C@@H]([C@H]1O)O[C@@H]1OC[C@H]([C@@H]([C@H]1O)O)O)OC[C@H]1O[C@@H](O[C@]([C@H]2CC[C@@]3([C@@H]2[C@H](O)C[C@H]2[C@@]3(C)CC[C@@H]3[C@]2(C)CC[C@@H](C3(C)C)O[C@@H]2O[C@H](CO)[C@H]([C@@H]([C@H]2O[C@@H]2O[C@H](CO)[C@H]([C@@H]([C@H]2O)O)O)O)O)C)(CCC=C(C)C)C)[C@@H]([C@H]([C@@H]1O)O)O | 100941543 |
| IMPHY008305 | 2-Heptadecanone | CCCCCCCCCCCCCCCC(=O)C | 18027 |
| IMPHY008835 | Ginsenoside rb3 | CC/C(=C/CC[C@](C1CC[C@]2(C1[C@@H](O)CC1C2CCC2[C@]1(C)CCC(C2(C)C)O[C@@H]1O[C@H](CO)[C@H]([C@@H]([C@H]1O[C@@H]1O[C@H](CO)[C@H]([C@@H]([C@H]1O)O)O)O)O)C)(O[C@@H]1O[C@H](CO[C@@H]2OC[C@H]([C@@H]([C@H]2O)O)O)[C@H]([C@H]([C@H]1O)O)O)C)/C | 6440398 |
| IMPHY008936 | alpha-Guaiene | CC(=C)[C@@H]1CC[C@@H](C2=C(C1)[C@@H](C)CC2)C | 5317844 |
| IMPHY009323 | n9-Formylharman | O=Cn1c2ccccc2c2c1C(=NCC2)C | 129650345 |
| IMPHY009355 | Tetracosane | CCCCCCCCCCCCCCCCCCCCCCCC | 12592 |
| IMPHY009369 | Nonadecane | CCCCCCCCCCCCCCCCCCC | 12401 |
| IMPHY009370 | Ginsenoside Rc | OC[C@H]1O[C@@H](O[C@H]2CC[C@]3([C@H](C2(C)C)CC[C@@]2([C@@H]3C[C@@H](O)[C@H]3[C@@]2(C)CC[C@@H]3[C@@](O[C@@H]2O[C@H](CO[C@@H]3O[C@H]([C@@H]([C@H]3O)O)CO)[C@H]([C@@H]([C@H]2O)O)O)(CCC=C(C)C)C)C)C)[C@@H]([C@H]([C@@H]1O)O)O[C@@H]1O[C@H](CO)[C@H]([C@@H]([C@H]1O)O)O | 12855889 |
| IMPHY009375 | Docosane | CCCCCCCCCCCCCCCCCCCCCC | 12405 |
| IMPHY009377 | Pentacosane | CCCCCCCCCCCCCCCCCCCCCCCCC | 12406 |
| IMPHY009382 | Heneicosane | CCCCCCCCCCCCCCCCCCCCC | 12403 |
| IMPHY009389 | Pentadecane | CCCCCCCCCCCCCCC | 12391 |
| IMPHY009490 | Tricosane | CCCCCCCCCCCCCCCCCCCCCCC | 12534 |
| IMPHY009624 | Ethyl palmitate | CCCCCCCCCCCCCCCC(=O)OCC | 12366 |
| IMPHY009743 | beta-Gurjunene | C[C@@H]1CC[C@@H]2[C@H]([C@H]3[C@@H]1CCC3=C)C2(C)C | 6450812 |
| IMPHY010080 | beta-Elemene | C=C[C@]1(C)CC[C@H](C[C@H]1C(=C)C)C(=C)C | 6918391 |
| IMPHY010585 | (1aR,7R,7aR,7bS)-1,1,7,7a-tetramethyl-2,3,5,6,7,7b-hexahydro-1aH-cyclopropa[a]naphthalene | C[C@@H]1CCC=C2[C@@]1(C)[C@H]1[C@H](C1(C)C)CC2 | 15560278 |
| IMPHY010599 | Besigomsin | COc1cc2C[C@](C)(O)[C@@H](C)Cc3c(-c2c(c1OC)OC)c(OC)c1c(c3)OCO1 | 3001662 |
| IMPHY010685 | Ginsenoyne J | CCCCCCC/C=C/CC#C/C=C/C(C=C)O | 131752800 |
| IMPHY010920 | Ginsenoside Rh1 | OC[C@H]1O[C@@H](O[C@H]2C[C@]3(C)[C@@H]([C@@]4([C@@H]2C(C)(C)[C@@H](O)CC4)C)C[C@H]([C@H]2[C@@]3(C)CC[C@@H]2[C@](CCC=C(C)C)(O)C)O)[C@@H]([C@H]([C@@H]1O)O)O | 12855920 |
| IMPHY010932 | (3S,4S,5S,6R)-2-[(3R,4S,5S,6R)-2-[[(3S,6S,8R,9R,10R,12R,17S)-3,12-dihydroxy-4,4,8,10,14-pentamethyl-17-[(2S)-6-methyl-2-[(3R,4S,5S,6R)-3,4,5-trihydroxy-6-(hydroxymethyl)oxan-2-yl]oxyhept-5-en-2-yl]-2, | OC[C@H]1OC(O[C@H]2C[C@]3(C)[C@@H]([C@@]4(C2C(C)(C)[C@@H](O)CC4)C)C[C@H](C2C3(C)CCC2[C@@](OC2O[C@H](CO)[C@H]([C@@H]([C@H]2O)O)O)(CCC=C(C)C)C)O)[C@@H]([C@H]([C@@H]1O)O)OC1O[C@H](C)[C@H]([C@@H]([C@@H]1O)O)O | 122130479 |
| IMPHY010987 | Malonylginsenoside Rb(1) | OC[C@H]1O[C@@H](O[C@H]2CC[C@]3([C@H](C2(C)C)CC[C@@]2([C@@H]3C[C@@H](O)[C@H]3[C@@]2(C)CC[C@@H]3[C@@](O[C@@H]2O[C@H](CO[C@@H]3O[C@H](CO)[C@H]([C@@H]([C@H]3O)O)O)[C@H]([C@@H]([C@H]2O)O)O)(CCC=C(C)C)C)C)C)[C@@H]([C@H]([C@@H]1O)O)O[C@H]1O[C@H](COC(=O)CC(=O)O)[C@H]([C@@H]([C@H]1O)O)O | 118987129 |
| IMPHY010991 | Malonyl ginsenoside Rd | OC[C@H]1O[C@@H](O[C@H]2CC[C@]3([C@H](C2(C)C)CC[C@@]2([C@@H]3C[C@@H](O)[C@H]3[C@@]2(C)CC[C@@H]3[C@@](O[C@@H]2O[C@H](CO)[C@H]([C@@H]([C@H]2O)O)O)(CCC=C(C)C)C)C)C)[C@@H]([C@H]([C@@H]1O)O)O[C@@H]1O[C@H](COC(=O)CC(=O)O)[C@H]([C@@H]([C@H]1O)O)O | 14162967 |
| IMPHY011084 | (1S,6S,7R)-2,6,8,8-Tetramethyltricyclo[5.2.2.01,6]undec-2-ene | CC1=CCC[C@@]2([C@@]31CC[C@@H]2C(C3)(C)C)C | 15973057 |
| IMPHY011102 | Spinacine | OC(=O)[C@@H]1Cc2nc[nH]c2CN1 | 162899 |
| IMPHY011272 | Malonyl ginsenoside Rb2 | OC[C@H]1O[C@@H](O[C@H]2CC[C@]3([C@H](C2(C)C)CC[C@@]2([C@@H]3C[C@@H](O)[C@H]3[C@@]2(C)CC[C@@H]3[C@@](O[C@@H]2O[C@H](CO[C@@H]3OC[C@@H]([C@@H]([C@H]3O)O)O)[C@H]([C@@H]([C@H]2O)O)O)(CCC=C(C)C)C)C)C)[C@@H]([C@H]([C@@H]1O)O)O[C@@H]1O[C@H](COC(=O)CC(=O)O)[C@H]([C@@H]([C@H]1O)O)O | 14162969 |
| IMPHY011454 | Gypenoside VIII | OC[C@H]1O[C@@H](O[C@H]2CC[C@]3([C@H](C2(C)C)CC[C@@]2([C@@H]3C[C@@H](O)[C@H]3[C@@]2(C)CC[C@@H]3[C@@](O[C@@H]2O[C@H](CO)[C@H]([C@@H]([C@H]2O)O)O)(CCC=C(C)C)C)C)C)[C@@H]([C@H]([C@@H]1O)O)O[C@@H]1O[C@H](CO)[C@H]([C@@H]([C@H]1O)O)O | 11679800 |
| IMPHY011548 | Falcarinol | CCCCCCC/C=CCC#CC#C[C@@H](C=C)O | 5281149 |
| IMPHY011579 | Eremophilene | CC(=C)[C@@H]1CCC2=CCC[C@@H]([C@]2(C1)C)C | 12309744 |
| IMPHY011749 | Humulene epoxide II | C/C/1=CCC(C)(C)/C=C/C[C@@]2([C@@H](CC1)O2)C | 10704181 |
| IMPHY011761 | Humulene | C/C/1=CCC(C)(C)/C=C/C/C(=C/CC1)/C | 5281520 |
| IMPHY011793 | (+)-gamma-Cadinene | CC1=C[C@@H]2[C@@H](CC1)C(=C)CC[C@H]2C(C)C | 6432404 |
| IMPHY011826 | Oleanolic acid | O[C@H]1CC[C@]2([C@H](C1(C)C)CC[C@@]1([C@@H]2CC=C2[C@@]1(C)CC[C@@]1([C@H]2CC(C)(C)CC1)C(=O)O)C)C | 10494 |
| IMPHY011957 | (+)-delta-Cadinene | CC1=C[C@@H]2C(=C(C)CC[C@H]2C(C)C)CC1 | 441005 |
| IMPHY012018 | gamma-Aminobutyric acid | NCCCC(=O)O | 119 |
| IMPHY012043 | 1,8-Heptadecadiene-4,6-diyne-3,10-diol | CCCCCCCC(C=CC#CC#CC(C=C)O)O | 4557 |
| IMPHY012050 | D-Galactose | OC[C@H]1OC(O)[C@@H]([C@H]([C@H]1O)O)O | 6036 |
| IMPHY012061 | alpha-Pinene | CC1=CCC2CC1C2(C)C | 6654 |
| IMPHY012147 | beta-Pinene | C=C1CCC2CC1C2(C)C | 14896 |
| IMPHY012151 | 2-Methyltetradecane | CCCCCCCCCCCCC(C)C | 15268 |
| IMPHY012344 | (2aS,4aR)-2,2,4a,8-tetramethyl-2a,3,4,5,6,7-hexahydro-1H-cyclobuta[i]inden-8-ol | CC1(C)CC23[C@H]1CC[C@@]3(C)CCCC2(C)O | 131039 |
| IMPHY012636 | beta-Neoclovene | C=C1CCCC2(C31CCC2C(C3)(C)C)C | 595094 |
| IMPHY012667 | Caryophyllene oxide | C=C1CC[C@H]2O[C@@]2(CC[C@@H]2[C@@H]1CC2(C)C)C | 1742210 |
| IMPHY012723 | Linolenic acid | CC/C=CC/C=CC/C=CCCCCCCCC(=O)O | 5280934 |
| IMPHY012789 | Ginsenoyne B | C=CCCCCCC(C(CC#CC#CC(C=C)O)O)Cl | 5317633 |
| IMPHY013064 | Ginsenoside Ro | OC[C@H]1O[C@@H](O[C@H]2[C@@H](O[C@@H]([C@H]([C@@H]2O)O)C(=O)O)O[C@H]2CC[C@]3([C@H](C2(C)C)CC[C@@]2([C@@H]3CC=C3[C@@]2(C)CC[C@@]2([C@H]3CC(C)(C)CC2)C(=O)O[C@@H]2O[C@H](CO)[C@H]([C@@H]([C@H]2O)O)O)C)C)[C@@H]([C@H]([C@@H]1O)O)O | 11815492 |
| IMPHY013275 | Ginsenoyne H | C=CCCCCCC1OC1CC#CC#CC(OC(=O)C)CC | 15725814 |
| IMPHY013710 | Ginsenoside M7cd | OCC1OC(OC(C2CCC3(C2C(O)CC2C3(C)CC(C3C2(C)CCC(C3(C)C)O)O)C)(CCC(C(=C)C)O)C)C(C(C1O)O)O | 131752701 |
| IMPHY014340 | 3-sec-Butyl-2-methoxy-5-methylpyrazine | CCC(c1nc(C)cnc1OC)C | 15590167 |
| IMPHY014690 | (-)-Globulol | C[C@@H]1CC[C@@H]2[C@@H]1[C@H]1[C@H](C1(C)C)CC[C@@]2(C)O | 12304985 |
| IMPHY014708 | beta-Selinene | C=C1CCC[C@]2([C@H]1C[C@@H](CC2)C(=C)C)C | 442393 |
| IMPHY014785 | (E)-2-epi-beta-caryophyllene | C/C/1=CCCC(=C)[C@H]2[C@@H](CC1)C(C2)(C)C | 6429274 |
| IMPHY014817 | Aromadendrene | CC1CCC2C1C1C(C1(C)C)CCC2=C | 91354 |
| IMPHY014831 | beta-Caryophyllene | C/C/1=CCCC(=C)[C@@H]2[C@@H](CC1)C(C2)(C)C | 5281515 |
| IMPHY014836 | beta-Sitosterol | CC[C@@H](C(C)C)CC[C@H]([C@H]1CC[C@@H]2[C@]1(C)CC[C@H]1[C@H]2CC=C2[C@]1(C)CC[C@@H](C2)O)C | 222284 |
| IMPHY014893 | D-Glucose | OC[C@H]1OC(O)[C@@H]([C@H]([C@@H]1O)O)O | 5793 |
| IMPHY014919 | D-Galacturonic Acid | OC1O[C@H](C(=O)O)[C@@H]([C@@H]([C@H]1O)O)O | 439215 |
| IMPHY014949 | Isointermedeol | CC(=C)C1CCC2(C(C1)C(C)(O)CCC2)C | 527217 |
| IMPHY014986 | Ledol | C[C@@H]1CC[C@H]2[C@@H]1[C@H]1[C@H](C1(C)C)CC[C@@]2(C)O | 92812 |
| IMPHY014990 | Linoleic acid | CCCCC/C=CC/C=CCCCCCCCC(=O)O | 5280450 |
| IMPHY015032 | Panasinsanol A | CC1(C)CC23[C@H]1CC[C@@]3(C)CCC[C@@]2(C)O | 133454 |
| IMPHY015056 | L-Rhamnose | O[C@H]1[C@H](C)OC([C@@H]([C@@H]1O)O)O | 25310 |
| IMPHY015128 | T-Muurolol | CC1=C[C@@H]2[C@H](CC1)[C@@](C)(O)CC[C@H]2C(C)C | 3084331 |
| IMPHY015263 | 2-Heptylfuran | CCCCCCCc1ccco1 | 19603 |
| IMPHY016012 | Allo-Aromadendrene | C[C@@H]1CC[C@H]2[C@@H]1C1C(C1(C)C)CCC2=C | 42608158 |
| IMPHY016919 | Ginsenoside Rb2 | OC[C@H]1O[C@@H](O[C@H]2CC[C@]3([C@H](C2(C)C)CC[C@@]2([C@@H]3C[C@@H](O)[C@H]3[C@@]2(C)CC[C@@H]3[C@@](O[C@@H]2O[C@H](CO[C@@H]3OC[C@@H]([C@@H]([C@H]3O)O)O)[C@H]([C@@H]([C@H]2O)O)O)(CCC=C(C)C)C)C)C)[C@@H]([C@H]([C@@H]1O)O)O[C@@H]1O[C@H](CO)[C@H]([C@@H]([C@H]1O)O)O | 6917976 |
| IMPHY001560 | 1-Heptadecanol | CCCCCCCCCCCCCCCCCO | 15076 |
| IMPHY003618 | Ginsenoside Rg3 | OC[C@H]1O[C@@H](O[C@H]2CC[C@]3([C@H](C2(C)C)CC[C@@]2([C@@H]3C[C@@H](O)[C@H]3[C@@]2(C)CC[C@@H]3[C@](CCC=C(C)C)(O)C)C)C)[C@@H]([C@H]([C@@H]1O)O)O[C@@H]1O[C@H](CO)[C@H]([C@@H]([C@H]1O)O)O | 9918693 |
| IMPHY003983 | Ginsenoside rb1 | OC[C@H]1O[C@@H](O[C@H]2CC[C@]3([C@H](C2(C)C)CC[C@@]2([C@@H]3C[C@@H](O)[C@H]3[C@@]2(C)CC[C@@H]3[C@@](O[C@@H]2O[C@H](CO[C@@H]3O[C@H](CO)[C@H]([C@@H]([C@H]3O)O)O)[C@H]([C@@H]([C@H]2O)O)O)(CCC=C(C)C)C)C)C)[C@@H]([C@H]([C@@H]1O)O)O[C@@H]1O[C@H](CO)[C@H]([C@@H]([C@H]1O)O)O | 9898279 |
| IMPHY004040 | Ginsenoside Rh2 | OC[C@H]1O[C@@H](O[C@H]2CC[C@]3([C@H](C2(C)C)CC[C@@]2([C@@H]3C[C@@H](O)[C@H]3[C@@]2(C)CC[C@@H]3[C@](CCC=C(C)C)(O)C)C)C)[C@@H]([C@H]([C@@H]1O)O)O | 119307 |
| IMPHY004045 | Ginsenoside RG2 | OC[C@H]1O[C@@H](O[C@H]2C[C@]3(C)[C@@H]([C@@]4([C@@H]2C(C)(C)[C@@H](O)CC4)C)C[C@H]([C@H]2[C@@]3(C)CC[C@@H]2[C@](CCC=C(C)C)(O)C)O)[C@@H]([C@H]([C@@H]1O)O)O[C@@H]1O[C@@H](C)[C@@H]([C@H]([C@H]1O)O)O | 21599924 |
| IMPHY004187 | L-(+)-Arabinose | OC[C@@H]([C@@H]([C@H](C=O)O)O)O | 5460291 |
| IMPHY004235 | D-Glucuronic Acid | OC1O[C@H](C(=O)O)[C@H]([C@@H]([C@H]1O)O)O | 94715 |
| IMPHY004273 | Ginsenoside RG1 | OC[C@H]1O[C@@H](O[C@H]2C[C@]3(C)[C@@H]([C@@]4([C@@H]2C(C)(C)[C@@H](O)CC4)C)C[C@H]([C@H]2[C@@]3(C)CC[C@@H]2[C@@](O[C@@H]2O[C@H](CO)[C@H]([C@@H]([C@H]2O)O)O)(CCC=C(C)C)C)O)[C@@H]([C@H]([C@@H]1O)O)O | 441923 |
| IMPHY004275 | Ginsenoside-rgl | OCC1OC(O[C@H]2C[C@]3(C)[C@@H]([C@@]4([C@@H]2C(C)(C)[C@@H](O)CC4)C)C[C@H]([C@H]2[C@@]3(C)CCC2[C@@](OC2OC(CO)C(C(C2O)O)O)(CCC=C(C)C)C)O)C(C(C1O)O)O | 24728657 |
| IMPHY004388 | Kaempferol | Oc1ccc(cc1)c1oc2cc(O)cc(c2c(=O)c1O)O | 5280863 |
| IMPHY004812 | Ginsenoside Rf | OC[C@H]1O[C@@H](O[C@H]2C[C@]3(C)[C@@H]([C@@]4([C@@H]2C(C)(C)[C@@H](O)CC4)C)C[C@H]([C@H]2[C@@]3(C)CC[C@@H]2[C@](CCC=C(C)C)(O)C)O)[C@@H]([C@H]([C@@H]1O)O)O[C@@H]1O[C@H](CO)[C@H]([C@@H]([C@H]1O)O)O | 441922 |
| IMPHY006971 | Methyl palmitate | CCCCCCCCCCCCCCCC(=O)OC | 8181 |
| IMPHY007322 | ginsenoside F2 | OC[C@H]1O[C@@H](O[C@H]2CC[C@]3([C@H](C2(C)C)CC[C@@]2([C@@H]3C[C@@H](O)[C@H]3[C@@]2(C)CC[C@@H]3[C@@](O[C@@H]2O[C@H](CO)[C@H]([C@@H]([C@H]2O)O)O)(CCC=C(C)C)C)C)C)[C@@H]([C@H]([C@@H]1O)O)O | 9918692 |
| IMPHY007327 | Palmitic acid | CCCCCCCCCCCCCCCC(=O)O | 985 |
| IMPHY007366 | alpha-Santalene | CC(=CCCC1(C)C2CC3C1(C)C3C2)C | 94164 |
| IMPHY008305 | 2-Heptadecanone | CCCCCCCCCCCCCCCC(=O)C | 18027 |
[truncated: 409,276 more chars]
